# Dutch multidisciplinary guideline on anterior knee pain: Patellofemoral pain and patellar tendinopathy

**DOI:** 10.1002/ksa.12367

**Published:** 2024-07-24

**Authors:** Martin Ophey, Sander Koëter, Lianne van Ooijen, Mathijs van Ark, Fred Boots, Shanna Ilbrink, Nienke A. Lankhorst, Tom Piscaer, Myrthe Vestering, Mirre den Ouden Vierwind, Robbart van Linschoten, Sietske van Berkel

**Affiliations:** ^1^ IJsveldFysio – Private Physiotherapy Clinic Nijmegen The Netherlands; ^2^ Department of Orthopaedic Surgery and Sports Medicine, Amsterdam UMC Location University of Amsterdam Amsterdam The Netherlands; ^3^ Orthopaedic Surgery Canisius Wilhelmina Hospital Nijmegen The Netherlands; ^4^ Profysic – Private Clinic for Sport Podiatry Eindhoven The Netherlands; ^5^ Physiotherapy Department Hanze University of Applied Sciences Groningen The Netherlands; ^6^ Centre of Expertise Primary Care (ECEZG) Groningen The Netherlands; ^7^ Boots Solide Werken Gorinchem The Netherlands; ^8^ Jessica Gal Sportartsen, Amsterdam & Sport‐ en Beweegkliniek Haarlem The Netherlands; ^9^ Independent General Practitioner The Hague The Netherlands; ^10^ Department of Orthopaedics and Sports Medicine Erasmus MC Rotterdam The Netherlands; ^11^ Department of Radiology Gelderse Vallei Hospital Ede The Netherlands; ^12^ Knowledge Institute of the Dutch Association of Medical Specialists Utrecht The Netherlands; ^13^ Region Nordjylland Sportsmedicinsk Klinik Frederikshavn Denmark; ^14^ Department of Sports Medicine Isala Hospital Zwolle The Netherlands

**Keywords:** conservative treatment, knee, knee joint, patellofemoral pain syndrome, tendinopathy

## Abstract

**Purpose:**

The purpose of this study was to develop a multidisciplinary guideline for patellofemoral pain (PFP) and patellar tendinopathy (PT) to facilitate clinical decision‐making in primary and secondary care.

**Methods:**

A multidisciplinary expert panel identified questions in clinical decision‐making. Based on a systematic literature search, the strength of the scientific evidence was determined according to the Grading of Recommendations, Assessment, Development and Evaluations (GRADE) method and the weight assigned to the considerations by the expert panel together determined the strength of the recommendations.

**Results:**

After confirming PFP or PT as a clinical diagnosis, patients should start with exercise therapy. Additional conservative treatments are indicated only when exercise therapy does not result in clinically relevant changes after six (PFP) or 12 (PT) weeks. Pain medications should be reserved for cases of severe pain. The additional value of imaging assessments for PT is limited. Open surgery is reserved for very specific cases of nonresponders to exercise therapy and those requiring additional conservative treatments. Although the certainty of evidence regarding exercise therapy for PFP and PT had to be downgraded (‘very low GRADE’ and ‘low GRADE’), the expert panel advocates its use as the primary treatment strategy. The panel further formulated weaker recommendations regarding additional conservative treatments, pain medications, imaging assessments and open surgery (‘very low GRADE’ to ‘low GRADE’ assessment or absence of scientific evidence).

**Conclusion:**

This guideline recommends starting with exercise therapy for PFP and PT. The recommendations facilitate clinical decision‐making, and thereby optimizing treatment and preventing unnecessary burdens, risks and costs to patients and society.

**Level of Evidence:**

Level V, clinical practice guideline.

**Abbreviations**
AGREE II: Appraisal of Guidelines for Research and Evaluation II
AKPS: Anterior Knee Pain Scale
GRADE: Grading of Recommendations, Assessment, Development and Evaluations
NPF: Dutch Patient Federation (Nederlandse Patienten Federatie)
NSAID: nonsteroidal anti‐inflammatory drug
OA: osteoarthritis
PFP: patellofemoral pain
PT: patellar tendinopathy
RCT: randomized controlled trial

## INTRODUCTION

Anterior knee pain is one of the most common presentations of knee pain affecting adolescents and adults, particularly in physically active populations [39, 47]. In 2010, the Netherlands Association of Sports Medicine published a monodisciplinary guideline for patellofemoral pain (PFP) [7]. Since then, progress in the scientific literature (e.g., consensus statements and other monodisciplinary guidelines) [10, 56] has led to an increased awareness of the need for a comprehensive multidisciplinary approach with the involvement of physical therapists, podiatrists and orthopaedic surgeons. Furthermore, the previous guideline focused only on PFP; patellar tendinopathy (PT) was not covered.

Although PFP and PT appear to have an unfavourable long‐term prognosis [26, 27, 31], no multidisciplinary evidence‐based guidelines exist. In clinical practice, patients should be advised about optimal treatment and timelines. Moreover, in addition to scientific evidence, patient expectations and values are important to include in treatment guidelines. Therefore, a clinical approach for treating anterior knee pain based on evidence and patient information is necessary.

This study aimed to develop a multidisciplinary evidence‐based guideline to facilitate clinical decision‐making, diagnosis and treatment of PFP and PT for healthcare providers involved in the treatment of these patients and working in primary and secondary care.

## METHODS

### Terminology

According to the 2016 Manchester statement, PFP is the most preferred term and is defined as ‘pain around or behind the patella, which is aggravated by at least one activity that loads the patellofemoral joint during weight bearing on a flexed knee’ [12]. PT is characterized by tenderness on palpation and load‐dependent pain at the inferior pole of the patella [46]. According to the 2019 International Scientific Tendinopathy Symposium Consensus, ‘tendinopathy is the preferred term for persistent tendon pain and loss of function related to mechanical loading’ [46]. Consistency in the use of terms and definitions in communication between healthcare providers and patients reduces unnecessary confusion and increases the therapeutic alliance [5]. Therefore, in this guideline, the terms ‘patellofemoral pain’ and ‘patellar tendinopathy’ are used.

### Panel selection and scoping questions

**Figure 1:**
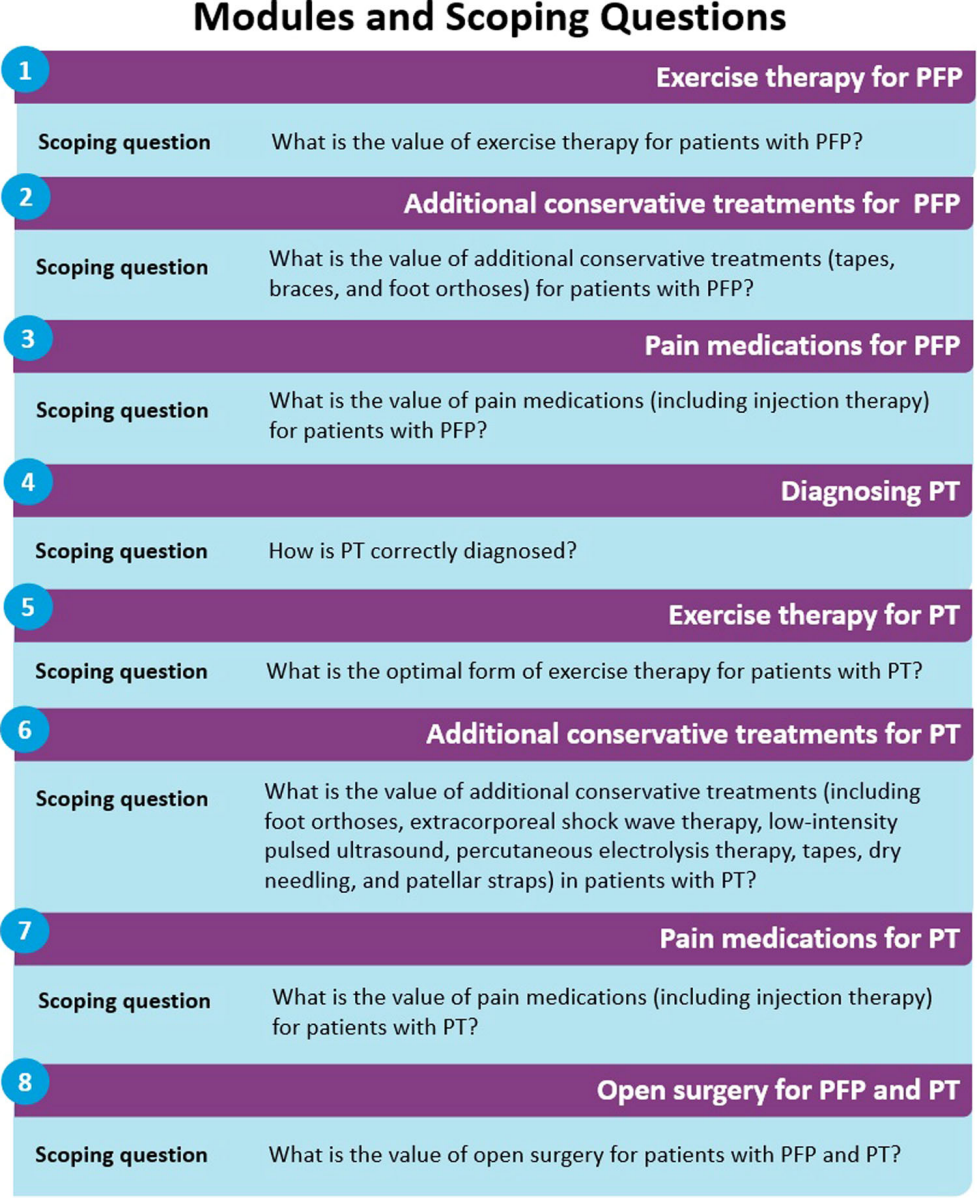
Modules and Scoping Questions. Overview of the eight distinct guideline Modules and Scoping Questions for PFP and PT. PFP, patellofemoral pain; PT, patellar tendinopathy.

In 2019, as part of the preparatory phase of the guideline process, an expert panel was commissioned by the Netherlands Association of Sports Medicine to update and expand the existing 2010 guideline. The panel consisted of representatives from all relevant healthcare disciplines (general practice, occupational medicine, sports medicine, orthopaedic surgery, radiology, podiatry and physical therapy) involved in treating patients with anterior knee pain. The panel received methodological support from the Knowledge Institute of the Dutch Association of Medical Specialists. More information about the selection of the expert panel and declaration of interest is provided in the online Supporting Information. Additionally, a written invitational conference was organized and the involved medical associations were invited to suggest clinical questions for the optimal care of patients with anterior knee pain. The patient's perspective was clarified through a survey of patients with PFP and PT for inclusion in the recommendations. In cooperation with the panel, the Dutch Patient Federation (Nederlandse Patienten Federatie [NPF]) developed an online survey assessing patient characteristics, pros and cons, and levels of satisfaction with treatment options in primary and secondary care. Data were collected from patients with PFP and PT, between January and April 2020. Based on the results of this preparatory phase, eight major clinical questions were prioritized. These clinical questions addressed uncertainties related to diagnosing PT, the value of exercise therapy including additional conservative treatments, the role of pain medications and indications for open surgery regarding PFP and PT. Each clinical question was developed within distinct guideline Modules and Scoping Questions (Figure 1). After presenting the scoping question, each module contains relevant background, results of the evidence review, rationale and, finally, the recommendations.

Other conditions that can result in anterior knee pain, such as specific intraarticular pathologies (chondral defects, patellofemoral osteoarthritis [OA]), patellar instability or recurrent patellar dislocation, fractures, tendinopathies other than PT, apophysitis, bursitis or referred pain from the hip, were not covered.

### Evidence review

Regarding the eight predefined modules and scoping questions (Figure 1), a detailed description of the systematic literature search and selection strategy, as well as the assessment of the risk of bias of individual studies, can be found under ‘Construction of modules’ in the module section in the online Supporting Information. This guideline was developed based on the Appraisal of Guidelines for Research and Evaluation II (AGREE II) [8]. The Grading of Recommendations, Assessment, Development and Evaluations (GRADE) method was used to rate the certainty of the evidence (Table 1).

**Table 1:** GRADE classification [25].

GRADE	Definition
High	There is high confidence that the true effect of treatment is close to the estimated effect of treatment; it is very unlikely that the literature conclusion will change clinically relevant when results of new large‐scale research are added to the literature analysis.
Moderate	There is reasonable assurance that the true effect of treatment is close to the estimated effect of treatment; it is possible that the conclusion will change in a clinically relevant way when the results of new large‐scale studies are added to the literature analysis.
Low	There is low certainty that the true effect of treatment is close to the estimated effect of treatment; there is a real chance that the conclusion will change in a clinically relevant way when results of new large‐scale research are added to the literature analysis.
Very low	There is very low certainty that the true effect of treatment is close to the estimated effect of treatment; the literature conclusion is very uncertain.

Abbreviation: GRADE, Grading of Recommendations, Assessment, Development and Evaluation.

The strength of the scientific evidence and the weight assigned to the considerations by the expert panel together determine the strength of the recommendations. In accordance with the GRADE method, a low GRADE value of conclusions in the systematic literature analysis does not preclude a strong recommendation a priori; weak recommendations are also possible with a high GRADE value [25, 44]. Moreover, the strength of a recommendation is always determined by weighing all the relevant arguments [25]. The evidence review and study selection for each module are presented in the online Supporting Information.

### Consensus process

The expert panel considered pain and function as critical outcome measures and return to sports/work, duration of absenteeism and patient satisfaction as important outcome measures for decision‐making. The expert panel met in person and online, and the panel chair (SvB) ensured that all members had equal and uninhibited votes. Every module, including the final recommendations, was discussed with all members of the expert panel to create a unanimous recommendation. During the commentary and authorization phases, the draft guideline was submitted to all medical associations involved, as well as the NPF and other relevant stakeholders. These associations provided comments that were viewed and processed by the expert panel. The final version of the guideline was approved by all involved medical associations. The literature conclusions, considerations and summaries with tables and/or figures per module are presented in the online Supporting Information.

## RESULTS

### Patient perspective

Fifty‐two patients with PFP and PF responded to the NPF survey (Table 2). Thirty‐eight participants, comprising 73% of the total, were women. Thirty‐nine (75%) participants reported experiencing anterior knee pain for more than 12 months. Not all treatment options were offered to all patients. Notably, not all patients exercise therapy mentioned as a (first) treatment option. Furthermore, 12 (40%) patients with PFP reported dissatisfaction with the results of exercise therapy, and among these, three (25%) patients reported an increase in knee pain.

**Table 2:** Characteristics of survey participants.

	PFP (*n* = 43)	PT (*n* = 9)
Gender		
Women	34 (79%)	4 (44%)
Men	9 (21%)	5 (56%)
Age		
12–18 years	4 (9%)	1 (11%)
19–30 years	24 (56%)	7 (78%)
31–40 years	6 (14%)	0
41–65 years	9 (21%)	1 (11%)
Time since diagnosis		
<3 months	3 (7%)	2 (22%)
3–12 months	7 (16%)	1 (11%)
1–5 years	23 (54%)	5 (56%)
>5 years	10 (23%)	1 (11%)
Offered treatment options		
Rest	22 (51%)	7 (78%)
Exercise therapy	28 (65%)	8 (89%)
Tapes/braces/foot orthoses/patellar strap	34 (79%)	5 (56%)
Pain medications	14 (33%)	3 (33%)
Surgery	9 (21%)	1 (9%)
Other (e.g., dry needling)	16 (37%)	5 (56%)
Satisfied and very satisfied with treatment result^a^		
Rest	5/18 (28%)	2/7 (29%)
Exercise therapy	15/30 (50%)	5/9 (56%)
Tapes/braces/foot orthoses/patellar straps	13/25 (52%)	2/6 (33%)
Pain medications	3/13 (23%)	0
Surgery	9/13 (69%)	1/1 (100%)
Other (e.g., dry needling)	7/7 (100%)	2/4 (50%)

*Note*: Data are numbers (percentages).

Abbreviations: *n*, numbers; PFP, patellofemoral pain; PT, patellar tendinopathy.

^a^ 5‐Point Likert Scale: (1) *very unsatisfied*, (2) *unsatisfied*, (3) *neutral*, (4) *satisfied*, (5) *very satisfied*. Not all treatment options were offered to all patients.

### Recommendations

The systematic search results, included studies, GRADE levels of evidence, meta‐analyses and the expert panel's rationale for recommendations can be found in the online Supporting Information.

#### Module 1: ‘Exercise therapy for PFP’

Scoping question: What is the value of exercise therapy for patients with PFP?

Background: In daily practice, not all patients with PFP are advised to perform exercises under the supervision of a physical therapist. Additionally, the types, series, repetitions, criteria (pain/fatigue) and durations of exercise therapy programmes (including patient education) under the supervision of physical therapists can vary greatly.

Evidence review: One systematic review and three additional randomized controlled trials (RCTs) were included (‘low GRADE’) [21, 24, 41].

Rationale: Despite the low level of evidence, the expert panel advocates for the initiation of exercise therapy for patients with PFP [partly] under the supervision of a physical therapist. The characteristics of exercise therapy programmes in the included studies differed widely, as did the characteristics of the patients with PFP in clinical practice. The expert panel recommends that exercise therapy programmes be tailored to individual patient characteristics.

Recommendations:

- PFP should be treated with quadriceps‐ and/or hip‐focused exercise therapy for at least 6 to 12 weeks, preferably [partly] under the supervision of a physical therapist.
- A structured exercise therapy programme (volume and intensity), which continuously adjusts to the patient's pain intensity, should be offered.
- Patient education should be prioritized.
- Progression (volume and intensity), pain (Visual Analogue Scale) and function (Kujala Anterior Knee Pain Scale [AKPS]) should be evaluated after 6 and 12 weeks of performing the exercise programme in a structured manner.
- Additional conservative treatments should be considered when, after 6 weeks of exercise therapy, no clinically relevant changes occur (Module 2: ‘Additional conservative treatments for PFP’).
- The diagnosis of PFP should be reconsidered if no improvement in pain and function occurs after 12 weeks of structured quadriceps‐ and/or hip‐focused exercise therapy.

#### Module 2: ‘Additional conservative treatments for PFP’

Scoping question: What is the value of additional conservative treatments (tapes, braces and foot orthoses) for patients with PFP?

Background: Various conservative treatments are commonly used in the management of PFP. Usually, these treatments are adjuncts to exercise therapy (multimodal treatment). However, their additional benefits remain unclear. The three most commonly used additional conservative treatments in the Netherlands are tapes, braces and foot orthoses.

Evidence review: Three systematic reviews (including six RCTs) and eight additional RCTs were included (‘very low GRADE’) [1, 2, 6, 9, 19, 20, 23, 33, 40, 48, 51].

Rationale: Evidence supporting the regular application of tapes, braces and foot orthoses is lacking or conflicting. Therefore, the expert panel advocates for the application of these treatments only in specific cases.

Recommendations:

- Initially, PFP should be treated using exercise therapy for 6 to 12 weeks (Module 1: ‘Exercise therapy for PFP’).
- Tapes, braces, or foot orthoses should be considered as additional conservative treatments in combination with exercise therapy if no clinically relevant changes occur after 6 to 12 weeks of exercise therapy (Module 1: ‘Exercise therapy for PFP’).
- The patient should be informed of the lack of evidence regarding these additional conservative treatments and should be involved in decision‐making regarding the choice of additional treatments.

#### Module 3: ‘Pain medications for PFP’

Scoping question: What is the value of pain medications (including injection therapy) for patients with PFP?

Background: Some patients try a short course of paracetamol (acetaminophen) or nonsteroidal anti‐inflammatory drugs (NSAIDs) before visiting a (sports medicine‐) physician or physical therapist. Injections of corticosteroids or hyaluronic acid are mainly used to treat adults with symptomatic OA; however, the literature suggests that they may also positively affect pain in patients with PFP. Other forms of injection therapies used in clinical practice include autologous platelets (platelet‐rich plasma, PRP) and prolotherapy (glucose). However, whether these emerging interventions are effective in patients with PFP remains unclear.

Evidence review: One systematic review (including three RCTs) was included (‘very low GRADE’ to ‘low GRADE’) [22].

Rationale: The expert panel advocates discussing pain management and the use of pain medications during consultations. Oral pain medications for short‐term pain relief are indicated only in cases of severe pain. It is advisable to start treatment with paracetamol (acetaminophen) because of its safety profile. A (topical) NSAID is the second choice. This is in line with the Dutch recommendations for the management of knee OA [15]. Because the body of knowledge on injection therapy is small or absent, the expert panel sees no role of injection therapy in the management of patients with PFP.

Recommendations:

- Caution should be exercised in advising any type of pain medication for PFP.
- PFP should not be treated with injection therapy.

#### Module 4: ‘Diagnosing PT’

Scoping question: How is PT correctly diagnosed?

Background: The diagnosis of PT is mainly based on patient history and physical examination. Patients typically report local load‐dependent pain at the inferior pole of the patella [43]. PFP and OA are common differential diagnoses. The diagnostic accuracy of patient history and physical examination, as well as the additional value of imaging assessments, remains unclear.

Evidence review: Two studies on physical examinations were included (‘very low GRADE’) [34, 35].

Rationale: Owing to the absence of literature on the diagnostic value of patient history, recommendations are based on a consensus paper [46] and the opinions of the expert panel. When considering additional imaging assessment, diagnostic ultrasound emerges as the most suitable and readily accessible technique.

Recommendations:

- The following criteria should be used for a correct clinical diagnosis of PT:
  o Load‐dependent pain at the inferior pole of the patella; and …
    ▪ … a positive palpation test for pain at the inferior patellar pole and/or …
    ▪ … a positive Royal London hospital test and/or …
    ▪ … pain at the inferior patellar pole during the single leg decline squat.
- Additional imaging assessments should be undertaken as a confirmatory test when uncertainty remains regarding the correct clinical diagnosis of PT or in the absence of clinically relevant changes after 12 weeks of exercise therapy (Module 5: ‘Exercise therapy for PT’).
- The patient should be informed of the lack of evidence regarding additional imaging assessments and the possible extra financial burden.*

#### Module 5: ‘Exercise therapy for PT’

Scoping question: What is the optimal form of exercise therapy for patients with PT?

Background: Treatment of PT using exercise therapy is the most substantiated treatment in the literature; when an adjunct conservative treatment is chosen, it is regularly combined with exercise therapy. However, there is ambiguity regarding the type of exercise therapy that should be administered. Moreover, there is limited knowledge about the effectiveness of different exercise therapy programmes; frequency, intensity, type and time factors; and duration of continued exercise therapy after return to activity (sports/work).

The aim of this module was to elucidate the effectiveness of specific exercise therapy in patients with PT compared to other types of specific exercise therapy or a control group.

Evidence review: Five RCTs were included (‘very low GRADE’) [3, 14, 17, 29, 52].

Rationale: Despite the low level of evidence, the expert panel advocates for the use of exercise therapy as the first choice for patients with PT. Although there is no ‘one size fits all plan’, the expert panel suggests the use of a four‐stage treatment plan based on Rudavsky and Cook (2014) [43].

**Figure 2:**
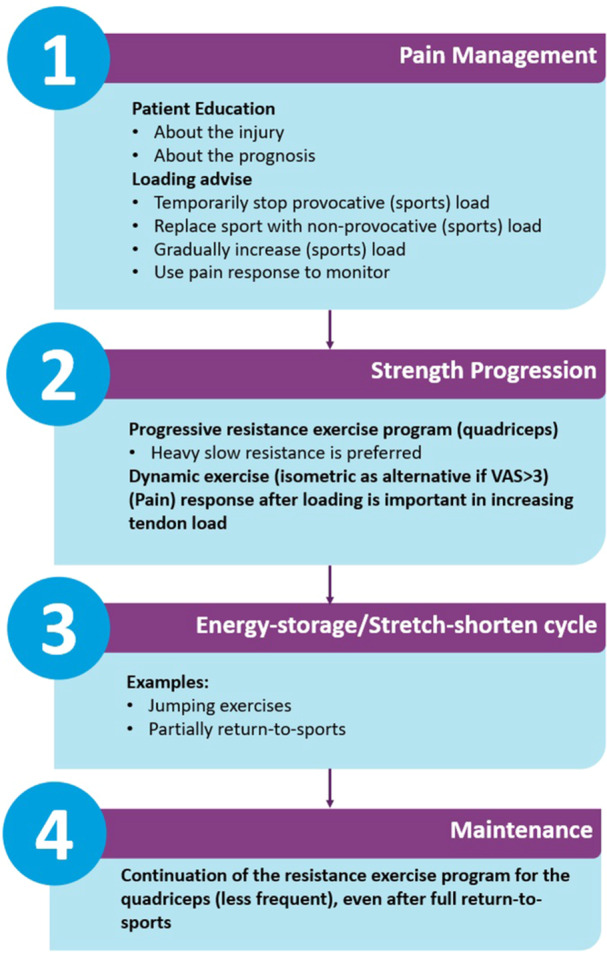
Four‐step‐treatment‐plan for PT (adjusted from Rudavsky and Cook [43]). Structured treatment plan for PT starting with adequate pain management, followed by strength progression and restoration of the tendon's energy‐storage capacity, and concluded with maintenance strategies. PT, patellar tendinopathy; VAS, Visual Analogue Scale.

Recommendations:

- Use the following four‐step‐treatment‐plan to treat patients with PT(Figure 2):
  1. Pain management: Patient education and loading advice.
  2. Strength progression:
    ▪ A progressive resistance exercise programme for the quadriceps should be performed for at least 12 weeks while continuously adjusting exercise intensity for the individual patient.
    ▪ Heavy slow resistance training is preferred, although different types of training programmes/contractions (e.g., isometric contractions) may be used to minimize the reactive pain response of the patellar tendon.
  3. Energy storage/stretch‐shorten cycle: Depending on the type of sport, start with plyometric exercises.
  4. Maintenance of muscle strength.
- Additional conservative treatments should be considered when, after 12 weeks of thorough exercise therapy (including education and loading advice), no clinically relevant changes occur (Module 6: ‘Additional conservative treatments for PT’ and Module 7: ‘Pain medications for PT’).

#### Module 6: ‘Additional conservative treatments for PT’

Scoping question: What is the value of additional conservative treatments (including foot orthoses, extracorporeal shock wave therapy, low‐intensity pulsed ultrasound, percutaneous electrolysis therapy, tapes, dry needling and patellar straps) in patients with PT?

Background: There are numerous additional conservative treatments for PT; however, the additional value of these treatments remains unclear.

Evidence review: Six RCTs were included (‘very low GRADE’) [32, 50, 54, 55, 57, 58]. No literature was found on the effectiveness of foot orthoses, percutaneous electrolysis therapy or dry needling.

Rationale: Evidence supporting the regular application of these additional treatments is lacking. Therefore, the expert panel advocates for the application of these treatments only in specific cases. If no clinically relevant changes occur after exercise therapy for 12 weeks (Module 5: ‘Exercise therapy for PT’), the expert panel prefers the ‘patellar straps’ due to their low cost and ease of use.

Recommendations:

- Initially, PT should be treated with exercise therapy (including education and loading advice) (Module 5: ‘Exercise therapy for PT’).
- Additional conservative treatments (‘patellar straps’) should be considered when no clinically relevant changes occur after 12 weeks of exercise therapy (Module 5: ‘Exercise therapy for PT’).
- The patient should be informed of the lack of evidence regarding these additional conservative treatments and should be involved in decision‐making regarding the choice of additional treatments.

#### Module 7: ‘Pain medications for PT’

Scoping question: What is the value of pain medications (including injection therapy) for patients with PT?

Background: Physicians sometimes prescribe oral or local medications to reduce pain. When conservative treatment fails, injection therapy is sometimes administered. The Dutch guideline on Achilles tendinopathy emphasizes the uncertainties regarding the effectiveness of injection therapy and assumes that exercise therapy is effective after 1 year [53].

Evidence review: Three RCTs were included (‘very low GRADE’ to ‘low GRADE’) [42, 45, 49].

Rationale: The expert panel advocates discussing pain management and the use of pain medications during consultations. Oral pain medications for short‐term pain relief are indicated only in cases of severe pain. Because of these side effects, paracetamol (acetaminophen) is the first choice, whereas a (topical) NSAID is the second choice. This is in line with the Dutch recommendations for the management of knee OA [15]. While using prescribed medications, patients should be aware of medication dependence and/or tendon overuse due to pain suppression. Owing to the small or absent body of evidence, the expert panel sees no role for injection therapy in the management of patients with PT.

Recommendations:

- Caution should be exercised in advising any type of pain medication for PT.
- PT should not be treated with injection therapy.

#### Module 8: ‘Open surgery for PFP and PT’

Scoping question: What is the value of open surgery for patients with PFP and PT?

Background: When PFP and PT are unresponsive to conservative treatments, patients are sometimes referred to an orthopaedic surgeon for evaluation, confirmation of diagnosis, potential supplementary diagnostics and appraisal of indications for surgical treatment. The Dutch Guideline on Knee Arthroscopy recommends not performing arthroscopy for PFP and exercising caution in performing arthroscopy for PT [4, 28]. However, the role of open surgery in the surgical treatment of PFP and PT remains unclear.

Evidence review: No studies have been conducted on open surgery for PFP. One systematic review of open surgery for PT was included (‘low GRADE’) [13].

Rationale: Recommendations of the expert panel were based solely on clinical expertise. Imaging assessments (magnetic resonance imaging or computed tomography) are obligatory and should be evaluated for post‐traumatic, degenerative, or congenital morphological causes of PFP. When open surgery for PT is indicated, the expert panel recommends magnetic resonance imaging assessment to confirm the exact location and degree of patellar tendon abnormalities before surgery.

Recommendations:

- Initially, PFP and PT should only be treated with non‐operative treatments for at least 6 months (see Module 2: ‘Exercise therapy for PFP’, Module 3: ‘Additional conservative treatments for PFP’, Module 5: ‘Exercise for PT’ and Module 6: ‘Additional conservative treatments for PT’).
- Surgery should only be considered when conservative treatment fails; the variable and unpredictable outcomes and potential risks of open surgery for PFP and PT should be discussed, and shared decision‐making should be employed when choosing the treatment.
- Open surgery should be considered only when, following at least 6 months of non‐operative treatments, including exercise therapy, the patient continues to experience persistent and disabling symptoms and functional limitations, and
  o Anatomical morphology is determined to be the cause of persistent PFP confirmed by imaging assessments or
  o PT is confirmed by imaging assessments.

## DISCUSSION

**Figure 3:**
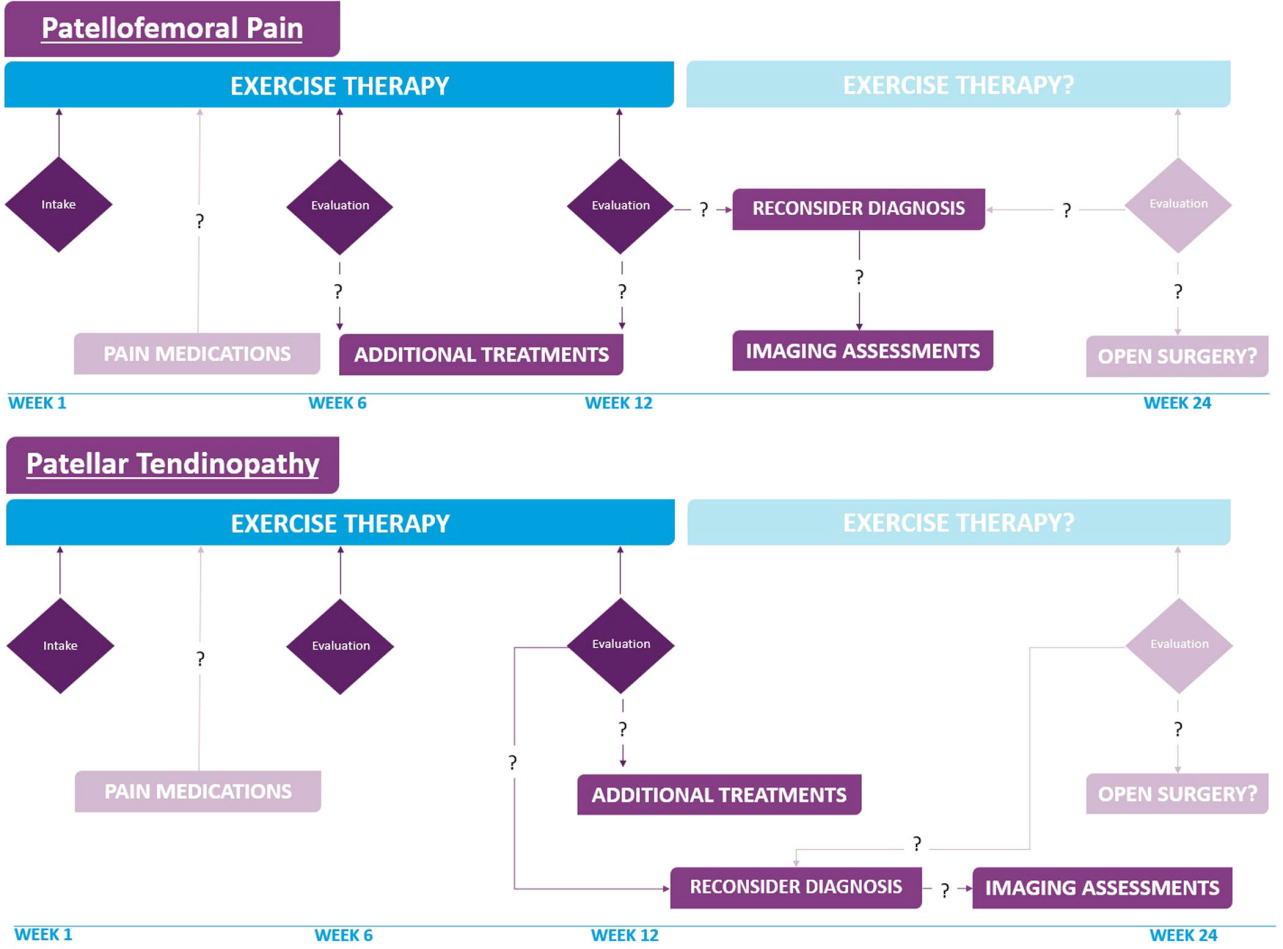
Evidence‐based clinical approaches for PFP and PT. Optimal treatment and timelines with exercise therapy as the cornerstone in the first 6 and 12 weeks for PFP and PT, respectively. Pain medications may play a role in this first part in cases with severe pain, with additional conservative treatments only when exercise therapy does not result in clinically relevant changes. Due to the limited additional value of imaging assessments for PT, its use should be considered only after 12 weeks. Reconsider diagnosis after 12 weeks (PFP and PT) and open surgery is reserved for very specific cases only. PFP, patellofemoral pain; PT, patellar tendinopathy.

After ruling out other clinical conditions and confirming PFP or PT as a clinical diagnosis, this guideline recommends starting with exercise therapy preferably [partly] under the supervision of a physical therapist and following this treatment for 6 (PFP) to 12 (PT) weeks. Additional conservative treatments are only indicated when exercise therapy does not result in clinically relevant changes and pain medications should be reserved for exceptional cases of severe pain. The additional value of imaging assessments for PT is limited. Open surgery is reserved for very specific cases, which do not respond to exercise therapy or additional conservative treatments (Figure 3).

An innovative aspect of this guideline is that criteria and explicit timelines have been established to clarify when clinically relevant changes can and should be expected. The expert panel delineated clinically relevant changes as measurable changes on accepted patient‐reported outcome measures, including a minimum of 10 points on the AKPS [11] or 16 points on the Victorian Institute of Sport Assessment Scale‐Patella [30]. The limited additional value of other conservative treatments, pain medications, imaging assessments and open surgery has been highlighted to support shared decision‐making. A systematic approach to making clinical practice recommendations was adopted using the AGREE II instrument and GRADE method [8, 25, 44]. The process of development of this guideline revealed relevant knowledge gaps, such as the parameters of exercise therapy and the added value of additional conservative treatments, medications and open surgery. An in‐depth discussion of the results for each scoping question can be found in the online Supporting Information.

Although the certainty of the evidence regarding exercise therapy for the treatment of PFP and PT had to be downgraded due to risk of bias (study limitations), imprecision (number of included patients) or heterogeneity (inconsistencies), the expert panel advocates for the use of exercise therapy as the primary treatment strategy. This finding is consistent with recent consensus statements and clinical practice guidelines [10, 56]. The expert panel aimed to inform more healthcare providers, as well as patients with PFP or PT, of the value of exercise therapy as the first choice for treatment.

Because most of the included studies did not describe all exercise therapy parameters, reproducibility of the exact treatment protocols and optimal treatment in clinical practice is arduous. One‐quarter of the PFP respondents who reported dissatisfaction with the results of exercise therapy reported an increase in knee pain. Remarkably, only one RCT in patients with PFP stated explicitly that ‘no self‐reported increase in usual pain after the training session or the next morning’ is allowed [41]. For patients with PT, pain during or after exercise therapy was better covered in the included RCTs [3, 14, 17, 29, 52].

The expert panel advocates for the use of paracetamol (acetaminophen) as the first choice of pain medication in select cases of patients with PFP and PT; however, no evidence exists regarding the effectiveness of this specific drug and no evidence for the use of this drug during training is available. Although the expert panel currently sees no role for injection therapy, including cell therapies, in the management of patients with PT, available evidence suggests that this perspective may change in the future with the emergence of RCTs featuring larger sample sizes and long‐term follow‐ups [36, 37].

Patients with PFP or PT also reported receiving a whole range of conservative treatments (additional to exercise therapy or as stand‐alone treatments), while our literature research regarding these treatments concluded that scientific evidence for these additional treatments is either absent or had a ‘very low GRADE’ to ‘low GRADE’ (online Supporting Information modules ‘Additional conservative treatments’ and ‘Pain medications’ for PFP and PT). Therefore, the expert panel formulated weaker recommendations regarding these interventions. Despite the absence of scientific evidence for open surgery, the expert panel felt an urgent need to formulate a cautious but decisive recommendation for an orthopaedic assessment to ascertain the indication for open surgery. This is reserved for nonresponders to diligent exercise therapy or exercise therapy plus additional conservative treatments for at least six months. In cases of distinct anatomical pathology, such as central patellar tendon necrosis, more prompt surgical intervention may be warranted.

This guideline has been accessible since 9 May 2022 on the database website of the Dutch Association of Medical Specialists [16]. Furthermore, a ‘patient version’ developed by the expert panel is available online [38]. The guideline will be partially (scoping questions and modules) or entirely evaluated and updated after 2027. Additional modules with other scoping questions can be added to the guideline at later stages.

The patient perspective was guaranteed by the involvement of the NPF during the preparatory, commentary and authorization phase of the guideline and clarified by means of the NPF survey. However, the patients or their representatives did not participate in the panel meetings. Furthermore, the voice of patients with PT may be underrepresented in the NPF survey since only nine patients with PT compared to 43 patients with PFP completed the survey. Additionally, socioeconomic backgrounds or other characteristics of the participants of the NPF survey indicating marginalized groups are unknown. This may have led to the panel's considerations failing to see specific questions and needs of marginalized groups. As a result, recommendations in this guideline may be less appropriate regarding these groups. During future guideline developments, these limitations in involvement of patient participation in general and marginalized groups in particular should be taken into account.

Another limitation worth noting concerns the relatively informal approach employed to achieve consensus within the expert panel. This guideline was formulated in accordance with the internal guideline of the Dutch Association of Medical Specialists (Federatie Medische Specialisten). Throughout the different stages of development of this guideline, all members of the expert panel participated in meetings where rationales and preliminary recommendations were formulated. Subsequent to these meetings, panelists were invited to revisit the rationales and recommendations, with opportunities provided for them to propose modifications. However, it is acknowledged that contemporary standards emphasize the use of more formal methodologies for achieving consensus among groups of experts, citing greater reliability [18]. Informal consensus‐building methods carry the inherent risk of peer pressure influencing individual opinions and may lead to discussions being dominated by a minority of experts [18]. Although the process of formulating rationales and recommendations may lack complete transparency, we assert that the consensus‐building process was equitable and that the integrity of the final recommendations remained intact. Furthermore, the draft guideline was reviewed by all involved parties and finally approved.

## CONCLUSIONS

This guideline recommends starting with exercise therapy for PFP and PT. Additional conservative treatments are only indicated in the absence of clinically relevant changes and pain medications should be reserved for patients with severe pain. The additional value of imaging assessments for PT is limited. Open surgery may play a role in very specific cases. The recommendations in this guideline facilitate clinical decision‐making for healthcare providers in both primary and secondary care and thereby optimizing treatment and preventing unnecessary burdens, risks and costs to patients and society.

## AUTHOR CONTRIBUTIONS

Sietske van Berkel initiated and coordinated the project. All members of the expert panel supported the project by searching literature and screening trials for inclusion, interpretation of the results of the data analyses and adherence of the process to the standards of the Dutch Association of Medical Specialists. All members of the expert panel were responsible for screening the trials, risk of bias assessment, data extraction, interpretation of results of the data analyses and formulation of final recommendations. Every member of the expert panel was responsible for one specific module of the guideline and drafted the first version of the module. Martin Ophey, Sander Koëter, Lianne van Ooijen, Robbart van Linschoten and Sietske van Berkel drafted the translated version of the Dutch guideline into English. Mathijs van Ark, Fred Boots, Shanna Ilbrink, Nienke A. Lankhorst, Tom Piscaer, Myrthe Vestering and Mirre den Ouden Vierwind provided comments, and the final version was reviewed and approved by all members of the expert panel. All members agreed to be accountable for all their work. The corresponding author attests that all listed authors meet the authorship criteria and that no others meeting the criteria have been omitted.

## CONFLICT OF INTEREST STATEMENT

The personal financial interests, personal relationships, external research funding, intellectual property, and other potential conflicts of all the authors are described in the online Supporting Information.

## ETHICS STATEMENT

Not applicable.

## Supporting information

Supporting Information

## Data Availability

Data are available on reasonable request.

## Footnotes

* The Dutch healthcare system provides basic healthcare insurance including excess (‘eigen risico’). Therefore, patients are responsible for the costs for additional imaging assessments. These additional healthcare costs are maximized to €385.00 per year (2024).
