# Supplementary material for: Dutch multidisciplinary guideline on anterior knee pain: Patellofemoral pain and patellar tendinopathy
Source: Knee Surg Sports Traumatol Arthrosc. 2024 Jul 24;33(2):457–69. doi: 10.1002/ksa.12367 (PMC11792096; doi:10.1002/ksa.12367)
Supplement: Supplementary file 1 — Supporting Information [file KSA-33-457-s001.docx]

ONLINE SUPPLEMENTARY FILE

Dutch multidisciplinary guideline on anterior knee pain: patellofemoral pain and patellar tendinopathy

Table of content

[INTRODUCTION](#_Toc124345425) 3

[CONSTRUCTIONS OF MODULES 1](#_Toc124345429)3

- [Module 1 ‘Exercise therapy for PFP’ 1](#_Toc124345430)4
- [Module 2 ‘Additional conservative treatments for PFP’](#_Toc124345437) 67
- [Module 3 ‘Pain medications for PFP’ 12](#_Toc124345451)2
- [Module 4 ‘Diagnosing PT’ 1](#_Toc124345463)64
- [Module 5 ‘Exercise therapy for PT’ 2](#_Toc124345477)02
- [Module 6 ‘Additional conservative treatments for PT’ 2](#_Toc124345485)53
- [Module 7 ‘Pain medications for PT’](#_Toc124345500) 293
- [Module 8 ‘Open surgery for PFP and PT’ 3](#_Toc124345511)34

# INTRODUCTION

**Composition of the Dutch multidisciplinary expert panel ‘Anterior Knee Pain’**

M.J. Ophey, MSc, (Sports-) Physical Therapist, Royal Dutch Society for Physio- therapy (KNGF) and Dutch Society for Sports Physical Therapy (NVFS)

S. Koëter, MD, PhD, Orthopaedic Surgeon, Dutch Orthopaedic Association (NOV)

L.M. van Ooijen, BSc, (Sports-) Podiatrist and Manual Therapist, Dutch Association for Podotherapists (NVvP)

M. van Ark, PhD, Physical Therapist, Royal Dutch Society for Physiotherapy (KNGF) and Dutch Society for Sports Physical Therapy (NVFS)

G.P.G. Boots, MD, Occupational Physician, Netherlands Society of Occupational Medicine (NVAB)

S. Ilbrink, MD, Sports Medicine Physician, Netherlands Association of Sports Medicine (VSG)

N. E. Aerts - Lankhorst, MD, PhD, General Practitioner, Dutch College of General Practitioners (NHG)

T.M. Piscaer, MD, PhD, Orthopaedic Surgeon, Dutch Orthopaedic Association (NOV)

M. Vestering, MD, Radiologist, Radiological Society of the Netherlands (NVvR)

R. van Linschoten, MD, PhD, Sports Medicine Physician, Netherlands Association of Sports Medicine (VSG)

S. van Berkel, MD, PhD, Sports Medicine Physician, Netherlands Association of Sports Medicine (VSG)

# Methodological support

1. Mirre den Ouden - Vierwind, PhD, advisor, Knowledge Institute of the Dutch Association of Medical Specialists
2. Saskia Persoon, PhD, advisor, Knowledge Institute of the Dutch Association of Medical Specialists
3. Miriam van der Maten, MSc, research librarian, Knowledge Institute of the Dutch Association of Medical Specialists
4. Florien Ham, MSc, junior adviser, Knowledge Institute of the Dutch Association of Medical Specialists

# Declaration of interests

The code “prevention of influence by conflicts of interest” has been followed. All expert panel members have stated in writing whether they have had direct or indirect financial interests in the last three years. During development or revision, changes in interests are communicated to the chairman. The declaration is reaffirmed during the comment phase.

An overview of the interests can be found in Table 1. The signed declarations can be requested from the secretariat of Knowledge Institute of the Dutch Association of Medical Specialists.

The Guideline was financed by the Quality Fund for Medical Specialists, which is a quality fund for medical specialists in the Netherlands.

The financier has had no influence on the content of the guideline modules.

| **Member** | **Function** | **Ancillary positions** | **Declared interests** | **Action taken** |
| --- | --- | --- | --- | --- |
| *Ophey* | Working part-time as a physiotherapist at private clinic for physical therapy "YsveldFysio" in Nijmegen.  Parttime PhD candidate “patellofemoral pain” at the AMC orthopedics department | Continuing education of physiotherapists for various organizations at home and abroad (10-20 days per year), whereby a single training day also relates to the theme of "patellofemoral pain". This work has been paid | Scientific research focused on mobility in the kinetic chain in patients with patellofemoral pain (risk factor) and on homeostasis disturbances in the extensor apparatus (not subsidized) | No action |
| *Koëter* | Orthopaedic Surgeon CWZ | Part-trainer Sports Medicine CWZ  Head of research support office CWZ | None | No action |
| *Van Ooijen* | Sportspodiatrist at Profysic Sportpodotherapie | None | None | No action |
| *Van Ark* | Lecturer - Hanze University of Applied Sciences Groningen physiotherapy training physiotherapist / researcher – peescentrum Expertise Center Primary Care Groningen | Guest lecturer tendon injuries (paid courses/lessons on tendon injuries)  Triathlon Association Volunteer (unpaid) | None | No action |
| *Boots* | Occupational physician, Independent '(Boots) Solide Werken'. Working for various companies and for Volandis, the knowledge institute for sustainable employability in the Dutch construction industry. | Sports inspections at the SMA Gorinchem (paid)  Medical affairs Committee Lifesaving Dordrecht (volunteer/unpaid training life saving and first aid, advice events).  Member of the prevention working group of NVAB (unpaid) | None | No action |
| *Ilbrink* | Sports Medicine Physician 1. Jessica Gal Sportartsen 2. Sport- en Beweegkliniek (ZZP-er) | None | None | No action |
| *Aerts – Lankhorst* | General practitioner | None | None | No action |
| *Piscaer* | Orthopedic surgeon-traumatology, ErasmusMC | None | None | No action |
| *Vestering* | Radiologist Gelderse Vallei Hospital, Ede | None | None | No action |
| *Van Linschoten* | Sport Medicine Physician, independent | Editor-in-chief Sports & Medicine | None | No action |
| *Van Berkel** | Sports Medicine Physician, Isala Zwolle | None | None | No action |

Table 1 Overview of the potential conflicts of interests all expert panel members

* denotes the chairman of the expert panel.

# Method guideline development

AGREE II

This guideline has been developed according to the internal guideline of the Dutch Association of Medical Specialists (FMS). This internal guideline is in concordance with the Appraisal of Guidelines for Research and Evaluation II (AGREE II) (Brouwers, 2010).

Analysis and scoping questions

During the preparatory phase, the expert panel inventoried bottlenecks in the care for patients with anterior knee pain. Problems were also put forward by the Royal Dutch Society for Physiotherapy, Dutch Association of Podiatrists, Loop Foundation, the Association for Sports and Medicine and the National Health Care Institute through a written invitational conference. Based on the results of the analysis, the expert panel prioritized scoping questions.

Thereafter, the expert panel made an inventory of which outcome measures are relevant for the patient, looking at both desired and undesired effects.

Outcome measures

After formulating the search question associated with the initial question, the expert panel inventoried which outcome measures are relevant to the patient, looking at both desired and undesired effects. A maximum of eight outcome measures were used. The expert panel rated these outcome measures according to their relative importance in decision-making regarding recommendations, as critical (critical to decision-making), important (but not critical), and unimportant. The expert panel also defined at least for the crucial outcome measures which differences they considered clinically (patient) relevant.

Method literature summary

A detailed description of the literature search and selection strategy and the assessment of the risk-of-bias of the individual studies can be found under 'Search and selection' in the module section. The assessment of the strength of the scientific evidence is explained below.

Assessment of the strength of the scientific evidence

The strength of the scientific evidence was determined according to the GRADE method. GRADE stands for Grading Recommendations Assessment, Development and Evaluation (see http://www.gradeworkinggroup.org/). The basic principles of the GRADE methodology are: naming and prioritizing the clinically (patient) relevant outcome measures, a systematic review per outcome measure, and an assessment of the weight of evidence per outcome measure based on the eight GRADE domains (downgrading domains: risk of bias, inconsistency, indirectness, imprecision, and publication bias; domains for upgrading: dose-effect relationship, large effect, and residual plausible confounding).

GRADE distinguishes four grades for the quality of scientific evidence: high, moderate, low and very low (Table 2). These degrees refer to the degree of certainty that exists about the literature conclusion, in particular the degree of certainty that the literature conclusion adequately supports the recommendation (Schünemann, 2013; Hultcrantz, 2017).

| **GRADE** | **Definition** |
| --- | --- |
| High | There is high confidence the true effect of treatment is close to the estimated effect of treatment; it is very unlikely that the literature conclusion will change clinically relevant when results of new large-scale research are added to the literature analysis. |
| Moderate | There is reasonable assurance the true effect of treatment is close to the estimated effect of treatment; it is possible that the conclusion changes clinically relevant when results of new large-scale studies are added to the literature analysis. |
| Low | There is low certainty the true effect of treatment is close to the estimated effect of treatment; there is a real chance that the conclusion will change clinically relevant when results of new large-scale research are added to the literature analysis. |
| Very low | There is very low certainty the true effect of treatment is close to the estimated effect of treatment; the literature conclusion is very uncertain. |

Table 2 GRADE Classification

When assessing (grading) the strength of the scientific evidence in guidelines according to the GRADE methodology, limits for clinical decision-making play an important role (Hultcrantz, 2017). These are the limits that, if exceeded, would lead to an adjustment of the recommendation. To set limits for clinical decision-making, all relevant outcome measures and considerations should be considered. The boundaries for clinical decision-making are therefore not directly comparable with the minimal clinically important difference (MCID). Particularly in situations where an intervention has no significant disadvantage and the costs are relatively low, the threshold for clinical decision-making regarding the effectiveness of the intervention may lie at a lower value (closer to zero effect) than the MCID (Hultcrantz, 2017).

Considerations (from evidence to recommendation)

In addition to (the quality of) the scientific evidence, other aspects are also important in the decision making to recommendation and are taken into account, such as additional arguments from, for example, biomechanics or physiology, values ​​and preferences of patients, costs (required resources), acceptability, feasibility and implementation. These aspects are systematically listed and assessed (weighted) under the heading 'Considerations' and may be (partly) based on expert opinion. A structured format based on the evidence-to-decision framework of the international GRADE Working Group was used (Alonso-Coello, 2016a; Alonso-Coello, 2016b). This evidence-to-decision framework is an integral part of the GRADE methodology.

Recommendation

The recommendations answer the initial question and are based on the available scientific evidence and the most important considerations, and a weighting of the favorable and unfavorable effects of the relevant interventions. The strength of the scientific evidence and the weight assigned to the considerations by the working group together determine the strength of the recommendation. In accordance with the GRADE method, a low GRADE value of conclusions in the systematic literature analysis does not preclude a strong recommendation a priori, and weak recommendations are also possible with a high GRADE value (Hultcrantz 2017, Schüneman 2013).

The strength of the recommendation is always determined by weighing all relevant arguments together. The expert panel has included with each recommendation how they arrived at the direction and strength of the recommendation.

The GRADE methodology distinguishes between strong and weak (or conditional) recommendations. The strength of a recommendation refers to the degree of certainty that the benefits of the intervention outweigh the harms (or vice versa) across the spectrum of patients targeted by the recommendation. The strength of a recommendation has clear implications for patients, practitioners and policy makers. A recommendation is not a dictate, even a strong recommendation based on high quality evidence (‘high GRADE’) will not always apply, under all possible circumstances and for each individual patient.

Organization of care

In the constraint analysis and in the development of the guideline module, explicit attention was paid to the organization of care: all aspects that are preconditions for providing care (such as coordination, communication, (financial) resources, manpower and infrastructure). Preconditions that are relevant for answering this specific initial question are mentioned in the considerations.

Commentary and authorization phase

The draft guideline was submitted to all medical associations, the NPF and other relevant stakeholders. They provided comments which were viewed and processed by the expert panel. The final version of the guideline was approved by all involved medical associations.

References

Agoritsas T, Merglen A, Heen AF, Kristiansen A, Neumann I, Brito JP, Brignardello-Petersen R, Alexander PE, Rind DM, Vandvik PO, Guyatt GH. UpToDate adherence to GRADE criteria for strong recommendations: an analytical survey. BMJ Open. 2017 Nov 16;7(11):e018593. doi: 10.1136/bmjopen-2017-018593. PubMed PMID: 29150475; PubMed Central PMCID: PMC5701989.

Alonso-Coello P, Schünemann HJ, Moberg J, Brignardello-Petersen R, Akl EA, Davoli M, Treweek S, Mustafa RA, Rada G, Rosenbaum S, Morelli A, Guyatt GH, Oxman AD; GRADE Working Group. GRADE Evidence to Decision (EtD) frameworks: a systematic and transparent approach to making well informed healthcare choices. 1: Introduction. BMJ. 2016 Jun 28;353:i2016. doi: 10.1136/bmj.i2016. PubMed PMID: 27353417.

Alonso-Coello P, Oxman AD, Moberg J, Brignardello-Petersen R, Akl EA, Davoli M, Treweek S, Mustafa RA, Vandvik PO, Meerpohl J, Guyatt GH, Schünemann HJ; GRADE Working Group. GRADE Evidence to Decision (EtD) frameworks: a systematic and transparent approach to making well informed healthcare choices. 2: Clinical practice guidelines. BMJ. 2016 Jun 30;353:i2089. doi: 10.1136/bmj.i2089. PubMed PMID: 27365494.

Brouwers MC, Kho ME, Browman GP, Burgers JS, Cluzeau F, Feder G, Fervers B, Graham ID, Grimshaw J, Hanna SE, Littlejohns P, Makarski J, Zitzelsberger L; AGREE Next Steps Consortium. AGREE II: advancing guideline development, reporting and evaluation in health care. CMAJ. 2010 Dec 14;182(18):E839-42. doi: 10.1503/cmaj.090449. Epub 2010 Jul 5. Review. PubMed PMID: 20603348; PubMed Central PMCID: PMC3001530.

Hultcrantz M, Rind D, Akl EA, Treweek S, Mustafa RA, Iorio A, Alper BS, Meerpohl JJ, Murad MH, Ansari MT, Katikireddi SV, Östlund P, Tranæus S, Christensen R, Gartlehner G, Brozek J, Izcovich A, Schünemann H, Guyatt G. The GRADE Working Group clarifies the construct of certainty of evidence. J Clin Epidemiol. 2017 Jul;87:4-13. doi: 10.1016/j.jclinepi.2017.05.006. Epub 2017 May 18. PubMed PMID: 28529184; PubMed Central PMCID: PMC6542664.

Medisch Specialistische Richtlijnen 2.0 (2012). Adviescommissie Richtlijnen van de Raad Kwalitieit. <http://richtlijnendatabase.nl/over_deze_site/over_richtlijnontwikkeling.html>

Neumann I, Santesso N, Akl EA, Rind DM, Vandvik PO, Alonso-Coello P, Agoritsas T, Mustafa RA, Alexander PE, Schünemann H, Guyatt GH. A guide for health professionals to interpret and use recommendations in guidelines developed with the GRADE approach. J Clin Epidemiol. 2016 Apr;72:45-55. doi: 10.1016/j.jclinepi.2015.11.017. Epub 2016 Jan 6. Review. PubMed PMID: 26772609.

Schünemann H, Brożek J, Guyatt G, et al. GRADE handbook for grading quality of evidence and strength of recommendations. Updated October 2013. The GRADE Working Group, 2013. Available from http://gdt.guidelinedevelopment.org/central_prod/_design/client/handbook/handbook.html.

# CONSTRUCTIONS OF MODULES

# Construction of Module 1 ‘Exercise therapy for PFP’

Scoping Question: What is the value of exercise therapy for patients with PFP?

Search and select

A systematic review of the literature was performed to answer the following question:

What is the effect of exercise therapy in patients with PFP on pain, function, return to sports/ work, duration of absenteeism, patient satisfaction, and patient recovery?

P: patients with PFP (adolescents/adults, non-traumatic)

I: exercise therapy

C: control/ placebo/ wait and see policy

O: pain, function, return to sports/ work, duration of absenteeism, patient satisfaction, and patient recovery

Relevant outcome measures

The guideline development expert panel considered pain and function as a critical outcome measures for decision making; and return to sport/work, duration of absenteeism, and patient satisfaction as important outcome measures for decision making. For the outcome pain the Visual Analogue Scale (VAS) and the Numeric Rating Scale (NRS) were used. For the outcome function the Kujala score/ Anterior Knee Pain Score (AKPS) are often used in studies, however, other measurement instruments could also be used. Return to sport/work was measured with the Tegner score. Satisfaction with the result of treatment and recovery were usually measured on a Likert scale.

The expert panel defined a difference of 2 cm (of 10 cm) on the VAS or 2 categories on the NRS scale as a minimal clinically (patient) important difference, according to Crossley, 2004. For the Kujala score/ AKPS score, a difference of 10 point (of 100 points) was defined as a minimal clinically (patient) important difference, according to Crossley, 2004. A minimal clinically important difference for return to sport/work measured with the Tegner score was not predefined. A minimal clinically important difference for duration of absenteeism, patient satisfaction and patient recovery was not predefined.

Search and select (Methods)

The databases Pubmed and Embase (via Embase.com) were searched with relevant search terms until 22 April 2020. The systematic literature search resulted in 890 hits. Studies were selected based on the following criteria: Systematic reviews (searched in at least two databases, and detailed search strategy, risk of bias assessment and results of the individual studies available) and randomized controlled trials (RCTs) that included at least 20 patients with PFP, compared exercise therapy with a control group, and included at least one of the defined outcome measures. Initially eight reviews were selected based on their title and abstract. After reading the full text, seven reviews were excluded (see the table for exclusion under Literature), and 1 Cochrane systematic review was included (van der Heijden, 2015). Of the 16 RCTs published after 2014 and selected based on title and abstract screening, 11 were excluded, 1 study was included in the Cochrane review, and 3 RCTs (described in 4 articles (Hott 2019, Hott 2020, Rathleff 2015, Saad 2018)) were added to the analysis of the Cochrane review.

Results search

One systematic review and four RCTs studies were included in the analysis of the literature. Important study characteristics and results are summarized in the evidence tables. The assessment of the risk of bias is summarized in the risk of bias tables.

Key Items: 28778218 OR 25603546 OR 26311988 OR 26039034 OR 28559754 OR 28476901

| Search | Query | Items found |
| --- | --- | --- |
| #13 | Search #8 AND #4 | 607 |
| #12 | Search #8 AND #2 | 220 |
| #11 | Search #8 AND #1 | 114 |
| #10 | Search #9 AND #5 | 6 |
| #9 | Search #8 AND #3 | 288 |
| #8 | Search #6 AND #7 | 1067 |
| #7 | Search ("Exercise Therapy"[Mesh] OR "Exercise"[tiab] OR "exercises"[tiab] OR Physiotherap*[tiab] OR physical therap*[tiab] OR "resistance training"[tiab] OR (("strengthen"[tiab] OR "strengthening"[tiab] OR "training"[tiab] OR "eccentric"[tiab] OR "isometric"[tiab] OR "concentric"[tiab] OR "isotonic"[tiab]) AND ("hip"[tiab] OR "hips"[tiab] OR "knee"[tiab] OR "knees"[tiab] OR "gluteal"[tiab] OR "gluteus"[tiab] OR "quadriceps"[tiab] OR "vastus medialis"[tiab])) OR "Mensendieck"[tiab] OR "Cesar therapy"[tiab] OR "pilates"[tiab] OR "squat"[tiab] OR "squats"[tiab] OR "squatting"[tiab]) | 354697 |
| #6 | Search ("Patellofemoral Pain Syndrome"[Mesh] OR "Patellofemoral Pain"[tiab] OR "Anterior Knee Pain" OR "Patellofemoral Syndrome"[tiab] OR patellofemoral dysfunction*[tiab] OR patellofemoral disorder*[tiab] OR "PFP"[ti] OR "PFPS"[ti] OR "retro-patellar pain"[tiab] OR "retropatellar pain"[tiab] OR "peri-patellar pain"[tiab] OR "anterior knee pain"[tiab] OR Patella chondropath*[tiab] OR Patellar chondropath*[tiab] OR Patella chrondromalacia*[tiab] OR Patellar chrondromalacia*[tiab] OR "runner's knee"[tiab] OR "lateral facet compression syndrome"[tiab] OR chondromalacia patella*[tiab]) | 3785 |
| #5 | Search 28778218 25603546 26311988 26039034 28559754 28476901[uid] | 6 |
| #4 | Search ("cohort studies"[mesh] OR "case-control studies"[mesh] OR "comparative study"[pt] OR "risk factors"[mesh] OR "cohort"[tw] OR "compared"[tw] OR "groups"[tw] OR "case control"[tw] OR "multivariate"[tw]) | 7850089 |
| #3 | Search #1 OR #2 | 1001329 |
| #2 | Search ((random*[tiab] AND (controlled[tiab] OR control[tiab] OR placebo[tiab] OR versus[tiab] OR vs[tiab] OR group[tiab] OR groups[tiab] OR comparison[tiab] OR compared[tiab] OR arm[tiab] OR arms[tiab] OR crossover[tiab] OR cross-over[tiab]) AND (trial[tiab] OR study[tiab])) OR ((single[tiab] OR double[tiab] OR triple[tiab]) AND (masked[tiab] OR blind*[tiab]))) | 725560 |
| #1 | Search ("Meta-Analysis as Topic"[Mesh] OR “Meta-Analysis”[Publication Type] OR metaanaly*[tiab] OR metanaly*[tiab] OR meta-analy*[tiab] OR meta synthes*[tiab] OR metasynthes*[tiab] OR meta ethnograph*[tiab] OR metaethnograph*[tiab] OR meta summar*[tiab] OR metasummar*[tiab] OR meta-aggregation[tiab] OR metareview[tiab] OR meta-review[tiab] OR overview of reviews[tiab] OR ((systematic*[ti] OR scoping[ti] OR umbrella[ti] OR meta-narrative[ti] OR metanarrative[ti] OR evidence based[ti]) AND (review*[ti] OR overview*[ti])) OR ((evidence[ti] OR narrative[ti] OR metanarrative[ti] OR qualitative[ti]) AND synthesis[ti]) OR systematic review[pt] OR prisma[tiab] OR preferred reporting items[tiab] OR quadas*[tiab] OR systematic review*[tiab] OR systematic literature[tiab] OR structured literature search[tiab] OR systematic overview*[tiab] OR scoping review*[tiab] OR umbrella review*[tiab] OR mapping review*[tiab] OR systematic mapping[tiab] OR evidence synthes*[tiab] OR narrative synthesis[tiab] OR metanarrative synthesis[tiab] OR research synthesis[tiab] OR qualitative synthesis[tiab] OR realist synthesis[tiab] OR realist review[tiab] OR realist evaluation[tiab] OR systematic qualitative review[tiab] OR mixed studies review[tiab] OR mixed methods synthesis[tiab] OR mixed research synthesis[tiab] OR quantitative literature review[tiab] OR systematic evidence review[tiab] OR evidence-based review[tiab] OR comprehensive literature search[tiab] OR integrated review*[tiab] OR integrated literature review[tiab] OR integrative review*[tiab] OR integrative literature review*[tiab] OR structured literature review*[tiab] OR systematic search and review[tiab] OR meta-narrative review*[tiab] OR metanarrative review[tiab] OR systematic narrative review[tiab] OR systemic review[tiab] OR systematized review[tiab] OR systematic research synthesis[tiab] OR bibliographic*[tiab] OR hand-search*[tiab] OR handsearch*[tiab] OR manual search*[tiab] OR searched manually[tiab] OR manually searched[tiab] OR journal database*[tiab] OR review authors independently[tiab] OR reviewers independently[tiab] OR independent reviewers[tiab] OR independent review authors[tiab] OR electronic database search*[tiab] OR (study selection[tiab] AND data extraction[tiab]) OR (selection criteria[tiab] AND data collection[tiab]) OR (selection criteria[tiab] AND data analysis[tiab]) OR (evidence acquisition[tiab] AND evidence synthesis[tiab]) OR (pubmed[tiab] AND embase[tiab]) OR (medline[tiab] AND embase[tiab]) OR (pubmed[tiab] AND cochrane[tiab]) OR (medline[tiab] AND cochrane[tiab]) OR (embase[tiab] AND cochrane[tiab]) OR (pubmed[tiab] AND psycinfo[tiab]) OR (medline[tiab] AND psycinfo[tiab]) OR (embase[tiab] AND psycinfo[tiab]) OR (cochrane[tiab] AND psycinfo[tiab]) OR (pubmed[tiab] AND web of science[tiab]) OR (medline[tiab] AND web of science[tiab]) OR (embase[tiab] AND web of science[tiab]) OR (psycinfo[tiab] AND web of science[tiab]) OR (cochrane[tiab] AND web of science[tiab]) OR ((literature[ti] OR qualitative[ti] OR quantitative[ti] OR integrated[ti] OR integrative[tiab] OR rapid[ti] OR short[ti] OR critical*[ti] OR mixed stud*[ti] OR mixed method*[ti] OR focused[ti] OR focussed[ti] OR structured[ti] OR comparative[ti] OR comparitive[ti] OR evidence[ti] OR comprehensive[ti] OR realist[ti]) AND (review*[ti] OR overview*[ti]) AND (literature search[tiab] OR structured search[tiab] OR electronic search[tiab] OR search strategy[tiab] OR gray literature[tiab] OR grey literature[tiab] OR Review criteria[tiab] OR eligibility criteria[tiab] OR inclusion criteria[tiab] OR exclusion criteria[tiab] OR predetermined criteria[tiab] OR included studies[tiab] OR identified studies[tiab] OR (systematic search[tiab] AND literature[tiab]) OR strength of evidence[tiab] OR citation*[tiab] OR references[tiab] OR database search*[tiab] OR electronic database*[tiab] OR data base search*[tiab] OR electronic data-base*[tiab] OR search criteria[tiab] OR study selection[tiab] OR data extraction[tiab] OR methodological quality[tiab] OR methodological characteristics[tiab] OR methodologic quality[tiab] OR methodologic characteristics[tiab])) OR ((literature review[tiab] OR literature search*[tiab]) AND (structured search[tiab] OR electronic search[tiab] OR Search strategy[tiab] OR gray literature[tiab] OR grey literature[tiab] OR review criteria[tiab] OR eligibility criteria[tiab] OR inclusion criteria[tiab] OR exclusion criteria[tiab] OR predetermined criteria[tiab] OR included studies[tiab] OR identified studies[tiab] OR (systematic search[tiab] AND literature[tiab]) OR strength of evidence[tiab] OR citation*[tiab] OR references[tiab] OR database search*[tiab] OR electronic database*[tiab] OR data base search*[tiab] OR electronic data-base*[tiab] OR search criteria[tiab] OR study selection[tiab] OR data extraction[tiab] OR methodological quality[tiab] OR methodological characteristics[tiab] OR methodologic quality[tiab] OR methodologic characteristics[tiab]))) NOT ("Comment" [Publication Type] OR "Letter" [Publication Type]) NOT (“Animals”[Mesh] NOT “Humans”[Mesh]) | 341259 |

Table 1.1 Pubmed search

| Query | Results |
| --- | --- |
| #7 AND #3 | **367** |
| #7 AND #2 | **408** |
| #7 AND #1 | **108** |
| #6 NOT ('conference abstract'/it OR 'editorial'/it OR 'letter'/it OR 'note'/it) NOT (('animal experiment'/exp OR 'animal model'/exp OR 'nonhuman'/exp) NOT 'human'/exp) | **1308** |
| #4 AND #5 | **1577** |
| 'kinesiotherapy'/exp OR 'physiotherapy'/exp OR 'exercise':ti,ab,kw OR 'exercises':ti,ab,kw OR 'physiotherap*':ti,ab,kw OR 'physical therap*':ti,ab,kw OR 'resistance training'/de OR 'resistance training':ti,ab,kw OR (('strengthen':ti,ab,kw OR 'strengthening':ti,ab,kw OR 'training':ti,ab,kw OR 'eccentric':ti,ab,kw OR 'isometric':ti,ab,kw OR 'concentric':ti,ab,kw OR 'isotonic':ti,ab,kw) AND ('hip':ti,ab,kw OR 'hips':ti,ab,kw OR 'knee':ti,ab,kw OR 'knees':ti,ab,kw OR 'gluteal':ti,ab,kw OR 'gluteus':ti,ab,kw OR 'quadriceps':ti,ab,kw OR 'vastus medialis':ti,ab,kw)) OR 'mensendieck':ti,ab,kw OR 'cesar therapy':ti,ab,kw OR 'pilates':ti,ab,kw OR 'squat':ti,ab,kw OR 'squats':ti,ab,kw OR 'squatting':ti,ab,kw | **547746** |
| 'patellofemoral pain syndrome'/exp OR 'patellofemoral pain':ti,ab,kw OR 'patellofemoral syndrome':ti,ab,kw OR 'patellofemoral dysfunction*':ti,ab,kw OR 'patellofemoral disorder*':ti,ab,kw OR 'pfp':ti OR 'pfps':ti OR 'retro-patellar pain':ti,ab,kw OR 'retropatellar pain':ti,ab,kw OR 'peri-patellar pain':ti,ab,kw OR 'anterior knee pain':ti,ab,kw OR 'patella chondropath*':ti,ab,kw OR 'patellar chondropath*':ti,ab,kw OR 'patella chrondromalacia*':ti,ab,kw OR 'patellar chrondromalacia*':ti,ab,kw OR 'runner s knee':ti,ab,kw OR 'lateral facet compression syndrome':ti,ab,kw OR 'chondromalacia patella*':ti,ab,kw | **4834** |
| 'major clinical study'/de OR 'clinical study'/de OR 'case control study'/de OR 'family study'/de OR 'longitudinal study'/de OR 'retrospective study'/de OR 'prospective study'/de OR 'cohort analysis'/de OR ((cohort NEAR/1 (study OR studies)):ab,ti) OR (('case control' NEAR/1 (study OR studies)):ab,ti) OR (('follow up' NEAR/1 (study OR studies)):ab,ti) OR (observational NEAR/1 (study OR studies)) OR ((epidemiologic NEAR/1 (study OR studies)):ab,ti) OR (('cross sectional' NEAR/1 (study OR studies)):ab,ti) | **5215053** |
| 'clinical trial'/exp OR 'randomization'/exp OR 'single blind procedure'/exp OR 'double blind procedure'/exp OR 'crossover procedure'/exp OR 'placebo'/exp OR 'prospective study'/exp OR rct:ab,ti OR random*:ab,ti OR 'single blind':ab,ti OR 'randomised controlled trial':ab,ti OR 'randomized controlled trial'/exp OR placebo*:ab,ti | **3024338** |
| 'meta analysis'/de OR cochrane:ab OR embase:ab OR psycinfo:ab OR cinahl:ab OR medline:ab OR ((systematic NEAR/1 (review OR overview)):ab,ti) OR ((meta NEAR/1 analy*):ab,ti) OR metaanalys*:ab,ti OR 'data extraction':ab OR cochrane:jt OR 'systematic review'/de | **492675** |

Table 1.2 Embase search

|  | **Embase.com** | **PubMed (legacy)­** | **Deduplicated** |
| --- | --- | --- | --- |
| SR/meta analysis | 108 | 114 | 136 |
| RCTs | 408 | 220 | 349 |
| Observational studies | 367 | 607 | 408 |
| Other |  |  |  |
| Total | 883 | 941 | 894 |

Table 1.3 Search results

| **Author and year** | **Reason for exclusion** |
| --- | --- |
| Alba-Martin 2015 | Review without meta-analysis from the 10 included RCTS, pure descriptive |
| Ashraf 2017 | Language (RCT) |
| Azahin 2016 | RCT: control group received knee exercises |
| Bolgla 2005 | Review without meta-analysis |
| Bolgla 2016 | RCT: control group received knee exercises |
| Clijsen 2014 | Review with meta-analysis, overlaps largely with van der Heijden 2015 |
| Drew 2017 | feasibility study |
| Ferber 2015 | RCT: control group received knee exercises |
| Foroughi 2019 | RCT: both groups receive strength and stretching exercises |
| Frye 2012 | Review including RCT’s, cohort and observational studies |
| Heijntjes 2003 | Old Cochrane review, update by van der Heijden 2015 |
| Karakuay 2014 | Language |
| Kooijker 2014 | Included studies overlap with van der Heijden 2015 |
| Lun, 2005 | Control group receives brace |
| Rabelo 2017 | Control group receives strength exercises for hip and knee |
| Rogan 2019 | Review included 3 RCT’s >2014 which are found in the search for this module |
| Saltychev 2008 | Review without meta-analyse |
| Sharif 2002 | Control group in RCT (included 10 minutes ultrasonic therapy at pulsed mode, quadriceps strengthening, and active short act extension exercises and proprioception training five days a week) |
| Shetty 2016 | Control group in RCT received conventional physical therapy and inactive population |
| Soleimani 2017 | Language |
| Taylor, 2003 | Pilot study <20 participants |

Table 1.4 Excluded studies

| **Study reference** | **Study characteristics** | **Patient characteristics** | **Intervention (I)** | **Comparison / control (C)** | **Follow-up** | **Outcome measures and effect size** | **Comments** |
| --- | --- | --- | --- | --- | --- | --- | --- |
| van der Heijden, 2015 | SR and meta-analysis of RCTs  *Literature search up to (month/year)*  **A:** Abrahams, 2003  **B:** Clark, 2000  **C:** Fukuda, 2010  **D:** Herrington, 2007  **E:** Loudon, 2004  **F:** Moyano, 2013  **G:** Song, 2009  **H:** Van Linschoten, 2009  Study design: RCTs and quasi-randomised trials  Setting and Country:  **A**: n.r., UK  **B**: Australia  **C:** n.r., Brazil  **D:** Physical Therapy Department at Riyadh Armed Forces Hospital; Saudi Arabia  **E:** n.r., USA  **F:** n.r., Spain  **G:** n.r., Taiwan  **H:** n.r., the Netherlands  Source of funding and conflicts of interest:  Not reported | Inclusion criteria SR:  Exclusion criteria SR:  *31 studies included in total, 10 studies compared exercise therapy with control*  Important patient characteristics at baseline:  N, mean age  **A**: 78 patients, 29 yrs, 50% female  **B**: 81 patients, 28 yrs, 44% female  **C**: 70 patients, 25 yrs, all female  **D:** 45 patients, 27 yrs, all male  **E:** 32 patients, 27 yrs, 76% female  **F:** 94 patients, 40 yrs, 43% female  **G:** 89 patients, 41 yrs, 87% female  **H:** 131 patients, 24 yrs, 64% female  Groups comparable at baseline? | Describe intervention:  **A**: 1) Traditional exercise protocol / 2) Same exercise protocol with thigh adduction and tibia medial rotation during eccentric squat  **B**: 1) Exercise + tape: 6 sessions and daily training at home / 2) Exercise: 6 sessions and daily training at home  **C**: 1) Knee exercises including iliopsoas strengthening in non-weight bearing, seated knee extension 90°-45°, leg press 0°-45°, squatting 0°-45° / 2) Knee and hip exercises including iliopsoas strengthening in non-weight bearing, seated knee extension 90°-45°, leg press 0°-45°, squatting 0°-45°, hip abduction against elastic band (standing), hip abduction with weights (side lying), hip external rotation against elastic band (sitting), side-stepping against elastic band, 3 x 1 minute lateral rotator muscles  **D:** 1) Single Joint Non-Weight Bearing (= OKC) including knee extension exercises in a seated position from 90° of knee flexion to full extension / 2) Multi Joint Weight Bearing (= CKC) including leg press exercise in a seated position from 90° of knee flexion to full extension  **E:** 1) Home exercises + 5 physical therapy visits / 2) Supervised exercises twice a week for 4 weeks, plus 1 physical therapy visit at 6 weeks and 1 at 8 weeks; and additional home exercises  **F:** 1) 'Classic stretching protocol' (stretching exercises for hip and knee muscles) and quadriceps strengthening exercises: 3 times per week  2) Proprioceptive neuromuscular facilitation stretching applied to hamstrings and quadriceps and, after the 4th week, aerobic exercise: 3 times per week  **G:** 1) Hip adduction combined with leg-press exercise (knee + hip): 3 times a week / 2) Leg-press exercise only (knee): 3 times a week  **H:** Exercise therapy including static and dynamic exercises for quadriceps, adductor and gluteal muscles: 9 times in 6 weeks + daily at home | Describe control:  **A**: waiting list  **B**: 1) Tape: 6 sessions and daily at home / 2) no treatment. Additional intervention in all groups: education  **C**: no treatment  **D:** no treatment  **E:** no treatment  **F:** Health educational materials  **G:** Health educational material  **H:** Usual care: "wait and see" approach | Endpoint of follow-up:  **A**: 6 weeks  **B**: 12 months  **C**: 4 weeks  **D:** 6 weeks  **E:** 8 weeks  **F:** 16 weeks  **G:** 8 weeks  **H**: 12 months  For how many participants were no complete outcome data available?  (intervention/control)  **A**: no dropouts  **B**: 12% dropout in the short-term; 39% dropout at 12 months follow-up  **C**: 11% dropout in the short term  **D**: no dropout  **E**: 10% dropout  **F**: 2.7% dropout in the short-term  **G**: 11% dropout in the short-term  **H**: 11% dropout in the short-term | Outcome measure pain (short-term)  Effect measure: mean difference (95% CI):  **B:** 0.59 (-1.90, 0.72)  -1.10 (-2.23, 0.04)  **C:** -1.80 (-3.30, -0.30)  -0.60 (-2.30, 1.10)  **D:** -4.00 (-5.47, -2.53)  -3.29 (-4.89, -1.69)  **H:** -0.79 (-1.80, 0.22)  Pooled effect (random effects model):  -1.46 (95% CI -2.39 to -0.54) favoring intervention group  Heterogeneity (I^2^): 74%  Outcome measure pain (long-term)  Effect measure: mean difference (95% CI):  **B:** -2.11 (-4.37, 0.15)  -0.69 (-2.52, 1.14)  **H:** -0.97 (-2.05, 0.11)  Pooled effect (fixed effects model):  -1.07 (95% CI -1.93 to -0.21) favoring intervention group  Heterogeneity (I^2^): 0%  Outcome measure function (short-term)  Effect measure: standardized mean difference (95% CI):  **B:** 0.69 (-0.01, 1.38)  0.69 (-0.39, 0.91)  **C:** 0.96 (0.19, 1.74)  1.21 (0.43, 2)  **D:** 5.93 (3.86, 8)  3.43 (1.99, 4.86)  **E:** 1.29 (0.06, 2.52)  1.27 (0.11, 2.43)  **F:** 1.05 (0.35, 1.76)  1 (0.28, 1.72)  **H:** 0.23 (-0.11, 0.58)  Pooled effect (random effects model):  1.1(95% CI 0.58 to 1.63) favoring intervention group  Heterogeneity (I^2^): 84%  Outcome measure function (long-term)  Effect measure: standardized mean difference (95% CI):  **B:** 0.59 (-0.27, 1.46)  0.32 (-0.44, 1.09)  **F:** 6.16 (4.7, 7.63)  1.6 (0.88, 2.32)  **H:** 0.21 (-0.14, 0.55)  Pooled effect (random effects model):  1.62 (95% CI 0.31 to 2.94) favoring intervention group  Heterogeneity (I^2^): 97% | Author’s conclusion  This review has found very low quality but consistent evidence that exercise therapy for patellofemoral pain syndrome (PFPS) may result in clinically important reduction in pain and improvement in functional ability, as well as enhancing long-term recovery. However, the best form of exercise therapy and whether this result would apply to all people with PFPS are unknown. |

Table 1.5 Evidence table for systematic review of RCTs and observational studies (intervention studies)

| **Study reference** | **Study characteristics** | **Patient characteristics ^2^** | **Intervention (I)** | **Comparison / control (C) ^3^** | **Follow-up** | **Outcome measures and effect size ^4^** | **Comments** |
| --- | --- | --- | --- | --- | --- | --- | --- |
| Hott, 2019/  Hott, 2020 | Type of study:  single-blind RCT  Setting and country:  outpatient clinic at the Department of Physical Medicine and Rehabilitation at  Sørlandet Hospital,  Norway  Funding and conflicts of interest:  Funded by The Research  Department of Sørlandet Hospital.  No conflicts of interest | Inclusion criteria:  Aged between 16 years 40 years, a minimum 3-month history of PFP (pain, ≥3 of 10) reproduced by at least 2 activities  (stair ascent/descent, hopping, running, prolonged sitting, squatting, kneeling) and present on at least 1 clinical test (compression of the patella, palpation of the patellar facets). For patients with bilateral pain, the worst knee was included.  Exclusion criteria:  (1) clinical, radiographic, or MRI findings indicative of other specific pathology, including meniscal, ligament, or cartilage injury, as well as osteoarthritis, epiphysitis, significant knee joint effusion, or recurrent patellar subluxation or dislocation; (2) significant pain from hip or back hindering the ability to perform the prescribed exercises; (3) previous surgery to the knee joint; (4) nonsteroidal anti-inflammatory drug or cortisone use over an extended period; (5) previous trauma to the knee joint with an effect on the presenting clinical condition; and (6) physiotherapy or other similar exercises for patellofemoral pain within the previous 3 months.  N total at baseline:  Knee: 37  Hip: 39  Control: 36  Important prognostic factors^2^:  *age ± SD:*  *Knee:* *28.5±6.2*  *Hip:* *27.8±8.6*  *C:* *26.3±7.0*  *Sex:*  *Kneel: 35% M*  *Hip: 36% M*  *C: 33% M*  Groups comparable at baseline?  Yes | The hip-based and knee-based exercise regimens were matched in dosage and progression. Three sessions per week were performed for 6 weeks: 1 under supervision of the physiotherapist and 2 home sessions, with at least 1 day between sessions. Initial dosage was 3 sets of 10 repetitions for each exercise, with progression to a maximum 3 3 20 repetitions. Each repetition was performed dynamically over 2 to 3 seconds, with a 2-second pause between repetitions and a 30-second pause between sets. Additional resistance thereafter was achieved through weights or elastic tubing depending on the exercise.  The knee-focused exercise regimens intended to maximally isolate the quadriceps muscles. The exercises consisted of straight-leg raises in the supine position, supine terminal knee extensions (from 10^○^ of flexion to full extension), and a mini-squat (45^○^ of flexion) with the back supported against the wall (to reduce stabilizing requirements from the hip muscles).  The hip-focused exercises consisted of side-lying hip abduction, hip external rotation (clam shell), and prone hip extension. These exercises were intended to maximally isolate the hip abductors, extensors, and external rotators without stimulating the quadriceps muscles. | The control group was encouraged by the study physiotherapist to be physically active in accordance with standardized information. | Length of follow-up:  3 months (Hott, 2019)  12 months (Hott, 2020)  Loss-to-follow-up 3 months:  Knee: 6 (16%)  Reasons: discontinued intervention, unavailable, illness  Hip: 3 (8%)  Reasons: unavailable, illness  Control: 3 (8%)  Reasons: dissatisfied with group allocation, time constraints  Loss-to-follow-up 6 months:  Knee: 8 (22%)  Reasons: discontinued intervention, unavailable  Hip: 6 (15%)  Reasons: unavailable, illness  Control: 8 (22%)  Reasons: dissatisfied with group allocation, time constraints | Outcome measures and effect size (include 95%CI and p-value if available):  AKPS (function) at 3 months  Knee: 74.4 (69.8-79.0)  Hip: 73.1 (69.5-76.7)  Control: 73.1 (68.2-78.0)  AKPS (function) at 12 months  Knee: 76.6 (70.4 to 83.0)  Hip: 77.6 (73.4 to 81.8)  Control: 78.7 (73.0 to 84.4)  Usual pain at 3 months  Knee: 2.6 (1.8-3.4)  Hip: 2.9 (2.4-3.5)  Control: 3.2 (2.5-3.9)  Usual pain at 12 months  Knee: 2.8 (1.7 to 3.9)  Hip: 2.2 (1.4 to 3.0)  Control: 2.6 (2.1 to 3.1)  Worst pain at 3 months  Knee: 4.0 (3.0-5.1)  Hip: 4.9 (4.1-5.7)  Control: 5.0 (4.1-5.9)  Worst pain at 12 months  Knee: 3.7 (2.5 to 4.9))  Hip: 4.4 (3.6 to 5.3)  Control: 3.5 (2.4 to 4.6) | At inclusion, all participants attended an individual 1-hour consultation with a specialist in physical medicine and rehabilitation. |
| Rathleff, 2015 | Type of study:  cluster RCT  Setting and country:  upper secondary school, Denmark  Funding and conflicts of interest:  funded by the Danish Rheumatism Association, The Association of Danish Physiotherapists Research Fund and The Obelske Family Foundation. MSR is being funded by a full-time PhD scholarship from the Graduate School of Health Sciences at Aarhus University  Competing interests: None. | Inclusion criteria:  insidious onset of anterior knee or retropatellar pain of more than 6 weeks duration and provoked by at least two of the following situations: prolonged sitting or kneeling, squatting, running, hopping or stair climbing; tenderness on palpation of the patella, pain when stepping down or double leg squatting; and worst pain during the previous week of more than 30 mm on a 100 mm visual analogue scale (VAS).  Exclusion criteria:  concomitant injury or pain from the hip, lumbar spine or other knee structures; previous knee surgery; self-reported patellofemoral instability; knee joint effusion; use of physiotherapy for treating knee pain within the previous year; or at least weekly use of anti-inflammatory drugs.  N total at baseline:  Intervention: 62  Control:59  Important prognostic factors^2^:  *Age ± SD:*  *I:17.2±1.1*  *C:17.3±0.9*  *Sex:*  *I: 26% M*  *C: 14% M*  Groups comparable at baseline?  Yes | Patient education and exercise therapy:  The exercise therapy consisted of a combination of supervised group training sessions and unsupervised home-based exercises.  The supervised group training sessions consisted of neuromuscular training of the muscles around the foot, knee and hip, strength training for the knee and hip, patellofemoral soft tissue mobilisation, and stretching of the muscles around the hip and knee. To progressively match the exercise level to the performance level of each participant, all exercises were available in multiple levels of difficulty. All adolescents started with exercises at level 1 and progressed from there. The supervised exercises were offered three times per week on school premises immediately after the end of the school day for 3 months.  The unsupervised home exercises consisted of approximately 15 min of quadriceps and hip muscle retraining and stretching. Instructions were given immediately after patient education together with a five-page leaflet with pictures and descriptions of the exercises. The exercises were to be performed each day except on the days of supervised group training. The adolescents were instructed to incorporate the exercises into their normal daily routines. Taping corrections were applied in a predetermined order of anterior tilt, medial tilt, glide and fat pad unloading until the participant’s pain was reduced by at least 50%.Tape was only used if adolescents achieved a minimum of 50% reduction in pain measured with a 10 cm VAS during a two-leg squat immediately after application of the tape. | Patient education:  One physiotherapist delivered the patient education in the two clusters randomised to patient education alone. The standardised patient education was held one-on-one with the adolescents and their parents. It lasted for about 30 min and covered: pain management; how to modify physical activity using pacing and load management strategies; information on optimal knee alignment during daily tasks; and responses to questions from the adolescent or the parents. Adolescents also received this information in an eight-page leaflet. | Length of follow-up:  3, 6 , 12 and 24 months  Loss-to-follow-up:  Intervention:  3 months: 13 (21%)  6 months: 18 (29%)  12 months: 10 (16%)  24 months: 14 (23%)  Reasons: n.r.  Control:  3 months: 7 (12%)  6 months: 19 (32%)  12 months: 1 (2%)  24 months: 8 (14%)  Reasons : n.r.  Incomplete outcome data:  Intervention:  N (%)  Reasons (describe)  Control:  N (%)  Reasons (describe) | Outcome measures and effect size (include 95%CI and p-value if available):  Recovery 3 months  I: 29%  C: 19%  OR (95% CI): 1.88 (1.25-2.81)  Recovery 6 months  I: 32%  C: 23%  OR (95% CI): 1.43 (0.22-9.24)  Recovery 12 months  I: 38%  C: 29%  OR (95% CI): 1.73 (1.02-2.93)  Recovery 24 months  I: 44%  C: 22%  OR (95% CI): 2.52 (1.65-3.86)  Worst pain 3 months  I: 51 (44;58)  C: 40 (24;56)  Adjusted mean difference (95% CI): −11 (−30 to 9)  Worst pain 6 months  I: 51 (31;70)  C: 41 (21;60)  Adjusted mean difference (95% CI): −10 (−38 to 19)  Worst pain 6 months  I: 49 (45;53)  C: 37 (34;39)  Adjusted mean difference (95% CI):-−11 (−18 to 5)  Worst pain 24 months  I: 35 (1;69)  C: 24 (15;33)  Adjusted mean difference (95% CI):−11 (−46 to 25) |  |
| Saad, 2018 | Type of study:  4arm, randomized controlled assessor-blinded trial  Setting and country: university campus Ribeirão Preto Medical School, Universidade de São Paulo (USP), Ribeirão Preto, SP, Brazil  Funding and conflicts of interest:  This study received financial support of The State of São Paulo Research Foundation --- FAPESP (process number: 2010-/12561-9).  The authors declare no conflicts of interest. | Inclusion criteria:  Female recreational athlete , defined as participating in aerobic or athletic activity at least 3 times per week for at least 30 min and had anterior knee pain with a minimum intensity of 3 or greater on the 10-cm VAS for at least three months before the study assessment.  insidious onset of symptoms; retropatellar or peripatellar pain with at least 2 of the following activities (ascending/descending stairs, running, kneeling, squatting, prolonged sitting or jumping).  Exclusion criteria:  previous history of knee surgery; history of back, hip, or ankle joint injury or pain; (3) patellar instability; (4) lesion or pain during palpation or test of any structure of knee and (6) any neurological involvement that would affect gait  N total at baseline:  Quadriceps group: 10  Hip group: 10  Stretching group: 10  Control group: 10  Important prognostic factors^2^:  *Age ± SD:*  *Quadriceps group: 23.2±2.53*  *Hip group: 22.5±1.08*  *Stretching group: 21.3±1.16*  *Control group: 23.2±1.03*  Groups comparable at baseline? | Quadriceps group  The exercises in this group focused specifically on quadriceps strengthening  1.Bike – 15 min. warmup  2.Straight Leg Raise – 3x 10 repetitions with ankle weights resistance*.  3.Seated knee extension (open kinetic chain exercise, 90º – 60º of knee flexion) – 3x 10 repetitions with resistance*.  4.Leg Press (closed kinetic chain exercise, 0º – 45º of knee flexion) – 3x 10 repetitions with resistance*.  5.Wall Slide Squat at 90 º - 3 sets of 1 minute  Hip group  This group performed exercises to strengthen hip stabilizing muscles  1.Bike – 15 minutes warmup  2.Straight Leg Raise inside lying– 3x 10 repetitions using ankle weights as resistance*.  3.Supine Bridge on Ball Lateral bridge 3x 10 repetitions with 10 second isometric hold on the last repetition  4.Seated Hip Abduction 3x 10 repetitions with resistance*.  5.Strengthening the extensors, abductors, and hip external rotators at four support positions 3x 10 repetitions with ankle weights resistance*.  *Resistance = the weights were increased based on the patient’s reports on the Rating of Perceived Exertion (RPE) scale based on Borg's Scale of Effort.  Stretching group  In this group, the physical therapist monitored and stabilized the patients during the stretching exercises for all muscles involved in knee and hip stabilization:  1.Quadriceps stretching (standing/lying position)  2.Hamstrings stretching (supine position/seated position)  3.Gastrocnemius and soleus stretching (with assistance of ramp/standing position)  4.External rotators of hip and iliotibial band stretching  5.Flexors of hip stretching (on the floor/with assistance of the therapist)  6.Aductors stretching  7.Abdominal stretching  All exercises: 3x 30s stretches | Patients included in this group did not have any kind of intervention for eight weeks, but they were tested at the start of the program & at the end like the other 3 groups. | Length of follow-up:  8 weeks  Loss-to-follow-up:  Quadriceps group: 1 (10%)  Hip group: 0  Stretching group: 0  Control group: 0  Reasons: n.r. | Outcome measures and effect size (include 95%CI and p-value if available):  Pain  Quadriceps group: 0.56±0.89  Hip group: 0.55±0.8  Stretching group: 0.14±0.25  Control group: 3.69±0.6  AKPS  Quadriceps group: 90.11±6.11  Hip group: 91.8±5.67  Stretching group: 91.0±6.62  Control group: 81.9±8.41 |  |

Table 1.6 Evidence table for intervention studies

n.r.= not reported, VAS: visual analog scale

1. of patients between treatment groups (case-control studies) or multivariate adjustment for prognostic factors (confounders) (cohort studies); the evidence table should contain sufficient details on these procedures.

2. Provide data per treatment group on the most important prognostic factors ((potential) confounders).

3. For case-control studies, provide sufficient detail on the procedure used to match cases and controls.

4. For cohort studies, provide sufficient detail on the (multivariate) analyses used to adjust for (potential) confounders.

| **Study**  **First author, year** | **Appropriate and clearly focused question?^1^**  **Yes/no/unclear** | **Comprehensive and systematic literature search?^2^**  **Yes/no/unclear** | **Description of included and excluded studies?^3^**  **Yes/no/unclear** | **Description of relevant characteristics of included studies?^4^**  **Yes/no/unclear** | **Appropriate adjustment for potential confounders in observational studies?^5^**  **Yes/no/unclear/not applicable** | **Assessment of scientific quality of included studies?^6^**  **Yes/no/unclear** | **Enough similarities between studies to make combining them reasonable?^7^**  **Yes/no/unclear** | **Potential risk of publication bias taken into account?^8^**  **Yes/no/unclear** | **Potential conflicts of interest reported?^9^**  **Yes/no/unclear** |
| --- | --- | --- | --- | --- | --- | --- | --- | --- | --- |
| Van der Heijden, 2015 | Yes | Yes | Yes | yes | Not applicable | Yes | Yes | Yes, for included studies    For future updates of the review, we will explore the possibility of publication bias using a funnel plot if there are data from at least 10 trials available for pooling | Yes  None declared |

Table 1.7 Quality assessment for systematic reviews of RCTs and observational studies

Based on AMSTAR checklist (Shea, 2007; BMC Methodol 7: 10; doi:10.1186/1471-2288-7-10) and PRISMA checklist (Moher, 2009; PLoS Med 6: e1000097; doi:10.1371/journal.pmed1000097)

1. Research question (PICO) and inclusion criteria should be appropriate and predefined.

2. Search period and strategy should be described; at least Medline searched; for pharmacological questions at least Medline + EMBASE searched.

3. Potentially relevant studies that are excluded at final selection (after reading the full text) should be referenced with reasons.

4. Characteristics of individual studies relevant to research question (PICO), including potential confounders, should be reported.

5. Results should be adequately controlled for potential confounders by multivariate analysis (not applicable for RCTs).

6. Quality of individual studies should be assessed using a quality scoring tool or checklist (Jadad score, Newcastle-Ottawa scale, risk of bias table et cetera).

7. Clinical and statistical heterogeneity should be assessed; clinical: enough similarities in patient characteristics, intervention and definition of outcome measure to allow pooling? For pooled data: assessment of statistical heterogeneity using appropriate statistical tests (for example Chi-square, I^2^)?

8. An assessment of publication bias should include a combination of graphical aids (for example funnel plot, other available tests) and/or statistical tests (for example Egger regression test, Hedges-Olken). Note: If no test values or funnel plot included score “no”. Score “yes” if mentions that publication bias could not be assessed because there were fewer than 10 included studies.

9. Sources of support (including commercial co-authorship) should be reported in both the systematic review and the included studies. Note: To get a “yes,” source of funding or support must be indicated for the systematic review AND for each of the included studies.

| **Study reference**  (first author, publication year) | **Describe method of randomisation^1^** | **Bias due to inadequate concealment of allocation?^2^**  (unlikely/likely/unclear) | **Bias due to inadequate blinding of participants to treatment allocation?^3^**  (unlikely/likely/unclear) | **Bias due to inadequate blinding of care providers to treatment allocation?^3^**  (unlikely/likely/unclear) | **Bias due to inadequate blinding of outcome assessors to treatment allocation?^3^**  (unlikely/likely/unclear) | **Bias due to selective outcome reporting on basis of the results?^4^**  (unlikely/likely/unclear) | **Bias due to loss to follow-up?^5^**  (unlikely/likely/unclear) | **Bias due to violation of**  **intention to treat analysis?^6^**  (unlikely/likely/unclear) |
| --- | --- | --- | --- | --- | --- | --- | --- | --- |
| Hott, 2019/  Hott, 2020 | The randomization sequence was computer generated with blocks of a variable size, stratified by sex, and unknown to anyone in the research team. The sequence was concealed in opaque envelopes, stored by a nurse not otherwise involved in the study, and delivered sequentially to the study physiotherapist at randomization. | Unlikely | Unclear  Patients in the control group were lost to follow up since they were dissatisfied with the allocation | Unlikely  Physiotherapists providing the interventions were blinded to baseline measures. | Unlikely  Members of the research team who handled outcome measures were blinded to treatment allocation. Data analysis and writing of the manuscript were performed blinded until consensus about the interpretation was reached | Unlikely | Unclear | Unlikely  The principle of intention to treat was used in the main analysis. |
| Rathleff, 2015 | The four schools were randomised either to patient education or patient education and exercise therapy using a computergenerated sequence developed by the main investigator (MSR).  Cluster randomisation was chosen to minimise the contamination between individuals, which could occur if more than one adolescent in each class were diagnosed with PFP but randomised to different treatment groups. | Unlikely | Unlikely | Unclear | Unclear  The first author and a statistician not involved in the study performed all analyses. They were not blinded to group allocation during the analyses. | Unlikely | Unclear  Follow-up rate ranged from 73% to 91% with a 91% follow-up rate at the primary endpoint at 12 months. | Unlikely |
| Saad, 2018 | The subjects were randomly allocated to one of four groups: quadriceps strengthening group (QG), hip strengthening group (HG), stretching group (SG) or a control group(CG) (no treatment). The randomization schedule was generated using R 2.7.2 statistical software. The allocation was concealed by the use of consecutively numbered, sealed and opaque envelopes. | Unlikely | Unlikely | Unclear  One provider treated all groups | Unlikely  Assessor was blinded | Unlikely | Unlikely | Unlikely |

Table 1.8 Risk of bias table for intervention studies (randomized controlled trials)

1. Randomisation: generation of allocation sequences have to be unpredictable, for example computer generated random-numbers or drawing lots or envelopes. Examples of inadequate procedures are generation of allocation sequences by alternation, according to case record number, date of birth or date of admission.

2. Allocation concealment: refers to the protection (blinding) of the randomisation process. Concealment of allocation sequences is adequate if patients and enrolling investigators cannot foresee assignment, for example central randomisation (performed at a site remote from trial location) or sequentially numbered, sealed, opaque envelopes. Inadequate procedures are all procedures based on inadequate randomisation procedures or open allocation schedules.

3. Blinding: neither the patient nor the care provider (attending physician) knows which patient is getting the special treatment. Blinding is sometimes impossible, for example when comparing surgical with non-surgical treatments. The outcome assessor records the study results. Blinding of those assessing outcomes prevents that the knowledge of patient assignement influences the proces of outcome assessment (detection or information bias). If a study has hard (objective) outcome measures, like death, blinding of outcome assessment is not necessary. If a study has “soft” (subjective) outcome measures, like the assessment of an X-ray, blinding of outcome assessment is necessary.

4. Results of all predefined outcome measures should be reported; if the protocol is available, then outcomes in the protocol and published report can be compared; if not, then outcomes listed in the methods section of an article can be compared with those whose results are reported.

5. If the percentage of patients lost to follow-up is large, or differs between treatment groups, or the reasons for loss to follow-up differ between treatment groups, bias is likely. If the number of patients lost to follow-up, or the reasons why, are not reported, the risk of bias is unclear.

6. Participants included in the analysis are exactly those who were randomized into the trial. If the numbers randomized into each intervention group are not clearly reported, the risk of bias is unclear; an ITT analysis implies that (a) participants are kept in the intervention groups to which they were randomized, regardless of the intervention they actually received, (b) outcome data are measured on all participants, and (c) all randomized participants are included in the analysis.

# Summary of literature

The Cochrane systematic review of van der Heijden (2015) described the effects of exercise therapy on pain, function and recovery in adolescents and adults with PFP. A total of 31 randomized and quasi-randomized studies, comprising 1690 participants, were included in this review. Different comparisons were included: exercise therapy versus control, exercise therapy versus other conservative therapy, hip and knee exercises versus knee exercises. To answer the clinical question, only the data of the eight studies included in the comparison ‘exercise therapy versus control’ were extracted from this review (Abrahams 2003; Clark 2000; Fukuda 2010; Herrington 2007; Loudon 2004; Moyano 2013; Song 2009; Van Linschoten 2009). These studies included different types of exercise therapy and different control groups: as described in Table 1.9. Overall, there was a moderate risk of bias (ROB) in these studies, given the uncertainty about or lack of blinding of participants, personnel, and outcome assessors.

In addition to the review, Hott (2019) and Hott (2020) describe the same single-blind RCT. In this RCT three groups were compared; knee exercises (n=37), hip exercises (n=39) and free physical activity (n=36) on function, at different times of follow up: 3 months (Hott, 2019) and 12 months (Hott, 2020). Details of the exercise therapy and control groups can be found in Table 1.9.

Rathleff (2015) described a cluster RCT. A total of 121 adolescents with PFP participated, the experimental group received exercise therapy and education and the control group received education alone. The effects were evaluated on patients’ satisfaction and recovery at eight weeks postintervention.

The RCT of Saad (2018) compared three different exercises groups (8 weeks intervention) and a control group. A total of 40 recreational female athletes with PFP participated. The effects were evaluated on pain and function at eight weeks postintervention.

|  | **Intervention group** | **Control group** |
| --- | --- | --- |
| Abrahams (2003) | traditional exercise protocol: semi squat in neutral to 30 degrees knee flexion held for 2 seconds with subsequent straightening of the knee and rising: 15 repetitions, 3 times daily, for 6 weeks | waiting list controls |
|  | traditional exercise protocol with thigh adduction and tibia medial rotation during eccentric squat 15 repetitions, 3 times daily, for 6 weeks |  |
| Clark (2000) | Exercise + tape (+stretching and education): 6 sessions and daily training at home  Exercise included wall squat, sit to stand, proprioceptive balance, specific exercises for gluteus medius and maximus, progressive step-down exercises  3 months | education alone (no treatment) |
|  | Exercise (+stretching and education): 6 sessions and daily training at home  Exercise included wall squat, sit to stand, proprioceptive balance, specific exercises for gluteus medius and maximus, progressive step-down exercises  3 months |  |
|  | Tape (+education): 6 sessions and daily at home |  |
| Fukuda (2010) | Knee exercise group: Knee exercises including iliopsoas strengthening in non-weight bearing, seated knee extension  90°-45°, leg press 0°-45°, squatting 0°-45°  3 treatment sessions per week for 4 weeks | no treatment |
|  | knee and hip exercise group: Knee and hip exercises including iliopsoas strengthening in non-weight bearing, seated knee extension 90°-45°, leg press 0°-45°, squatting 0°-45°, hip abduction against elastic band (standing), hip abduction  with weights (side lying), hip external rotation against elastic band (sitting), side-stepping against elastic band, 3 x 1-minute lateral rotator muscles  3 treatment sessions per week for 4 weeks |  |
| Herrington (2007) | Multi Joint Weight Bearing (= CKC) including leg press exercise in a seated position from 90° of knee flexion to full extension  6 weeks, 3 times per week | no treatment |
|  | Single Joint Non-Weight Bearing (= OKC) including knee extension exercises in a seated position from 90° of knee flexion to full extension  6 weeks, 3 times per week |  |
| Hott (2019&2020) | Knee- focused exercise: straight-leg raises in the supine position, supine terminal knee extensions (from 10° of flexion to full extension), and a mini-squat (45° of flexion) with the back supported against the wall (to reduce stabilizing requirements from the hip muscles).  Hip-focused exercise: (side-lying hip abduction, hip external rotation (clam shell), and prone hip extension. These exercises were intended to maximally isolate the hip abductors, extensors, and external rotators without stimulating the quadriceps muscles.  3 sessions per week (one supervised and two home sessions) for 6 weeks.  Initial dosage was three sets of 10 repetitions for each exercise, progressing to a maximum three sets of 20 repetitions.  At inclusion, all participants attended an individual 1-hour consultation with a specialist in physical medicine and rehabilitation | Physical activity: encouragement of physiotherapist to be physically active in accordance with the patient education component but received no specific exercise regime.  At inclusion, all participants attended an individual 1-hour consultation with a specialist in physical medicine and rehabilitation. |
| Loudon (2004) | supervised exercise programme twice a week for 4 weeks, plus 1 physical therapy visit at 6 weeks and 1 at 8 weeks, and additional home exercises  Exercises included quadriceps exercises starting with isometrics followed by straight leg raises followed by closed kinetic chain, such as leg press, mini squat, step-up, lunge and balance and reach | information leaflet (no treatment) orthotics and taping if indicated |
|  | home exercise programme + 5 physical therapy visits, 8 weeks |  |
| Moyano (2013) | 'Classic stretching protocol' (stretching and exercises for hip and knee muscles) and quadriceps strengthening exercises  3 times per week for 16 weeks | Health educational materials |
|  | Proprioceptive neuromuscular facilitation stretching applied to hamstrings and quadriceps and, after the 4th week, aerobic exercise 45 minutes  3 times per week for 16 weeks |  |
| Rathleff (2015) | patient education (see control group) combined with exercise therapy  The exercise therapy consisted of a combination of supervised group training sessions (3x pw for 3 months, consisting of neuromuscular training, strength training, mobilisation and stretching) and unsupervised home-based exercises (daily 15 min of quadriceps and hip muscle retraining and stretching). In addition, taping corrections were applied if adolescents achieved a minimum pain reduction of 50% during a two-leg squat. | patient education provided by physiotherapist one-on-one to the adolescents and their parents. The 30-minute session consisted of pain management; how to modify physical activity using pacing and load management strategies; information on optimal knee alignment during daily tasks; and responses to questions of the patient and parents. Patients also received an information leaflet. |
| Saad (2018) | hip strengthening  2x pw 50 min individual session  8 weeks | no treatment |
|  | quadriceps strengthening  2x pw 50 min individual session  8 weeks |  |
|  | stretching exercises for hip and knee stabilizers  2x pw 50 min individual session  8 weeks |  |
| Song (2009) | Leg-press exercise only (0-45^o^) (knee)  3 times a week during 8 weeks | Health educational material |
|  | Hip adduction combined with leg-press exercise (knee + hip)  3 times a week during 8 weeks |  |
| Van Linschoten (2009) | Exercise therapy including static and dynamic exercises for quadriceps, adductor and gluteal muscles: 9 times in 6 weeks + daily at home for 3 months | usual care ('wait and see policy') |

Table 1.9 Intervention types and control groups

# Results

Pain (crucial)

Pain was measured by a visual analogue scale (VAS) or numerical (pain) rating scale (N(P)RS).

Pain during activity in the short-term (≤ 3 months) was presented in five studies included in the review (Clark, 2000; Fukuda, 2010; Herrington, 2007; Van Linschoten, 2009; n=375). In addition, Saad (2018, n=40) reported pain intensity for the four study groups, it was not specified whether or not this pain score was obtained during activity. In the meta-analysis (Figure 1.1), the data of the control groups were split for studies that included multiple comparisons, so that the individual results of each intervention could be presented while avoiding double counting of those in the control group. The small study of Saad (2018) has a large impact due to the four groups, see Figure 1.1. The pooled data resulted in a mean difference (MD) of -2.24 (95%CI: -3.05 to -1.43), favoring exercise therapy.

Two studies in the Cochrane review determined the long-term effect of exercise therapy on pain during activity (Clark, 2000; van Linschoten, 2009; n=180). The MD was -1.03 (95%CI -1.89 to -0.17), favouring exercise therapy.


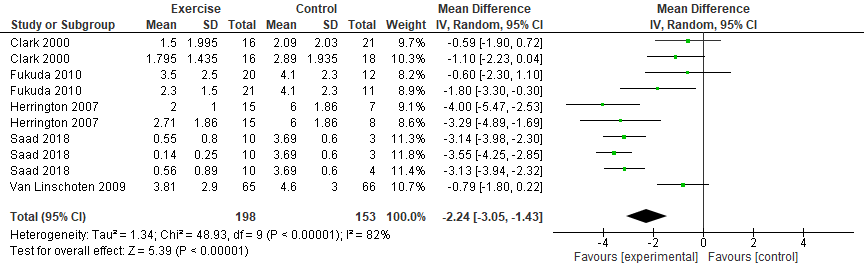


Figure 1.1 Pooled results exercise therapy on pain short-term

Two studies in the Cochrane review determined the long-term effect of exercise therapy on pain during activity (Clark, 2000; van Linschoten, 2009; n=180). The MD was -1.03 (95%CI -1.89 to -0.17), favouring exercise therapy (Figure 1.2).


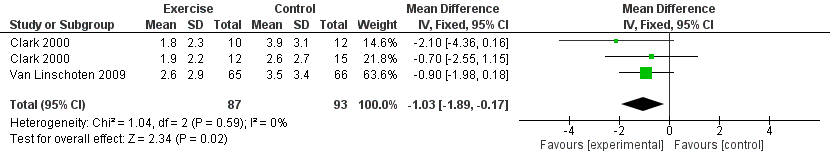


Figure 1.2. Pooled results exercise therapy on pain long-term

Level of evidence of the literature

The level of evidence regarding the outcome pain (short-term effects) started at high as it was based on randomized controlled trials, but was downgraded by 2 levels due to study limitations (risk of bias, -1), and heterogeneity in study results (inconsistency, -1). The final level is low.

The level of evidence regarding the outcome pain (long-term effects) started at high as it was based on RCTs, but was downgraded by 2 levels due to study limitations (risk of bias, -1), and limited number of included patients (imprecision, -1). The final level is low.

Function (crucial)

Functional ability was scored with the Anterior Knee Pain Scale (AKPS) (Kujala 1993), (modified) function scale (Werner 1993), Lysholm score (Lysholm 1982), and (modified)Functional Index Questionnaire (MFIQ 0 to 16/100). A higher score means better function. Function (short-term effects) was presented in nine studies (Abrahams 2003; Clark, 2000; Fukuda, 2010; Herrington, 2007; Hott, 2020; Loudon, 2004; Saad, 2018; Song, 2009; Van Linschoten, 2009; (n=673). The meta-analysis showed a SMD of 0.87 (95%CI: 0.45 to 1.30), favoring exercise therapy (Figure 1.3). To interpret these results, the SMD was multiplied with the pooled SD (10.33) of all studies using the AKPS (Fukuda 2010; Herrington, 2007; Hott, 2020; Loudon, 2004; Saad, 2018; Van Linschoten, 2009). The mean difference in functional ability was estimated at 8.98 higher (95%CI: 4.65 to 13.43) favoring the exercise group, this is not a clinically important difference.


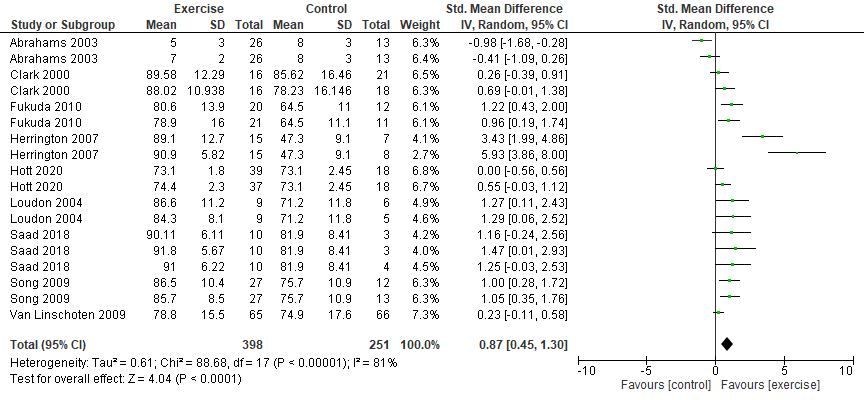
Figure 1.3 Pooled results exercise therapy on function short-term

Function (long-term effects) was presented in three studies in the Cochrane review (Clark, 2000; Moyano, 2013; Van Linschoten, 2009; 274 participants). Besides, long-term function was reported by Hott, 2020 (n=112) using the AKPS score. The pooled data resulted in a SMD of 0.93 (95%CI: -0.02 to 1.89), favoring exercise therapy (Figure 1.4). To interpret these results, the SMD was multiplied with the pooled SD (11.98) of all studies using the AKPS (Hott, 2020; Moyano, 2013; Van Linschoten, 2009). The mean difference in functional ability was estimated at 11.14 higher (95%CI: -0.23 to 23.72) favouring the exercise group.


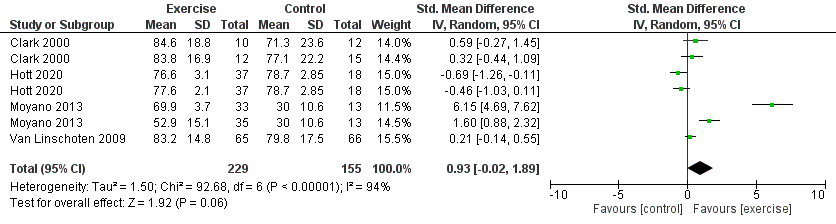
Figure 1.4 Pooled results exercise therapy on function long-term

Level of evidence of the literature

The level of evidence regarding the outcome function (short-term effects) started at high as it was based on RCTs, but was downgraded by 2 levels to low due to study limitations (risk of bias, -1), and heterogeneity (inconsistency, -1). The final level is low.

The level of evidence regarding the outcome function (long-term effects) started at high as it was based on RCTs, but was downgraded by 3 levels to very low due to study limitations (risk of bias, -1), limited number of included patients (imprecision, -1), and heterogeneity (inconsistency, -1). The final level is very low.

Return to sport/work (important)

Return to sport/work was not described as an outcome in the included studies.

Level of evidence of the literature

The level of evidence regarding the outcome return to sport/work was not assessed due to lack of studies.

Duration of absenteeism (important)

Duration of absenteeism was not described as an outcome in the included studies.

Level of evidence of the literature

The level of evidence regarding the outcome duration of absenteeism was not assessed due to lack of studies.

Patient satisfaction (important)

Patient satisfaction was reported in the study of Rathleff (2015), patients were asked about their satisfaction with the result of the treatment on a 5-point Likert scale, ranging from “highly satisfied” to “not satisfied at all”. After 3 months 51% of the patients in the education and exercise group (n=62) were satisfied compared to 19% in the education group (n=59), resulting in a risk ratio of 2.63 (95%CI: 1.49 to 4.63). After 12 months, 60% was satisfied in the education and exercise group compared to 36% of the education group, risk ratio is 1.67 (95%CI: 1.12 to 2.49).

Level of evidence of the literature

The level of evidence regarding the outcome patient satisfaction started at high as it was based on a RCT, but was downgraded by 2 levels to low due to study limitations (risk of bias, -1), and limited number of included patients (imprecision, -1). The final level is low.

Recovery (important)

Patient recovery was reported in the review of van der Heijden, 2015. Recovery was measured with the seven-point Likert scale at 3 and 12 months by Van Linschoten (2009) and at 3, 6 and 12 months by Rathleff (2015), and with the number of patients no longer troubled by symptoms at 12 months (Clark, 2000). After 3 months the pooled data from two studies (Van Linschoten 2009; Rathleff, 2015; 243 participants) showed that the risk ratio (RR) is 1.32 favoring exercise therapy, 95%CI 0.91 to 1.92 (Figure 1.5).


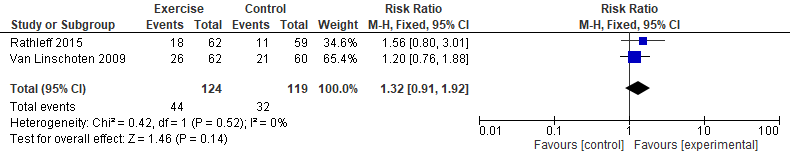


Figure 1.5 Pooled results of exercise therapy on recovery short-term

After 12 months the pooled data from three studies (Clark 2000; Van Linschoten 2009; Rathleff, 2015; 265 participants) showed that the risk ratio (RR) is 1.33 favoring exercise therapy, 95%CI 1.01 to 1.75 (Figure 1.6).


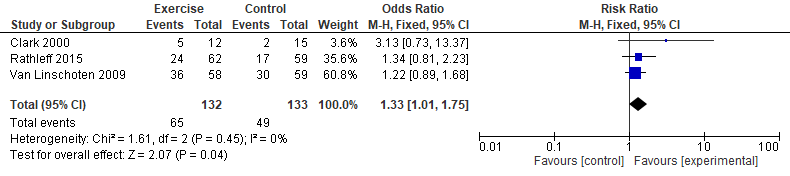


Figure 1.6 Pooled results of exercise therapy on recovery long-term

Level of evidence of the literature

The level of evidence regarding the outcome recovery in the short term started at high as it was based on randomized controlled trials, but was downgraded by 2 levels due to study limitations (risk of bias, -1), and limited number of included patients (imprecision, -1). The final level is low.

The level of evidence regarding the outcome recovery in the long term started at high as it was based on randomized controlled trials, but was downgraded by 2 levels due to study limitations (risk of bias, -1), and limited number of included patients (imprecision, -1). The final level is low.

# Conclusions

Pain (crucial)

| **low GRADE** | The evidence suggests exercise therapy reduces pain in the short-term.  *Sources: van der Heijden (2015); Saad (2018)* |
| --- | --- |

| **low GRADE** | The evidence suggests that exercise therapy results in little to no difference in pain in the long-term.  *Sources: van der Heijden, 2015* |
| --- | --- |

Function (crucial)

| **low GRADE** | The evidence suggests that exercise therapy results in little to no difference in function in the short-term.  *Sources: van der Heijden, 2015; Hott 2020* |
| --- | --- |

| **very low GRADE** | The evidence is very uncertain about the effect of exercise therapy on function in the long-term.  *Sources: van der Heijden, 2015; Hott, 2020* |
| --- | --- |

Return to sport/work (important)

| **- GRADE** | The outcome measure return to sport/work was not reported in the included studies.  *Source: -* |
| --- | --- |

Duration of absenteeism (important)

| **- GRADE** | The outcome measure duration of absenteeism was not reported in the included studies.  *Source: -* |
| --- | --- |

Patient satisfaction (important)

| **low GRADE** | Exercise therapy in combination with education may result in a slightly positive effect on patient satisfaction in the short-term and in the long-term compared to education alone.  *Source: Rathleff, 2015* |
| --- | --- |

Patient recovery (important)

| **low GRADE** | The evidence suggests that exercise therapy with or without education may results in a higher number of recovered patients in the short-term compared to education alone or usual care.  *Sources: Rathleff, 2015; van der Heijden, 2015* |
| --- | --- |

| **low GRADE** | The evidence suggests that exercise therapy with or without education may results in a higher number of recovered patients in the long-term compared to education alone or usual care.  *Sources: Rathleff, 2015; van der Heijden, 2015* |
| --- | --- |

# Considerations – from evidence to recommendation

The literature shows a clinically relevant short-term effect of exercise therapy on pain in patients with PFP. No clinically relevant differences were found for long-term pain and short-term functioning. The strength of evidence of the studies found is low, due to imprecision and risk of bias in the studies. Return to sport and/or work and duration of absence were not described in the included studies. Patient satisfaction was only described in the RCT by Rathleff, 2015, in which patient satisfaction was measured on a 5-point scale. Patients who received exercise therapy in addition to education seem to be more satisfied than when receiving education alone (‘Low GRADE’). There also seems to be a positive effect of exercise therapy on patient-reported recovery.

It remains complicated to summarize the different interventions (duration, exercises, intensity) done in the different studies and evaluated with different outcome measures.

Duration

The duration of the intervention period in the included studies ranged from four weeks (Fukuda, 2010) to four months (Moyano, 2012), with supervision by a physical therapist ranging from once every two weeks (Clark 2000) to three times a week (Fukuda 2010; Herrington 2007; Moyano 2012; Rathleff 2015). The interventions consisted partly of a single exercise (Abrahams, 2003; Herrington, 2007) and partly of exercise programs lasting between 25 minutes (Van Linschoten, 2009) and 50 minutes (Saad, 2018). In some studies, in addition to supervised exercise sessions, daily home exercises were prescribed that lasted between 15 minutes (Rathleff, 2015) and 25 minutes (Van Linschoten, 2009).

Exercises

The exercise programs in the included studies were quadriceps-focussed (e.g., Abrahams, 2003; Loudon, 2004; Herrington, 2007), hip-focussed (Hott, 2019 and 2020; Saad, 2018) or a combination of both (e.g., Clark, 2000; Fukuda, 2010; Rathleff, 2015). Quadriceps-focussed exercise programs include open chain and closed chain exercises (Abrahams 2003; Song 2009; Herrington 2007). For example, leg extension (open chain) were performed between zero and ten degrees of flexion (Hott 2019 and 2020), between 60 and 90 degrees of flexion (Saad, 2018) or between 0 and 90 degrees of flexion (Herrington 2007). For example, as closed chain exercises, the leg press between 0 and 45 degrees of flexion (Fukuda, 2010) and between 0 and 90 degrees of flexion (Loudon, 2004; Herrington, 2007) have been used. Other commonly used closed-chain exercises include the squat, the one-leg squat and the step-up (Song, 2009; Fukuda, 2010; Hott, 2019 and 2020; Loudon, 2004; Moyano, 2013). Hip-focussed exercise programs commonly include the clam exercise (Hott, 2019 and 2020), side-lying abduction (Saad, 2018; Hott, 2019 and 2020), hip abduction in stance (Fukuda, 2010), hip external rotation in sitting (Fukuda, 2010), prone hip extensions (Saad, 2018; Hott, 2019 and 2020), seated hip abduction (Saad, 2018) and the side plank (Saad, 2018).

Intensity

The scope of exercises ranged from one series of 15 repetitions three times a day (Abrahams, 2003), to four series of six repetitions (Herrington, 2007). For example, isometric exercises involved three one-minute load times (Saad, 2018). It is often unclear how exercise intensities were determined, what the criteria for determining those intensities were, and whether/how over the course of the intervention period intensities were adjusted based on decreasing pain, improved motor control, and recruitment (Clark, 2000; Abrahams, 2003; Loudon, 2004; Van Linschoten, 2009). In the study by Rathleff, 2015, exercise intensity was initially determined using three criteria: (1) good movement quality (control of hip, knee and foot), (2) completion of the number of repetitions, (3) no increase in pain after exercise or the next morning. The exercises were then weighted over the intervention period using a build-up in exercises.

It is often unclear whether subjects actually performed the exercise program under supervision or at home as planned (Clark, 2000; Abrahams, 2003; Fukuda, 2010; Moyano, 2012). Only the study by Rathleff, 2015 provides an explicit subgroup analysis, showing that patients with high compliance (>80% of all supervised exercise sessions present) were more likely to recover.

Study populations and subgroups

In the included studies, there is a certain range of characteristics of subjects in terms of gender and age. In general, both women and men were included as subjects. in the study by Herrington, 2007 only men participated and in the study by Fukuda, 2010 only women participated. In general, the studies were conducted on young adult subjects (18-30 years old). The study by Rathleff, 2015 involved adolescents (mean age of seventeen years) and the study by Moyano, 2012 involved adults (mean age of 40 years).

The main component of the interventions studied was a form of exercise therapy that focused on activating quadriceps and hip muscles. Seven of the studies included also included stretching exercises of the major muscle groups (mostly quadriceps and hamstrings). The expert panel believes that in patients with shortening of these muscle groups, in individual cases, the activating quadriceps and hip muscle exercises should be combined with stretching exercises for shortened muscle groups.

Expectations and values of patients (and possibly their caretakers)

To gain insight into the expectations and values of patients with PFP, a questionnaire was prepared and distributed in collaboration with the Netherlands Patients Federation (NPF). The questionnaire was completed by 43 PFP patients, 34 were men (79%). Four patients were under 18 years old (9%), 23 patients aged between 19 and 30 years (54%), six patients between 31 and 40 years (14%) and nine patients between 41 and 65 years (21%) (the age of one patient is unknown). With 28 of these PFP patients (65%), the treatment option "exercises, e.g. under the supervision of the physical therapist" was discussed. A total of 30 of the 43 PFP patients (70%) performed exercises, 15 of these patients (50%) were (very) satisfied with the exercises and nine patients (30%) were (very) dissatisfied. Patients named a number of advantages of this treatment option such as "own direction", "actively dealing with symptoms", "clear explanation of exercises and intended effect for symptoms", "combination of symptom treatment and dosed improving movement" and "strength in the legs/ getting stronger". Patients named some disadvantages of this treatment option such as "consistent execution is difficult and takes time", "no guidance with it, there was also no evaluation", "pain/ too much pain".

Conclusion of the expert panel is: more patients with PFP should be made aware of the potentially beneficial effects of "exercise therapy" treatment option. Sufficient guidance and evaluation should be provided.

Cost

Exercise therapy for patients with PFP is performed under the supervision of the physical therapist, especially patients with more unfavourable prognostic factors (e.g., high VAS-W, low AKPS, long symptom duration, bilateral symptoms) and higher functional demands (e.g., return to sport) should benefit from more contacts with the physical therapist than patients with a more favourable prognostic profile and lower functional demands. There is evidence that exercise therapy in adolescents and young adults is cost-effective (Tan, 2010). Frequency and extent of counseling by the physical therapist will depend on several factors (symptom duration and intensity, goal of rehabilitation, compliance, etc.), but often requires more counseling than the seven or nine treatments currently found in supplemental health insurance in the Netherlands. In the included studies, the number of physical therapist contacts ranged from six contacts in three months (Clark, 2000) to 48 contacts in four months (Moyano, 2012).

The direct costs of physical therapy counseling is for the patient. If the patient has supplemental health insurance and the physical therapist provides contracted care, physical therapy counseling will be at the expense of the supplemental health insurance. In recent years, the extent of physical therapy counseling from the supplemental health insurance has decreased significantly, often limiting the contact to six or nine treatments. In patients with a favourable prognostic profile and lower functional requirements, this limited contact may be sufficiently effective through a targeted combination of exercise therapy supervised by the physical therapist and independent home exercise. In patients with an unfavourable prognostic profile and higher functional demands, six or nine treatments are generally insufficient. Patients in this situation must start paying for physical therapy care themselves, which in practice often leads to non-optimal care and lack of therapy effects, because, after all, not every patient has the financial means. This is an undesirable situation.

Acceptability, feasibility and implementation.

Exercise therapy for patients with PFP should be tailored to the patient's needs and abilities. Exercises are preferably performed under the supervision of the physical therapist. In addition, exercises for home are instructed, which can initially be performed without additional aids or devices. In preparation for return to sport, the physical therapist's facilities (exercise/training gym) will take on a greater role and it may become necessary for the patient to have access to a gym/training gym. To achieve effects of exercise therapy in patients with PFP, patient compliance is a critical factor, as the patient will need to perform exercise therapy either under supervision or independently over an extended period of time. The study by Rathleff, 2015 showed that patients with higher compliance had a higher likelihood of recovery. Numerous barriers to compliance have been described in the literature (e.g., Holt 2020). In the context of exercise therapy in PFP, the main barriers to compliance are: insufficient education about the rationale for exercises and dosages, time frame over which effects can be expected, increase in anterior knee pain due to exercise therapy, and insufficient variation in exercise therapy. This is also supported from the literature (de Oliveira Silva 2020, Winters 2020).

#

# Rationale of the recommendations

Despite the limitations of clinical studies, exercise therapy appears to provide clinically relevant short-term improvement in pain symptoms.

In clinical practice, patients with PFP show a wide range regarding signs and symptoms (symptom duration, intensity, degree of limitations in daily living, coordination and strength of the quadriceps and hip muscles). Thus, a patient with high symptom intensity as well as weak hip muscles may initially benefit more from a pain-free and hip-focused exercise program. Conversely, a patient with low symptom intensity and quadriceps weakness may benefit from a quadriceps-focused exercise program, using "no increase in pain after exercise or the next morning" as a criterion. Therefore, the expert panel believes that choices regarding exercises, scope, intensity and whether or not to accept pain should be made in consultation with the patient. Many of the exercises can initially be performed without equipment. However, additional exercise equipment is needed when sport-specific training and return-to-sport are treatment goals.

# References

Abrahams, S., Guilliford, D., Korkia, P, Prince, J. (2003). The influence of leg positioning in exercise programmes for patellofemoral joint pain. *Journal of Orthopaedic Medicine*;25(3):107-13.

Collins, N. J., Barton, C. J., van Middelkoop, M., Callaghan, M. J., Rathleff, M. S., Vicenzino, B. T., … Crossley, K. M. (2018). 2018 Consensus statement on exercise therapy and physical interventions (orthoses, taping and manual therapy) to treat patellofemoral pain: recommendations from the 5^th^ International Patellofemoral Pain Research Retreat, Gold Coast, Australia, 2017. *British Journal of Sports Medicine*. Retrieved from http://bjsm.bmj.com/content/early/2018/06/20/bjsports-2018-099397.abstract

Clark, D.I., Downing, N., Mitchell, J., et al. (2000). Physiotherapy for anterior knee pain: a randomised controlled trial. *Annals of the Rheumatic Diseases*;59(9):700–4.

Crossley, K. M., van Middelkoop, M., Callaghan, M. J., Collins, N. J., Rathleff, M. S., & Barton, C. J. (2016). 2016 Patellofemoral pain consensus statement from the 4th International Patellofemoral Pain Research Retreat, Manchester. Part 2: recommended physical interventions (exercise, taping, bracing, foot orthoses and combined interventions). *British Journal of Sports Medicine*, *50*, 844–852. https://doi.org/http://dx.doi.org/10.1136/bjsports-2016-096268

Crossley, K.M., Bennell, K.L., Cowan, S.M., Green, S. (2004). Analysis of outcome measures for persons with patellofemoral pain: which are reliable and valid? *Archives of Physical Medicine & Rehabilitation*;85(5):815-22.

de Oliveira Silva, D., Pazzinatto, M. F., Rathleff, M. S., Holden, S., Bell, E., Azevedo, F., & Barton, C. (2020). Patient Education for Patellofemoral Pain: A Systematic Review. *Journal of Orthopaedic & Sports Physical Therapy*, 1–36. https://doi.org/10.2519/jospt.2020.9400

Fukuda, T.Y., Rossetto, F.M., Magalhaes, E., et al. (2010). Short-term effects of hip abductors and lateral rotators strengthening in females with patellofemoral pain syndrome: a randomized controlled clinical trial. *Journal of Orthopaedic and Sports Physical Therapy*;40(11):736-42.

Herrington, L., Al-Sherhi, A. (2007). A controlled trial of weight-bearing versus non-weight-bearing exercises for patellofemoral pain*. Journal of Orthopaedic & Sports Physical Therapy*; 37(4):155-60.

Holden, S., Rathleff, M. S., Thorborg, K., Holmich, P., & Graven-Nielsen, T. (2020). Mechanistic pain profiling in young adolescents with patellofemoral pain before and after treatment: a prospective cohort study. *PAIN;* 161(5).

Holt, C. J., McKay, C. D., Truong, L. K., Le, C. Y., Gross, D. P., & Whittaker, J. L. (2020). Sticking to It: A Scoping Review of Adherence to Exercise Therapy Interventions in Children and Adolescents With Musculoskeletal Conditions. *Journal of Orthopaedic & Sports Physical Therapy*, 1–54. https://doi.org/10.2519/jospt.2020.9715

Hott, A., Brox, J.I., Pripp, A.P. et al. (2019) Effectiveness of Isolated Hip Exercise, Knee Exercise, or Free Physical Activity for Patellofemoral Pain. A Randomized Controlled Trial. *The American Journal of Sports Medicine*;47(6):1312–1322

Hott, A., Brox, J.I., Pripp, A.P. et al. (2020) Patellofemoral pain: One year results of a randomized trial comparing hip exercise, knee exercise, or free activity*. Scand J Med Sci Sports*; 30:741–753.

Loudon, J.D., Gajewsk, B., Goist-Foley, H.L., Loudon, K.L. (2004). The effectiveness of exercise in treating patellofemoral-pain syndrome. *Journal of Sport Rehabilitation*;13:323-42.

Moyano, F., Valenza, M.C., Martin, L. et al. (2013). Effectiveness of different exercises and stretching physiotherapy on pain and movement in patellofemoral pain syndrome: a randomized controlled trial. *Clinical Rehabilitation*;27(5):409-17.

Powers, C. M., Witvrouw, E., Davis, I. S., & Crossley, K. M. (2017). Evidence-based framework for a pathomechanical model of patellofemoral pain: 2017 patellofemoral pain consensus statement from the 4th International Patellofemoral Pain Research Retreat, Manchester, UK: part 3. *British Journal of Sports Medicine*; 51(24), 1713.

Rathleff, M.S., Roos, E.M., Olesen, J.L., Rasmussen, S. (2015) Exercise during school hours when added to patient education improves outcome for 2 years in adolescent patellofemoral pain: a cluster randomised trial. *Br J Sports Med*; 49:406–412.

Saad, M.C., Vasconcelos, R.A., Mancinelli, L.V.O., et al. (2018). Is hip strengthening the best treatment option for females with patellofemoral pain? A randomized controlled trial of three different types of exercises. *Braz J Phys Ther.*;22(5):408-416.

Song, C.Y., Lin, Y.F., Wei, T.C., et al. (2009). Surplus value of hip adduction in leg-press exercise in patients with patellofemoral pain syndrome: a randomized controlled trial. *Physical Therapy*;89(5):409-18.

Tan, S. S., Van Linschoten, R. L., Van Middelkoop, M., Koes, B. W., Bierma-Zeinstra, S. M., & Koopmanschap, M. A. (2010). Cost-utility of exercise therapy in adolescents and young adults suffering from the patellofemoral pain syndrome. *Scandinavian Journal of Medicine & Science in Sports,* 20(4), 568–579. https://doi.org/10.1111/j.1600-0838.2009.00980.x

Van Linschoten, R., Van Middelkoop, M., Berger, M.Y., et al. (2009) Supervised exercise therapy versus usual care for patellofemoral pain syndrome: an open label randomised controlled trial. BMJ;339(7728):1010-13.

Winters, M., Holden, S., Lura, C. B., Welton, N. J., Caldwell, D. M., Vicenzino, B. T., … Rathleff, M. S. (2020). Comparative effectiveness of treatments for patellofemoral pain: a living systematic review with network meta-analysis. *British Journal of Sports Medicine, bjsports-2020-102819*

# Construction of Module 2 ‘Additional conservative treatments for PFP’

#

Scoping Question: What is the value of additional conservative treatments (including taping, brace and foot orthoses) for patients with PFP?

# Search and select

Taping

What is the efficacy of taping in combination with exercises when compared to exercises alone in patients with PFP?

P: patients with PFP (adolescents/adults, non-traumatic)

I: taping in combination with exercises

C: exercises alone (same as intervention)

O: pain, mobility/function, return to sport/work, patient satisfaction, patient recovery

Knee orthoses

What is the efficacy of bracing (knee orthoses) in combination with exercises when compared to exercises alone in patients with PFP?

P: patients with PFP (adolescents/adults, non-traumatic)

I: bracing/knee orthoses in combination with exercises

C: exercises alone (same as intervention)

O: pain, mobility/function, return to sport/work, patient satisfaction, patient recovery

Foot orthoses

What is the efficacy of foot orthoses in combination with exercises when compared to exercises alone in patients with PFP?

P: patients with PFP (adolescents/adults, non-traumatic)

I: foot orthoses in combination with exercises

C: exercises alone (same as intervention)

O: pain, mobility/function, return to sport/work, patient satisfaction, patient recovery

Relevant outcome measures

The expert panel considered pain and function as a critical outcome measures for decision making; and return to sport/work, patient satisfaction and patient recovery as important outcome measures for decision making.

For the outcome pain the Visual Analogue Scale (VAS) and the Numeric Rating Scale (NRS) were used. For the outcome function Kujala score/ Anterior Knee Pain Score (AKPS) are used. Return to sport/work was measured with the Tegner score. Satisfaction with the result of treatment and recovery were usually measured on a Likert scale.

The expert panel defined a difference of 2 cm (of 10 cm) on the VAS or NRS scale as a minimal clinically (patient) important difference for pain, in line with Crossley, 2004. For the Kujala score/ AKPS score regarding function, a difference of 10 point (of 100 points) was defined as a minimal clinically (patient) important difference, in line with Crossley, 2004. In case pooling of the results of individual studies was indicated and the studies used different functional scores, a standardized mean difference of 0.5 was used. A minimal clinically important difference for return to sport/work, patient satisfaction and patient recovery was not predefined.

Search and select (Methods)

For all PICO’s together, the databases Medline (via OVID) and Embase (via Embase.com) were searched with relevant search terms until April 28^th^, 2020. The systematic literature search resulted in 475 hits. Studies were selected based on the following criteria:

- systematic review (including evidence tables, risk of bias assessments, published in 2010 or later) or RCT (published in 2000 or later);
- including patients with PFP, excluding Osgood-Schlatter syndrome, Sinding-Larsen-Johansson syndrome, Iliotibial band syndrome, osteoarthritis;
- comparing treatment with tape, braces/knee orthoses or foot orthoses in combination with exercise therapy, with exercise therapy alone.

Eighty-tree studies were initially selected based on title and abstract screening and checking references from relevant reviews. After reading the full text, 65 studies were excluded (see the table with reasons for exclusion), and 18 studies (three systematic reviews and 15 RCTs from which seven were included in systematic reviews as well) were included.

Results

Regarding taping, one systematic review (including three RCTs meeting inclusion criteria) and five additional RCTs were included in the analysis of the literature. Regarding braces/knee orthoses, one systematic review (including two RCTs meeting inclusion criteria) and three additional RCTs were included in the analysis of the literature. Regarding foot orthoses, one systematic review (including one RCT meeting inclusion criteria) was included in the analysis of the literature. Important study characteristics and results are summarized in the evidence tables. The assessment of the risk of bias is summarized in the risk of bias tables

PICO

| **PICO** | **Concepts** |
| --- | --- |
| P  Patient, population, problem | Patients with PFP |
| I  Intervention | *overige conservatieve behandelingen  1) inlays, insoles, foot orthoses, orthotics  2) taping, tape, kinesio tape 3) brace, bracing, knee support 4) massage  5) dry needling, needle, acupuncture  6) behaviour, behavioral therapy, information, (re-)education, pain management, psychological intervention, psychology 7) walking/running therapy, running training, running retraining, gait 8) bike fitting, bike fit,  9) biomechanics, alignment analysis, tracking, maltracking, malalignment  *10) multimodal treatment |
| C  Comparison |  |
| O  Outcome: | pain, mobility/function, return to sport/work |

Table 2.1 PICO module 2

Key articles: 29925502 OR 31475628 OR 25603546 OR 32034562

| Search | Query | Items found |
| --- | --- | --- |
| #14 | Search (#9 OR #10 OR #11 OR #12) AND #13 | 4 |
| #13 | Search 29925502 31475628 25603546 32034562[uid] | 4 |
| #12 | Search #8 AND #4 | 8 |
| #11 | Search #8 AND #3 | 366 |
| #10 | Search #8 AND #2 | 123 |
| #9 | Search #8 AND #1 | 76 |
| #8 | Search #7 AND ( "2010/01/01"[PDat] : "2030/12/31"[PDat] ) | 625 |
| #7 | Search #5 AND #6 | 1027 |
| #6 | Search ("Orthotic Devices"[Mesh] OR "orthotic"[tiab] OR "orthotics"[tiab] OR "Orthose"[tiab] OR "Orthoses"[tiab] OR "inlays"[tiab] OR "inlay"[tiab] OR "brace"[tiab] OR "braces"[tiab] OR "bracing"[tiab] OR "knee support"[tiab] OR "Tape"[tiab] OR "tapes"[tiab] OR "taping"[tiab] OR "Massage"[Mesh:NoExp] OR "Massage"[tiab] OR "Massages"[tiab] OR "Dry Needling"[Mesh] OR Dry Needl*[tiab] OR "Acupuncture Therapy"[Mesh] OR "Acupuncture"[tiab] OR "Behavior Therapy"[Mesh] OR "Behavioral Therapy"[tiab] OR "behavior therapy"[tiab] OR "behaviour therapy"[tiab] OR "Behavioral Therapies"[tiab] OR "behavior therapies"[tiab] OR "behaviour therapies"[tiab] OR "mindfullness"[tiab] OR "meditation"[tiab] OR "Patient Education as Topic"[Mesh:NoExp] OR "Patient Education"[tiab] OR "patient re-education"[tiab] OR "patient reeducation"[tiab] OR "Consumer Health Information"[Mesh] OR "health literacy"[tiab] OR "information"[tiab] OR "Pain Management"[Mesh] OR "Pain Management"[tiab] OR "Psychology"[Mesh] OR Psycholog*[tiab] OR "Psychotherapy"[Mesh:NoExp] OR Psychotherap*[tiab] OR "Walking"[Mesh] OR "walk"[tiab] OR "walks"[tiab] OR "Walking"[tiab] OR "ambulation"[tiab] OR "gait"[tiab] OR "Running"[Mesh] OR "Running"[tiab] OR "jogging"[tiab] OR "Bicycling"[Mesh] OR "bicycling"[tiab] OR "biking"[tiab] OR "bike"[tiab] OR "bikes"[tiab] OR "Combined Modality Therapy"[Mesh:NoExp] OR "Combined Modality"[tiab] OR "multi-modal"[tiab] OR "multimodal"[tiab]) | 2187120 |
| #5 | Search ("Patellofemoral Pain Syndrome"[Mesh] OR "Patellofemoral Pain"[tiab] OR "Anterior Knee Pain" OR "Patellofemoral Syndrome"[tiab] OR patellofemoral dysfunction*[tiab] OR patellofemoral disorder*[tiab] OR "PFP"[ti] OR "PFPS"[ti] OR "retro-patellar pain"[tiab] OR "retropatellar pain"[tiab] OR "peri-patellar pain"[tiab] OR "anterior knee pain"[tiab] OR Patella chondropath*[tiab] OR Patellar chondropath*[tiab] OR Patella chrondromalacia*[tiab] OR Patellar chrondromalacia*[tiab] OR "runner's knee"[tiab] OR "lateral facet compression syndrome"[tiab] OR chondromalacia patella*[tiab]) | 3788 |
| #4 | Search ("Practice Guideline" [Publication Type] OR "Practice Guidelines as Topic"[Mesh] OR "practice guideline"[tiab] OR "practice guidelines"[tiab]) | 155620 |
| #3 | Search ("cohort studies"[mesh] OR "case-control studies"[mesh] OR "comparative study"[pt] OR "risk factors"[mesh] OR "cohort"[tw] OR "compared"[tw] OR "groups"[tw] OR "case control"[tw] OR "multivariate"[tw]) | 7854601 |
| #2 | Search ((random*[tiab] AND (controlled[tiab] OR control[tiab] OR placebo[tiab] OR versus[tiab] OR vs[tiab] OR group[tiab] OR groups[tiab] OR comparison[tiab] OR compared[tiab] OR arm[tiab] OR arms[tiab] OR crossover[tiab] OR cross-over[tiab]) AND (trial[tiab] OR study[tiab])) OR ((single[tiab] OR double[tiab] OR triple[tiab]) AND (masked[tiab] OR blind*[tiab]))) | 726105 |
| #1 | Search ("Meta-Analysis as Topic"[Mesh] OR “Meta-Analysis”[Publication Type] OR metaanaly*[tiab] OR metanaly*[tiab] OR meta-analy*[tiab] OR meta synthes*[tiab] OR metasynthes*[tiab] OR meta ethnograph*[tiab] OR metaethnograph*[tiab] OR meta summar*[tiab] OR metasummar*[tiab] OR meta-aggregation[tiab] OR metareview[tiab] OR meta-review[tiab] OR overview of reviews[tiab] OR ((systematic*[ti] OR scoping[ti] OR umbrella[ti] OR meta-narrative[ti] OR metanarrative[ti] OR evidence based[ti]) AND (review*[ti] OR overview*[ti])) OR ((evidence[ti] OR narrative[ti] OR metanarrative[ti] OR qualitative[ti]) AND synthesis[ti]) OR systematic review[pt] OR prisma[tiab] OR preferred reporting items[tiab] OR quadas*[tiab] OR systematic review*[tiab] OR systematic literature[tiab] OR structured literature search[tiab] OR systematic overview*[tiab] OR scoping review*[tiab] OR umbrella review*[tiab] OR mapping review*[tiab] OR systematic mapping[tiab] OR evidence synthes*[tiab] OR narrative synthesis[tiab] OR metanarrative synthesis[tiab] OR research synthesis[tiab] OR qualitative synthesis[tiab] OR realist synthesis[tiab] OR realist review[tiab] OR realist evaluation[tiab] OR systematic qualitative review[tiab] OR mixed studies review[tiab] OR mixed methods synthesis[tiab] OR mixed research synthesis[tiab] OR quantitative literature review[tiab] OR systematic evidence review[tiab] OR evidence-based review[tiab] OR comprehensive literature search[tiab] OR integrated review*[tiab] OR integrated literature review[tiab] OR integrative review*[tiab] OR integrative literature review*[tiab] OR structured literature review*[tiab] OR systematic search and review[tiab] OR meta-narrative review*[tiab] OR metanarrative review[tiab] OR systematic narrative review[tiab] OR systemic review[tiab] OR systematized review[tiab] OR systematic research synthesis[tiab] OR bibliographic*[tiab] OR hand-search*[tiab] OR handsearch*[tiab] OR manual search*[tiab] OR searched manually[tiab] OR manually searched[tiab] OR journal database*[tiab] OR review authors independently[tiab] OR reviewers independently[tiab] OR independent reviewers[tiab] OR independent review authors[tiab] OR electronic database search*[tiab] OR (study selection[tiab] AND data extraction[tiab]) OR (selection criteria[tiab] AND data collection[tiab]) OR (selection criteria[tiab] AND data analysis[tiab]) OR (evidence acquisition[tiab] AND evidence synthesis[tiab]) OR (pubmed[tiab] AND embase[tiab]) OR (medline[tiab] AND embase[tiab]) OR (pubmed[tiab] AND cochrane[tiab]) OR (medline[tiab] AND cochrane[tiab]) OR (embase[tiab] AND cochrane[tiab]) OR (pubmed[tiab] AND psycinfo[tiab]) OR (medline[tiab] AND psycinfo[tiab]) OR (embase[tiab] AND psycinfo[tiab]) OR (cochrane[tiab] AND psycinfo[tiab]) OR (pubmed[tiab] AND web of science[tiab]) OR (medline[tiab] AND web of science[tiab]) OR (embase[tiab] AND web of science[tiab]) OR (psycinfo[tiab] AND web of science[tiab]) OR (cochrane[tiab] AND web of science[tiab]) OR ((literature[ti] OR qualitative[ti] OR quantitative[ti] OR integrated[ti] OR integrative[tiab] OR rapid[ti] OR short[ti] OR critical*[ti] OR mixed stud*[ti] OR mixed method*[ti] OR focused[ti] OR focussed[ti] OR structured[ti] OR comparative[ti] OR comparitive[ti] OR evidence[ti] OR comprehensive[ti] OR realist[ti]) AND (review*[ti] OR overview*[ti]) AND (literature search[tiab] OR structured search[tiab] OR electronic search[tiab] OR search strategy[tiab] OR gray literature[tiab] OR grey literature[tiab] OR Review criteria[tiab] OR eligibility criteria[tiab] OR inclusion criteria[tiab] OR exclusion criteria[tiab] OR predetermined criteria[tiab] OR included studies[tiab] OR identified studies[tiab] OR (systematic search[tiab] AND literature[tiab]) OR strength of evidence[tiab] OR citation*[tiab] OR references[tiab] OR database search*[tiab] OR electronic database*[tiab] OR data base search*[tiab] OR electronic data-base*[tiab] OR search criteria[tiab] OR study selection[tiab] OR data extraction[tiab] OR methodological quality[tiab] OR methodological characteristics[tiab] OR methodologic quality[tiab] OR methodologic characteristics[tiab])) OR ((literature review[tiab] OR literature search*[tiab]) AND (structured search[tiab] OR electronic search[tiab] OR Search strategy[tiab] OR gray literature[tiab] OR grey literature[tiab] OR review criteria[tiab] OR eligibility criteria[tiab] OR inclusion criteria[tiab] OR exclusion criteria[tiab] OR predetermined criteria[tiab] OR included studies[tiab] OR identified studies[tiab] OR (systematic search[tiab] AND literature[tiab]) OR strength of evidence[tiab] OR citation*[tiab] OR references[tiab] OR database search*[tiab] OR electronic database*[tiab] OR data base search*[tiab] OR electronic data-base*[tiab] OR search criteria[tiab] OR study selection[tiab] OR data extraction[tiab] OR methodological quality[tiab] OR methodological characteristics[tiab] OR methodologic quality[tiab] OR methodologic characteristics[tiab]))) NOT ("Comment" [Publication Type] OR "Letter" [Publication Type]) NOT (“Animals”[Mesh] NOT “Humans”[Mesh]) |  |

Table 2.2 Pubmed search

| **No.** | **Query** | **Results** |
| --- | --- | --- |
| #13 | #9 AND #4 | **7** |
| #12 | #9 AND #3 | **152** |
| #11 | #9 AND #2 | **128** |
| #10 | #9 AND #1 | **44** |
| #9 | #8 AND [2010-2030]/py | **495** |
| #8 | #7 NOT ('conference abstract'/it OR 'editorial'/it OR 'letter'/it OR 'note'/it) NOT (('animal experiment'/exp OR 'animal model'/exp OR 'nonhuman'/exp) NOT 'human'/exp) | **793** |
| #7 | #5 AND #6 | **966** |
| #6 | ('orthosis'/exp OR 'orthotic':ti,ab,kw OR 'orthotics':ti,ab,kw OR 'orthose':ti,ab,kw OR 'orthoses':ti,ab,kw OR 'inlays':ti,ab,kw OR 'inlay':ti,ab,kw OR 'brace':ti,ab,kw OR 'braces':ti,ab,kw OR 'bracing':ti,ab,kw OR 'knee support':ti,ab,kw OR 'tape':ti,ab,kw OR 'tapes':ti,ab,kw OR 'taping':ti,ab,kw OR 'massage'/exp OR 'massage':ti,ab,kw OR 'massages':ti,ab,kw OR 'dry needling'/de OR dry) AND needl*:ti,ab,kw OR 'acupuncture'/exp OR 'acupuncture':ti,ab,kw OR 'behavior therapy'/exp OR 'behavioral therapy':ti,ab,kw OR 'behavior therapy':ti,ab,kw OR 'behaviour therapy':ti,ab,kw OR 'behavioral therapies':ti,ab,kw OR 'behavior therapies':ti,ab,kw OR 'behaviour therapies':ti,ab,kw OR 'mindfulness'/exp OR 'mindfullness':ti,ab,kw OR 'meditation'/exp OR 'meditation':ti,ab,kw OR 'patient education'/de OR 'patient education':ti,ab,kw OR 'patient re-education':ti,ab,kw OR 'patient reeducation':ti,ab,kw OR 'consumer health information'/de OR 'health literacy':ti,ab,kw OR 'information':ti,ab,kw OR 'pain management':ti,ab,kw OR 'psychology'/exp OR psycholog*:ti,ab,kw OR 'psychotherapy'/de OR psychotherap*:ti,ab,kw OR 'walking'/exp OR 'walk':ti,ab,kw OR 'walks':ti,ab,kw OR 'walking':ti,ab,kw OR 'ambulation':ti,ab,kw OR 'gait':ti,ab,kw OR 'running'/de OR 'running':ti,ab,kw OR 'jogging':ti,ab,kw OR 'cycling'/de OR 'bicycling':ti,ab,kw OR 'biking':ti,ab,kw OR 'bike':ti,ab,kw OR 'bikes':ti,ab,kw OR 'combined modality':ti,ab,kw OR 'multi-modal':ti,ab,kw OR 'multimodal':ti,ab,kw | **2776901** |
| #5 | 'patellofemoral pain syndrome'/exp OR 'patellofemoral pain':ti,ab,kw OR 'patellofemoral syndrome':ti,ab,kw OR 'patellofemoral dysfunction*':ti,ab,kw OR 'patellofemoral disorder*':ti,ab,kw OR 'pfp':ti OR 'pfps':ti OR 'retro-patellar pain':ti,ab,kw OR 'retropatellar pain':ti,ab,kw OR 'peri-patellar pain':ti,ab,kw OR 'anterior knee pain':ti,ab,kw OR 'patella chondropath*':ti,ab,kw OR 'patellar chondropath*':ti,ab,kw OR 'patella chrondromalacia*':ti,ab,kw OR 'patellar chrondromalacia*':ti,ab,kw OR 'runner s knee':ti,ab,kw OR 'lateral facet compression syndrome':ti,ab,kw OR 'chondromalacia patella*':ti,ab,kw | **4835** |
| #4 | 'practice guideline'/de OR 'practice guideline':ti,ab,kw OR 'practice guidelines':ti,ab,kw | **420008** |
| #3 | 'major clinical study'/de OR 'clinical study'/de OR 'case control study'/de OR 'family study'/de OR 'longitudinal study'/de OR 'retrospective study'/de OR 'prospective study'/de OR 'cohort analysis'/de OR ((cohort NEAR/1 (study OR studies)):ab,ti) OR (('case control' NEAR/1 (study OR studies)):ab,ti) OR (('follow up' NEAR/1 (study OR studies)):ab,ti) OR (observational NEAR/1 (study OR studies)) OR ((epidemiologic NEAR/1 (study OR studies)):ab,ti) OR (('cross sectional' NEAR/1 (study OR studies)):ab,ti) | **5217977** |
| #2 | 'clinical trial'/exp OR 'randomization'/exp OR 'single blind procedure'/exp OR 'double blind procedure'/exp OR 'crossover procedure'/exp OR 'placebo'/exp OR 'prospective study'/exp OR rct:ab,ti OR random*:ab,ti OR 'single blind':ab,ti OR 'randomised controlled trial':ab,ti OR 'randomized controlled trial'/exp OR placebo*:ab,ti | **3025856** |
| #1 | 'meta analysis'/de OR cochrane:ab OR embase:ab OR psycinfo:ab OR cinahl:ab OR medline:ab OR ((systematic NEAR/1 (review OR overview)):ab,ti) OR ((meta NEAR/1 analy*):ab,ti) OR metaanalys*:ab,ti OR 'data extraction':ab OR cochrane:jt OR 'systematic review'/de | **493008** |

Table 2.3 Embase results

|  | **EMBASE** | **PubMed** | **Deduplicated** |
| --- | --- | --- | --- |
| SRs | 44 | 76 | 84 |
| RCTs | 128 | 123 | 142 |
| Observational studies | 152 | 366 | 242 |
| Other | 7 | 8 | 9 |
| Total | 331 | 573 | 477 |

Table 2.4 Search results

| 1. **Author and year** | 1. **Reason for exclusion** |
| --- | --- |
| 1. SR |  |
| 1. Barton 2010 | 1. Cochrane review available; references checked |
| 1. Barton 2014 | 1. Cochrane review available; references checked |
| 1. Chang 2015 | 1. Cochrane review available; references checked |
| 1. Collins 2012 | 1. Cochrane review available; references checked |
| 1. Collins 2018 | 1. No evidence tables available; references checked |
| 1. Crossely 2016 | 1. No evidence tables available; references checked |
| 1. Eckenrode 2018 | 1. Does not fit PICO (manual therapy); references checked |
| 1. Lake 2011 | 1. Cochrane review available; references checked |
| 1. Logan 2017 | 1. Cochrane review available; references checked |
| 1. Saltychev 2018 | 1. Cochrane review available; references checked |
| 1. Swart 2012 | 1. Cochrane review available; references checked |
| 1. RCTs after 2017 |  |
| 1. Aliberti 2019 | 1. Wrong intervention (no taping, braces, insoles) |
| 1. Bazett-Jones 2017 | 1. Wrong intervention (no taping, braces, insoles) |
| 1. Behrangrad 2020 | 1. Wrong intervention (no taping, braces, insoles) |
| 1. Bonacci 2020 | 1. Wrong comparison |
| 1. Bonacci 2018 | 1. Wrong comparison |
| 1. Bonanno 2018 | 1. Wrong patientgroup |
| 1. Collins 2017 | 1. Wrong patientgroup |
| 1. dos Santos 2019 | 1. Wrong intervention (no taping, braces, insoles) |
| 1. Drew 2017 | 1. Wrong intervention (no taping, braces, insoles) |
| 1. Emamvirdi 2019 | 1. Wrong intervention (no taping, braces, insoles) |
| 1. Esculier 2018 | 1. Wrong intervention (no taping, braces, insoles) |
| 1. EspÃ­-LÃ³pez 2017 | 1. Wrong intervention (no taping, braces, insoles) |
| 1. Gaitonde 2019 | 1. Narrative review |
| 1. Kolle 2020 | 1. No combination with exercises |
| 1. Korakakis 2018 | 1. Wrong intervention (no taping, braces, insoles) |
| 1. Matthews 2017 | 1. Protocol |
| 1. Matthews 2020 | 1. Wrong comparison |
| 1. Melo 2018 | 1. No combination with exercises |
| 1. Nielsen 2020 | 1. No comparison |
| 1. Priore 2020 | 1. Wrong comparison |
| 1. Prohorova 2019 | 1. Russian |
| 1. Rathleff 2018 | 1. No comparison |
| 1. Selhorst 2020 | 1. No comparison |
| 1. Selhorst 2018 | 1. Wrong intervention (no taping, braces, insoles) |
| 1. Sutlive 2018 | 1. Wrong intervention (no taping, braces, insoles) |
| 1. Talbot 2020 | 1. Wrong intervention (no taping, braces, insoles) |
| 1. Wang 2020 | 1. Wrong patientgroup |
| 1. Yamamoto 2019 | 1. Wrong patientgroup |
| 1. Zahednejad 2017 | 1. Russian |
| 1. Zarei 2020 | 1. Wrong intervention (no taping, braces, insoles) |
| 1. Additional references (after 2000) from SR |  |
| 1. Aytar 2011 | 1. No combination with exercises |
| 1. Banan Khojaste 2016 | 1. Wrong comparison |
| 1. Bolgla 2011 | 1. Cochrane review available; references checked |
| 1. Campolo 2013 | 1. No combination with exercises |
| 1. Collins 2008 | 1. Similar to Collins 2009 |
| 1. Crossley 2002 | 1. Wrong comparison |
| 1. Denton 2005 | 1. Wrong comparison |
| 1. Derassari 2010 | 1. No combination with exercises |
| 1. Johnston 2004 | 1. Wrong design |
| 1. Kalron 2013 | 1. Cochrane review available; references checked |
| 1. Kaya 2010 | 1. No comparison |
| 1. Kurt 2016 | 1. No combination with exercises |
| 1. Kuru 2012 | 1. Wrong comparison |
| 1. Lan 2010 | 1. No comparison |
| 1. Lee 2012 | 1. Wrong design |
| 1. Lee 2013 | 1. No combination with exercises |
| 1. Lewinson 2015 | 1. No combination with exercises |
| 1. Mason 2011 | 1. No combination with exercises |
| 1. Mills 2012 | 1. No combination with exercises |
| 1. Mostamand 2010 | 1. No comparison |
| 1. Mostamand 2011 | 1. No combination with exercises |
| 1. Osario 2013 | 1. No combination with exercises |
| 1. Rathleff 2015 | 1. Wrong intervention (no taping, braces, insoles) |
| 1. Syme 2009 | 1. Wrong comparison |

Table 2.5 Excluded studies

| **Study reference** | **Study characteristics** | **Patient characteristics** | **Intervention (I)** | **Comparison / control (C)** | **Follow-up** | **Outcome measures and effect size** | **Comments** |
| --- | --- | --- | --- | --- | --- | --- | --- |
| Taping |  |  |  |  |  |  |  |
| Callaghan, 2012 | SR and meta-analysis of RCTs  *Literature search up to August 2011*  **A**: Clark, 2000  **B**: Tunay, 2003  **C**: Whittingham, 2004  Study design:  **A**: RCT, parallel  **B**: RCT, parallel  **C**: RCT, parallel,  Setting and Country:  A: UK B:Turkey C:UK, military  Source of funding and conflicts of interest:  SR: University of Manchester, UK; University of Central Lancashire, UK; Department of Health Post Doctoral Awart, UK; Arthritis Research, UK; no conflicts of interest  A: not reported B: not reported C: not reported | Inclusion criteria SR: - RCT and quasi-randomised controlled trials evaluating patellar taping for adults with patellofemoral pain syndrome  - adults, aged 18 and above, diagnosed with patellofemoral pain syndrome.  - patellar taping - outcome: pain; function; activity levels; quality of life - timing immediately after the completion of a treatment programme/preferably at least six months follow-up when taping is used as part of a treatment programme  Exclusion criteria SR:  - treatment after patellar fracture, dislocation or subluxation - history of recurrent dislocation and subluxation - concomitant neurological, rheumatological or cardiovascular problems -outcome measures such  EMG (electromyogram) data, gait analysis, patellar position or  Alignment studied without pain evaluation  *5 studies included (3 relevant for this review)*  Important patient characteristics at baseline:  N, mean age  **A**: I: 20, Not reported per group  C: 20, not reported per group  **B**: I: 20, Not reported per group  C: 20, not reported per group  **C**: age: 18.7 years Ia: n=10 Ib: n=10 C: n=10  *Sex:*  **A**: not reported per group  **B**: not reported per group  **C**: 80% Male  Groups comparable at baseline? not reported | Describe intervention:  **A**: Patellar taping, exercise & education . Tape was applied from the lateral border of the patella  pulling medially and upwards over the medial femoral condyle. Taping in this way should reduce pain  on the squat test and wall/step down test. If this did not eliminate the pain then the taping was repeated in knee flexion. Type of tape used is not described. Six treatments over period of 3 months. Length of time of each treatment not stated.  **B**: Patellar taping, ice and home exercises, treatment for 3 weeks  **C**: Ia: Patellar taping + standardised exercise programme; Active taping technique (McConnel): underwrap  and one corrective strip of tape. Correction of patellar malalignments of tilt, rotation or glide as identified by the treating physiotherapist. Ib: Placebo taping + standardised exercise programme; Placebo taping: underwrap and one strip of tape with no correction of patellar position. | Describe control:  **A**: Exercise & education Education: leaflet “Knee pain in young adults” and sessions on (a) an explanation of the nature of anterior knee pain, the anatomy of the patellofemoral joint, and possible causes of anterior knee pain;  (b) footwear and appropriate sporting activities; (c) pain controlling drugs; (d) stress relaxation techniques, ice and massage; (e) diet and weight advice; and (f) prognosis and self help.  Exercise: stretching to the hamstring, iliotibial band, quadriceps and gastrocnemius muscles. Eccentric, isotonic and isometric strengthening exercises to the lower limb Six treatments over period of 3 months. Length of time of each treatment not stated.  **B**: Ice and home exercises, treatment for 3 weeks  **C**: Exercise programme alone: non–weight-bearing isometric, inner-range isotonic and straight leg raise quadriceps exercises. A variety of weight-bearing exercises (e.g. squats). Stretches for the quadriceps, hamstrings, gastrocnemius, and iliotibial band. No home exercise programme. | End-point of follow-up:  **A**: 3 months, 12 months  **B**: 3 weeks  **C**: 1,2,3,4 weeks  For how many participants were no complete outcome data available?  (intervention/control)  **A:** "10 patients withdrew from the study and these were included on an intention  to treat basis." Participant flow provided.  **B**: not reported  **C**: "All subjects remained in the group to which they were originally assigned." | Outcome measure-1: pain  VAS, after treatment  Effect measure: mean difference [95% CI]:  **A**: 0.81 (-0.44, 2.06)  **B**: 0.35 (-0.43, 1.13) C: -1.45 (-1.91, -0.99)  Pooled effect (random effects model:  -0.16 [95% CI -1.67 to 1.34] favoring taping  Heterogeneity (I^2^): 91.1%  VAS, 12 months  Effect measure: mean difference [95% CI]:  **A**: -0.13 (-1.99, 1.73) (favoring taping  Outcome measure-2: function  FIQ, end of treatment  Effect measure: mean difference [95% CI]:  **C**: 2.5 (-.82, 3.18) (favoring taping  Cincinnati knee activity score, end of treatment  Effect measure: mean difference [95% CI]:  **B**: 8.1 (2.93, 13.27) (favoring taping  WOMAC score, end of treatment  Effect measure: mean difference [95% CI]:  **A**: 1.5 (-6.24, 9.24) (favoring no taping  WOMAC score, 12 months  Effect measure: mean difference [95% CI]:  **A**: -0.8 (-15.24, 13.64) (favoring taping | Facultative:  Brief description of author’s conclusion: the currently available evidence from trials reporting clinically relevant outcomes is low quality and insuLicientto draw conclusions on the eLects of taping, whether used on its own or as part of a treatment programme.  Risk of Bias due to issues regarding blinding and allocation concealment |
| Bracing |  |  |  |  |  |  |  |
| Smith, 2015 | SR and meta-analysis of [RCTs / cohort / case-control studies]  *Literature search up to May 2015*  **A**: Evcik, 2010  **B**: Lun, 2005  Study design: RCT A: RCT, parallel B: RCT, parallel  Setting and Country: A: single center, Turkey B: single center, Canada  Source of funding and conflicts of interest:  [commercial / non-commercial / industrial co-authorship] SR: funding: national institute for health research via Cochrane infrastructure funding to the Cochrane bone, joint and muscle trauma group; conflicts of interest: Benjamin T Drew currently holds a NIHR/HEE Clinical Doctoral Fellowship grant. A: not reported  B: not reported | Inclusion criteria SR: - (quasi) randomised controlled trials evaluating knee orthoses for treating PFPS. - subjectively reporting pain diagnosed by trial authors as 'patellofemoral pain syndrome',  'patellofemoral pain', 'anterior knee pain syndrome', 'patellar  dysfunction', 'chondromalacia patellae', 'patellar syndrome',  'patellofemoral syndrome' or 'chondropathy'. - trials evaluating the use of a knee orthosis - outcome: pain, functional outcomes, quality of life, impact on sporting or occupational participation, resource use/costs of intervention, participant satisfaction, complications of orthoses  Exclusion criteria SR: - cross-over designs - asymptomatic or non-pathological patients - history of fracture, patellar dislocation, patellar tendinopathy, Hoffa’s syndrome, Osgood Schlatter syndrome, Sinding-Larsen-Johansson syndrome, iliotibial band friction syndrome, osteoarthritis rheumatoid arthritis, plica syndromes, or tibiofemoral injury or dysfunction - trials reporting the use of orthoses following operative interventions - mixed populations regarding knee pathology  *5 studies included (2 relevent for this review)*  Important patient characteristics at baseline:  *Number of patients; characteristics important to the research question and/or for statistical adjustment (confounding in cohort studies); for example, age, sex, bmi, ...*  N, mean age  **A**: I: 41 patients, 42.2 (SD 15.3) yrs  C: 45 patients, 41.0 (SD 9.3) yrs  **B**: Ia: 32 patients, 45 knees, 35 (SD11)  Ib: 31 patients, 44 knees, 35 (SD9) yrs  C: 34 patients, 44 knees, 35 (SD 11) yrs  *Sex:*  **A**: I: 15% Male  C: 18% Male  **B**: not reported  Groups comparable at baseline? not reported | Describe intervention:  **A**: knee sleeve + exercise therapy knee sleeve: Altex Patellar  Knee support (Altex Patellar knee support AL-2285C), which is a neoprene sleeve with a patellar cut-out.  This was worn whilst performing the exercises as well as during the day for the six-week study period.  The knee support was only removed at night for sleeping. **B-a**: exercise and knee brace group The brace was a Special FX Knee Brace (Generation II Orthotics, Inc, Richmond, BC). It has a Y-shaped inferior patellar buttress pad and an external  stabilisation strap to help control patellar movement  **B-b**: exercise and knee sleeve group  Knee sleeve constructed with same sleeve material as the patellar brace. No hole was made in the sleeve over the patella | Describe control:  **A**: exercise therapy Standardised protocol developed by a  physiotherapist. This consisted of isometric and isotonic programmes for quadriceps muscles, performed five times per week. All participants performed 10 repetitions per day for six weeks. All participants provided with an exercise sheet, outlining the programme.  **B**: exercise group Structured home rehabilitation programme only. This structured home-rehabilitation programme consisted of a strengthening component, consisting of a 6- stage progression of 2-leg eccentric drop squats, then single leg lunges, and finally 1-leg eccentric drop  squats. The stretching component of the rehabilitation programme consisted of seated spinal rotations, supine hip external rotation, standing quadriceps stretch, and sitting hamstring stretch. Stretches were performed daily prior to and after the strengthening component of the programme. Each  stretch was performed passively 3 times, with each stretch held for 30 seconds | End-point of follow-up:  **A**: 6 weeks  **B**: 3, 6 and 12 weeks  For how many participants were no complete outcome data available?  (intervention/control)  **A:** Results section (page 102): "all patients completed the regular exercise program", therefore none appeared lost to follow-up  **B**: Separate participant flow not provided for individual groups. Thus group allocation of the 21 withdrawals and 2 cross-overs excluded from the analyses. | Outcome measure-1: pain  Pain during activity 0-10  Knee sleeve:  Effect measure: mean difference [95% CI]:  **A**: -0.5 (-1.6, 0.6), 6 weeks  **B**: -0.1 (-1.58, 1.38), 12 weeks  Pooled effect (fixed effects model), inclusive Miller 1997:  -0.48 [95% CI -1.31 to 0.35] favoring sleeve  Heterogeneity (I^2^): 0%  Knee brace:  Effect measure: mean difference [95% CI]:  **B**: -0.2 (-1.68, 1.28), 12 weeks  All braces: Pooled effect (fixed effects model), inclusive Miller 1997:  -0.46 [95% CI -1.16 to 0.24] favoring brace  Heterogeneity (I^2^): 0%  All braces, B included with number of knee instead of number of patients A: -0.5 (-1.6, 0.6), 6 weeks  Ba: -0.2 (-1.43, 1.03), 12 weeks Bb: -0.1 (-1.33, 1.13), 12 weeks Pooled effect (fixed effects model), inclusive Miller 1997:  -0.41 [95% CI -1.04 to 0.23] favoring brace  Heterogeneity (I^2^): 0%  Outcome measure-2, function functional scores  Effect measure: std. mean difference [95% CI]:  A: -0.08 (-0.5, 0.34), womac  Ba: -0.35 (-0.95, 0.24), knee function scale Bb: -0.47 (-1.07, 0.13), knee function scale Pooled effect (fixed effects model):  -0.25 [95% CI -0.55 to 0.05] favoring brace  Heterogeneity (I^2^): 0%  functional scores, B included with number of knee instead of number of patients  Effect measure: std. mean difference [95% CI]:  A: -0.08 (-0.5, 0.34), womac  Ba: -0.35 (-0.85, 0.14), knee function scale Bb: -0.47 (-0.97, 0.05), knee function scale Pooled effect (fixed effects model):  -0.28 [95% CI -0.55 to -0.01] favoring brace  Heterogeneity (I^2^): 0% | Facultative:  Brief description of author’s conclusion: very low  quality evidence from clinically heterogeneous trials using different types of knee orthoses (knee brace, sleeve and strap) that using a  knee orthosis did not reduce knee pain or improve knee function in the short term (under three months) in adults who were also undergoing an exercise programme for treating PFPS  Risk of bias: regarding allocation, blinding, incomplete outcome data etc.  GRADE regarding VAS: very low (two levels risk of bias, one level downgraded indirectness)  GRADE regarding functional outcome: very low (two levels risk of bias, one level downgraded imprecision) |
| Insoles |  |  |  |  |  |  |  |
| Hossain, 2011 | SR and meta-analysis of RCTs  *Literature search up to March 2010*  **A**: Collins, 2008  **B**: Wiener-Ogilvie, 2004  Study design:  A: RCT, parallel B: RCT, parallel  Setting and Country: A: single center within a community setting, Brisbane, Australia  B: single center trial in Winshaw, Lanarkshire, UK  Source of funding and conflicts of interest:  SR: Betsy Cadwaladr University health Board, Bangor, UK; University of Oxford, UK; University hospitals of Birmingham, UK; no conflicts of interest  A: not reported  B: not reported | Inclusion criteria SR: - randomised or quasi-randomised studies - skeletally mature adults )aged 18 years or over) - at least one intervention arm where foot orthoses were compared with not treatment, placebo, analgesia or another orthotic device. Extended to included comparisons of foot orthoses plus another physical therapy intervention versus the same physical therapy intervention on its own and comparisons of foot orthoses versus another physical therapy intervention. - outcome: knee pain as quantified by VAS or other method; knee function used knee function score (e.g. WOMAC score, IKSS score); patient satisfaction; patient reported quality of life; use of analgesics/non-steroidal nati-inflammatory drugs; adverse events  Exclusion criteria SR: - adolescents  *3 studies included (2 relevant for current review)*  Important patient characteristics at baseline:  N  **A**: I: 44   C: 45  **B**: I: 10  C: 10  age  **A**: not reported per group  **B**: I: 61.8 years  C: 51 years  *Sex:*  **A**: not reported per group  **B**: not reported per group  Groups comparable at baseline? *not reported* | Describe intervention:  **A**: foot orthoses + physiotherapy: participants received both orthoses and physiotherapy and had an extra  appointment with the physiotherapist if necessary.  orthoses: received prefabricated, commercially available orthoses fitted to their shoes and customised,  if necessary, to optimise comfort through heat moulding and by adding wedge or heel raises.  The participants were advised to continue exercise and activities that did not provoke their pain.  The foot orthoses group were prescribed additional home exercise programme. Whereas this was prescribed to be performed bilaterally twice daily.  **B**: foot orthoses + exercises  Patients received supervised treatment over a 4 week period.  Patients receiving foot orthoses  were assessed by the podiatrist once a week for 3 weeks to check for the fitness of the orthoses. Patients were advised to continue to wear the orthoses and perform exercises on their own  accord after 4 weeks.  Foot orthoses group were provided with foot orthoses with a 40° rearfoot post. Rearfoot posts and forefoot posts were adjusted using 20° or 40° additional wedges, if necessary. | Describe control:  **A**: physiotherapy physiotherapy consisted of patellar mobilisation, patellar taping, a progressive programme  of vasti muscle retraining exercises with electromyographic biofeedback, hamstring and anterior hip  stretches, hip external rotator retraining, and a home exercise programme.  The participants were advised to continue exercise and activities that did not provoke their pain.  **B**: exercises Patients received supervised treatment over a 4 week period. Patients receiving the knee exercise regimen were seen twice a week for two weeks and then once a  week for a further two weeks by a physiotherapist (6 sessions in total).  Patients were advised to continue to perform exercises on their own  accord after 4 weeks.  exercise therapy:  • Isometric quadriceps contractions in full knee and hip extension.  • Isometric quadriceps contractions with hip slightly flexed and knee in full extension.  • Isotonic quadriceps contractions without resistance.  • Isotonic quadriceps contractions with resistance.  • Isotonic hamstring contractions.  • Dynamic stepping exercise.  • Hamstrings stretching exercises.  • Isometric hip adductors contractions.  • Dynamic side stepping. | End-point of follow-up:  **A**: 6, 12 and 52 weeks  **B**: 8 weeks  For how many participants were no complete outcome data available?  (intervention/control)  **A:** I: n=1, lost to follow-up C: n=3, lost to follow-up  **B**: I: n=1, drop out  C: n=1, drop out due to injury | Outcome measure-1: pain  VAS (worst pain)  Effect measure: mean difference [95% CI]:  **A**: -3.7 (-12.99, 5.59) (6 weeks)  **A**: -3.4 (-13.52. 6.75) (52 weeks)  No pooled effect calculated  F-36 pain scale (change scores at 8 weeks, positive scores = pain reduction)  Effect measure: mean difference [95% CI]:  **B**: 9.6 (-8.99, 28.19)  Knee pain numbers with global improvement)  Effect measure: RR [95% CI]:  **8 weeks A**: 0.98 (0.86 – 1.11)  **B**: 1.33 (0.41, 4.33)  Pooled effect (fixed effects model):  1 [95% CI 0.86 to 1.17]  Heterogeneity (I^2^): 0%  **52 weeks A**: 1.01 (0.82 – 1.23)  Outcome measure-2: function  Functional index questionnaire (FIQ)  Effect measure: mean difference [95% CI]:  **A**: 0.4 (-0.59, 1.39) (6 weeks)  A: -0.4 (-1.51, 0.71) (52 weeks)  No pooled effect calculated  Knee function: anterior knee pain (Kujala anterior knee pain scale) Effect measure: mean difference [95% CI]:  **A**: 0.2 (-3.72, 4.12) (6 weeks)  **A**: 3.6 (-0.52, 7.72) (52 weeks)  No pooled effect calculated | Facultative:  Brief description of author’s conclusion: Though limited, the  available evidence does not reveal any clear advantage of foot orthoses over simple insoles or physiotherapy for PFJ pain  Included studies show risk of bias due to issues regarding blinding, selective reporting, similarity at baseline and equal treatment of different groups |

Table 2.6 Evidence table for systematic review of RCTs and observational studies (intervention studies)

| **Study**  **First author, year** | **Appropriate and clearly focused question?^1^**  **Yes/no/unclear** | **Comprehensive and systematic literature search?^2^**  **Yes/no/unclear** | **Description of included and excluded studies?^3^**  **Yes/no/unclear** | **Description of relevant characteristics of included studies?^4^**  **Yes/no/unclear** | **Appropriate adjustment for potential confounders in observational studies?^5^**  **Yes/no/unclear/notapplicable** | **Assessment of scientific quality of included studies?^6^**  **Yes/no/unclear** | **Enough similarities between studies to make combining them reasonable?^7^**  **Yes/no/unclear** | **Potential risk of publication bias taken into account?^8^**  **Yes/no/unclear** | **Potential conflicts of interest reported?^9^**  **Yes/no/unclear** |
| --- | --- | --- | --- | --- | --- | --- | --- | --- | --- |
| Taping |  |  |  |  |  |  |  |  |  |
| Callaghan, 2012 | Yes | Yes | Yes | Yes | Not applicable | Yes | Yes | No | No |
| Bracing |  |  |  |  |  |  |  |  |  |
| Smith, 2015 | Yes | Yes | Yes | Yes | Not applicable | Yes | Yes | Yes | No |
| Insoles |  |  |  |  |  |  |  |  |  |
| Hossain, 2011 | Yes | Yes | Yes | Yes | Not applicable | Yes | Yes | Yes | No |

Table 2.7 Quality assessment for systematic reviews of RCTs and observational studies

Based on AMSTAR checklist (Shea et al.; 2007, BMC Methodol 7: 10; doi:10.1186/1471-2288-7-10) and PRISMA checklist (Moher et al 2009, PLoS Med 6: e1000097; doi:10.1371/journal.pmed1000097)

1. Research question (PICO) and inclusion criteria should be appropriate and predefined
2. Search period and strategy should be described; at least Medline searched; for pharmacological questions at least Medline + EMBASE searched
3. Potentially relevant studies that are excluded at final selection (after reading the full text) should be referenced with reasons
4. Characteristics of individual studies relevant to research question (PICO), including potential confounders, should be reported
5. Results should be adequately controlled for potential confounders by multivariate analysis (not applicable for RCTs)
6. Quality of individual studies should be assessed using a quality scoring tool or checklist (Jadad score, Newcastle-Ottawa scale, risk of bias table etc.)
7. Clinical and statistical heterogeneity should be assessed; clinical: enough similarities in patient characteristics, intervention and definition of outcome measure to allow pooling? For pooled data: assessment of statistical heterogeneity using appropriate statistical tests (e.g. Chi-square, I^2^)?
8. An assessment of publication bias should include a combination of graphical aids (e.g., funnel plot, other available tests) and/or statistical tests (e.g., Egger regression test, Hedges-Olken). Note: If no test values or funnel plot included, score “no”. Score “yes” if mentions that publication bias could not be assessed because there were fewer than 10 included studies.
9. Sources of support (including commercial co-authorship) should be reported in both the systematic review and the included studies. Note: To get a “yes,” source of funding or support must be indicated for the systematic review AND for each of the included studies.

| **Study reference**  (first author, publication year) | **Describe method of randomisation^1^** | **Bias due to inadequate concealment of allocation?^2^**  (unlikely/likely/unclear) | **Bias due to inadequate blinding of participants to treatment allocation?^3^**  (unlikely/likely/unclear) | **Bias due to inadequate blinding of care providers to treatment allocation?^3^**  (unlikely/likely/unclear) | **Bias due to inadequate blinding of outcome assessors to treatment allocation?^3^**  (unlikely/likely/unclear) | **Bias due to selective outcome reporting on basis of the results?^4^**  (unlikely/likely/unclear) | **Bias due to loss to follow-up?^5^**  (unlikely/likely/unclear) | **Bias due to violation of**  **intention to treat analysis?^6^**  (unlikely/likely/unclear) |
| --- | --- | --- | --- | --- | --- | --- | --- | --- |
| Taping |  |  |  |  |  |  |  |  |
| Arrebola, 2020 | Sealed envelops | Unlikely | Likely | Likely | Unlikely | Unlikely | Likely | Unclear |
| Begum, 2020 | Not reported | Unclear | Likely | Likely | Unlikely | Unlikely | Unclear | Likely |
| Ghourbanpour, 2018 | Not reported | Unclear | Likely | Likely | Unclear | Unlikely | Unlikely | Unclear |
| Gunay, 2017 | Computerized random numbers | Unclear | Likely | Likely | Unclear | Unlikely | Unclear | Unclear |
| Akbas, 2011 | Random number generator | Unclear | Likely | Likely | Unclear | Unlikely | Unclear | Unclear |
| Bracing |  |  |  |  |  |  |  |  |
| Alsaharani, 2019 | Simple randomization | Unclear | Likely | Likely | Unclear | Unlikely | Unclear | Likely |
| Uboldi, 2018 | Not reported | Unclear | Likely | Likely | Unclear | Likely | Unlikely | Likely |
| Petersen, 2016 | Not reported | Unclear | Likely | Likely | Unclear | Unlikely | Unlikely | Unclear |

Table 2.8 Risk of bias table for intervention studies (randomized controlled trials)

Randomisation: generation of allocation sequences have to be unpredictable, for example computer generated random-numbers or drawing lots or envelopes. Examples of inadequate procedures are generation of allocation sequences by alternation, according to case record number, date of birth or date of admission.

1. Allocation concealment: refers to the protection (blinding) of the randomisation process. Concealment of allocation sequences is adequate if patients and enrolling investigators cannot foresee assignment, for example central randomisation (performed at a site remote from trial location) or sequentially numbered, sealed, opaque envelopes. Inadequate procedures are all procedures based on inadequate randomisation procedures or open allocation schedules..
2. Blinding: neither the patient nor the care provider (attending physician) knows which patient is getting the special treatment. Blinding is sometimes impossible, for example when comparing surgical with non-surgical treatments. The outcome assessor records the study results. Blinding of those assessing outcomes prevents that the knowledge of patient assignement influences the proces of outcome assessment (detection or information bias). If a study has hard (objective) outcome measures, like death, blinding of outcome assessment is not necessary. If a study has “soft” (subjective) outcome measures, like the assessment of an X-ray, blinding of outcome assessment is necessary.
3. Results of all predefined outcome measures should be reported; if the protocol is available, then outcomes in the protocol and published report can be compared; if not, then outcomes listed in the methods section of an article can be compared with those whose results are reported.
4. If the percentage of patients lost to follow-up is large, or differs between treatment groups, or the reasons for loss to follow-up differ between treatment groups, bias is likely. If the number of patients lost to follow-up, or the reasons why, are not reported, the risk of bias is unclear
5. Participants included in the analysis are exactly those who were randomized into the trial. If the numbers randomized into each intervention group are not clearly reported, the risk of bias is unclear; an ITT analysis implies that (a) participants are kept in the intervention groups to which they were randomized, regardless of the intervention they actually received, (b) outcome data are measured on all participants, and (c) all randomized participants are included in the analysis.

Summary of literature

#

# Taping

Summary of literature

Calaghan (2012) is a Cochrane review that included meta-analyses of the effect of knee-taping and exercises (n=50; age and gender not reported) compared with exercises (mostly stretching and strengthening lower limbs) alone (n=50; age and gender not reported) on pain and function in patients with PFP. Three included RCTs were relevant for the current literature review. Different taping techniques (see evidence table) were used in each study. In Clark (2000), both groups also received education and in Tunay (2003), both groups also received treatment with ice in addition to the described interventions above. In Wittingham (2004) groups did not receive additional interventions besides taping and exercises, but a placebo taping with exercises group besides the exercise only control group was included as well. Meta-analyses were performed for outcome measures at the end of treatment (three weeks to three months).

Arrebola (2020) studied the effect of two KT® taping techniques (see evidence table) in combination with exercises (technique 1: n=13, age (SD)=30.4(8.4) years; technique 2: n=14, age (SD)=27.9 (9.4) years) compared to exercises (hip and quadriceps strengthening) alone (n=16, age (SD)=30.3 (7.9)) on pain and function in females with PFP. The treatment period was 12 weeks.

Begum (2020) studied the effect of the McConnel taping technique in combination with exercises (n=16) compared to exercises (strengthening m. vastus medialis) alone (n=25) on pain and function in patients with PFP. Patients were 36.0 (SD=7.4) years old. The treatment period was two weeks.

Ghourbanpour (2018) studied the effect of the McConnell taping technique in combination with exercises (n=15, age (SD)=33.9(10.3) years, gender not reported) compared to exercises (strengthening m. vastus medialis oblique, stretching hamstring muscles and iliotibial band and patellar mobilization) alone (n=15, age (SD)=37.2(12.4) years, gender not reported) on pain and function in patients with anterior knee pain. The treatment period was four weeks.

Gunay (2017) studied the effect of kinesio taping in combination with exercises (n=25 knees age (SD)=36.0(8.0) years, 31.3% male) compared to sham taping with exercises (n=25 knees, age (SD)=31.7(8.5) years, 50% male) and no exercises (strengthening quadriceps, m. gluteus medius, stretching quadriceps, hamstring and gastrocnemius muscles and iliotibial band) alone (n=25 knees, age (SD)=33.8(6.7) years, 61.5% male) on pain and function in patients with PFP. The treatment period was six weeks.

Akbas (2011) investigated the effect of kinesio taping in combination with exercises (n=15, age (SD)=41.0(11.3) years) compared to exercises (muscle strengthening and soft tissue stretching) alone (n=16, age (SD)=44.9(7.8) years) on pain in female patients with PFP. The treatment period was six weeks.

# Results

Pain

Callaghan (2012) performed a meta-analysis of pain VAS score after treatment period. As all additional selected RCTs reported pain VAS scores and numerical pain rating scores (NPRS), we extended this meta-analysis to evaluate the effect of taping in addition to exercises on pain-scores after the treatment period. Gunay (2017) could not be included in meta-analysis as only median(range)-scores (I: VAS (range)=30(0-50), n=25 knees; C: VAS (range) = (20(0-40), n=25 knees) values were reported. In both the meta-analysis (Figure 2.1) and Gunay (2017), for post-intervention measures, no statistically significant or clinically relevant effect of taping as an addition to exercise therapy (MD (95%CI): -0.75(-1.57, 0.08), favoring tape)) on pain was found.


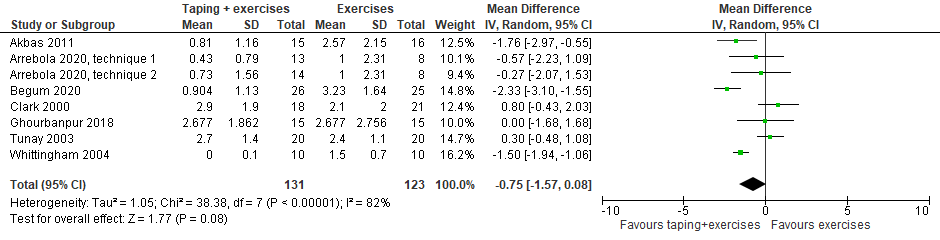


Figure 2.1 Pooled results taping on pain short-term

Three studies evaluated the results on the long-term. No statistically significant or clinically relevant differences in pain scores between groups were found. Due to heterogeneity in follow-up periods (see table 2.8) and limited reporting (eg. Gunay, 2017), data could not be pooled.

|  | MD (95%CI) negative value: favoring tape | n | Follow-up, after ending treatment |
| --- | --- | --- | --- |
| Arrebola 2020, technique 1, NPRS(1-10) | -0.70 (-2.09, 0.69) | I: 13 C: 16 | 6 weeks |
| Clark 2000,  VAS (0-10) | -0.13 (-0.96, 3.52) | I: 10 C: 12 | 9 months |
| Gunay 2017,  VAS (0-100) | -10 (not reported) | I: 25 knees C: 25 knees | 6 weeks |

Table 2.9 Pain scores after taping and follow-up period

Level of evidence of the literature

The level of evidence of RCTs starts at high, but was downgraded to low because of study limitations (1 level, risk of bias, see risk of bias tables) and low number of participants (1 level, imprecision).

Function

Several studies (Akbas, 2011; Arrebola, 2020; Gunay, 2017) evaluated the additional effect of taping to an exercise program on function using the Kujala Anterior Knee Pain Scale (see evidence tables). A meta-analysis was performed (Figure 2.2). Gunay (2017) could not be included in meta-analysis as only median(range)-scores were reported (I: Kujala median(range)=87 (74-100), n=25 knees; C: Kujala median (range) =87(82-94)), n=25 knees, difference were not statistically significant nor clinically relevant). Taping in combination with exercises did not result in statistically significant or clinically relevant improved function after six weeks intervention when compared to exercises alone, MD (95%CI): 0.69 (-3.82, 5.20).


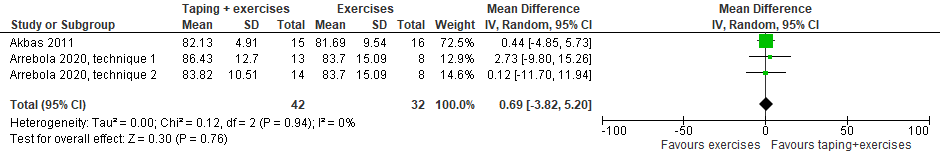


Figure 2.2 Pooled results taping on function short-term

After a follow-up period, two studies, investigated the difference in function after treatment with tape and exercises compared to exercises alone as well. Due to not reporting mean (SD) values (e.g. Gunay, 2017), data could not be pooled. Conflicting results regarding the effect of taping as an addition to exercise therapy were reported.

Level of evidence of the literature

Function short term (after six weeks intervention)

The level of evidence of RCTs starts at high, but was downgraded to low because of study limitations (1 level, risk of bias, see risk of bias tables) and low number of participants (1 level, imprecision).

Function follow-up six weeks

The level of evidence of RCTs starts at high, but was downgraded to very low because of study limitations (1 level, risk of bias), conflicting study results (1 level, inconsistency) and low number of participants (1 level, imprecision).

Return to sport/work

Return to sport/work was not described as an outcome in the included studies.

Level of evidence of the literature

The level of evidence regarding the outcome return to sport/work was not assessed due to lack of studies.

Patient satisfaction

Patient satisfaction was not described as an outcome in the included studies.

Level of evidence of the literature

The level of evidence regarding the outcome patient satisfaction was not assessed due to lack of studies.

Patient recovery

Patient recovery was not described as an outcome in the included studies.

Level of evidence of the literature

The level of evidence regarding the outcome patient recovery was not assessed due to lack of studies.

# Conclusions

| **low GRADE** | The addition of taping to an exercise program may have no effect on pain in patients with PFP.  *Sources: Akbas, 2011; Arrebola, 2020; Callaghan, 2012; Begum, 2020; Ghourbanpour, 2018; Gunay, 2017* |
| --- | --- |

| **low GRADE** | The addition of taping to an exercise program may have no effect on function in patients with PFP after six weeks intervention.  *Sources: Akbas, 2011; Arrebola, 2020; Gunay, 2017* |
| --- | --- |

| **Very low GRADE** | The effect of the addition of taping to an exercise program on function in patients with PFP during follow-up (six weeks post-intervention) is unclear.  *Sources: Akbas, 2011; Arrebola, 2020* |
| --- | --- |

| **- GRADE** | The outcome measures return to work/sport, patient satisfaction and recovery in patients with PFP were not reported in the included studies.  Source: - |
| --- | --- |

# Braces/knee orthosis

Summary of literature

Smith (2015) is a Cochrane review which included meta-analyses evaluating the effect of treatment of knee orthosis (sleeve and Special FX knee brace) and exercises compared with exercises (mostly stretching and strengthening lower limbs) alone on pain and function in patients with PFP. Two RCTs were relevant for the current literature review (Lun, 2005; Evcik, 2010). Together, these studies included 104 patients in three intervention groups (mean age (SD)respectively: 42.2 (15.3) years, 35 (11) years and 35 (9) years; % male/female not reported) and 79 patients in two control groups(mean ages (SD) : 41.0(9.3) years, 35(11); % male/female not reported). Based on follow-up periods, treatment duration ranged from 6-12 weeks.

Alsharani (2019) studied the effect of a knee brace (brace set to resist knee flexion) in combination with exercises (n=21, age (SD)=30.8(5.6) years, 42.9% male) compared to exercises alone (specific exercises that are part of the protonic therapy program; for control group, the brace was replaced with a sport cord; n=20, age (SD)=26.7(3.0) years, 60% male) on pain and function in patients with PFP. The treatment period was four weeks.

Uboldi (2018) studied the effect of a knee brace (Reaction Knee Brace; DJO Global, Vista, California, United states) in combination with exercises compared to exercises (rehabilitation protocol) alone on pain and function in patients with PFP. 60 patients (30 in each group) were analysed and had a mean age (SD) of 20(4) years. A total of 22% was male. The treatment period was not defined.

Petersen (2016) studied the effect of a knee brace (Patella Pro) in combination with exercises (n=78, age (SD)=28.0(9.4) years, 34.2% male) compared to exercises alone (home based exercise program; n=78, age (SD)=28.0(8.1) years, 21.1% male) on pain and function in patients with anterior knee pain. The treatment period was six weeks.

# Results

Pain

Smith (2015) performed a meta-analysis of reported VAS pain scores. As two additional RCTs reported pain VAS scores and numerical pain rating scores (NPRS) as well, this meta-analysis was extended to investigate the effect of bracing in addition to exercises on pain-scores after the treatment period (see figure 3). No significant and clinically relevant effect of bracing as an addition to exercise therapy (MD (95%CI): 0.33 (-1.10, 1.77), favoring exercises alone)) on pain was found when treatment period was finished.

Petersen (2016) could not be included in the meta-analysis as results were only reported in figures and narrative text. In this study, inconsistent results were noted for pain after six and twelve weeks wearing the brace. During climbing stairs or playing sports, but not during walking and rest, a significant decrease in pain for the group wearing the brace compared to the control group was found.

Uboldi (2018) and Petersen (2016) reported pain scores after a follow-up period. Peterson (2016) reported no significant differences in pain scores between groups after 1 year starting treatment (values not reported). However, Uboldi did report statistically significant differences in pain scores after six (MD (95%CI): -1.60 (-2.38, -0.82)) and twelve months (MD (95%CI): -0.9 (1.69, -0.11)) in favour of bracing. However, these differences are deemed not clinically relevant. Results of the meta-analysis are presented in Figure 2.3.


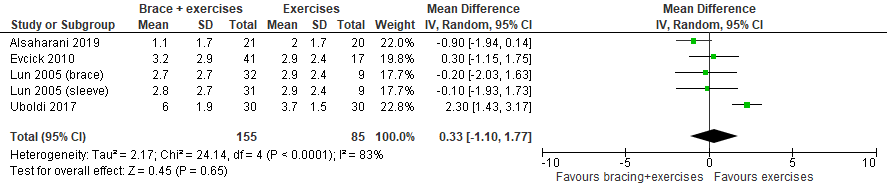


Figure 2.3 Pooled results brace on pain short-term

Level of evidence of the literature

Pain

The level of evidence of RCTs starts at high, but was downgraded three levels to very low because of study limitations (2 level, risk of bias, see risk of bias) and low number of participants (1 level, imprecision).

Function

Smith (2015) performed a meta-analysis of reported functional scores using the post-intervention scores. One study used the Kujala Knee Pain Score (Lun, 2005). As the additional RCTs reported post-intervention knee function scores as well, we extended this meta-analysis. Petersen (2016) could not be included as the results were only reported in figures and narrative text. They found significantly improved Kujala scores after six and twelve weeks for the group receiving a brace in addition to exercises when compared to the scores of the group receiving only exercises.

In the meta-analysis, no statistically significant nor clinically relevant effect of bracing in addition to exercise therapy was found on function (MD (95%CI): -0.42(-3.68, 2.83), favouring exercises alone) (Figure 2.4).


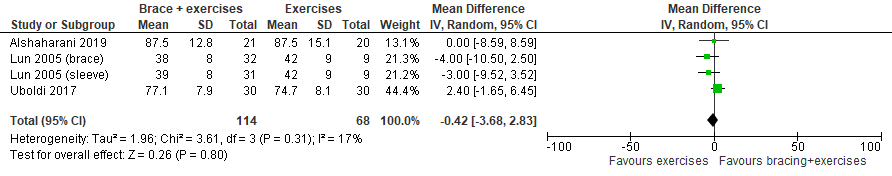


Figure 2.4 Pooled results of brace on function short-term

On the long term, only Uboldi (2018) investigated the added value of a brace to exercise therapy regarding function with the Kujala scale. No statistically significant and clinically relevant effect was found at 6 months (MD (95%CI): 2.90 (-1.05, 6.85)) and 12 months (MD (95%CI: 2.50 (-1.50, 6.50)).

Level of evidence of the literature

Function

The level of evidence of RCTs starts at high, but was downgraded to very low because of study limitations (2 levels, risk of bias, see risk of bias tables) and low number of participants (1 level, imprecision).

Return to sport/work

Return to sport/work was not described as an outcome in the included studies.

Level of evidence of the literature

The level of evidence regarding the outcome return to sport/work was not assessed due to lack of studies.

Patient satisfaction

Patient satisfaction was not described as an outcome in the included studies.

Level of evidence of the literature

The level of evidence regarding the outcome patient satisfaction was not assessed due to lack of studies.

Patient recovery

Petersen (2016) reported no statistically significant difference between groups in proportions of patients reporting recovery. As values were not reported, clinical relevance could not be determined.

Level of evidence of the literature

The level of evidence of RCTs starts at high, but was downgraded very low because of study limitations (1 level, risk of bias, see risk of bias tables) and low number of studies and not reporting confidence intervals (2 levels, imprecision).

# Conclusions

| **very low GRADE** | It is unclear whether the addition of bracing to an exercise program has an effect on pain in patients with PFP.  *Sources: Alsharani, 2019; Petersen, 2016; Smith, 2015; Uboldi, 2017* |
| --- | --- |

| **very low GRADE** | It is unclear whether the addition of bracing to an exercise program has an effect on function in patients with PFP.  *Sources: Alsharani, 2019; Petersen, 2016; Smith, 2015; Uboldi, 2018* |
| --- | --- |

| **- GRADE** | The outcome measures return to work/sport and patient satisfaction in patients with PFP were not reported in the included studies.  Source: - |
| --- | --- |

| **very low GRADE** | It is unclear whether the addition of bracing to an exercise program has an effect on patient recovery in patients with PFP.  *Sources: Petersen, 2016* |
| --- | --- |

#

# Foot orthosis

Summary of literature

Hossain (2011) is a Cochrane review investigating the effect of foot orthosis and exercises (n=54) compared with exercises alone (n=55) on pain and function in patients with PFP. Patient characteristics were not reported per group. Duration of treatment was not for all studies clearly described. Based on follow-up periods, treatment duration ranged from 4-8 weeks, but treatment could be continued unsupervised.

# Results

Pain

Hossain (2011) reported effect of treatment on VAS in one RCT (Collins, 2008) (Table 2.9). Adding foot orthosis did not have a statistically significant or clinically relevant effect on pain in patients with PFP.

|  | MD (95%CI) | n |
| --- | --- | --- |
| Collins 2008,  6 weeks VAS (0-100) | -3.7 (-12.99, 5.59) | I: 42 C: 41 |
| Collins 2008,  52 weeks VAS (0-100) | -3.4 (-13.52, 6.72) | I: 43 C: 42 |

Table 2.9 Pain scores after treatment with foot orthoses

Level of evidence of the literature

The level of evidence of RCTs starts at high, but was downgraded to very low because of study limitations (1 level, risk of bias, Hossain, 2011) and low number of participants (2 levels, imprecision).

Function

Hossain (2011) reported effect of treatment the Kujala anterior knee pain scale. Foot orthosis did not have a statistically significant or clinically relevant additional effect to exercises on pain in patients with PFP (Table 2.10).

|  | MD (95%CI) positive value favors orthosis | N |
| --- | --- | --- |
| Collins 2008,  6 weeks Kujala Anterior Knee Pain (0-100) | 0.2 (-3.72, 4.12) | I: 42 C: 41 |
| Collins 2008,  52 weeks Kujala Anterior Knee Pain (0-100) | 3.6 (-0.52, 7.72) | I: 43 C: 42 |

Table 2.10 Function scores after treatment with foot orthoses

Level of evidence of the literature

The level of evidence of RCTs starts at high, but was downgraded to very low because of study limitations (1 level, risk of bias, see risk of bias tables and Hossain, 2011) and low number of participants (2 levels, imprecision).

Return to sport/work

Return to sport/work was not described as an outcome in the included studies.

Level of evidence of the literature

The level of evidence regarding the outcome return to sport/work was not assessed due to lack of studies.

Patient satisfaction

Patient satisfaction was not described as an outcome in the included studies.

Level of evidence of the literature

The level of evidence regarding the outcome patient satisfaction was not assessed due to lack of studies.

Patient recovery

Patient recovery was not described as an outcome in the included studies.

Level of evidence of the literature

The level of evidence regarding the outcome patient recovery was not assessed due to lack of studies.

# Conclusions

| **very low GRADE** | It is unclear whether the addition of foot orthoses to an exercise program has an effect on pain in patients with PFP.  *Sources: Hossain, 2011* |
| --- | --- |

| **very low GRADE** | It is unclear whether the addition of foot orthoses to an exercise program has an effect on function in patients with PFP.  *Sources: Hossain, 2011* |
| --- | --- |

| **- GRADE** | The outcome measures return to work/sport, patient satisfaction and recovery in patients with PFP were not reported in the included studies.  Source: - |
| --- | --- |

# Considerations – from evidence to recommendation

Pros and cons of intervention and quality of evidence

Taping

Based on the literature summary, treatment with tape, in addition to exercise therapy, does not appear to have a clinically relevant effect on the crucial outcome measures pain and function. The effect on function after six weeks is unclear. Important outcome measures return to sport/work, satisfaction and recovery cannot further contribute to the direction of decision-making. Regarding these outcome measures, no literature was included in the abstract.

Knee braces

Based on the literature summary, it is unclear whether treatment with a knee brace, in addition to exercise therapy, has an effect on the crucial outcome measures pain and function because of the very low strength of evidence. Mainly caused by limitations in the design of the studies and small numbers of patients. Important outcome measures return to sport/work, satisfaction and recovery cannot further contribute to the direction of decision-making. Regarding these outcome measures, no literature was included in the abstract.

Foot orthoses

Based on the literature summary, it is unclear whether treatment with foot orthoses, in addition to exercise therapy, has an effect on the crucial outcome measures pain and function due to the very low strength of evidence. This very low strength of evidence is mainly caused by limitations in the design of the studies and small numbers of patients. Important outcome measures return to sport/work, satisfaction and recovery cannot further contribute to the direction of decision-making. Regarding these outcome measures, no literature was included in the abstract.

Due to a lack of evidence and conflicting results, regular use of tape, foot orthoses or bracing cannot be recommended. The expert panel advocates taping and/or foot orthoses in combination with exercise therapy to achieve short-term effects in individual cases. For the use of tape, braces and foot orthoses, the added value in addition to exercise therapy for the treatment of pain is unclear. The quality of evidence is (very) low mainly due to limitations in the design of the studies and small patient numbers. We found no side effects or complications in the literature for the use of tape, foot orthoses or braces compared to control groups.

The ‘Patellofemoral pain clinical practice guideline” (Willy, 2019), advises a personal intervention for the use of tape and/or foot orthoses when 1) there is a static and / or 2) dynamic medialization of the foot or large joint mobility of the foot, in combination with exercise therapy in short term. We didn’t find this in our study because the emerged literature is based on large groups of patients and not selected on individual characteristics in biomechanical variables. It is expected that personalization of treatment based on biomechanical variables may lead to better clinical results. For example foot orthoses that support the arch of the foot and tape which influences the patellar movement.

A widely cited article, Collins, 2008, on the complementary effects of foot orthoses to exercise therapy in PFP finds a significant difference after 6 weeks between 'foot orthoses' (custom-made) and 'flat inserts' (ready-made) only on the 'recovery' domain. Foot orthoses scored better than flat inserts. On the pain domain, no significant differences were found at any measurement point between the different interventions (foot orthoses, flat inserts and physical therapy). Although early results for global improvement favored the foot orthoses, this effect was no longer apparent after 12 weeks and 52 weeks.

In clinical practice, the application of tape around the kneecap in particular is seen as a method to directly reduce pain and improve function. The fitting of foot orthoses is also done regularly in practice. The expert panel realizes that this application occurs in the clinical setting, although there is a lack of evidence. With taping, it cannot be ruled out that observed improvements are based on a placebo effect.

In the study by Akbas, 2011, the patients have an average high(er) age; the expert panel believes that this study may not be about PFP. Since little difference emerges from the study, the effects are ultimately negligible on the outcomes for this guideline.

We have focused on the AKPS/Kujala score in this guideline, as these are primarily used in the literature to measure pain scores. Other scores (KOOS/WOMAC and Tegner) have not been validated for PFP but have been found in some literature reviews. These have no cut-off point for PFP; no "minimal clinical important difference" for sports or work resumption and do not include satisfaction and recovery.

Uboldi, 2018 reports numbers of returning athletes but does not use a Tegner score. Petersen, 2016 uses the "sports and recreational activities" domain in the KOOS as a score; again, no cutoff values are known and have not been validated for PFP.

Expectations and values of patients (and possibly their caretakers)

To gain insight into the values and expectations of patients with PFP, a questionnaire was prepared and distributed in collaboration with the Netherlands Patients Federation (NPF). The questionnaire was completed by 43 PFP patients (21 who have had PFP and 22 who currently have PFP). 75% of the respondents indicated that they had received additional treatment such as foot orthoses, tape or brace.

The goal of the respondents is to achieve normal function in daily living, as soon as possible resume work and sport as before is also indicated.

50% of the respondents are satisfied with the tape, 10% is unsatisfied. 3 patient used a brace, 1 is satisfied, 1 neutral and 1 unsatisfied. 54% of the respondents is satisfied with the foot orthoses, 18% is unsatisfied.

For the use of tape, foot orthoses and braces, no side effects or complications are found in the literature compared to the control group. The expert panel also believes that these interventions have no adverse side effects.

Cost

There are costs associated with the manufacture of a fitted brace and foot orthoses. There is no economic analysis available on the costs of these devices, nor whether these outweigh the benefits (cost-effectiveness). It is therefore not possible for the expert panel to make a statement on these costs. Globally, it can be stated that the costs of tape and ready-made braces and foot orthoses are low (€20-€50). Fitted braces and foot orthoses are usually more expensive (ranging from €100 to €250). Although costs are relatively low and often partially compensated by insurance, the effect of additional treatments is unclear. From this point of view, the patient should make personal consideratiosn before starting additional treatment(s).

Acceptability, feasibility and implementation

Conservative treatments, such as taping, braces and foot orthoses, are already widely used in clinical practice. Good knowledge of what your co-treaters can and do is essential in good decision-making about the use of additional treatment. This requires an extensive functioning/communicating multidisciplinary network.

Objections that may be raised by the patient probably lie mainly in the costs. Although these are relatively low, not everyone has the right opportunity and finances. The mentioned additional treatments are not yet compensated by the basic insurance. In addition, the compensation vary per insurer and supplementary insurance package, with not always the entire amount is reimbursed, but only partially. Patients would like to be informed about the advantages and disadvantages of conservative treatments.

According to the expert panel, there are enough healthcare providers in the Netherlands to provide individually fitted tape, brace and foot orthoses. However, the distribution of podiatrists in particular is less in some parts of the country, so waiting times may be longer in some regions.

# Rationale of the recommendations

Due to a lack of evidence and partly conflicting outcomes, the structural application of foot orthoses, taping or bracing in PFP cannot be recommended. In individual cases and in combination with exercise therapy, taping and/or foot orthoses can be considered to achieve short-term effects.

#

# References

Akbaş, E. & Atay, A. & Yuksel, I. (2011). The effects of additional kinesio taping over exercise in the treatment of PFP. *Acta orthopaedica et traumatologica turcica*. 45. 335-41. 10.3944/AOTT.2011.2403.

Alshaharani, M. S., Lohman, E. B., Bahjri, K., Harp, T., Alameri, M., Jaber, H., & Daher, N. S. (2019). Comparison of Protonics™ Knee Brace With Sport Cord on Knee Pain and Function in Patients With Patellofemoral Pain Syndrome: A Randomized Controlled Trial, *Journal of Sport Rehabilitation*, 29(5), 547-554.

Arrebola, L. S., de Carvalho, R. T., Wun, P. Y. L., de Oliveira, P. R., dos Santos, J. F., de Oliveira, V. G. C., & Pinfildi, C. E. (2020). Investigation of different application techniques for Kinesio Taping® with an accompanying exercise protocol for improvement of pain and functionality in patients with patellofemoral pain syndrome: A pilot study. Journal of Bodywork and Movement Therapies, 24(1), 47-55.

Begum, R., Tassadaq, N., Ahmed, S., Qazi, W. A., Javed, S., & Murad, S. (2020). Effects of McConnell taping combined with strengthening exercises of vastus medialis oblique in females with patellofemoral pain syndrome. *JPMA. The Journal of the Pakistan Medical Association*, *70*(4), 728-730.

Callaghan, M. J., & Selfe, J. (2012). Patellar taping for patellofemoral pain syndrome in adults. *Cochrane Database of Systematic Reviews*, (4).

Clark, D. I., Downing, N., Mitchell, J., Coulson, L., Syzpryt, E. P., & Doherty, M. (2000). Physiotherapy for anterior knee pain: a randomised controlled trial. *Annals of the rheumatic diseases*, *59*(9), 700-704.

Collins, N., Crossley, K., Beller, E., Darnell, R., McPoil, T., & Vicenzino, B. (2008). Foot orthoses and physiotherapy in the treatment of patellofemoral pain syndrome: randomised clinical trial. *Bmj*, *337*, a1735.

Ghourbanpour, A., Talebi, G. A., Hosseinzadeh, S., Janmohammadi, N., & Taghipour, M. (2018). Effects of patellar taping on knee pain, functional disability, and patellar alignments in patients with patellofemoral pain syndrome: A randomized clinical trial. *Journal of bodywork and movement therapies*, 22(2), 493-497.

Günay, E., Sarıkaya, S., Özdolap, Ş., & Büyükuysal, Ç. (2017). Effectiveness of the kinesiotaping in the patellofemoral pain syndrome. *Turkish Journal of Physical Medicine and Rehabilitation*, 63(4), 299.

Lun, V. M., Wiley, J. P., Meeuwisse, W. H., & Yanagawa, T. L. (2005). Effectiveness of patellar bracing for treatment of patellofemoral pain syndrome. *Clinical Journal of Sport Medicine*, *15*(4), 235-240.

Hossain, M., Alexander, P., Burls, A., & Jobanputra, P. (2011). Foot orthoses for patellofemoral pain in adults. *Cochrane Database of Systematic Reviews*, (1).

Petersen, W., Ellermann, A., Rembitzki, I. V., Scheffler, S., Herbort, M., Brüggemann, G. P., ... & Liebau, C. (2016). Evaluating the potential synergistic benefit of a realignment brace on patients receiving exercise therapy for patellofemoral pain syndrome: a randomized clinical trial. *Archives of orthopaedic and trauma surgery*, *136*(7), 975-982.

Smith, T. O., Drew, B. T., Meek, T. H., & Clark, A. B. (2015). Knee orthoses for treating patellofemoral pain syndrome. *Cochrane Database of Systematic Reviews*, (12).

Tunay V.B. Baltaci G., Tunay S. & Ergun N. (2003) A comparison of different treatment approaches to patellofemoral pain syndrome. *The pain clinic*, 15(2) 179-184.

Uboldi, F. M., Ferrua, P., Tradati, D., Zedde, P., Richards, J., Manunta, A., & Berruto, M. (2018). Use of an elastomeric knee brace in patellofemoral pain syndrome: short-term results. *Joints*, *6*(2), 85.

Willy RW, Hoglund LT, Barton CJ, Bolgla LA, Scalzitti DA, Logerstedt DS, Lynch AD, Snyder-Mackler L, McDonough CM. (2019) Patellofemoral Pain. J Orthop Sports Phys Ther. Sep;49(9):CPG1-CPG95. doi: 10.2519/jospt.2019.0302. PMID: 31475628Whittingham, M., Palmer, S., & Macmillan, F. (2004). Effects of taping on pain and function in patellofemoral pain syndrome: a randomized controlled trial. *Journal of Orthopaedic & Sports Physical Therapy*, *34*(9), 504-510.

# Construction of Module 3 ‘Pain medications for PFP’

#

Scoping Question: What is the value of pain medications, (including injection therapy), for patients with PFP?

# Search and select

A systematic review of the literature was performed to answer the following question: What is the effect of medical treatments, incl. injection therapy, in patients with PFP on pain, function, return to sports/work, duration of absenteeism and patient satisfaction?

P: patients with PFP

I: medical treatment, including injection therapy

C: no treatment/intervention or care as usual (conservative treatment)

O: pain, function, return to sports/ work, duration of absenteeism, patient satisfaction, patient recovery and adverse events.

The outcome measures were defined as following:

- Pain: as determined with the Visual Analogue Scale (VAS 0 - 10) or the Numeric Rating Scale (NRS 0 - 10);
- Function: as determined with the Kujala score (0 - 100)/Anterior Knee Pain Score (AKPS 0 - 100) or the number of patients without symptoms;
- Return to sports/work: as determined with the Tegner activity scale (0 - 10) or with the number of patients who returned to sports/activities;
- Duration of absenteeism; not defined beforehand.
- Patient satisfaction: as determined on a Likert scale;
- Patient recovery: as determined on a Likert scale
- Adverse events: as determined with any adverse events reported in the study.

Relevant outcome measures

The guideline development expert panel considered pain and function as *critical* outcome measures for decision making; and return to sport/work, duration of absenteeism, patient satisfaction, recovery and adverse events as *important* outcome measures for decision making.

The expert panel defined a difference of 2 cm (of 10 cm) on the VAS or 2 categories on the NRS scale as a minimal clinically (patient) important difference, according to Crossley, 2004. For the Kujala score/AKPS score, a difference of 10 point (of 100 points) was defined as a minimal clinically (patient) important difference, according to Crossley, 2004. A minimal clinically important difference for return to sport/work measured with the Tegner score was not predefined, neither for duration of absenteeism, patient satisfaction, patient recovery and adverse events.

Search and select (Methods)

The databases Medline (via OVID) and Embase (via Embase.com) were searched with relevant search terms until 27-10-2020. The detailed search strategy is depicted in tables. The systematic literature search resulted in 159 hits. Studies were selected based on the following criteria: Systematic reviews (searched in at least two databases, and detailed search strategy, risk of bias assessment and results of the individual studies available) and randomized controlled trials (RCTs) that included at least 20 patients with PFP, compared medical treatment with a control group of no treatment or care as usual (conservative treatment), and included at least one of the defined outcome measures. Initially four studies were selected based on title and abstract screening. After reading the full text, three studies were excluded (Table 3.4), and one Cochrane review was included.

Results

One Cochrane review was included in the analysis of the literature. Important study characteristics and results are summarized in the evidence tables. The assessment of the risk of bias is summarized in the risk of bias table.

| **No.** | **Query** | **Results** |
| --- | --- | --- |
| #15 | #12 OR #13 OR #14 | 99 |
| #14 | #8 AND #11 NOT (#12 OR #13) | 29 |
| #13 | #8 AND #10 NOT #12 | 46 |
| #12 | #8 AND #9 | 24 |
| #11 | 'major clinical study'/de OR 'clinical study'/de OR 'case control study'/de OR 'family study'/de OR 'longitudinal study'/de OR 'retrospective study'/de OR 'prospective study'/de OR 'cohort analysis'/de OR cohort*:ab,ti OR (('case control' NEAR/1 (study OR studies)):ab,ti) OR (('follow up' NEAR/1 (study OR studies)):ab,ti) OR (observational NEAR/1 (study OR studies)) OR ((epidemiologic NEAR/1 (study OR studies)):ab,ti) OR (('cross sectional' NEAR/1 (study OR studies)):ab,ti) | 5718113 |
| #10 | 'clinical trial'/exp OR 'randomization'/exp OR 'single blind procedure'/exp OR 'double blind procedure'/exp OR 'crossover procedure'/exp OR 'placebo'/exp OR 'prospective study'/exp OR rct:ab,ti OR random*:ab,ti OR 'single blind':ab,ti OR 'randomised controlled trial':ab,ti OR 'randomized controlled trial'/exp OR placebo*:ab,ti | 3146557 |
| #9 | 'meta analysis'/exp OR 'meta analysis (topic)'/exp OR metaanaly*:ti,ab OR 'meta analy*':ti,ab OR metanaly*:ti,ab OR 'systematic review'/de OR 'cochrane database of systematic reviews'/jt OR prisma:ti,ab OR prospero:ti,ab OR (((systemati* OR scoping OR umbrella OR 'structured literature') NEAR/3 (review* OR overview*)):ti,ab) OR ((systemic* NEAR/1 review*):ti,ab) OR (((systemati* OR literature OR database* OR 'data base*') NEAR/10 search*):ti,ab) OR (((structured OR comprehensive* OR systemic*) NEAR/3 search*):ti,ab) OR (((literature NEAR/3 review*):ti,ab) AND (search*:ti,ab OR database*:ti,ab OR 'data base*':ti,ab)) OR (('data extraction':ti,ab OR 'data source*':ti,ab) AND 'study selection':ti,ab) OR ('search strategy':ti,ab AND 'selection criteria':ti,ab) OR ('data source*':ti,ab AND 'data synthesis':ti,ab) OR medline:ab OR pubmed:ab OR embase:ab OR cochrane:ab OR (((critical OR rapid) NEAR/2 (review* OR overview* OR synthes*)):ti) OR ((((critical* OR rapid*) NEAR/3 (review* OR overview* OR synthes*)):ab) AND (search*:ab OR database*:ab OR 'data base*':ab)) OR metasynthes*:ti,ab OR 'meta synthes*':ti,ab | 677177 |
| #8 | #1 AND (#2 OR #3 OR #4 OR #5 OR #6 OR #7) AND ([english]/lim OR [dutch]/lim) AND [2000-2020]/py NOT ('conference abstract'/it OR 'editorial'/it OR 'letter'/it OR 'note'/it) | 251 |
| #7 | 'steroid'/exp OR steroid*:ti,ab,kw OR 'corticosteroid'/exp OR (((cortex OR cortical OR cortico OR adrenocortical) NEAR/2 (hormone* OR steroid*)):ti,ab,kw) OR corticoid*:ti,ab,kw OR corticosteroid:ti,ab,kw OR cortisone:ti,ab,kw OR adrenocorticosteroid*:ti,ab,kw | 1751292 |
| #6 | 'hyaluronic acid'/exp OR 'hyaluronic acid':ti,ab,kw | 47627 |
| #5 | 'thrombocyte rich plasma'/exp OR 'platelet rich plasma':ti,ab,kw OR 'thrombocyte rich plasma':ti,ab,kw OR prp:ti,ab,kw OR 'autologous conditioned plasma':ti,ab,kw OR acp:ti,ab,kw OR 'autologous blood injection*':ti,ab,kw OR 'prolotherapy'/exp OR 'prolotherapy':ti,ab,kw OR 'proliferation therapy':ti,ab,kw OR 'regenerative injection therapy':ti,ab,kw | 43983 |
| #4 | 'nonsteroid antiinflammatory agent'/exp OR 'lidocaine'/exp OR 'paracetamol'/exp OR ((('non steroid*' OR nonsteroid*) NEAR/2 ('anti inflammatry' OR antiinflammatory) NEAR/2 (drug* OR agent* OR compound*)):ti,ab,kw) OR lidocaine:ti,ab,kw OR paracetamol:ti,ab,kw OR acetaminophen*:ti,ab,kw | 1022032 |
| #3 | 'glycosaminoglycan polysulfate'/exp OR 'glycosaminoglycan polysul*ate':ti,ab,kw | 2188 |
| #2 | 'drug therapy'/exp OR (((drug OR pharma* OR medicament OR medicinal) NEAR/2 (treatment* OR therap*)):ti,ab,kw) OR 'pharmaco therapy':ti,ab,kw OR 'pharmaco treatment':ti,ab,kw OR pharmacotherapy:ti,ab,kw OR pharmacotreatment:ti,ab,kw OR 'injection'/exp OR injection*:ti,ab,kw | 3758395 |
| #1 | 'patellofemoral pain syndrome'/exp OR 'patellofemoral pain':ti,ab,kw OR 'patellofemoral syndrome':ti,ab,kw OR 'patellofemoral dysfunction*':ti,ab,kw OR 'patellofemoral disorder*':ti,ab,kw OR 'pfp*':ti OR 'pfps':ti OR 'retro-patellar pain':ti,ab,kw OR 'retropatellar pain':ti,ab,kw OR 'peri-patellar pain':ti,ab,kw OR 'anterior knee pain':ti,ab,kw OR 'patella chondropath*':ti,ab,kw OR 'patellar chondropath*':ti,ab,kw OR 'patella chrondromalacia*':ti,ab,kw OR 'patellar chrondromalacia*':ti,ab,kw OR 'runner s knee':ti,ab,kw OR 'lateral facet compression syndrome':ti,ab,kw OR 'chondromalacia patella*':ti,ab,kw | 5144 |

Table 3.1 Embase search strategy

| **Search** | **Query** | **Items found** |
| --- | --- | --- |
| 1 | 1 exp "Patellofemoral Pain Syndrome"/ or "Patellofemoral Pain".ti,ab,kf. or 'Anterior Knee Pain'.ti,ab,kf. or 'Patellofemoral Syndrome'.ti,ab,kf. or 'patellofemoral dysfunction*'.ti,ab,kf. or 'patellofemoral disorder*'.ti,ab,kf. or 'PFP'.ti,ab,kf. or 'PFP*'.ti,ab,kf. or 'retro-patellar pain'.ti,ab,kf. or 'retropatellar pain'.ti,ab,kf. or 'peri-patellar pain'.ti,ab,kf. or 'anterior knee pain'.ti,ab,kf. or 'Patella chondropath*'.ti,ab,kf. or 'Patellar chondropath*'.ti,ab,kf. or 'Patella chrondromalacia*'.ti,ab,kf. or 'Patellar chrondromalacia*'.ti,ab,kf. or 'runner* knee'.ti,ab,kf. or 'lateral facet compression syndrome'.ti,ab,kf. or 'chondromalacia patella*'.ti,ab,kf. | 6817 |
| 2 | exp Drug Therapy/ or dt.fs. or ((drug or pharma* or medicament or medicinal) adj2 (treatment* or therap*)).ti,ab,kf. or pharmaco-therapy.ti,ab,kf. or pharmaco-treatment.ti,ab,kf. or pharmacotherapy.ti,ab,kf. or pharmacotreatment.ti,ab,kf. or exp Injections/ or injection*.ti,ab,kf. | 3468665 |
| 3 | Glycosaminoglycans/ or 'glycosaminoglycan polysul*ate'.ti,ab,kf. | 24527 |
| 4 | exp "Anti-Inflammatory Agents, Non-Steroidal"/ or exp Lidocaine/ or exp Acetaminophen/ or (('non steroid*' or nonsteroid*) adj2 ('anti inflammatry' or antiinflammatory) adj2 (drug* or agent* or compound*)).ti,ab,kf. or lidocaine.ti,ab,kf. or paracetamol.ti,ab,kf. or acetaminophen*.ti,ab,kf. | 255368 |
| 5 | exp "Platelet-Rich Plasma"/ or 'platelet rich plasma'.ti,ab,kf. or 'thrombocyte rich plasma'.ti,ab,kf. or prp.ti,ab,kf. or 'autologous conditioned plasma'.ti,ab,kf. or acp.ti,ab,kf. or 'autologous blood injection*'.ti,ab,kf. or exp Prolotherapy/ or 'prolotherapy'.ti,ab,kf. or 'proliferation therapy'.ti,ab,kf. or 'regenerative injection therapy'.ti,ab,kf. | 31509 |
| 6 | exp Hyaluronic Acid/ or 'hyaluronic acid'.ti,ab,kf. | 29767 |
| 7 | exp Steroids/ or steroid*.ti,ab,kf. or exp "Adrenal Cortex Hormones"/ or ((cortex or cortical or cortico or adrenocortical) adj2 (hormone* or steroid*)).ti,ab,kf. or corticoid*.ti,ab,kf. or corticosteroid.ti,ab,kf. or cortisone.ti,ab,kf. or adrenocorticosteroid*.ti,ab,kf. | 1076672 |
| 8 | 1 and (2 or 3 or 4 or 5 or 6 or 7) | 468 |
| 9 | limit 8 to (english language and yr="2000 -Current") | 385 |
| 10 | meta-analysis/ or meta-analysis as topic/ or (metaanaly* or meta-analy* or metanaly*).ti,ab,kf. or systematic review/ or cochrane.jw. or (prisma or prospero).ti,ab,kf. or ((systemati* or scoping or umbrella or "structured literature") adj3 (review* or overview*)).ti,ab,kf. or (systemic* adj1 review*).ti,ab,kf. or ((systemati* or literature or database* or data-base*) adj10 search*).ti,ab,kf. or ((structured or comprehensive* or systemic*) adj3 search*).ti,ab,kf. or ((literature adj3 review*) and (search* or database* or data-base*)).ti,ab,kf. or (("data extraction" or "data source*") and "study selection").ti,ab,kf. or ("search strategy" and "selection criteria").ti,ab,kf. or ("data source*" and "data synthesis").ti,ab,kf. or (medline or pubmed or embase or cochrane).ab. or ((critical or rapid) adj2 (review* or overview* or synthes*)).ti. or (((critical* or rapid*) adj3 (review* or overview* or synthes*)) and (search* or database* or data-base*)).ab. or (metasynthes* or meta-synthes*).ti,ab,kf. | 480558 |
| 11 | exp clinical trial/ or randomized controlled trial/ or exp clinical trials as topic/ or randomized controlled trials as topic/ or Random Allocation/ or Double-Blind Method/ or Single-Blind Method/ or (clinical trial, phase i or clinical trial, phase ii or clinical trial, phase iii or clinical trial, phase iv or controlled clinical trial or randomized controlled trial or multicenter study or clinical trial).pt. or random*.ti,ab. or (clinic* adj trial*).tw. or ((singl* or doubl* or treb* or tripl*) adj (blind$3 or mask$3)).tw. or Placebos/ or placebo*.tw. | 2216703 |
| 12 | Epidemiologic studies/ or case control studies/ or exp cohort studies/ or Controlled Before-After Studies/ or Case control.tw. or cohort*.tw. or Cohort analy$.tw. or (Follow up adj (study or studies)).tw. or (observational adj (study or studies)).tw. or Longitudinal.tw. or Retrospective*.tw. or prospective*.tw. or consecutive*.tw. or Cross sectional.tw. or Cross-sectional studies/ or historically controlled study/ or interrupted time series analysis/ | 3684772 |
| 13 | 9 and 10 | 17 |
| 14 | (9 and 11) not 13 | 52 |
| 15 | (9 and 12) not (13 or 14) | 32 |
| 16 | 13 or 14 or 15 | 101 |

Table 3.2 Medline search strategy

|  | **Embase** | **OVID/MEDLINE** | **Deduplicated** |
| --- | --- | --- | --- |
| SRs | 24 | 17 | 30 |
| RCT | 46 | 52 | 78 |
| Observational | 29 | 32 | 51 |
| Tota\al | 99 | 101 | 159 |

Table 3.3 Search results

| 1. **Author and year** | 1. **Reason for exclusion** |
| --- | --- |
| 1. Chen, 2015 | 1. Incorrect control group and incorrect outcome measures reported |
| 1. Hart, 2019 | 1. Outcome measures reported |
| 1. Orscelik, 2015 | 1. Incorrect controle group |
| 1. Raatikainen, 1990 | Small study population >20 |

Table 3.4 Excluded studies

| **Study reference** | **Study characteristics** | **Patient characteristics** | **Intervention (I)** | **Comparison / control (C)** | **Follow-up** | **Outcome measures and effect size** | **Comments** |
| --- | --- | --- | --- | --- | --- | --- | --- |
| Heintjes, 2004 | SR and meta-analysis of Controlled trials (randomised or not)  *Literature search up to [January/2004]*  **A**: Kannus, 1992  **B**: Suter, 1998  Study design:  **A**: RCT  **B**: RCT  Setting and Country:  **A**: Finland  **B**: n.r.  Source of funding and conflicts of interest:  of diflunisal.  **A**: no conflict of interest.  **B**: n.r. | Inclusion criteria: People suffering from patellofemoral pain syndrome (includinganterior knee pain syndrome and chondromalacia patellae). No restrictions on age or setting were  applied.  Exclusion criteria: Studies which specifically focus on other named knee pathologies  such as HoLa's disease, Osgood Schlatter disease, Sinding-Larsen-  Johansson's disease, iliotibial band friction syndrome, tendinitis,  neuromas, intra-articular pathology including osteoarthritis,  rheumatoid arthritis, traumatic injuries (such as injured ligaments,  meniscal tears, patellar fractures and patellar luxation), plica  syndromes, and more rarely occurring pathologies were excluded.  *2 studies included*  Important patient characteristics at baseline:  Number of patients  **A**: 53  **B**: 42  Groups comparable at baseline?  Yes | Describe intervention:  **A**: 50 mg GAGPS + 10 mg lidocaine (5 injections/week)  **B**: 550 mg Naproxen twice daily for 7 days (NSAID) | Describe control:  **A**: Saline + lidocaine (5 injections/week)  **B**: 550 mmg placebo twice daily for 7 days. | End-point of follow-up:  **A**: 6 weeks and 6 months  **B**: 7 days  For how many participants were no complete outcome data available?  (intervention/control)  **A**: n.r.  **B**: n.r. | **Comparison 1: GAGPS injection vs. placebo injection**  Outcome measure-1: Pain during sporting activities Defined by VAS (0 – 100)  Effect measure: mean difference (95% CI):  **A**: at 6 weeks: MD of -5.0 (-22.34 – 12.34) at 6 months: MD of-0.50 (-18.74 – 17.74).  **B**: n.r.  Outcome measure-2: Function (full squat)  Defined by the number of patients free of symptoms  Effect measure: mean difference (95% CI):  **A**: at 6 weeks: 1.05 (0.68 – 1.60)  At 6 months: 1.05 (0.58 – 1.60)  **B**: n.r.  Outcome measure-2: Function (one leg jumping)  Defined by the number of patients free of symptoms  Effect measure: mean difference (95% CI):  **A:** at 6 weeks: 0.90 (0.44 – 1.83) at 6 months: 0.77 (0.45 – 1.31) **B:** n.r.  Outcome measure – 3: Return to sports  Defined by the number of participants who had returned to sports.  Effect measure: risk ratio (95% CI)).  **A:** at 6 weeks: 1.42 (0.83 – 2.41) **B:** n.r.  Outcome measure-4: Duration of absenteeism  **A**: n.r.  **B**: n.r.  Outcome measure – 5: Satisfaction:  Defined by the number of patients who rated ‘excellent on their overall assessment’.  Effect measure: risk ratio (95% CI)  **A:** at 6 weeks: 0.94 (0.49 – 1.83)  At 6 months: 1.42 (0.83 – 2.41). **B:** n.r.  Outcome measure – 6: Recovery Defined by the number of patients who had recovered completely.  Effect measure: risk ratio (95% CI)  **A:** at 6 weeks: 0.94 (0.49 – 1.83) at 6 months: 1.06 (0.75 – 1.51) **B:** n.r.  Outcome measure – 7: Adverse events Defined by any adverse events reported.  Effect measure: risk ratio (95% CI)  **A:** at 6 weeks: 0.35 (0.02 – 8.08)  At 6 months: n.r. **B:** n.r.  **Comparison 2: GAPS injection vs. no injection**  Outcome measure-1: Pain during sporting activities  Defined by VAS (0 – 100)  Effect measure: mean difference (95% CI):  **A:** at 6 weeks: MD of -6.0 (-20.70 – 8.70) at 6 months: MD of 1.06 (0.75 – 1.51)  **B:** n.r.  Outcome measure-2: Function (full squat)  Defined by the number of patients free of symptoms.  Effect measure: risk ratio (95% CI)  **A**: at 6 weeks: 1.97 (0.66 – 5.86) at 6 months: 1.83 (0.94 – 3.56)  **B**: n.r.  Outcome measure-2: Function (one leg jumping)  Defined by the number of patients free of symptoms.  **A:** at 6 weeks: 2.20 (1.03 – 4.68)  At 6 months: 1.35 (0.62 – 2.94) **B:**  Outcome measure – 3: Return to sports  Defined by the number of patients who had returned to full physical activity.  Effect measure: risk ratio (95% CI)  **A:** at 6 weeks: 1.33 (0.8 – 2.23) at 6 months: 1.4 (0.92 – 2.14) **B:** n.r.  Outcome measure-4: Duration of absenteeism  **A**: n.r.  **B**: n.r.  Outcome measure – 5: Satisfaction:  Defined by the number of patients who rated ‘excellent on their overall assessment’.  Effect measure: risk ratio (95% CI)  **A:** at 6 weeks: 1 (0.5 – 2) at 6 months: 1.09 (0.71 – 1.69**) B:** n.r.  Outcome measure – 6: Recovery Defined by the number of patients who had recovered completely.  Effect measure: risk ratio (95% CI)  **A:** at 6 weeks: 2 (0.88 – 4.54) at 6 months: 1.3 (0.83 – 2.03) **B:** n.r.  Outcome measure – 7: Adverse events **A:** n.r. **B:** n.r.  **Comparison 3: NSAID vs placebo:**  Outcome measure-1: Pain difference after maximal voluntary muscle contractions  Defined by difference in VAS (0 – 100).  Effect measure: mean difference (95% CI):  **A:** n.r.  **B:** at 7 days: -2.9 (-5.4 - -0.4)  Outcome measure-2: Function  **A**: n.r.  **B**: n.r.  Outcome measure – 3: Return to sports  **A**: n.r.  **B**: n.r.  Outcome measure – 4: Duration of absenteeism  **A**: n.r.  **B**: n.r.  Outcome measure – 5: Satisfaction:  **A**: n.r.  **B**: n.r.  Outcome measure – 6: Recovery **A**: n.r.  **B**: n.r.  Outcome measure – 7: Adverse events **A:** n.r. **B:** n.r. | Facultative:  Brief description of author’s conclusion: There is only limited evidence for the effectiveness of NSAIDs for short-term pain reduction in PFPS. The evidence for the effect of glycosaminoglycan polysulphate is conflicting and merits further investigation. The anabolic steroid nandrolone may be effective, but is too controversial for treatment of PFPS. |

Table 3.5 Evidence table

| **Study reference**  (first author, publication year) | **Describe method of randomisation^1^** | **Bias due to inadequate concealment of allocation?^2^**  (unlikely/likely/unclear) | **Bias due to inadequate blinding of participants to treatment allocation?^3^**  (unlikely/likely/unclear) | **Bias due to inadequate blinding of care providers to treatment allocation?^3^**  (unlikely/likely/unclear) | **Bias due to inadequate blinding of outcome assessors to treatment allocation?^3^**  (unlikely/likely/unclear) | **Bias due to selective outcome reporting on basis of the results?^4^**  (unlikely/likely/unclear) | **Bias due to loss to follow-up?^5^**  (unlikely/likely/unclear) | **Bias due to violation of**  **intention to treat analysis?^6^**  (unlikely/likely/unclear) |
| --- | --- | --- | --- | --- | --- | --- | --- | --- |
| Kannus, 1992 | After the initial clinical and radiologic evaluations and quadriceps strength measurements, the patients were randomized into 1 of 3 treatment groups: group A was treated conservatively, with rest from pain-producing activities,isometric exercises for the quadriceps muscle, and oral doses of NSAIDs; group B received the same conservative treatment and 5 IA injections (once a week) of 1 ml of physiologic saline combined with 1 ml of lidocaine (10 mg/ml) (placebo injections); and group C was treated in the same way as group B, but 1 ml of GAGPS (50 mg/ml) was used instead of the saline. | Unlikely | Unlikely | Unlikely | Unlikely | Unlikely | Unlikely | Unlikely |
| Suter, 1998 | RCT, randomisation method not defined, double blind. | Unclear | Unlikely | Unlikely | Unlikely | Unlikely | Unlikely | Likely |

Table 3.6 Risk of bias table

1. Randomisation: generation of allocation sequences have to be unpredictable, for example computer generated random-numbers or drawing lots or envelopes. Examples of inadequate procedures are generation of allocation sequences by alternation, according to case record number, date of birth or date of admission.
2. Allocation concealment: refers to the protection (blinding) of the randomisation process. Concealment of allocation sequences is adequate if patients and enrolling investigators cannot foresee assignment, for example central randomisation (performed at a site remote from trial location) or sequentially numbered, sealed, opaque envelopes. Inadequate procedures are all procedures based on inadequate randomisation procedures or open allocation schedules..
3. Blinding: neither the patient nor the care provider (attending physician) knows which patient is getting the special treatment. Blinding is sometimes impossible, for example when comparing surgical with non-surgical treatments. The outcome assessor records the study results. Blinding of those assessing outcomes prevents that the knowledge of patient assignement influences the proces of outcome assessment (detection or information bias). If a study has hard (objective) outcome measures, like death, blinding of outcome assessment is not necessary. If a study has “soft” (subjective) outcome measures, like the assessment of an X-ray, blinding of outcome assessment is necessary.
4. Results of all predefined outcome measures should be reported; if the protocol is available, then outcomes in the protocol and published report can be compared; if not, then outcomes listed in the methods section of an article can be compared with those whose results are reported.
5. If the percentage of patients lost to follow-up is large, or differs between treatment groups, or the reasons for loss to follow-up differ between treatment groups, bias is likely. If the number of patients lost to follow-up, or the reasons why, are not reported, the risk of bias is unclear
6. Participants included in the analysis are exactly those who were randomized into the trial. If the numbers randomized into each intervention group are not clearly reported, the risk of bias is unclear; an ITT analysis implies that (a) participants are kept in the intervention groups to which they were randomized, regardless of the intervention they actually received, (b) outcome data are measured on all participants, and (c) all randomized participants are included in the analysis.

Summary of literature

The Cochrane systematic review of Heintjes (2004) described the effects of different types of pharmacotherapy for patients with PFP. In total, eight randomized trials were included in this review, comparing the following medical treatments: different types of NSAIDs versus placebo, different types of NSAIDs versus laser therapy, NSAID versus another NSAID, glucocorticoid steroids versus ultrasound therapy, anabolic steroids versus placebo, glycosaminoglycan polysulphate (GAGPS) (intra-articular injection) versus placebo injection and GAGPS versus no injection. To answer our clinical question, only the data of two studies were extracted from this review (Kannus, 1992 and Suter, 1998). Three studies were not in line with the comparison that was defined by the expert panel (Antich 1986, Marchese 1998, Fulkerson, 1986) and two studies did not report outcome measures that were defined by the expert panel (Bentley, 1981; Darracott, 1973).

Kannus (1992) described a randomized controlled trial, including 53 participants who suffered from chronic (mean: 16 months, SD: 19 months) PFP. Patient were allocated in three groups: The intervention group (n=16) received intra-articular injections of GAGPS (50 mg) plus basic conservative treatment (elimination of symptom-producing activities, quadriceps exercises and oral doses of NSAIDs). The placebo group received placebo injections (1 ml lidocaine, 10 mg/ml) plus basic conservative treatment (n=17). The control group received basic conservative treatment alone (n=16).

Four patients were lost to follow-up or no complete outcome data were available due to inadvertent randomization (n=2), absence during first examination (n=1) and discontinuation of treatment due to reactive synovitis (n=1). The follow-up was at the end of the treatment (six weeks) and at six months. The effects were evaluated on patients’ pain, function, return to sports, satisfaction, recovery and adverse events.

Raatikainen (1990) described a randomized controlled trial, including 31 patients with chondromalacia who were allocated in two groups: The intervention group (n=13) received 12 intramuscular GAGPS injections (50 mg) and the control group (n=14) received placebo injections (physiologic saline). One patient was lost to follow-up and from three patients no complete outcome data were available. The follow-up was at the end of the treatment (six weeks), at ten weeks and after 58 weeks. The effects were evaluated on patients’ pain when going downstairs and patients’ recovery, corresponding the overall therapeutic effect assessed by the physician. The outcome measures were evaluated using a four-point scale: 0=none, 1=slight, 3=severe or extensive.

Suter (1998) described a double-blind randomized trial, including 42 PFP patients who were allocated in two groups: The intervention group (n=20) received naproxen (550 mg) and the control group (n=22) received placebo (not described). Six patients dropped out because of drug intolerance or time constraints. The follow-up was at the end of treatment (seven days). The effect was evaluated on patients’ pain only.

#

# Results

As different types of interventions and controls were compared in the included studies, we divided the results in three comparisons:

1. GAGPS injection versus placebo injection (Kannus, 1992; Raatikainen, 1990)

2. GAGPS injection versus no injection (Kannus, 1992)

3. NSAID administration versus placebo (Suter, 1998)

#

# GAGPS injection versus placebo injection

Pain (crucial)

Pain was measured in two studies included in the review (Kannus 1992, Raatikainen 1990). Kannus (1992) assessed pain during sporting activities with a VAS (0=no pain to 100=worst pain) in the short-term (after six weeks) and in the long-term (after six months). In the short-term, data resulted in a mean difference (MD) of -5.0 (95% CI: -22.34 to 12.34), favouring GAGPS injections. In the long-term, data resulted in a MD of -0.50 (95% CI: -18.74 to 17.74). Both differences were not clinically relevant. Raatikainen (1990) assessed pain when going downstairs by using a four-point scale (0=no pain to 4=severe or extensive pain). The number of patients who had improved after one year was reported. Results showed that more patients in the GAGPS injection group had improved (92%), compared to the placebo injection group (50%). Data resulted in a RR of 1.85 (95% CI: 1.07 to 3.19). This difference was clinically relevant.

Level of evidence of the literature

The level of evidence regarding the outcome pain started at high as it was based on randomized controlled trials. In the short-term and in the long-term, the level of evidence was downgraded by two levels due to crossing the borders of clinical relevance and limited number of included patients (-2; imprecision). The final level is low. After one year, the level of evidence was downgraded by three levels due to surrogate outcome measure (indirectness; -1) crossing the borders of clinical relevance, limited number of included patients (-2; imprecision). The final level is very low.

Function (crucial)

Function was measured by the number of patients free of symptoms (during a full squat and one leg jumping). Function in the short-term (after six weeks) and long-term (after six months) were presented in one study in the review (Kannus, 1992). In the short-term, data resulted in a risk ratio (RR) of 1.05 (95% CI: 0.68 to 1.60) during a full squat, favouring placebo injection. Data resulted in a RR of 0.90 (95% CI: 0.44 to 1.83) during one leg jumping, favouring GAGPS injection. Only during one leg jumping, this difference was clinically relevant. In the long-term, data resulted in a RR of 1.05 (95% CI: 0.68 to 1.60) during a full squat, favouring placebo injection. Data resulted in a RR of 0.77 (95% CI: 0.45 to 1.31) during one leg jumping, favouring GAGPS injection. Only during one leg jumping, this difference was clinically relevant.

Level of evidence of the literature

The level of evidence regarding the outcome function started at high as it was based on a randomized controlled trial. In the short-term and on the long-term, the level of evidence was downgraded by three levels due to surrogate outcome measures (-1; indirectness), crossing the line of clinical relevance and limited number of included patients (-2; imprecision). The final level is very low.

Return to sports (important)

Return to sports measured with the Tegner activity score (0=no return, 10=completely returned) was presented in one study in the review (Kannus, 1992). Return to sports was measured in the short-term (after six weeks) and in the long-term (after six months). In the short-term, data resulted in a MD of 0.60 (95% CI: -0.36 to 1.56), favouring placebo injection. In the long-term, data resulted in a MD of -0.20 (95% CI: -1.22 to 0.82), favouring GAGPS injection. Both differences were not clinically relevant.

Level of evidence of the literature

The level of evidence regarding the outcome return to sports started at high as it was based on a randomized controlled trial. On the short-term and on the long-term, the level of evidence was downgraded by two levels due to crossing the borders of clinical relevance and limited number of included patients (-2; imprecision). The final level is low.

Duration of absenteeism (important)

Duration of absenteeism was not described as an outcome in the included studies.

Level of evidence of the literature

The level of evidence regarding the outcome duration of absenteeism was not assessed due to lack of studies.

Satisfaction (important)

Satisfaction was measured by the number of patients who rated ‘excellent’ at their overall assessment in one study in the review (Kannus 1992). Satisfaction in the short-term (after six weeks) and in the long-term (after six months) were presented. In the short-term, less patients were satisfied in the GAGPS injection group (50%), compared to the placebo injection group (53%). Data resulted in a RR of 0.94 (95% CI: 0.49 to 1.83). In the long-term, more patients were satisfied in the GAGPS injection group (75%), compared to the placebo injection group (53%). Data resulted in a RR of 1.42 (95% CI: 0.83 to 2.41). Only in the long-term, this difference was clinically relevant.

Level of evidence of the literature

The level of evidence regarding the outcome satisfaction started at high as it was based on a randomized controlled trial. On the short-term and on the long-term, the level of evidence was downgraded by three levels due to crossing the line of clinical relevance, limited number of included patients (-2; imprecision) and bias due to indirect outcome reporting (-1; indirectness) The final level is very low.

Recovery (important)

Recovery was measured in two studies included in the review (Kannus 1992; Raatikainen 1990). Kannus (1992) assessed recovery on a five-point scale (1=complete subjective functional and clinical recovery to 5=overall status worse than before treatment), evaluated by a physician. The number of patients who had recovered completely was reported. Recovery in the short-term (after 6 weeks) and in the long-term (after six months) were presented. In the short-term, data resulted in a RR of 0.89 (95% CI: 0.54 to 1.44), favouring placebo. In the long-term, data resulted in a RR of 1.06 (95% CI: 0.75 to 1.51), favouring GAGPS injection. Only in the short-term, this difference was clinically relevant. Raatikainen (1990) assessed recovery on a four-point scale, evaluated by a physician. The number of patients with a moderate to good therapeutic effect was reported after one year. Results showed that more patients recovered in the GAGPS injection group (78%) compared to the placebo injection group (20%). Data resulted in a RR of 3.85 (1.34 to 11.05). This difference was clinically relevant.

Level of evidence of the literature

The level of evidence regarding the outcome recovery started at high as it was based on a randomized controlled trial. In the short-term and in the long-term, the level of evidence was downgraded by three levels due to surrogate outcome measures (-1; indirectness), crossing the borders of clinical relevance and limited number of included patients (-2; imprecision). The final level is very low. At one year, the level of evidence was downgraded by three levels due to indirect outcome reporting (-1; indirectness) and limited number of included patients (-2; imprecision). The final level is very low.

Adverse events (important)

Adverse events were measured in one study in the review (Kannus 1992), including any adverse events or complications. In the short-term, data resulted in a RR of 0.35 (95% CI: 0.02 to 8.08), favouring GAGPS injection. This difference was clinically relevant. In the long-term, no adverse events were reported.

The level of evidence in the literature

The level of evidence regarding the outcome adverse events started at high as it was based on a randomized controlled trial. In the short-term, the level of evidence was downgraded by two levels due to crossing the borders of clinical relevance and limited number of included patients (-2, imprecision). The final level is low. At one year, the level of evidence was downgraded by three levels due to indirect outcome reporting (-1; indirectness) and limited number of included patients (-2; imprecision). The final level is very low.

# Conclusions

Pain (crucial)

| **Low GRADE** | The evidence suggests that GAGPS injection results in little to no difference in pain in the short-term and in the long-term, compared to placebo injection.  *Sources: Heintjes, 2004; Kannus, 1992* |
| --- | --- |

| **Very low GRADE** | The evidence is very uncertain about the effect of GAGPS injection on pain after one year, compared to placebo injection.  *Sources: Heintjes, 2004; Raatikainen, 1990* |
| --- | --- |

Function (crucial)

| **Very Low GRADE** | The evidence is very uncertain about the effect of GAGPS injection on function in the short-term and in the long-term, compared to placebo injection.  *Sources: Heintjes, 2004; Kannus, 1992* |
| --- | --- |

Return to sports (important)

| **Low GRADE** | The evidence suggests that GAGPS injection may result in a slightly negative effect on return to sports in the short-term, compared to placebo injection.  *Sources: Heintjes, 2004; Kannus, 1992* |
| --- | --- |

| **Low GRADE** | The evidence suggests that GAGPS injection may result in a slightly positive effect on return to sports in the long-term, compared to placebo injection.  *Sources: Heintjes, 2004; Kannus, 1992* |
| --- | --- |

Duration of absenteeism (important)

| **- GRADE** | Duration of absenteeism was not reported in the included studies.  *Sources: -* |
| --- | --- |

Satisfaction (important)

| **Very low GRADE** | The evidence is very uncertain about the effect of GAGPS injection on satisfaction in the short-term and in the long-term, compared to placebo injection.  *Sources: Heintjes, 2004; Kannus, 1992* |
| --- | --- |

Recovery (important)

| **Very low GRADE** | The evidence is very uncertain about the effect of GAGPS injection on function in the short-term and in the long-term, compared to placebo injection.  *Sources: Heintjes, 2004; Kannus, 1992* |
| --- | --- |

Adverse events (important)

| **Low GRADE** | The evidence suggests that GAGPS injection may result more adverse events in the short-term, compared to placebo injection.  *Sources: Heintjes, 2004; Kannus, 1992* |
| --- | --- |

| **Low GRADE** | The evidence suggests that GAGPS injection results in little to no difference in adverse events in the long-term, compared to placebo injection.  *Sources: Heintjes, 2004; Kannus, 1992* |
| --- | --- |

#

# GAGPS injection versus no injection

Pain (crucial)

Pain was measured with a VAS (0=no pain to 100=worst pain) in one study in the review (Kannus, 1992). Pain during sporting activities in the short-term (after six weeks) and in the long-term (after six months) were presented. In the short-term, data resulted in a MD of -6.0 (95% CI: -20.70 to 8.70), favouring GAGPS injection. In the long-term, data resulted in a MD of 2.0 (95% CI: -15.14 to 19.14), favouring no injection. Both differences were neither statistically significant nor clinically relevant between the two groups.

Level of evidence of the literature

The level of evidence regarding the outcome pain started at high as it was based on a randomized controlled trial. At six weeks, the level of evidence was downgraded by two levels due crossing the borders of clinical relevance and limited number of included patients (-2; imprecision). The final level is low. At six months, the level of evidence was downgraded by two levels due to limited number of included patients (-2; imprecision). The final level is low.

Function (crucial)

Function was measured by the number of patients free of symptoms (during a full squat and one leg jumping). Function in the short-term (after six weeks) and in the long-term (after six months) were presented in one study in the review (Kannus, 1992). In the short-term, data resulted in a RR of 2.20 (95% CI: 1.03 to 4.68), favouring GAGPS injection during a full squat. During one leg jumping, this data resulted in a RR of 1.97 (95% CI: 1.14 to 3.95), favouring GAGPS injection. In the long-term, data resulted in a RR of 1.83 (95% CI: 0.94 to 3.56) when doing a full squat, favouring GAGPS injection. During one leg jumping, this data resulted in a RR of 1.35 (95% CI: 0.62 to 2.94), favouring GAGPS injection. All differences were clinically relevant.

Level of evidence of the literature

The level of evidence regarding the outcome function started at high as it was based on a randomized controlled trial. In all measurements, the level of evidence was downgraded by three levels due to study limitations (risk-of-bias, -1), crossing the line of clinical relevance and limited number of included patients (-2; imprecision). The final level is very low.

Return to sports (important)

Return to sports measured with the Tegner activity score (0=no return, 10=completely returned) was presented in one study in the review (Kannus, 1992). Return to sports was measured in the short-term (after six weeks) and in the long-term (after six months). In the short-term, data resulted in a MD of -1.90 (95% CI: -3.04 to -0.76), favouring GAGPS injection. In the long-term, data resulted in a MD of -1.50 (95% CI: -2.86 to -0.14), favouring GAGPS injection. The differences were not clinically relevant.

Level of evidence of the literature

The level of evidence regarding the outcome return to sports started at high as it was based on a randomized controlled trial. In the short-term and in the long-term, the level of evidence was downgraded by two levels due crossing the borders of clinical relevance and limited number of included patients (-2; imprecision). The final level is low.

Duration of absenteeism (important)

Duration of absenteeism was not described as an outcome in the included studies.

Level of evidence of the literature

The level of evidence regarding the outcome duration of absenteeism was not assessed due to lack of studies.

Satisfaction (important)

Satisfaction was not described as an outcome in the included studies.

Level of evidence of the literature

The level of evidence regarding the outcome satisfaction was not assessed due to lack of studies.

Recovery (important)

Recovery was measured by the number of patients who had recovered completely in one study in the review (Kannus, 1992) and was determined by a physician. The physician rated the overall therapeutic effect on a 5-point scale, from 1 = complete subjective, functional and clinical recovery to 5 = overall status worse than before treatment. Recovery in the short-term (after 6 weeks) and in the long-term (after six months) were presented. In the short-term, data resulted in a RR of 2 (95% CI: 0.88 to 4.54), favouring GAGPS injection. In the long-term, data resulted in a RR of 1.3 (95% CI: 0.83 to 2.03), favouring GAGPS injection. Both differences were clinically relevant.

Level of evidence of the literature

The level of evidence regarding the outcome recovery started at high as it was based on a randomized controlled trial. In the short-term and in the long-term, the level of evidence was downgraded by two levels due limited number of included patients and crossing the line of clinical relevance (-2; imprecision). The final level is low.

Adverse events (important)

Adverse events were measured in the study in the review (Kannus 1992), including any adverse events or complications. In the short-term and in the long-term, no adverse events were reported.

The level of evidence in the literature

The level of evidence regarding the outcome adverse events started at high as it was based on a randomized controlled trial. In the short-term and in the long-term, the level of evidence was downgraded by two levels due to limited number of included patients (-2; imprecision). The final level is low.

# Conclusions

Pain (crucial)

| **Low GRADE** | The evidence suggests that GAGPS injection results in little to no difference in pain in the short-term and in the long-term, compared to no injection.  *Sources: Heintjes, 2004; Kannus, 1992* |
| --- | --- |

Function (crucial)

| **Very low GRADE** | The evidence is very uncertain about the effect of GAGPS injection on function in the short-term and in the long-term, compared to no injection.  *Sources: Heintjes, 2004; Kannus, 1992* |
| --- | --- |

Return to sports (important)

| **Low GRADE** | The evidence suggests that GAGPS injection results in little to no difference in return to sports in the short-term and in the long-term, compared to no injection.  *Sources: Heintjes, 2004; Kannus, 1992* |
| --- | --- |

Duration of absenteeism (important)

| **- GRADE** | Duration of absenteeism was not reported in the included studies.  *Sources: -* |
| --- | --- |

Satisfaction (important)

| **- GRADE** | Satisfaction was not reported in the included studies.  *Sources: -* |
| --- | --- |

Recovery (important)

| **Low GRADE** | The evidence suggests that GAGPS injection may result in a slightly positive effect on recovery in the short-term and in the long-term, compared to no injection.  *Sources: Heintjes, 2004; Kannus, 1992* |
| --- | --- |

Adverse events (important)

| **Low GRADE** | The evidence suggests that GAGPS injection results in little to no difference in adverse events in the short-term and in the long-term, compared to no injection.  *Sources: Heintjes, 2004; Kannus, 1992* |
| --- | --- |

#

# NSAID administration versus placebo

Pain (crucial)

Pain difference before and after maximal voluntary muscle contractions was measured with a VAS (0=no pain to 100=worst pain) in one study included in the review (Suter, 1998). Pain difference in the short-term (after seven days) was presented. Data resulted in a MD of -2.9 (95% CI: -5.40 to -0.40), favouring NSAID administration (naproxen). This difference was not clinically relevant.

Level of evidence of the literature

The level of evidence regarding the outcome pain started at high as it was based on a randomized controlled trial but was downgraded by three levels because of inadequate concealment allocation (-1; risk of bias), limited number of included patients and crossing the borders of clinical relevance (-2; imprecision). The final level is very low.

Function; Return to sports; Duration of absenteeism; Satisfaction; Recovery

Suter (1998) did not include these outcomes.

Level of evidence of the literature

The level of evidence regarding the outcomes function; return to sports; duration of absenteeism; satisfaction; and recovery were not assessed due to lack of studies.

# Conclusions

Pain (crucial)

| **Very low GRADE** | The evidence is very uncertain about the effect of NSAID administration on recovery in the short-term, compared to placebo.  *Sources: Heintjes, 2004; Suter, 1998* |
| --- | --- |

Function; Return to sports; Duration of absenteeism; Satisfaction; Recovery; Adverse events

| **- GRADE** | Function, return to sports, duration of absenteeism, satisfaction, recovery and adverse events were not reported in the included studies.  *Sources: -* |
| --- | --- |

#

# Considerations – from evidence to recommendation

Looking at the conclusions regarding the crucial outcome measures pain and function, there is no clinically relevant effect of pain medications in PFP. The strength of evidence for the crucial outcome measures is low to very low, so the overall strength of evidence is also very low. Due to the small study populations and failure to cross the line of clinical relevance. Therefore, based on the literature, no recommendations can be made on the effect of pain medications (including injection therapy) on pain and function in patients with PFP. The literature summary cannot help us provide a direction for decision-making.

All the more important is to consider any adverse effects of the treatments. In one patient in the study by Kannus (1992), reactive synovitis occurred after administration of intra-articular placebo injection with physiological saline. Otherwise, no complications, side effects or other problems were reported. Of the total 42 patients included in Suter's (1998) study, 3 dropped out because of medication intolerance. What this intolerance entailed was not mentioned.

For further information of the side effects of pain medications, is referred to the Pharmacotherapeutic Compass (FK 2018; EPHOR 2014) and the NHG Standard on Pain (NHG 2018), official Summary of Product Characteristics (SmPC) information of the respective drugs can be accessed at http://www.cbg-med.nl

Expectations and values of patients (and possibly their caretakers)

To gain more insight into the values and expectations of patients with PFP, a questionnaire was prepared and distributed in collaboration with the Netherlands Patients Federation (NPF). Of the 43 patients who completed the questionnaire, 14 had some form of medication (analgesic n = 8, anti-inflammatory n = 6). None of the patients received injection therapy. Main reason for choosing medication is less pain, allowing, for example, ADL tasks to be performed better. One patient was satisfied with medication, the other 5 were neutral about it. The disadvantages mentioned were "temporary solution," "does not take away pain completely," and "risk of going beyond limit”.

Main reasons for choosing anti-inflammatory medication were less pain and the fact that it has an anti-inflammatory effect in addition to being analgesic. Two patients were satisfied with the anti-inflammatory medication, 1 neutral, 1 dissatisfied and 1 very dissatisfied. The main disadvantages are "temporary solution," "does not completely eliminate pain," and "serious side effects such as stomach upset and erectile dysfunction”.

The expert panel concludes from the above that patients do not see pain medications as a definitive solution.

Cost

No studies have been conducted on the (cost) effectiveness of pain medications for PFP. The cost of pain medications is low. Somewhat higher costs are associated with use of dermal NSAIDs and the comedication with a gastric protector, but this should not prevent short-term use, according to the expert panel.

Medication treatment of PFP in the form of paracetamol or NSAIDs is used in practice despite the lack of evidence. Injection therapy, to the knowledge of the expert panel, is not used in practice.

The only therapy for which there is some evidence, both positive and negative, are GAGPS injections. This form of therapy is not used in the Netherlands and because of the also very limited evidence base, it is not recommended by the expert panel.

All other forms of drug therapy in PFP have not been sufficiently studied and therefore cannot be recommended.

# Rationale of the recommendations

The strength of evidence for the crucial outcome measures is low to very low, the overall weight of evidence is also very low. Because small study populations and not crossing the line of clinical relevance. Therefore, no recommendations can be made on the basis of the literature on the effect of pain medications, (including injection therapy), on pain and function in patients with PFP. For patients pain medications is no definitive solution. The limited number of low/very low quality studies do not allow the recommendation of a specific pain medications. The expert panel does not recommend the use of medication for the treatment of patients with PFP. The expert panel recommends starting with exercise therapy and possibly additional conservative therapy (see module exercise therapy and module additional conservative treatments). Patients with PFP have pain on daily bases and as a result may also be limited in their daily functioning. Patients are looking for a quick solution and prescribing medication is sometimes insurmountable in these situations. A physician, must be well aware that medication can also have disadvantages like dependence and/or overload due to pain suppression. In situations with a lot of pain and limitations, short-term use of pain medications can be considered. Based on possible side effects, the expert panel prefers first choice paracetamol and second choice dermal NSAID (Derry 2015). The patient should be informed that medication is not a solution and the expert panel recommends combination with exercise therapy and possibly additional conservative therapy (see module 1 and 2).

# References

Derry S, Moore RA, Gaskell H, McIntyre M, Wiffen PJ. (2015). Topical NSAIDs for acute musculoskeletal pain in adults. *Cochrane Database Syst Rev;*(6):CD007402*.*

Heintjes, E.M., Berger, M., Bierma-Zeinstra, S.M.A., Bernsen, R.M.D., Verhaar, J.A.N. & Koes, B.W. (2004). *Pharmacotherapy for patellofemoral pain Syndrome, 3,* CD003470.

Kannus, P, Natri A., Niittymäki, S, Järvinen, M. (1992). Effect of intraarticular glycosaminoglycan polysulfate treatment on patellofemoral pain syndrome. *Arthritis and Rheumatism, 35*(9): 1053 – 61.

Raatikainen, T., Väänänen, K., Tamelander, G. (1990) Effect of glycosaminoglycan polysulfate on chondromalacia patellae – a placebo-controlled 1-year study. *Acta Orthopaedica Scandinavia,* 61(5):443-8.

Suter, E. Herzog, W., De Souza, K., Bray. R. (1998). Inhibition of the quadriceps muscles in patients with anterior knee pain. *Journal of Applied Biomechanics, 14*(4): 360 – 73.

# Construction of Module 4 ‘Diagnosing PT’

Scoping Question: How is PT correctly diagnosed?

# Search and select

A systematic review of the literature was performed to answer the following question: What is de diagnostic accuracy of history and/or physical examination to diagnose PT in patients suspected of PT?

P: patients suspected of PT (adults);

I: history and/or physical examination;

C: none;

R: imaging techniques like MRI, Ultrasound, Ultrasound Tissue Characterization;

O: sensitivity, specificity, positive predictive value (PVV), negative predictive value (NPV).

Timing/setting: Secondary care setting of patients with suspected of PT independent of the duration of symptoms.

Relevant outcome measures

Focusing on the secondary care setting, the specificity is more important than the sensitivity of the test. The guideline development expert panel therefore considered specificity and negative predictive value as critical outcome measures for decision making; and sensitivity and positive predictive value, were considered as important outcome measure for decision making.

The expert panel considered a sensitivity or specificity above 80% as sufficient.

Search and select (Methods)

The databases Medline (via OVID), Embase (via Embase.com), Cinahl, and PEDro were searched with relevant search terms until August 4, 2020. The detailed search strategy is depicted in tables. The systematic literature search resulted in 229 hits.

Results

Two studies were included in the analysis of the literature. Important study characteristics and results are summarized in the evidence tables. The assessment of the risk of bias is summarized in the risk of bias tables.

Key articles

lian o et al, relationship between symptoms of jumper’s knee and….scan j of med sci sport, 1996

<https://onlinelibrary.wiley.com/doi/epdf/10.1111/j.1600-0838.1996.tb00473.x>

khan et al , time to bandon tendinitis myth, BMJ, 2002: <https://www.ncbi.nlm.nih.gov/pmc/articles/PMC1122566/pdf/626.pdf>

Studies were selected based on the following criteria:

- systematic reviews, and observational studies;
- included suspected patients with PT;
- included at least one of the defined outcome measures or presented the data to calculate the defined outcomes.

Based on title and abstract screening, 16 studies were initially selected. After reading the full text, 14 studies were excluded (Table 4.3), and two studies were included.

| **Database** | **Search terms** |
| --- | --- |
| Embase | | ***No.*** | ***Query*** | ***Results*** | | --- | --- | --- | | #12 | #9 OR #10 OR #11 | 89 | | #11 | #5 AND #8 NOT (#9 OR #10) | 43 | | #10 | #5 AND #7 NOT #9 | 36 | | #9 | #5 AND #6 | 10 | | #8 | 'major clinical study'/de OR 'clinical study'/de OR 'case control study'/de OR 'family study'/de OR 'longitudinal study'/de OR 'retrospective study'/de OR 'prospective study'/de OR 'cohort analysis'/de OR ((cohort NEAR/1 (study OR studies)):ab,ti) OR (('case control' NEAR/1 (study OR studies)):ab,ti) OR (('follow up' NEAR/1 (study OR studies)):ab,ti) OR (observational NEAR/1 (study OR studies)) OR ((epidemiologic NEAR/1 (study OR studies)):ab,ti) OR (('cross sectional' NEAR/1 (study OR studies)):ab,ti) | 5361976 | | #7 | 'clinical trial'/exp OR 'randomization'/exp OR 'single blind procedure'/exp OR 'double blind procedure'/exp OR 'crossover procedure'/exp OR 'placebo'/exp OR 'prospective study'/exp OR rct:ab,ti OR random*:ab,ti OR 'single blind':ab,ti OR 'randomised controlled trial':ab,ti OR 'randomized controlled trial'/exp OR placebo*:ab,ti | 3091846 | | #6 | 'meta analysis'/de OR cochrane:ab OR embase:ab OR psycinfo:ab OR cinahl:ab OR medline:ab OR ((systematic NEAR/1 (review OR overview)):ab,ti) OR ((meta NEAR/1 analy*):ab,ti) OR metaanalys*:ab,ti OR 'data extraction':ab OR cochrane:jt OR 'systematic review'/de | 513404 | | #5 | #1 AND (#2 OR #4) AND #3 AND [1995-2020]/py NOT ('conference abstract'/it OR 'editorial'/it OR 'letter'/it OR 'note'/it) | 208 | | #4 | 'nuclear magnetic resonance imaging'/exp OR mri*:ti,ab,kw OR nmr:ti,ab,kw OR mra*:ti,ab,kw OR fmri*:ti,ab,kw OR ((('magnetic resonance' OR mr) NEAR/2 (imag* OR tomograph*)):ti,ab,kw) OR 'ultrasound'/exp OR 'ultrasound scanner'/exp OR 'echography'/exp OR ultraso*:ti,ab,kw OR sonograph*:ti,ab,kw OR echograph*:ti,ab,kw OR utc:ti,ab,kw | 2226429 | | #3 | 'sensitivity and specificity'/de OR sensitiv*:ti,ab,kw OR specific*:ti,ab,kw OR 'diagnostic accuracy'/exp OR 'diagnostic test accuracy study'/exp OR accuracy:ti,ab,kw OR 'predictive value'/exp OR ppv:ti,ab,kw OR npv:ti,ab,kw OR 'predictive value':ti,ab,kw OR predict*:ab,ti OR 'roc curve':ab,ti OR 'receiver operator':ab,ti OR 'receiver operators':ab,ti OR likelihood:ab,ti OR 'diagnostic error'/exp OR 'inter observer':ab,ti OR 'intra observer':ab,ti OR interobserver:ab,ti OR intraobserver:ab,ti OR validity:ab,ti OR kappa:ab,ti OR reliability:ab,ti OR reproducibility:ab,ti OR ((test NEAR/2 're-test'):ab,ti) OR ((test NEAR/2 'retest'):ab,ti) OR 'reproducibility'/exp OR 'differential diagnosis'/exp OR 'validation study'/de OR 'measurement precision'/exp OR 'diagnostic value'/exp OR 'reliability'/exp | 8159181 | | #2 | 'physical examination'/exp OR 'clinical examination'/exp OR 'anamnesis'/exp OR anamnesis:ti,ab,kw OR examination*:ti,ab,kw OR 'history taking':ti,ab,kw OR 'medical interview*':ti,ab,kw | 1500380 | | #1 | 'patellar tendinopathy'/exp OR 'jumper s knee':ti,ab,kw OR 'patella apicitis':ti,ab,kw OR 'patellar apicitis':ti,ab,kw OR 'patella apex syndrome':ti,ab,kw OR 'patellar apex syndrome':ti,ab,kw OR 'patella tip syndrome':ti,ab,kw OR 'patellar tip syndrome':ti,ab,kw OR 'patella tenosynovitis':ti,ab,kw OR 'patellar tenosynovitis':ti,ab,kw OR (('patellar ligament'/exp OR 'patella'/exp OR 'patella':ti,ab,kw OR 'patellar':ti,ab,kw) AND ('tendinitis'/exp OR 'tendinopathy':ti,ab,kw OR 'tendinopathies':ti,ab,kw OR 'tendinosis':ti,ab,kw OR 'tendinoses':ti,ab,kw OR 'tendinitis':ti,ab,kw OR 'tendinitides':ti,ab,kw OR 'tendonitis':ti,ab,kw OR 'tendonitides':ti,ab,kw OR 'peritendinitis':ti,ab,kw OR 'paratenonitis':ti,ab,kw OR ('tendon':ti,ab,kw AND ('pathology':ti,ab,kw OR 'rupture':ti,ab,kw OR 'ruptures':ti,ab,kw)))) | 2851 | |
| Medline (OVID) | 1 ("jumper s knee" or "patella apicitis" or "patellar apicitis" or "patella apex syndrome" or "patellar apex syndrome" or "patella tip syndrome" or "patellar tip syndrome" or "patella tenosynovitis" or "patellar tenosynovitis").ti,ab,kf. or ((exp Patellar Ligament/ or exp Patella/ or "Patella".ti,ab,kf. or "patellar".ti,ab,kf.) and (Tendinopathy/ or "Tendinopathy".ti,ab,kf. or "Tendinopathies".ti,ab,kf. or "tendinosis".ti,ab,kf. or "tendinoses".ti,ab,kf. or "tendinitis".ti,ab,kf. or "Tendinitides".ti,ab,kf. or "tendonitis".ti,ab,kf. or "Tendonitides".ti,ab,kf. or "peritendinitis".ti,ab,kf. or "paratenonitis".ti,ab,kf. or ("tendon" and ("pathology" or "rupture" or "ruptures")).ti,ab,kf.)) (2106)  2 exp physical examination/ or exp Medical History Taking/ or (anamnesis or examination or 'history taking' or 'medical interview*').ti,ab,kf. (2009967)  3 exp "Sensitivity and Specificity"/ or (Sensitiv* or Specific*).ti,ab. or (predict* or ROC-curve or receiver-operator*).ti,ab. or (likelihood or LR*).ti,ab. or exp Diagnostic Errors/ or (inter-observer or intra-observer or interobserver or intraobserver or validity or kappa or reliability).ti,ab. or reproducibility.ti,ab. or (test adj2 (re-test or retest)).ti,ab. or "Reproducibility of Results"/ or accuracy.ti,ab. or Diagnosis, Differential/ or Validation Studies.pt. or ppv.ti,ab,kf. or npv.ti,ab,kf. or 'predictive value'.ti,ab,kf. or exp Diagnostic Errors/ (6568253)  4 exp Magnetic Resonance Imaging/ or mri*.ti,ab,kf. or nmr.ti,ab,kf. or mra*.ti,ab,kf. or fmri*.ti,ab,kf. or (('magnetic resonance' or mr) adj2 (imag* or tomograph*)).ti,ab,kf. or exp Ultrasonography/ or exp Ultrasonics/ or ultraso*.ti,ab,kf. or sonograph*.ti,ab,kf. or echograph*.ti,ab,kf. or utc.ti,ab,kf. (1376068)  5 1 and (2 or 4) and 3 (209)  6 limit 5 to yr="1995 -Current" (202)  7 (meta-analysis/ or meta-analysis as topic/ or (meta adj analy$).tw. or ((systematic* or literature) adj2 review$1).tw. or (systematic adj overview$1).tw. or exp "Review Literature as Topic"/ or cochrane.ab. or cochrane.jw. or embase.ab. or medline.ab. or (psychlit or psyclit).ab. or (cinahl or cinhal).ab. or cancerlit.ab. or ((selection criteria or data extraction).ab. and "review"/)) not (Comment/ or Editorial/ or Letter/ or (animals/ not humans/)) (457917)  8 (exp clinical trial/ or randomized controlled trial/ or exp clinical trials as topic/ or randomized controlled trials as topic/ or Random Allocation/ or Double-Blind Method/ or Single-Blind Method/ or (clinical trial, phase i or clinical trial, phase ii or clinical trial, phase iii or clinical trial, phase iv or controlled clinical trial or randomized controlled trial or multicenter study or clinical trial).pt. or random*.ti,ab. or (clinic* adj trial*).tw. or ((singl* or doubl* or treb* or tripl*) adj (blind$3 or mask$3)).tw. or Placebos/ or placebo*.tw.) not (animals/ not humans/) (2010012)  9 Epidemiologic studies/ or case control studies/ or exp cohort studies/ or Controlled Before-After Studies/ or Case control.tw. or (cohort adj (study or studies)).tw. or Cohort analy$.tw. or (Follow up adj (study or studies)).tw. or (observational adj (study or studies)).tw. or Longitudinal.tw. or Retrospective*.tw. or prospective*.tw. or consecutive*.tw. or Cross sectional.tw. or Cross-sectional studies/ or historically controlled study/ or interrupted time series analysis/ [Onder exp cohort studies vallen ook longitudinale, prospectieve en retrospectieve studies] (3487543)  10 6 and 7 (11)  11 (6 and 8) not 10 (31)  12 (6 and 9) not (10 or 11) (91)  13 10 or 11 or 12 (133) |
| Cinahl | | **#** | **Query** | **Results** | | --- | --- | --- | | S5 | S1 AND (S2 OR S4) AND S3 | 123 | | S4 | (MH "Magnetic Resonance Imaging+") OR TI mri* OR TI nmr OR TI mra* OR TI fmri* OR AB mri* OR AB nmr OR AB mra* OR AB fmri* OR TI ('magnetic resonance' or mr) N2 (imag* or tomograph*)) OR AB ('magnetic resonance' or mr) N2 (imag* or tomograph*)) OR (MH "Ultrasonography+") OR TI ultraso* OR TI sonograph* OR TI echograph* OR TI utc OR AB ultraso* OR AB sonograph* OR AB echograph* OR AB utc | 292,719 | | S3 | (MH "Sensitivity and Specificity") OR (MH "Diagnostic Errors+") OR (MH "Predictive Value of Tests") OR (MH "Reproducibility of Results") OR (MH "Diagnosis, Differential") OR (MH "Reliability+") OR (MH "Validation Studies") OR TI sensitiv* OR TI specific* OR TI accuracy OR TI ppv OR TI npv OR TI 'predictive value' OR TI predict* OR TI 'roc curve' OR TI 'receiver operator' OR TI 'receiver operators' OR TI likelihood OR TI 'inter observer' OR TI 'intra observer' OR TI interobserver OR TI intraobserver OR TI validity OR TI kappa OR TI reliTIility OR TI reproducibility OR TI (test N2 're-test') OR TI (test N2 'retest') OR TI accuracy OR AB sensitiv* OR AB specific* OR AB accuracy OR AB ppv OR AB npv OR AB 'predictive value' OR AB predict* OR AB 'roc curve' OR AB 'receiver operator' OR AB 'receiver operators' OR AB likelihood OR AB 'inter observer' OR AB 'intra observer' OR AB interobserver OR AB intraobserver OR AB validity OR AB kappa OR AB reliability OR AB reproducibility OR AB (test N2 're-test') OR AB (test N2 'retest') OR AB accuracy | 1,262,432 | | S2 | (MH "Physical Examination+") OR (MH "Patient History Taking+") OR (TI anamnesis OR TI examination OR TI history taking OR TI medical interview*) OR (AB anamnesis OR AB examination OR AB history taking OR AB medical interview*) | 282,007 | | S1 | (MH "Patellar Tendinopathy") OR TI jumper s knee OR TI patella apicitis OR TI patellar apicitis OR TI patella apex syndrome OR TI patellar apex syndrome OR TI patella tip syndrome OR TI patellar tip syndrome OR TI patella tenosynovitis OR TI patellar tenosynovitis OR AB jumper s knee OR AB patella apicitis OR AB patellar apicitis OR AB patella apex syndrome OR AB patellar apex syndrome OR AB patella tip syndrome OR AB patellar tip syndrome OR AB patella tenosynovitis OR AB patellar tenosynovitis OR (((MH "Patellar Ligament") OR (MH "Patella") OR TI patella* OR AB patella*) AND ((MH "Tendinopathy+") OR TI tendinopath* OR AB tendinopath OR TI tendinos*s OR AB tendinos*s OR TI Tendinitis OR TI tendinitides OR TI tendonitis OR TI tendonitides OR TI peritendinitis OR TI paratenonitis OR AB Tendinitis OR AB tendinitides OR AB tendonitis OR AB tendonitides OR AB peritendinitis OR AB paratenonitis OR ((TI tendon OR AB tendon) AND (TI pathology OR AB pathology OR TI rupture* OR AB rupture*)))) | 1,029 | |
| PEDro | patella* jumper* tendino* [in titel]  → in body part field: lower leg & knee |

| **Database** | **Search terms** |
| --- | --- |
| Embase | | ***No.*** | ***Query*** | ***Results*** | | --- | --- | --- | | #12 | #9 OR #10 OR #11 | 89 | | #11 | #5 AND #8 NOT (#9 OR #10) | 43 | | #10 | #5 AND #7 NOT #9 | 36 | | #9 | #5 AND #6 | 10 | | #8 | 'major clinical study'/de OR 'clinical study'/de OR 'case control study'/de OR 'family study'/de OR 'longitudinal study'/de OR 'retrospective study'/de OR 'prospective study'/de OR 'cohort analysis'/de OR ((cohort NEAR/1 (study OR studies)):ab,ti) OR (('case control' NEAR/1 (study OR studies)):ab,ti) OR (('follow up' NEAR/1 (study OR studies)):ab,ti) OR (observational NEAR/1 (study OR studies)) OR ((epidemiologic NEAR/1 (study OR studies)):ab,ti) OR (('cross sectional' NEAR/1 (study OR studies)):ab,ti) | 5361976 | | #7 | 'clinical trial'/exp OR 'randomization'/exp OR 'single blind procedure'/exp OR 'double blind procedure'/exp OR 'crossover procedure'/exp OR 'placebo'/exp OR 'prospective study'/exp OR rct:ab,ti OR random*:ab,ti OR 'single blind':ab,ti OR 'randomised controlled trial':ab,ti OR 'randomized controlled trial'/exp OR placebo*:ab,ti | 3091846 | | #6 | 'meta analysis'/de OR cochrane:ab OR embase:ab OR psycinfo:ab OR cinahl:ab OR medline:ab OR ((systematic NEAR/1 (review OR overview)):ab,ti) OR ((meta NEAR/1 analy*):ab,ti) OR metaanalys*:ab,ti OR 'data extraction':ab OR cochrane:jt OR 'systematic review'/de | 513404 | | #5 | #1 AND (#2 OR #4) AND #3 AND [1995-2020]/py NOT ('conference abstract'/it OR 'editorial'/it OR 'letter'/it OR 'note'/it) | 208 | | #4 | 'nuclear magnetic resonance imaging'/exp OR mri*:ti,ab,kw OR nmr:ti,ab,kw OR mra*:ti,ab,kw OR fmri*:ti,ab,kw OR ((('magnetic resonance' OR mr) NEAR/2 (imag* OR tomograph*)):ti,ab,kw) OR 'ultrasound'/exp OR 'ultrasound scanner'/exp OR 'echography'/exp OR ultraso*:ti,ab,kw OR sonograph*:ti,ab,kw OR echograph*:ti,ab,kw OR utc:ti,ab,kw | 2226429 | | #3 | 'sensitivity and specificity'/de OR sensitiv*:ti,ab,kw OR specific*:ti,ab,kw OR 'diagnostic accuracy'/exp OR 'diagnostic test accuracy study'/exp OR accuracy:ti,ab,kw OR 'predictive value'/exp OR ppv:ti,ab,kw OR npv:ti,ab,kw OR 'predictive value':ti,ab,kw OR predict*:ab,ti OR 'roc curve':ab,ti OR 'receiver operator':ab,ti OR 'receiver operators':ab,ti OR likelihood:ab,ti OR 'diagnostic error'/exp OR 'inter observer':ab,ti OR 'intra observer':ab,ti OR interobserver:ab,ti OR intraobserver:ab,ti OR validity:ab,ti OR kappa:ab,ti OR reliability:ab,ti OR reproducibility:ab,ti OR ((test NEAR/2 're-test'):ab,ti) OR ((test NEAR/2 'retest'):ab,ti) OR 'reproducibility'/exp OR 'differential diagnosis'/exp OR 'validation study'/de OR 'measurement precision'/exp OR 'diagnostic value'/exp OR 'reliability'/exp | 8159181 | | #2 | 'physical examination'/exp OR 'clinical examination'/exp OR 'anamnesis'/exp OR anamnesis:ti,ab,kw OR examination*:ti,ab,kw OR 'history taking':ti,ab,kw OR 'medical interview*':ti,ab,kw | 1500380 | | #1 | 'patellar tendinopathy'/exp OR 'jumper s knee':ti,ab,kw OR 'patella apicitis':ti,ab,kw OR 'patellar apicitis':ti,ab,kw OR 'patella apex syndrome':ti,ab,kw OR 'patellar apex syndrome':ti,ab,kw OR 'patella tip syndrome':ti,ab,kw OR 'patellar tip syndrome':ti,ab,kw OR 'patella tenosynovitis':ti,ab,kw OR 'patellar tenosynovitis':ti,ab,kw OR (('patellar ligament'/exp OR 'patella'/exp OR 'patella':ti,ab,kw OR 'patellar':ti,ab,kw) AND ('tendinitis'/exp OR 'tendinopathy':ti,ab,kw OR 'tendinopathies':ti,ab,kw OR 'tendinosis':ti,ab,kw OR 'tendinoses':ti,ab,kw OR 'tendinitis':ti,ab,kw OR 'tendinitides':ti,ab,kw OR 'tendonitis':ti,ab,kw OR 'tendonitides':ti,ab,kw OR 'peritendinitis':ti,ab,kw OR 'paratenonitis':ti,ab,kw OR ('tendon':ti,ab,kw AND ('pathology':ti,ab,kw OR 'rupture':ti,ab,kw OR 'ruptures':ti,ab,kw)))) | 2851 | |
| Medline (OVID) | 1 ("jumper s knee" or "patella apicitis" or "patellar apicitis" or "patella apex syndrome" or "patellar apex syndrome" or "patella tip syndrome" or "patellar tip syndrome" or "patella tenosynovitis" or "patellar tenosynovitis").ti,ab,kf. or ((exp Patellar Ligament/ or exp Patella/ or "Patella".ti,ab,kf. or "patellar".ti,ab,kf.) and (Tendinopathy/ or "Tendinopathy".ti,ab,kf. or "Tendinopathies".ti,ab,kf. or "tendinosis".ti,ab,kf. or "tendinoses".ti,ab,kf. or "tendinitis".ti,ab,kf. or "Tendinitides".ti,ab,kf. or "tendonitis".ti,ab,kf. or "Tendonitides".ti,ab,kf. or "peritendinitis".ti,ab,kf. or "paratenonitis".ti,ab,kf. or ("tendon" and ("pathology" or "rupture" or "ruptures")).ti,ab,kf.)) (2106)  2 exp physical examination/ or exp Medical History Taking/ or (anamnesis or examination or 'history taking' or 'medical interview*').ti,ab,kf. (2009967)  3 exp "Sensitivity and Specificity"/ or (Sensitiv* or Specific*).ti,ab. or (predict* or ROC-curve or receiver-operator*).ti,ab. or (likelihood or LR*).ti,ab. or exp Diagnostic Errors/ or (inter-observer or intra-observer or interobserver or intraobserver or validity or kappa or reliability).ti,ab. or reproducibility.ti,ab. or (test adj2 (re-test or retest)).ti,ab. or "Reproducibility of Results"/ or accuracy.ti,ab. or Diagnosis, Differential/ or Validation Studies.pt. or ppv.ti,ab,kf. or npv.ti,ab,kf. or 'predictive value'.ti,ab,kf. or exp Diagnostic Errors/ (6568253)  4 exp Magnetic Resonance Imaging/ or mri*.ti,ab,kf. or nmr.ti,ab,kf. or mra*.ti,ab,kf. or fmri*.ti,ab,kf. or (('magnetic resonance' or mr) adj2 (imag* or tomograph*)).ti,ab,kf. or exp Ultrasonography/ or exp Ultrasonics/ or ultraso*.ti,ab,kf. or sonograph*.ti,ab,kf. or echograph*.ti,ab,kf. or utc.ti,ab,kf. (1376068)  5 1 and (2 or 4) and 3 (209)  6 limit 5 to yr="1995 -Current" (202)  7 (meta-analysis/ or meta-analysis as topic/ or (meta adj analy$).tw. or ((systematic* or literature) adj2 review$1).tw. or (systematic adj overview$1).tw. or exp "Review Literature as Topic"/ or cochrane.ab. or cochrane.jw. or embase.ab. or medline.ab. or (psychlit or psyclit).ab. or (cinahl or cinhal).ab. or cancerlit.ab. or ((selection criteria or data extraction).ab. and "review"/)) not (Comment/ or Editorial/ or Letter/ or (animals/ not humans/)) (457917)  8 (exp clinical trial/ or randomized controlled trial/ or exp clinical trials as topic/ or randomized controlled trials as topic/ or Random Allocation/ or Double-Blind Method/ or Single-Blind Method/ or (clinical trial, phase i or clinical trial, phase ii or clinical trial, phase iii or clinical trial, phase iv or controlled clinical trial or randomized controlled trial or multicenter study or clinical trial).pt. or random*.ti,ab. or (clinic* adj trial*).tw. or ((singl* or doubl* or treb* or tripl*) adj (blind$3 or mask$3)).tw. or Placebos/ or placebo*.tw.) not (animals/ not humans/) (2010012)  9 Epidemiologic studies/ or case control studies/ or exp cohort studies/ or Controlled Before-After Studies/ or Case control.tw. or (cohort adj (study or studies)).tw. or Cohort analy$.tw. or (Follow up adj (study or studies)).tw. or (observational adj (study or studies)).tw. or Longitudinal.tw. or Retrospective*.tw. or prospective*.tw. or consecutive*.tw. or Cross sectional.tw. or Cross-sectional studies/ or historically controlled study/ or interrupted time series analysis/ [Onder exp cohort studies vallen ook longitudinale, prospectieve en retrospectieve studies] (3487543)  10 6 and 7 (11)  11 (6 and 8) not 10 (31)  12 (6 and 9) not (10 or 11) (91)  13 10 or 11 or 12 (133) |
| Cinahl | | **#** | **Query** | **Results** | | --- | --- | --- | | S5 | S1 AND (S2 OR S4) AND S3 | 123 | | S4 | (MH "Magnetic Resonance Imaging+") OR TI mri* OR TI nmr OR TI mra* OR TI fmri* OR AB mri* OR AB nmr OR AB mra* OR AB fmri* OR TI ('magnetic resonance' or mr) N2 (imag* or tomograph*)) OR AB ('magnetic resonance' or mr) N2 (imag* or tomograph*)) OR (MH "Ultrasonography+") OR TI ultraso* OR TI sonograph* OR TI echograph* OR TI utc OR AB ultraso* OR AB sonograph* OR AB echograph* OR AB utc | 292,719 | | S3 | (MH "Sensitivity and Specificity") OR (MH "Diagnostic Errors+") OR (MH "Predictive Value of Tests") OR (MH "Reproducibility of Results") OR (MH "Diagnosis, Differential") OR (MH "Reliability+") OR (MH "Validation Studies") OR TI sensitiv* OR TI specific* OR TI accuracy OR TI ppv OR TI npv OR TI 'predictive value' OR TI predict* OR TI 'roc curve' OR TI 'receiver operator' OR TI 'receiver operators' OR TI likelihood OR TI 'inter observer' OR TI 'intra observer' OR TI interobserver OR TI intraobserver OR TI validity OR TI kappa OR TI reliTIility OR TI reproducibility OR TI (test N2 're-test') OR TI (test N2 'retest') OR TI accuracy OR AB sensitiv* OR AB specific* OR AB accuracy OR AB ppv OR AB npv OR AB 'predictive value' OR AB predict* OR AB 'roc curve' OR AB 'receiver operator' OR AB 'receiver operators' OR AB likelihood OR AB 'inter observer' OR AB 'intra observer' OR AB interobserver OR AB intraobserver OR AB validity OR AB kappa OR AB reliability OR AB reproducibility OR AB (test N2 're-test') OR AB (test N2 'retest') OR AB accuracy | 1,262,432 | | S2 | (MH "Physical Examination+") OR (MH "Patient History Taking+") OR (TI anamnesis OR TI examination OR TI history taking OR TI medical interview*) OR (AB anamnesis OR AB examination OR AB history taking OR AB medical interview*) | 282,007 | | S1 | (MH "Patellar Tendinopathy") OR TI jumper s knee OR TI patella apicitis OR TI patellar apicitis OR TI patella apex syndrome OR TI patellar apex syndrome OR TI patella tip syndrome OR TI patellar tip syndrome OR TI patella tenosynovitis OR TI patellar tenosynovitis OR AB jumper s knee OR AB patella apicitis OR AB patellar apicitis OR AB patella apex syndrome OR AB patellar apex syndrome OR AB patella tip syndrome OR AB patellar tip syndrome OR AB patella tenosynovitis OR AB patellar tenosynovitis OR (((MH "Patellar Ligament") OR (MH "Patella") OR TI patella* OR AB patella*) AND ((MH "Tendinopathy+") OR TI tendinopath* OR AB tendinopath OR TI tendinos*s OR AB tendinos*s OR TI Tendinitis OR TI tendinitides OR TI tendonitis OR TI tendonitides OR TI peritendinitis OR TI paratenonitis OR AB Tendinitis OR AB tendinitides OR AB tendonitis OR AB tendonitides OR AB peritendinitis OR AB paratenonitis OR ((TI tendon OR AB tendon) AND (TI pathology OR AB pathology OR TI rupture* OR AB rupture*)))) | 1,029 | |
| PEDro | patella* jumper* tendino* [in titel]  → in body part field: lower leg & knee |

Table 4.2 Search strategy

|  | **Embase** | **OVID/MEDLINE** | **Cinahl** | **Deduplicated** |
| --- | --- | --- | --- | --- |
| SRs | 10 | 11 | x | 13 |
| RCT | 36 | 31 | x | 53 |
| Observational | 43 | 91 | x | 99 |
| Total | 89 | 133 | 123 | 229 |

Table 4.2 Search results

| **Author and year** | **Reason for exclusion** |
| --- | --- |
| Anuar, 2004 | Not available |
| Bode, 2017 | Describe prevalence of symptoms |
| Cassel, 2015 | Describe prevalence of symptoms for both patellar and achilles tendinopathy |
| Cook, 2001 | Patient population too young |
| Dirrichs, 2016 | Not specified for patellar tendinopathy patients |
| Dirrichs, 2018 | Not specified for patellar tendinopathy patients |
| Khan, 1999 | Do not describe diagnostic accuracy (describe correlation clinical outcomes and ultrasound/MRI before and after surgery) |
| Lian, 1996 | Do not describe diagnostic accuracy (describe correlation symptoms and ultrasound) |
| Ooi, 2016 | Present sensitivity, specificity and accuracy of grey scale US, PD imaging, US elastography instead of anamnesis and physical examination |
| Pfirrmann, 2008 | Do not describe diagnostic accuracy (describe correlation symptoms and ultrasound) |
| Ramos, 2009 | Unclear reference test |
| Rath, 2010 | Cadaveric study |
| Warden, 2007 | Present sensitivity, specificity and accuracy of MRI and grey scale US in symptomatic and asymptomatic patients |
| Weinberg, 1998 | Compares gray versus color sonography |

Table 4.3 Excluded studies

| **Study reference** | **Patient selection** | **Index test** | **Reference standard** | **Flow and timing** | **Comments with respect to applicability** |
| --- | --- | --- | --- | --- | --- |
| Maffulli, 2017 | Was a consecutive or random sample of patients enrolled?  Consecutive sample  Was a case-control design avoided?  No  Did the study avoid inappropriate exclusions?  Unclear | Were the index test results interpreted without knowledge of the results of the reference standard?  No  (One of the testers was aware of the diagnosis, the other was not.)  If a threshold was used, was it pre-specified?  No threshold | Is the reference standard likely to correctly classify the target condition?  Yes  Typical features of tendinopathy were a hypoechogenic area within the tendon, loss of the normal ribbon-like intratendinous echostructure, increased anteroposterior diameter greater than 50% compared to the asymptomatic controlateral tendon  Were the reference standard results interpreted without knowledge of the results of the index test?  Unclear  The 2 testers performed the tests in the same day, separately,  without communicating each other. The testers and the patients were not aware of US findings. | Was there an appropriate interval between index test(s) and reference standard?  Yes,  On the same day and after two weeks repeated.  Did all patients receive a reference standard?  Yes  Did patients receive the same reference standard?  Yes  Were all patients included in the analysis?  Yes, but they were included 4 times; results of 2 days and 2 testers | Are there concerns that the included patients do not match the review question?  Yes, not suspected patients but two different groups of patients  Are there concerns that the index test, its conduct, or interpretation differ from the review question?  No  Are there concerns that the target condition as defined by the reference standard does not match the review question?  No |
|  | CONCLUSION:  Could the selection of patients have introduced bias?  **RISK: HIGH** | CONCLUSION:  Could the conduct or interpretation of the index test have introduced bias?  **RISK: HIGH** | CONCLUSION:  Could the reference standard, its conduct, or its interpretation have introduced bias?  **RISK: UNCLEAR** | CONCLUSION  Could the patient flow have introduced bias?  **RISK: UNCLEAR** |  |
| Mendonca, 2016 | Was a consecutive or random sample of patients enrolled?  Unclear  Patients were recruited by telephone using the records of a previous preseason assessment at the university.  Was a case-control design avoided?  Yes  Did the study avoid inappropriate exclusions?  Yes  The exclusion criterion was having Osgood-Schlatter disease or Sinding-Larsen-Johansson syndrome, identified on ultrasound imaging. | Were the index test results interpreted without knowledge of the results of the reference standard?  Yes. All clinical measures were kept confidential by the principal investigator, who was blinded to the ultrasound examination results. All ultrasound assessments were performed by the same radiologist,  who was blinded to the results of the clinical tests. A third researcher performed the statistical analyses.  If a threshold was used, was it pre-specified?  No | Is the reference standard likely to correctly classify the target condition?  Yes  Were the reference standard results interpreted without knowledge of the results of the index test?  Yes  Radiologist performed all blinded  ultrasound assessments. | Was there an appropriate interval between index test(s) and reference standard?  Yes, 2 consecutive days  Did all patients receive a reference standard?  Yes  Did patients receive the same reference standard?  Yes  Were all patients included in the analysis?  Yes | Are there concerns that the included patients do not match the review question?  No  Are there concerns that the index test, its conduct, or interpretation differ from the review question?  No  Are there concerns that the target condition as defined by the reference standard does not match the review question?  No |
|  | CONCLUSION:  Could the selection of patients have introduced bias?  **RISK: UNCLEAR** | CONCLUSION:  Could the conduct or interpretation of the index test have introduced bias?  **RISK: LOW** | CONCLUSION:  Could the reference standard, its conduct, or its interpretation have introduced bias?  **RISK: LOW** | CONCLUSION  Could the patient flow have introduced bias?  **RISK: LOW** |  |

Table 4.4 Risk of bias assessment diagnostic accuracy studies (Quadas, 2011)

Judgments on risk of bias are dependent on the research question: some items are more likely to introduce bias than others, and may be given more weight in the final conclusion on the overall risk of bias per domain:

Patient selection:

- Consecutive or random sample has a low risk to introduce bias.
- A case control design is very likely to overestimate accuracy and thus introduce bias.
- Inappropriate exclusion is likely to introduce bias.

Index test:

- This item is similar to “blinding” in intervention studies. The potential for bias is related to the subjectivity of index test interpretation and the order of testing.
- Selecting the test threshold to optimise sensitivity and/or specificity may lead to overoptimistic estimates of test performance and introduce bias.

Reference standard:

- When the reference standard is not 100% sensitive and 100% specific, disagreements between the index test and reference standard may be incorrect, which increases the risk of bias.
- This item is similar to “blinding” in intervention studies. The potential for bias is related to the subjectivity of index test interpretation and the order of testing.

Flow and timing:

- If there is a delay or if treatment is started between index test and reference standard, misclassification may occur due to recovery or deterioration of the condition, which increases the risk of bias.
- If the results of the index test influence the decision on whether to perform the reference standard or which reference standard is used, estimated diagnostic accuracy may be biased.
- All patients who were recruited into the study should be included in the analysis, if not, the risk of bias is increased.

Judgement on applicability:

Patient selection: there may be concerns regarding applicability if patients included in the study differ from those targeted by the review question, in terms of severity of the target condition, demographic features, presence of differential diagnosis or co-morbidity, setting of the study and previous testing protocols.

Index test: if index tests methods differ from those specified in the review question there may be concerns regarding applicability.

Reference standard: the reference standard may be free of bias but the target condition that it defines may differ from the target condition specified in the review question.

# Summary of literature

Maffulli (2017) included fifteen consecutive athletes (n=15, mean age: 28 years, 95%CI, 25.81 to 30.99) with chronic PT (clinical and imaging diagnosis) in this case-control to evaluate manual palpation and the Royal London Hospital (RLH) test. The group of PT patients was compared to a matched group of patients with achilles tendinopathy (n=15, mean age: 27 years, 95%CI, 25.56 to 30.43).

Both the palpation test and RLH test were performed on both day 1 and day 14 with the patient supine and the knee extended, by two different testers (n=120 observations in total). The testers examined the patient separately on the same day without communicating with each other. One of the testers was aware of the diagnosis, the other was not. Palpation was performed gently, at the attachment site of the patellar tendon, over the inferior pole of the patella, and along its whole length, from proximal to distal. Patients were asked about tenderness on palpation.

The Royal London Hospital test was performed once local tenderness had been elicited palpating the tendon with the knee extended, the tender portion of the tendon was palpated again with the knee flexed to 90°. The test was considered positive if the pain was markedly reduced or absent in knee flexion. In asymptomatic tendons, the test was performed with the knee extended, selecting an area of the tendon 1 cm distal to the patellar insertion.

All patients received a high-resolution ultrasound assessment. The maximum tendon thickness was measured, and typical features of tendinopathy were recorded; a hypoechogenic area within the tendon, loss of the normal ribbon-like intratendinous echo structure, increased anteroposterior diameter greater than 50% compared to the asymptomatic contralateral tendon. Patients with PT (n=15) showed thickness of the patellar tendon and most of them showed a hypoechogenic area within the tendon (n=13).

Mendonca (2016) evaluated the diagnostic accuracy of the single leg declined squat and other clinical tests to identify patellar tendon abnormalities (PTA). Athletes (n=52) in sports with a high prevalence of PTA (volleyball, basketball, soccer, and running) were recruited by telephone using the records of a previous preseason assessment at the university. A total of 47 agreed to participate, four were excluded (presence of Osgood-Schlatter disease or Sinding-Larsen-Johansson syndrome on ultrasound). The cross-sectional study was conducted with 43 athletes (mean age 24.8 ± 6.7 years). The single leg decline squat is one repetition of a single-leg squat, performed barefoot on a 30°-decline ramp, from a position of knee extension to 30° of knee flexion. This test is used to evaluate tendon reactivity during the squat, using the presence of patellar tendon pain during the squat to indicate a positive test. All ultrasound assessments were performed by one radiologist with 12 years of experience, blinded to the clinical test results. Athletes were placed in supine position with the knees flexed and feet supported. The radiologist examined the entire extension of both patellar tendons on the longitudinal (sagittal) and transverse (axial) planes using a high-resolution linear-transducer ultrasound. A positive diagnosis of PTAs was given when a tendon had hypoechoic areas.

#

# Results Palpation

Specificity (critical)

The specificity of palpation was evaluated in one study (Maffulli, 2017). Maffulli (2017) showed a specificity of 94% of the palpation test with the ultrasound as reference test (n=52 observations). The palpation test resulted in 3 positive outcomes and 49 negative outcomes.

Level of evidence of the literature

The level of evidence regarding the specificity of the palpation test was downgraded by three levels because of study limitations; patients were not suspected from PT but were already diagnosed with PT or achilles tendinopathy (control group), further, it was unclear whether the results of the index tests were interpreted without knowledge of the reference test (-2, risk of bias), and due to the small number of included patients (-1, imprecision). The final level is very low.

Negative predictive value (critical)

The negative predictive value (NPV) of palpation was evaluated in one study (Maffulli, 2017). Maffulli (2017) presented a range of NPV depending on the prevalence of PT in the population, ranging from 94 to 99%. Calculating the NPV of the palpation test based on the True Negative (TN) / (TN + False Negative (FN)) a NPV of 49/(49+1)= 98% was found.

Level of evidence of the literature

The level of evidence regarding the negative predictive value of the palpation test was downgraded by three levels because of study limitations; patients were not suspected from PT but were already diagnosed with PT or achilles tendinopathy (control group), further, it was unclear whether the results of the index tests were interpreted without knowledge of the reference test (-2, risk of bias), and due to the small number of included patients (-1, imprecision). The final level is very low.

Sensitivity (important)

The sensitivity of palpation was evaluated in one study (Maffulli, 2017). Maffulli (2017) showed a sensitivity of 98% of the palpation test with the ultrasound as reference test (n=68 observations). The palpation test resulted in 67 positive outcomes in the PT patients and one negative outcome.

Level of evidence of the literature

The level of evidence regarding the sensitivity of the palpation test was downgraded by three levels because of study limitations; patients were not suspected from PT but were already diagnosed with PT or achilles tendinopathy (control group), further, it was unclear whether the results of the index tests were interpreted without knowledge of the reference test (-2, risk of bias), and due to the small number of included patients (-1, imprecision). The final level is very low.

Positive predictive value (important)

The positive predictive value (PPV) of palpation was evaluated in one study (Maffulli, 2017). Maffulli (2017) presented a range of PPV depending on the prevalence of PT in the population, ranging from 84 to 98%. Calculating the PPV of the palpation test based on the True Positive (TP) / (TP + False Positive (FP)) a PPV of 67/(67+3)= 96% was found.

Level of evidence of the literature

The level of evidence regarding the positive predictive value of the palpation test was downgraded by three levels because of study limitations; patients were not suspected from PT but were already diagnosed with PT or achilles tendinopathy (control group), further, it was unclear whether the results of the index tests were interpreted without knowledge of the reference test (-2, risk of bias), and due to the small number of included patients (-1, imprecision). The final level is very low.

# Conclusions

palpation test

Specificity (critical)

| **Very low**  **GRADE** | It is unclear whether the specificity of the palpation test is sufficient to exclude the diagnosis PT.  *Sources: (Maffulli, 2017)* |
| --- | --- |

Negative predictive value (critical)

| **Very low**  **GRADE** | It is unclear whether the negative predictive value of the palpation test is sufficient to exclude the diagnosis PT.  *Sources: (Maffulli, 2017)* |
| --- | --- |

Sensitivity (important)

| **Very low**  **GRADE** | It is unclear whether the sensitivity of the palpation test is sufficient to adequately diagnose PT.  *Sources: (Maffulli, 2017)* |
| --- | --- |

Positive predictive value (important)

| **Very low**  **GRADE** | It is unclear whether the positive predictive value of the palpation test is sufficient to prove the diagnosis PT.  *Sources: (Maffulli, 2017)* |
| --- | --- |

#

# Result Royal London Hospital test

Specificity (critical)

The specificity of the Royal London Hospital test was evaluated in one study (Maffuli, 2017). The Royal London Hospital test resulted in one positive outcome and 51 negative outcomes in the control group. Resulting is a specificity of 98% (n=52 observations).

Level of evidence of the literature

The level of evidence regarding the specificity of the Royal London Hospital test was downgraded by three levels because of study limitations; patients were not suspected from PT but were already diagnosed with PT or achilles tendinopathy (control group), further, it was unclear whether the results of the index tests were interpreted without knowledge of the reference test (-2, risk of bias), and due to the small number of included patients (-1, imprecision). The final level is very low.

Negative predictive value (critical)

The NPV of the Royal London Hospital test was evaluated in one study (Maffulli, 2017). Maffulli (2017), presented a range of NPV depending on the prevalence of PT in the population, ranging from 73 to 96%. Calculating the NPV of the Royal London Hospital test based on the TN/(TN+FN) a NPV of 51/ (51+8) = 86% was found.

Level of evidence of the literature

The level of evidence regarding the NPV of the Royal London Hospital test was downgraded by three levels because of study limitations; patients were not suspected from PT but were already diagnosed with PT or achilles tendinopathy (control group), further, it was unclear whether the results of the index tests were interpreted without knowledge of the reference test (-2, risk of bias), and due to the small number of included patients (-1, imprecision). The final level is very low.

Sensitivity (important)

The sensitivity of the Royal London Hospital test was evaluated in one study (Maffuli, 2017), that reported a sensitivity of 88% (n=68 observations). The Royal London Hospital test resulted in 60 positive outcomes in the PT patients and 8 negative outcomes.

Level of evidence of the literature

The level of evidence regarding the sensitivity of the Royal London Hospital test was downgraded by three levels because of study limitations; patients were not suspected from PT but were already diagnosed with PT or achilles tendinopathy (control group), further, it was unclear whether the results of the index tests were interpreted without knowledge of the reference test (-2, risk of bias), and due to the small number of included patients (-1, imprecision).The final level is very low.

Positive predictive value (important)

The PVV of the Royal London Hospital test was evaluated in one study (Maffulli, 2017). Maffulli (2017), presented a range of PPV depending on the prevalence of PT in the population, ranging from 94 to 99%. Calculating the PPV of the Royal London Hospital test based on the TP/(TP+FP) a PPV of 60 / (60+1) = 98% was found.

Level of evidence of the literature

The level of evidence regarding the PVV of the Royal London Hospital test was downgraded by three levels because of study limitations; patients were not suspected from PT but were already diagnosed with PT or achilles tendinopathy (control group), further, it was unclear whether the results of the index tests were interpreted without knowledge of the reference test (-2, risk of bias), and due to the small number of included patients (-1, imprecision). The final level is very low.

# Conclusions

Specificity (critical)

| **Very low**  **GRADE** | It is unclear whether the specificity of the Royal London Hospital test is sufficient to exclude the diagnosis PT.  *Sources: (Maffulli, 2017)* |
| --- | --- |

Negative predictive value (critical)

| **Very low**  **GRADE** | It is unclear whether the negative predictive value of the Royal London Hospital test is sufficient to exclude the diagnosis PT.  *Sources: (Maffulli, 2017)* |
| --- | --- |

Sensitivity (important)

| **Very low**  **GRADE** | It is unclear whether the sensitivity of the Royal London Hospital test is sufficient to adequately diagnose PT.  *Sources: (Maffulli, 2017)* |
| --- | --- |

Positive predictive value (important)

| **Very low**  **GRADE** | It is unclear whether the positive predictive value of the Royal London Hospital test is sufficient to prove the diagnosis PT.  *Sources: (Maffulli, 2017)* |
| --- | --- |

#

# Result Single leg decline squat

Specificity (critical)

The specificity of the single leg decline squat was calculated from the data presented in one study (Mendonca, 2016). The single leg decline squat resulted in 6 negative outcomes and 23 negative outcomes on the ultrasound, providing a specificity of 79%.

Level of evidence of the literature

The level of evidence regarding the specificity of the single leg decline squat was downgraded by three levels because of the small number of included patients (-2, imprecision), and it is unknown whether the participants were suspected of patellar tendon abnormalities (-1, risk of bias). The final level is very low.

Negative predictive value (critical)

The NPV of the single leg decline squat was calculated from the data presented in one study (Mendonca, 2016). Calculating the NPV of the single leg decline squat test based on the TN/(TN+FN) a NPV of 85% was found.

Level of evidence of the literature

The level of evidence regarding the negative predictive value of the single leg decline squat was downgraded by three levels because of the small number of included patients (-2, imprecision), and it is unknown whether the participants were suspected of patellar tendon abnormalities (-1, risk of bias). The final level is very low.

Sensitivity (important)

The sensitivity of the single leg decline squat was calculated from the data presented in one study (Mendonca, 2016). The single leg decline squat resulted in 10 positive outcomes and 4 negative outcomes on the ultrasound, providing a sensitivity of 71%.

Level of evidence of the literature

The level of evidence regarding the sensitivity of the single leg decline squat was downgraded by three levels because of the small number of included patients (-2, imprecision), and it is unknown whether the participants were suspected of patellar tendon abnormalities (-1, risk of bias). The final level is very low.

Positive predictive value (important)

The PVV of the single leg decline squat was calculated from the data presented in one study (Mendonca, 2016). Calculating the PPV of the single leg decline squat based on the TP/(TP+FP) a PPV of 63% was found.

Level of evidence of the literature

The level of evidence regarding the positive predictive value of the single leg decline squat was downgraded by three levels because of the small number of included patients (-2, imprecision), and it is unknown whether the participants were suspected of patellar tendon abnormalities (-1, risk of bias). The final level is very low.

# Conclusions

Specificity (critical)

| **Very low**  **GRADE** | It is unclear whether the specificity of the single leg decline squat is sufficient to exclude the diagnosis PT.  *Sources: (Mendonca, 2016)* |
| --- | --- |

Negative predictive value(critical)

| **Very low**  **GRADE** | It is unclear whether the negative predictive value of the single leg decline squat is sufficient to exclude the diagnosis PT.  *Sources: (Mendonca, 2016)* |
| --- | --- |

Sensitivity (important)

| **Very low**  **GRADE** | It is unclear whether the sensitivity of the single leg decline squat is sufficient to adequately diagnose PT.  *Sources: (Mendonca, 2016)* |
| --- | --- |

Positive predictive value (important)

| **Very low**  **GRADE** | It is unclear whether the positive predictive value of the single leg decline squat is sufficient to prove the diagnosis PT.  *Sources: (Mendonca, 2016)* |
| --- | --- |

# Result History

No studies evaluated the specificity, negative predictive value, sensitivity, or positive predictive value of the history.

Level of evidence of the literature

The level of evidence regarding specificity, negative predictive value, sensitivity, or positive predictive value of the history was not graded due to lack of studies.

# Conclusions

| **-**  **GRADE** | No conclusions could be drawn about the history due to lack of studies. |
| --- | --- |

Expectations and values of patients (and/or their caretakers)

In the questionnaire administered to patients (and/or their caretakers), patients indicated the value imaging quite high, even if the outcome does not affect further treatment or recovery time. For confirming or excluding the diagnosis of PT, ultrasound is the most appropriate and accessible examination. For the demonstration or exclusion of intra-articular disorders, MRI is the appropriate examination. It should be kept in mind that MRI is many times more expensive than ultrasound, and less accessible.

Cost

In a large percentage of cases the cost of additional imaging must be paid by the patient. The added value for treatment and recovery time is limited. Whether these costs outweigh the (limited) health benefits must be considered per patient.

# Considerations – from evidence to recommendation

Based on the literature, it is unclear what the diagnostic accuracy of history and physical examination is, 2 studies were included (very low grade). Due to the lack of evidence, the recommendations are mainly based on consensus (ICON2019) and expert opinion. There are insufficient data to make a valid statement about the diagnostic accuracy of the history and physical examination for the diagnosis of PT. However, history and physical examination are leading in diagnosis of PT and differentiating between many other conditions. It is fundamental in the diagnosis and should always be applied, despite the lack of high quality scientific evidence. The general anamnestic criterion; “load-dependent pain at the lower pole of the patella” is the most obvious. For physical examination, 'the palpation test’, the ‘Royal London hospital test’ and the ‘single leg decline squat' all appear to have approximately the same very low level of evidence. Because of this and due to the lack of alternatives, the expert panel recommends all three, possibly in combination, for diagnosis. In case of doubt about the diagnosis or treatment-resistant symptoms, additional imaging can be considered. Consideration of the added value depends on the patient's and/or practitioner's need for certainty about the diagnosis and knowledge of the radiologist.

# Rationale of the recommendations

There are insufficient data to make a well-founded statement about the diagnostic accuracy of history and physical examination for diagnosing PT. Imaging to confirm the diagnosis of PT has limited value on medical grounds, but could be perceived as valuable by patients. In cases of doubt about the diagnosis or therapy-resistant symptoms, additional imaging may be considered. The Dutch health care system provides basic health care insurance including excess (‘eigen risico’). Therefore, the costs for additional imaging assessments are for the patient. The most appropriate and accessible examination for assessing the patellar tendon is ultrasound. The waiting time for additional imaging is relatively limited compared to the duration of the symptoms. Consideration of added value depends on the need for certainty of diagnosis on the part of patient and/or practitioner.

Have the additional imaging examination performed and reviewed by the person with the highest possible qualification for performing this particular examination.

Consider the following competencies when applying imaging:

- The person performing the imaging is able to critically consider the added value of the imaging technique. The application of the technique should be clinically relevant to the patient.

- The person performing and assessing the imaging has sufficient education and experience. Regular continuing education and peer review is recommended to maintain and renew knowledge.

- The person communicating the results of the imaging has adequate knowledge of the clinical picture and the relationship between imaging findings and the outcome of PT.

# References

Breda, S. J., Oei, E. H., Zwerver, J., Visser, E., Waarsing, E., Krestin, G. P., & de Vos, R. J. (Epub ahaed of print). Effectiveness of progressive tendon-loading exercise therapy in patients with patellar tendinopathy: a randomised clinical trial. British Journal of Sports Medicine;55(9):501-509.

Lee WC, Ng GY, Zhang ZJ, Malliaras P, Masci L, Fu SN. (2020). Changes on Tendon Stiffness and Clinical Outcomes in Athletes Are Associated With Patellar Tendinopathy After Eccentric Exercise. *Clin J Sport Med*;30(1):25-32.

Maffulli N., Oliva F., Loppini M., Aicale R., Spiezia F., King JB. (2017) The Royal London Hospital Test for the clinical diagnosis of patellar tendinopathy. *Muscles Ligaments Tendons J, 7*(2):315-322.

Mendonça Lde M., Ocarino J.M., Bittencourt N.F., Fernandes L.M., Verhagen E., Fonseca ST. (2016) The Accuracy of the VISA-P Questionnaire, Single-Leg Decline Squat, and Tendon Pain History to Identify Patellar Tendon Abnormalities in Adult Athletes. *J Orthop Sports Phys Ther, 46*(8):673-80.

Thijs KM, Zwerver J, Backx FJ, Steeneken V, Rayer S, Groenenboom P, Moen MH. (2017). Effectiveness of Shockwave Treatment Combined With Eccentric Training for Patellar Tendinopathy: A Double-Blinded Randomized Study. *Clin J Sport Med*;27(2):89-96.

van Ark, M., Cook, J., Docking, S., Zwerver, J., Gaida, J., van den Akker‐Scheek, I., & Rio, E. (2016). Do isometric and isotonic exercise programs reduce pain in athletes with patellar tendinopathy in‐season? A randomised clinical trial. *Journal of Science and Medicine in Sport*, 19(9), 702–706.

van Ark M, Rio E, Cook J, van den Akker-Scheek I, Gaida JE, Zwerver J, Docking S. (2018) Clinical Improvements Are Not Explained by Changes in Tendon Structure on Ultrasound Tissue Characterization After an Exercise Program for Patellar Tendinopathy. *Am J Phys Med Rehabil*.;97(10):708-714.

Visnes, H., Hoksrud, A., Cook, J., & Bahr, R. (2005). No effect of eccentric training on jumper's knee in volleyball players during the competitive season: A randomized clinical trial. *Scandinavian Journal of Medicine & Science in Sports*, 16(3), 227–234.

Warden SJ, Metcalf BR, Kiss ZS, Cook JL, Purdam CR, Bennell KL, Crossley KM. (2008). Low-intensity pulsed ultrasound for chronic patellar tendinopathy: a randomized, double-blind, placebo-controlled trial. *Rheumatology (Oxford)*;47(4):467-71.

**Z**werver J, Hartgens F, Verhagen E, van der Worp H, van den Akker-Scheek I, Diercks RL. (2011). No effect of extracorporeal shockwave therapy on patellar tendinopathy in jumping athletes during the competitive season: a randomized clinical trial. *Am J Sports Med*;39(6):1191-9.

# Construction of Module 5 ‘Exercise therapy for PT’

Scoping Question: What is the optimal form of exercise therapy for patients with PT?

# Search and select

A systematic review of the literature was performed to answer the following question: What is the effectiveness of exercise therapy in patients with PT when compared to other types of exercise therapy or a control group (standard care/ placebo/ wait and see policy) on pain, function, return to sport/ work, duration of absenteeism, patient satisfaction, and patient recovery?

P: patients with PT (adults)

I: exercise therapy

C: other type of exercise therapy or control group/ standard care/ placebo/ wait and see policy

O: pain, function, return to sport/ work, duration of absenteeism, patient satisfaction, and patient recovery

Relevant outcome measures

The guideline development expert panel considered pain and function as critical outcome measures for decision making; and return to sport/ work, duration of absenteeism, patient satisfaction, and patient recovery as important outcome measures for decision making.

For the outcome pain the Visual Analogue Scale (VAS) and the Numeric Rating Scale (NRS) were used. The VISA-P score is a questionnaire developed to measure pain and function in patients with PT. Return to sport/ work was measured with the Tegner score. Satisfaction with the result of treatment and recovery were usually measured on a Likert scale.

The expert panel defined a difference of 2 cm (of 10 cm) on the VAS or 2 points on the NRS score as a minimal clinically (patient) important difference, in line with Crossley (2004). A difference of 13 points on the VISA-P score is seen as a minimal clinically important difference (Hernandez-Sanchez 2014). A minimal clinically important difference for return to sport/ work, duration of absenteeism, patient satisfaction and patient recovery was not predefined.

Search and select (Methods)

The databases Pubmed and Embase (via Embase.com) were searched with relevant search terms until 29-04-2020. The detailed search strategy is depicted in the tables. The systematic literature search resulted in 316 hits. Studies were selected based on the following criteria: systematic reviews (searched in at least two databases, and detailed search strategy, risk of bias assessment and results of the individual studies available) and randomized controlled trials (RCTs) that included at least 20 patients with PT, compared exercise therapy with another exercise therapy or a control condition, and included at least one of the defined outcome measures. Nine RCT studies were initially selected based on title and abstract screening, seven of these RCT studies were part of a systematic review. The systematic review (Lim, 2018) provided an overview of the effects of isometric, eccentric and heavy slow resistance (HSR) exercises on pain and function in patients with PT, however it did not provide a meta-analysis of the included studies. Therefore, the systematic review was excluded. From the seven individual RCTs, three studies were excluded due to the small study population (n< 20) (see exclusion table). One study of two the initially selected RCT studies was excluded because it was written in Spanish (Table 5.4).

Results

Five RCT studies were included in the analysis of the literature. Of these five articles, one study was about isometric and isotonic (heavy slow resistance) training (van Ark, 2016), and four studies were about eccentric training (Frohm, 2007; Kongsgaard, 2009; Stasinopoulos, 2012; Visnes, 2005). Important study characteristics and results are summarized in the evidence table. The assessment of the risk of bias is summarized in the risk of bias table.

Key articles: 19793213 OR 23494258 OR 26707957 OR 29972281

| **Search** | **Query** | **Items found** |
| --- | --- | --- |
| #12 | Search (#8 OR #9 OR #10) AND #11 | 4 |
| #11 | Search 19793213 23494258 26707957 29972281[uid] | 4 |
| #10 | Search #7 AND #3 | 200 |
| #9 | Search #7 AND #2 | 76 |
| #8 | Search #7 AND #1 | 35 |
| #7 | Search #6 AND ("2000/01/01"[PDAT] : "2030/12/31"[PDAT]) | 361 |
| #6 | Search #4 AND #5 | 418 |
| #5 | Search ("Exercise Therapy"[Mesh] OR "Exercise"[tiab] OR "exercises"[tiab] OR Physiotherap*[tiab] OR physical therap*[tiab] OR "resistance training"[tiab] OR (("strengthen"[tiab] OR "strengthening"[tiab] OR "training"[tiab] OR "eccentric"[tiab] OR "isometric"[tiab] OR "concentric"[tiab] OR "isotonic"[tiab]) AND ("hip"[tiab] OR "hips"[tiab] OR "knee"[tiab] OR "knees"[tiab] OR "gluteal"[tiab] OR "gluteus"[tiab] OR "quadriceps"[tiab] OR "vastus medialis"[tiab])) OR "Mensendieck"[tiab] OR "Cesar therapy"[tiab] OR "pilates"[tiab] OR "squat"[tiab] OR "squats"[tiab] OR "squatting"[tiab]) | 354906 |
| #4 | Search ("jumper s knee"[tiab] OR "patella apicitis"[tiab] OR "patellar apicitis"[tiab] OR "patella apex syndrome"[tiab] OR "patellar apex syndrome"[tiab] OR "patella tip syndrome"[tiab] OR "patellar tip syndrome"[tiab] OR "patella tenosynovitis"[tiab] OR "patellar tenosynovitis"[tiab] OR (("Patellar Ligament"[Mesh] OR "Patella"[Mesh] OR "Patella"[tiab] OR "patellar"[tiab]) AND ("Tendinopathy"[Mesh:NoExp] OR "Tendinopathy"[tiab] OR "Tendinopathies"[tiab] OR "tendinosis"[tiab] OR "tendinoses"[tiab] OR "tendinitis"[tiab] OR "Tendinitides"[tiab] OR "tendonitis"[tiab] OR "Tendonitides"[tiab] OR "peritendinitis"[tiab] OR "paratenonitis"[tiab] OR ("tendon"[tiab] AND ("pathology"[tiab] OR "rupture"[tiab] OR "ruptures"[tiab]))))) | 2121 |
| #3 | Search ("cohort studies"[mesh] OR "case-control studies"[mesh] OR "comparative study"[pt] OR "risk factors"[mesh] OR "cohort"[tw] OR "compared"[tw] OR "groups"[tw] OR "case control"[tw] OR "multivariate"[tw]) | 7854601 |
| #2 | Search ((random*[tiab] AND (controlled[tiab] OR control[tiab] OR placebo[tiab] OR versus[tiab] OR vs[tiab] OR group[tiab] OR groups[tiab] OR comparison[tiab] OR compared[tiab] OR arm[tiab] OR arms[tiab] OR crossover[tiab] OR cross-over[tiab]) AND (trial[tiab] OR study[tiab])) OR ((single[tiab] OR double[tiab] OR triple[tiab]) AND (masked[tiab] OR blind*[tiab]))) | 726105 |
| #1 | Search ("Meta-Analysis as Topic"[Mesh] OR “Meta-Analysis”[Publication Type] OR metaanaly*[tiab] OR metanaly*[tiab] OR meta-analy*[tiab] OR meta synthes*[tiab] OR metasynthes*[tiab] OR meta ethnograph*[tiab] OR metaethnograph*[tiab] OR meta summar*[tiab] OR metasummar*[tiab] OR meta-aggregation[tiab] OR metareview[tiab] OR meta-review[tiab] OR overview of reviews[tiab] OR ((systematic*[ti] OR scoping[ti] OR umbrella[ti] OR meta-narrative[ti] OR metanarrative[ti] OR evidence based[ti]) AND (review*[ti] OR overview*[ti])) OR ((evidence[ti] OR narrative[ti] OR metanarrative[ti] OR qualitative[ti]) AND synthesis[ti]) OR systematic review[pt] OR prisma[tiab] OR preferred reporting items[tiab] OR quadas*[tiab] OR systematic review*[tiab] OR systematic literature[tiab] OR structured literature search[tiab] OR systematic overview*[tiab] OR scoping review*[tiab] OR umbrella review*[tiab] OR mapping review*[tiab] OR systematic mapping[tiab] OR evidence synthes*[tiab] OR narrative synthesis[tiab] OR metanarrative synthesis[tiab] OR research synthesis[tiab] OR qualitative synthesis[tiab] OR realist synthesis[tiab] OR realist review[tiab] OR realist evaluation[tiab] OR systematic qualitative review[tiab] OR mixed studies review[tiab] OR mixed methods synthesis[tiab] OR mixed research synthesis[tiab] OR quantitative literature review[tiab] OR systematic evidence review[tiab] OR evidence-based review[tiab] OR comprehensive literature search[tiab] OR integrated review*[tiab] OR integrated literature review[tiab] OR integrative review*[tiab] OR integrative literature review*[tiab] OR structured literature review*[tiab] OR systematic search and review[tiab] OR meta-narrative review*[tiab] OR metanarrative review[tiab] OR systematic narrative review[tiab] OR systemic review[tiab] OR systematized review[tiab] OR systematic research synthesis[tiab] OR bibliographic*[tiab] OR hand-search*[tiab] OR handsearch*[tiab] OR manual search*[tiab] OR searched manually[tiab] OR manually searched[tiab] OR journal database*[tiab] OR review authors independently[tiab] OR reviewers independently[tiab] OR independent reviewers[tiab] OR independent review authors[tiab] OR electronic database search*[tiab] OR (study selection[tiab] AND data extraction[tiab]) OR (selection criteria[tiab] AND data collection[tiab]) OR (selection criteria[tiab] AND data analysis[tiab]) OR (evidence acquisition[tiab] AND evidence synthesis[tiab]) OR (pubmed[tiab] AND embase[tiab]) OR (medline[tiab] AND embase[tiab]) OR (pubmed[tiab] AND cochrane[tiab]) OR (medline[tiab] AND cochrane[tiab]) OR (embase[tiab] AND cochrane[tiab]) OR (pubmed[tiab] AND psycinfo[tiab]) OR (medline[tiab] AND psycinfo[tiab]) OR (embase[tiab] AND psycinfo[tiab]) OR (cochrane[tiab] AND psycinfo[tiab]) OR (pubmed[tiab] AND web of science[tiab]) OR (medline[tiab] AND web of science[tiab]) OR (embase[tiab] AND web of science[tiab]) OR (psycinfo[tiab] AND web of science[tiab]) OR (cochrane[tiab] AND web of science[tiab]) OR ((literature[ti] OR qualitative[ti] OR quantitative[ti] OR integrated[ti] OR integrative[tiab] OR rapid[ti] OR short[ti] OR critical*[ti] OR mixed stud*[ti] OR mixed method*[ti] OR focused[ti] OR focussed[ti] OR structured[ti] OR comparative[ti] OR comparitive[ti] OR evidence[ti] OR comprehensive[ti] OR realist[ti]) AND (review*[ti] OR overview*[ti]) AND (literature search[tiab] OR structured search[tiab] OR electronic search[tiab] OR search strategy[tiab] OR gray literature[tiab] OR grey literature[tiab] OR Review criteria[tiab] OR eligibility criteria[tiab] OR inclusion criteria[tiab] OR exclusion criteria[tiab] OR predetermined criteria[tiab] OR included studies[tiab] OR identified studies[tiab] OR (systematic search[tiab] AND literature[tiab]) OR strength of evidence[tiab] OR citation*[tiab] OR references[tiab] OR database search*[tiab] OR electronic database*[tiab] OR data base search*[tiab] OR electronic data-base*[tiab] OR search criteria[tiab] OR study selection[tiab] OR data extraction[tiab] OR methodological quality[tiab] OR methodological characteristics[tiab] OR methodologic quality[tiab] OR methodologic characteristics[tiab])) OR ((literature review[tiab] OR literature search*[tiab]) AND (structured search[tiab] OR electronic search[tiab] OR Search strategy[tiab] OR gray literature[tiab] OR grey literature[tiab] OR review criteria[tiab] OR eligibility criteria[tiab] OR inclusion criteria[tiab] OR exclusion criteria[tiab] OR predetermined criteria[tiab] OR included studies[tiab] OR identified studies[tiab] OR (systematic search[tiab] AND literature[tiab]) OR strength of evidence[tiab] OR citation*[tiab] OR references[tiab] OR database search*[tiab] OR electronic database*[tiab] OR data base search*[tiab] OR electronic data-base*[tiab] OR search criteria[tiab] OR study selection[tiab] OR data extraction[tiab] OR methodological quality[tiab] OR methodological characteristics[tiab] OR methodologic quality[tiab] OR methodologic characteristics[tiab]))) NOT ("Comment" [Publication Type] OR "Letter" [Publication Type]) NOT (“Animals”[Mesh] NOT “Humans”[Mesh]) | 341721 |

Table 5.1 Ovid/medline search

| **No.** | **Query** | **Results** |
| --- | --- | --- |
| #11 | #8 AND #3 | **123** |
| #10 | #8 AND #2 | **138** |
| #9 | #8 AND #1 | **48** |
| #8 | #7 AND [2000-2030]/py | **509** |
| #7 | #6 NOT ('conference abstract'/it OR 'editorial'/it OR 'letter'/it OR 'note'/it) NOT (('animal experiment'/exp OR 'animal model'/exp OR 'nonhuman'/exp) NOT 'human'/exp) | **596** |
| #6 | #4 AND #5 | **723** |
| #5 | 'kinesiotherapy'/exp OR 'physiotherapy'/exp OR 'exercise':ti,ab,kw OR 'exercises':ti,ab,kw OR 'physiotherap*':ti,ab,kw OR 'physical therap*':ti,ab,kw OR 'resistance training'/de OR 'resistance training':ti,ab,kw OR (('strengthen':ti,ab,kw OR 'strengthening':ti,ab,kw OR 'training':ti,ab,kw OR 'eccentric':ti,ab,kw OR 'isometric':ti,ab,kw OR 'concentric':ti,ab,kw OR 'isotonic':ti,ab,kw) AND ('hip':ti,ab,kw OR 'hips':ti,ab,kw OR 'knee':ti,ab,kw OR 'knees':ti,ab,kw OR 'gluteal':ti,ab,kw OR 'gluteus':ti,ab,kw OR 'quadriceps':ti,ab,kw OR 'vastus medialis':ti,ab,kw)) OR 'mensendieck':ti,ab,kw OR 'cesar therapy':ti,ab,kw OR 'pilates':ti,ab,kw OR 'squat':ti,ab,kw OR 'squats':ti,ab,kw OR 'squatting':ti,ab,kw | **548302** |
| #4 | 'patellar tendinopathy'/de OR 'jumper s knee':ti,ab,kw OR 'patella apicitis':ti,ab,kw OR 'patellar apicitis':ti,ab,kw OR 'patella apex syndrome':ti,ab,kw OR 'patellar apex syndrome':ti,ab,kw OR 'patella tip syndrome':ti,ab,kw OR 'patellar tip syndrome':ti,ab,kw OR 'patella tenosynovitis':ti,ab,kw OR 'patellar tenosynovitis':ti,ab,kw OR (('patellar ligament'/de OR 'patella'/de OR 'patella':ti,ab,kw OR 'patellar':ti,ab,kw) AND ('tendinitis'/exp OR 'tendinopathy':ti,ab,kw OR 'tendinopathies':ti,ab,kw OR 'tendinosis':ti,ab,kw OR 'tendinoses':ti,ab,kw OR 'tendinitis':ti,ab,kw OR 'tendinitides':ti,ab,kw OR 'tendonitis':ti,ab,kw OR 'tendonitides':ti,ab,kw OR 'peritendinitis':ti,ab,kw OR 'paratenonitis':ti,ab,kw OR ('tendon':ti,ab,kw AND ('pathology':ti,ab,kw OR 'rupture':ti,ab,kw OR 'ruptures':ti,ab,kw)))) | **2795** |
| #3 | 'major clinical study'/de OR 'clinical study'/de OR 'case control study'/de OR 'family study'/de OR 'longitudinal study'/de OR 'retrospective study'/de OR 'prospective study'/de OR 'cohort analysis'/de OR ((cohort NEAR/1 (study OR studies)):ab,ti) OR (('case control' NEAR/1 (study OR studies)):ab,ti) OR (('follow up' NEAR/1 (study OR studies)):ab,ti) OR (observational NEAR/1 (study OR studies)) OR ((epidemiologic NEAR/1 (study OR studies)):ab,ti) OR (('cross sectional' NEAR/1 (study OR studies)):ab,ti) | **5223335** |
| #2 | 'clinical trial'/exp OR 'randomization'/exp OR 'single blind procedure'/exp OR 'double blind procedure'/exp OR 'crossover procedure'/exp OR 'placebo'/exp OR 'prospective study'/exp OR rct:ab,ti OR random*:ab,ti OR 'single blind':ab,ti OR 'randomised controlled trial':ab,ti OR 'randomized controlled trial'/exp OR placebo*:ab,ti | **3028069** |
| #1 | 'meta analysis'/de OR cochrane:ab OR embase:ab OR psycinfo:ab OR cinahl:ab OR medline:ab OR ((systematic NEAR/1 (review OR overview)):ab,ti) OR ((meta NEAR/1 analy*):ab,ti) OR metaanalys*:ab,ti OR 'data extraction':ab OR cochrane:jt OR 'systematic review'/de | **493574** |

Table 5.2 Embase search

|  | **EMBASE** | **PubMed** | **Deduplicated** |
| --- | --- | --- | --- |
| SRs | 48 | 35 | 56 |
| RCTs | 138 | 76 | 126 |
| Observationele studies | 123 | 200 | 134 |
| Other |  |  |  |
| Total | 309 | 311 | 316 |

Table 5.3 Search results

| **Author and year** | **Reason for exclusion** |
| --- | --- |
| Cannell, 2001 | Small study population (n<20) |
| Jonsson, 2005 | Small study population (n<20) |
| Lim, 2018 | Review without meta-analysis, studies were individually reviewed |
| Rosety-Rodriquez, 2006 | Language Spanish |
| Young, 2004 | Small study population (n<20) |

Table 5.4 Excluded studies

# Summary of literature

Frohm (2007) described a pilot randomized clinical trial (RCT) with 20 athletes with PT. Athletes were included in the study if they had the clinical diagnosis of PT verified by MRI or ultrasound imaging, and symptoms for at least 3 months. Participants were randomized in two groups: the group performing eccentric overload training with the Bromsman device (n=11) or the standard eccentric training group using a decline board (n=9). The sessions for both groups consisted of a standardized warming up, eccentric training alternated with trunk and foot stability training and each session was rounded off with stretching exercises of the quadriceps and hamstrings. The eccentric overload training group used the Bromsman device, which consists of a barbell (320 kg) suspended from wires that can be moved up and down along a chosen distance and at a preset speed by a hydraulic machine. The descending distance was individually set from a standing straight position to approximately 110°of knee flexion, and the speed was set to 0.11 m/s. The patients resisted the movement of the barbell using both legs: 4 sets of 4 repetitions (initial set for warm‐up, then maximal effort for next 3 sets). The other group performed unilateral squats on a 25° decline board with 3 sets of 15 repetitions, and daily home exercises (3 sets of 15 reps/day). If the VAS score was <3 for a set, the load was increased in 5 kg increments, and if the VAS exceeded >5, the load was reduced. The principal investigator supervised all the training sessions for all patients in both groups at the clinic. The effectiveness of the training was evaluated with the VISA-P score after 12 weeks. Secondary outcome measures were isokinetic muscle torque, dynamic function and muscle flexibility, as well as pain level estimations using visual analogue scale (VAS).

The RCT of Kongsgaard (2009) consisted of three arms: eccentric decline squat training (n=13), heavy slow resistance training (n=13), and peritendinous corticosteroid injections (n=13). Corticosteroid injections are not included in the current search question, and this study condition is therefore excluded from this summary. The eccentric training group performed three sets of 15 slow repetitions of eccentric unilateral squats on a 25^o^ decline board, twice a day for 12 weeks. Once a week the participants had a supervised training. Load was increased as pain diminished, using a backpack with increasing load. The heavy slow resistance (HSR) training consisted of three weekly sessions, including one supervised session. Every training consisted of four sets of squats, leg press and hack squats. All exercises were performed from extension to 90^o^ knee flexion, and patients were instructed to perform the exercise in 6 seconds per repetition (3s in eccentric phase and 3s in concentric phase). All participants could perform their sporting activities during the intervention period, if these activities could be performed with a maximum VAS score of 30 (range 0-100). All participants performed at least 75% of the sessions, the average compliance in the eccentric group was 89 ± 8% and in the HSR group 91 ± 5%. The effectiveness of the interventions was evaluated after 12 weeks intervention and after six months with the VISA-P and the VAS, further satisfaction was measured as “satisfied” or “not satisfied”.

Stasinopoulos (2012) compared eccentric training and stretching (n=22) with eccentric training alone (n=21). They included patients who had PT for at least 3 months. The training consisted of unilateral squats on a 25^o^ decline board. The squat was performed at a slow speed in three sets of 15 repetitions. The patients were told to go ahead with the exercise even if they experienced mild pain. When the squat was pain-free, the load was increased by holding weights in their hands. The group with stretching exercises performed static stretching of quadriceps and hamstrings before and after the eccentric training, each stretch lasted 30 seconds and there was a one-minute rest between each stretch. The training was performed five times per week for four weeks and was individualized based on the patient’s description of pain experienced during the training. The effectiveness of the training was evaluated with the VISA-P score after four and 24 weeks.

The randomized clinical trial of Van Ark (2015) described the effects of isometric and isotonic training in jumping athletes with PT. The isometric group (n=13) used the leg extension machine to perform five sets of 45s isometric contractions of each leg hold at knee angle 60° flexion at 80% MVC. The isotonic group (n=16) used the leg extension machine to perform four sets of eight repetitions, with 3s concentric phase immediately followed by 4s eccentric phase per repetition performed on 80% of 8RM. After performing the exercises for each leg, all participants rested for 15s before continuing with the first leg again. Weight was increased by 2.5% every week if possible. The program was demonstrated (including repetition maximum testing) at the gym where they were going to perform their exercises. Every week participants were followed-up in person or by phone, asking participants if they encountered any problems with the exercise program. The intervention consisted of four sessions per week for four weeks. The median number of sessions performed per week (compliance) in the isometric group was 3 (IQR 2.5-3.9) and in the isotonic group was 3 (IQR2.75-3.75). The effectiveness of the training was evaluated measuring pain during a single leg decline squat (SLDS) scored on a numeric rating scale (NRS), besides the VISA-P score was used to evaluate pain and function of the knee after four weeks.

Visnes (2005) conducted a RCT with volleyball players with patellar and/or quadriceps tendinopathy for at least three months and a VISA-P score <80. A total of 29 patients participated, 24 of them had PT, 2 patients had quadriceps tendinopathy only, and 3 patients had both quadriceps and PT. The participants were randomized to a training group (n=13) or a control group (n=16). The training consisted of eccentric home exercises (squats) on a 25° decline board. The squat went to 90° of knee flexion, which ensured that the subjects went past 60° of knee flexion, to place maximal load on the patellar tendon. If pain on the VAS score was <3 or 4 extra weight was recommended, the load was increased in 5 kg increments, and if VAS exceeded 6 or 7, it was recommended to reduce the load. Only 6 of the 13 players in this group did exercises with an additional external load, and the final load these players reported having used was 4.2 ± 4.9 kg. Patients were followed up by telephone when the training period started, and the training program was described and discussed in detail by the investigators. The training group was instructed to perform the eccentric training program twice a day (14 sessions per week), and the mean value reported was 8.2 ± 4.6 sessions per week. In addition, each player was instructed in person during the first half of the training period (2–6 weeks into the program) to ensure proper execution of the program and exercises. The players in the training group were encouraged to continue the eccentric training if they still had knee pain at the end of the 12-week treatment period. The control group did not receive a training intervention, however, both groups received an information package. The effectiveness of the training was evaluated with the VISA-P score 6 weeks after the 12 weeks intervention period and after 6 months, further global knee function and jumping performance were measured.

| **Study reference** | **Study characteristics** | **Patient characteristics ^2^** | **Intervention (I)** | **Comparison / control (C) ^3^** | **Follow-up** | **Outcome measures and effect size ^4^** | **Comments** |
| --- | --- | --- | --- | --- | --- | --- | --- |
| Frohm, 2007 | Type of study:  prospective  randomised short-term pilot study  Setting and country:  Athletes recruited from physicians and physiotherapists, Sweden  Funding and conflicts of interest:  Swedish Sports Research Council (CIF)  Competing interests: None. | Inclusion criteria:  - athlete  - clinical diagnosis of patellar tendinopathy using MRI of ultrasound  - a characteristic history of patellar tendinopathy continuously for at least 3 months, or recurrent for at least 6 months  Exclusion criteria:  - history of a local corticosteroid injection during the past 3 months, previous anterior cruciate ligament injury or reconstruction, diabetes, chronic inflammatory or rheumatic joint disease, or back pain during the past 3 months  N total at baseline:  I:11  C:9  Important prognostic factors^2^:  age ± SD:  I: 26 ± 8  C: 28 ± 8  Sex:  I:81%M  C: 78%M  Groups comparable at baseline?  Yes | Eccentric overload training device (Bromsman)  Standardised warm-up on a stationary bicycle for 15 min at 100 W  Eccentric strength training alternated with trunk and foot stability training. The trunk training consisted of 3x15 sit-up movements, and the foot stability exercises consisted of one-legged stance at 3x1 min on each leg.  The Bromsman eccentric overload training device consists of a barbell (320 kg) suspended from wires that can be moved up and down along a chosen distance and at a preset speed, by a hydraulic machine. The descending distance was individually set from a standing straight position to approximately 110°of knee flexion, and the speed was set to 0.11 m/s. The patients resisted the movement of the barbell using both legs: 4 sets of 4 repetitions (initial set for warm‐up, then maximal effort for next 3 sets).  Session was rounded off with standardised stretching of the quadriceps and hamstring muscles, complemented with an ice pack over the painful patellar tendon for 20 min.  2 session of 70‐min session per week for 12 weeks  The patients stopped their sport and training activities for the first six weeks of the intervention. During the last six weeks participants slowly resumed supervised jogging and plyometric jump training, but had to keep pain <5 on the 10 point scale. | Standard eccentric rehabilitation programme of squatting exercises on a decline board  Standardised warm-up on a stationary bicycle for 15 min at 100 W  Eccentric strength training alternated with trunk and foot stability training. The trunk training consisted of 3x15 sit-up movements, and the foot stability exercises consisted of one-legged stance at 3x1 min on each leg.  One-legged eccentric training was performed on a 25° board with 3 sets of 15 repetitions of unilateral squats. If the VAS score was <3 for a set, extra weight in front of the chest was added, the load was increased in 5 kg increments, and if VAS exceeded >5, the load was reduced.  Session was rounded off with standardised stretching of the quadriceps and hamstring muscles, complemented with an ice pack over the painful patellar tendon for 20 min.  2 session of 70‐min session per week for 12 weeks | Length of follow-up:  12 weeks  Loss-to-follow-up:  None | Outcome measures and effect size (include 95%CI and p-value if available):  VAS (0–10)  I: median 0 (interquartile range 0–1)  C: median 1 (interquartile range 1–2)  VISA-P  I: 86 (95% CI [71–92])  C: 75 (95% CI [46–83])  No significant difference between groups | Authors’ conclusion: Knee function in athletes with patellar tendinopathy after 3 months was increased similarly after overload eccentric training in the Bromsman device as after eccentric training on the decline board |
| Kongsgaard, 2009 | Type of study:  randomized-controlled single-blind trial  Setting and country:  recreational male  athletes (18–50 years) diagnosed with chronic patellar tendinopathy  applied for trial submission (self-selection following  advertisement).  Institute of Sports  Medicine, Copenhagen, Denmark  Funding and conflicts of interest:  Team Denmarks Research Foundation, The Danish Ministry  of Culture and the Danish Arthritis Foundation  Conflicts of interest: n.r. | Inclusion criteria:  - clinical diagnosis of patellar tendinopathy confirmed by ultrasound  - pain duration of >3 months  - 4-week ‘‘wash-out’’ period from any previous treatment  Exclusion criteria:  (1) corticosteroid  injections within 12 months, (2) previous knee surgery, (3)  arthritis, (4) diabetes or (5) any confounding diagnosis to the knee joint  N total at baseline:  I:13  C:13  Important prognostic factors^2^:  age ± SD:  I: 31.3 ± 8.3  C: 31.7 ± 8.5  BMI:  I: 24.8 ± 2.2  C: 24.4 ± 2.1  Groups comparable at baseline?  Yes | Subjects performed three sets of 15 slow repetitions of eccentric unilateral squats on a 25^o^ decline board twice daily (morning and evening) for 12 consecutive weeks. Subjects were instructed to spend approximately 3s completing each repetition and to have a 2-min rest period between sets. Pain during exercises was acceptable, but pain and discomfort was not to increase following cessation of training. Load was increased using an incrementally loaded backpack as pain diminished.  3 sessions including 1 supervised session per week for 12 weeks | Each session consisted of three bilateral exercises: squat, leg press and hack squat. Subjects completed four sets in each exercises with a 2–3-min rest between sets. The repetitions/loads were: 15 repetition maximum (RM) week 1, 12RM weeks 2–3, 10RM weeks 4–5, 8RM weeks 6–8 and 6RM weeks 9–12. All exercises were performed from complete extension to 901 of knee flexion and back again. Subjects were instructed to spend three seconds completing each of the eccentric and concentric phases, respectively (i.e. 6 s/repetition). Pain during exercises was acceptable but pain and discomfort was not to increase following cessation of training.  3 sessions including 1 supervised session per week for 12 weeks | Length of follow-up:  12 weeks  Loss-to-follow-up:  I: 1 (sports-related ankle sprain)  C: 0  Incomplete outcome data:  I: 9 returned follow up questionnaire  C: 11 returned follow up questionnaire | Outcome measures and effect size (include 95%CI and p-value if available):  VAS 12 weeks (0–100)  I: 31 ± 26  C: 19 ± 15  VISA-P 12 weeks  I: 75 ± 3  C: 78 ± 18  Satisfaction 12 week (satisfied/total)  I: 5/12  C: 9/13  VAS 6 months (0–100)  I: 22 ± 17  C: 13 ± 16  VISA-P 6 months  I: 756 ± 16  C: 86 ± 12  Satisfaction 6 months (satisfied/total)  I: 2/9  C: 8/11 |  |
| Stasinopoulos, 2012 | Type of study:  Controlled clinical trial  Setting and country:  private outpatient physiotherapy clinic in Athens, Greece  Funding and conflicts of interest:  This research received no specific grant from any funding agency in the public, commercial, or not for profit sectors  Conflicts of interest: n.r. | Inclusion criteria:  - between 18-30 years old  - tenderness with palpation over the inferior pole of the patella;  - no history of trauma to the knee;  - minimum duration of symptoms three months;  - unsuccessful conservative treatment before entering the study, but not in the preceding one month;  - no other current knee or lower extremity problems including anterior knee pain, muscle strains and hip or ankle injuries;  - positive decline squat test  Exclusion criteria:  -  N total at baseline:  I: 22  C: 21  Important prognostic factors^2^:  age ± SD:  I: 26.38 ± 4.32  C: 27.04 ± 5.11  Sex:  I: 72%M  C: 71%M  Groups comparable at baseline?  Yes | Eccentric training of patellar tendon and static  stretching exercises of quadriceps and hamstrings  Unilateral squat on a 25^o^ decline board, performed  at a slow speed and with mild pain  3 sets of 15 repetitions  Individualized stretching exercises before and after ECC exercises  30‐s hold, 1‐min rest between each stretch  5x per week for 4 weeks | Eccentric training of patellar tendon:  Unilateral squat on a 25^o^ decline board, performed  at a slow speed and with mild pain  3 sets of 15 repetitions  5x per week for 4 weeks | Length of follow-up:  4 and 24 weeks  Loss-to-follow-up:  none | Outcome measures and effect size (include 95%CI and p-value if available):  VISA-P week 4  I:86 (95%CI: 70 to 94)  C: 74 (95%CI: 58 to 82)  VISA-P week 24  I:94 (95%CI: 76 to 100)  C: 77 (95%CI: 68 to 84)  p < 0.05 | Authors’ conclusion: Eccentric training and static stretching exercises is superior to eccentric training alone to reduce pain and improve function in patients with patellar tendinopathy |
| Van Ark, 2016 | Type of study:  Randomized clinical trial  Setting and country:  Victorian volleyball leagues and basketball leagues, Australia  Funding and conflicts of interest:  The first author has been supported by Foundation “De DrieLichten”, “Wetenschappelijk College Fysiotherapie” and “Anna Foundation | NOREF” in The Netherlands for this project. This study has also been supported by the Australian Institute of Sport. | Inclusion criteria:  volleyball and basketball players (16–32years) playing or training at least three times per week, presenting with patellar tendinopathy diagnosed by an experienced physiotherapist.  Focal tendon pain at the inferior or superior pole of the patella and a history of exercise associated knee pain at the same spot.  Exclusion criteria:  Existence of other knee pathology, previous patellar tendon rupture, previous patellar tendon surgery, inflammatory disorders, metabolic bone diseases, type II diabetes, use of fluroquinolones or corticosteroids in the last 12 months, known familial hypercholesterolemia and chronic pain conditions  N total at baseline:  I: 13  C: 16  Important prognostic factors^2^:  Age ± SD:  I: 22.9 ± 4.9  C: 23.1 ± 4.7  Sex:  I: 92%M  C: 94%M  Groups comparable at baseline?  Yes | Isometric group  Leg extension machine: 5 sets of 45s isometric contractions of each leg hold at knee angle 60° flexion at 80% MVC  After performing the exercises for each leg, participants rested for 15 s before continuing with the first leg again. Weight was increased by 2.5% every week if possible.  4 sessions per week for 4 weeks | Isotonic group  Leg extension machine: 4 sets of 8 repetitions, with 3s concentric phase immediately followed by 4s eccentric phase per repetition performed on 80% of 8RM  After performing the exercises for each leg, participants rested for 15 s before continuing with the first leg again. Weight was increased by 2.5% every week if possible.  4 sessions per week for 4 weeks | Length of follow-up:  4 weeks  Loss-to-follow-up:  Drop-out before start program (not responding after inclusion and initial measurements) n=5  Drop-out during exercise program (researchers unable to contact) n=2  Completed exercise program n=22  Excluded  Did not play volleyball / basketball for 2 weeks n=2  (1 other injury, 1 personal reasons)  Incomplete outcome data:  Missing NRS (excluded in analysis f or NRS pain) n=1  Missing VISA (excluded in analysis for VISA) n= 2 | Outcome measures and effect size (include 95%CI and p-value if available):  Pain (NRS)  I median (IQR): 4.0 (2.0-5.0)  N=8  C median (IQR): 2.0 (1.0-3.0)  N=11  Median pain scores improved significantly over the 4-week intervention period within the isometric group (Z = −2.527, p = 0.012, r = −0.63) and within the isotonic group (Z = −2.952, p = 0.003, r = −0.63). There was no significant difference in NRS pain score change (U = 29.0, p = 0.208, r = 0.29) between the isometric group and isotonic group.  VISA-P  I median (IQR): 75.0 (72.5–87.0) N=8  C median (IQR): 79.0 (67.0–86.0) N=10  Median VISA-P scores improved significantly over the 4-week intervention period within the isometric group (Z = −2.201, p = 0.028, r = −0.55)and within the isotonic group (Z = −2.952, p = 0.003, r = −0.66).  There was no significant difference in VISA-P score change (U = 39.5, p = 0.965, r = −0.01) between the isometric group and isotonic group. | Authors’ conclusion: Both isometric and isotonic exercise programs can reduce pain and improve function in athletes with patellar tendinopathy in-season. |
| Visnes, 2005 | Type of study:  Randomized clinical trial  Setting and country:  male and female elite volleyball teams, Norway  Funding and conflicts of interest:  Royal Norwegian Ministry of Culture, the Norwegian Olympic Committee and Confederation of Sport, Norsk Tipping AS, and Pfizer AS. | Inclusion criteria:  patellar tendinopathy symptoms had to have been present for a minimum of 3 months, and the VISA-P < 80 points  aged 18-35 years old  Exclusion criteria:  history of knee problems caused by patellofemoral pain syndrome, inflammatory joint conditions, or degenerative conditions.  N total at baseline:  I: 15  C: 16  Important prognostic factors2:  age ± SD:  I:26.6 ± 4.6  C: 26.4 ± 3.4  Sex:  I: 62%M  C: 69%M  Groups comparable at baseline?  Yes | ECC training group:  The eccentric training program was performed on a 25^o^ decline board at home.  The squat went to 90^o^ of knee flexion, which ensured that the subjects went past 60 ^o^ of knee flexion, the joint angle thought to place maximal load on the patellar tendon  If the VAS score was <3 or 4 extra load was recommended, the load was increased in 5 kg increments, and if VAS exceeded 6 or 7, it was recommended to reduce the load.    Twice daily: 3 sets of 15 repetitions without warming up for 12 weeks.  The training group received an instruction booklet and a 25^o^ aluminium decline board. They were followed up by telephone when the training period started, and the training program was described and discussed in detail by the investigators. In addition, each player was instructed in person during the first half of the training period (2–6 weeks into the program) to ensure proper execution of the program and exercises.  The players in the training group were encouraged to continue the eccentric training if they still had knee pain at the end of the 12-week treatment period.  Both groups received an information package | Control group  No intervention—usual volleyball training  Both groups received an information package | Length of follow-up:  6 weeks and 6 months after the intervention period  Loss-to-follow-up:  I: N=3 (20%)  Reasons: 2 dropouts after randomization; one left the study before the training period started for reasons unrelated to the study, and the other did not return any data, between the 6 weeks and 6 months follow up one participant moved to unknown address  C:0 | Outcome measures and effect size (include 95%CI and p-value if available):  VAS (0–10)  Average VAS 5.1 ± 1.8 during 12 weeks eccentric exercises in ECC training group  VISA-P score at 12 weeks  I: 70.2 ± 15.4  C: 75.4 ± 16.7  between groups immediately  postintervention (p = 0.98), at 6weeks (p = 0.71) and 6 months  (p = 0.99) of follow‐up  The training group was included with a VISA score of 61 ± 15 and the control group with a score of 65 ± 9. |  |

Table 5.5 Evidence table

n.r.: not reported, IQR: inter quartile range, MVC: maximal voluntary contraction, RM: repetition maximum

Notes:

1. Prognostic balance between treatment groups is usually guaranteed in randomized studies, but non-randomized (observational) studies require matching of patients between treatment groups (case-control studies) or multivariate adjustment for prognostic factors (confounders) (cohort studies); the evidence table should contain sufficient details on these procedures

2. Provide data per treatment group on the most important prognostic factors [(potential) confounders]

3. For case-control studies, provide sufficient detail on the procedure used to match cases and controls

4. For cohort studies, provide sufficient detail on the (multivariate) analyses used to adjust for (potential) confounders

| **Study reference**  (first author, publication year) | **Describe method of randomisation^1^** | **Bias due to inadequate concealment of allocation?^2^**  (unlikely/likely/unclear) | **Bias due to inadequate blinding of participants to treatment allocation?^3^**  (unlikely/likely/unclear) | **Bias due to inadequate blinding of care providers to treatment allocation?^3^**  (unlikely/likely/unclear) | **Bias due to inadequate blinding of outcome assessors to treatment allocation?^3^**  (unlikely/likely/unclear) | **Bias due to selective outcome reporting on basis of the results?^4^**  (unlikely/likely/unclear) | **Bias due to loss to follow-up?^5^**  (unlikely/likely/unclear) | **Bias due to violation of**  **intention to treat analysis?^6^**  (unlikely/likely/unclear) |
| --- | --- | --- | --- | --- | --- | --- | --- | --- |
| Frohm, 2007 | The patients were allocated to either of the two treatment groups by random draw of a sealed, opaque envelope that contained the group assignment. | Unlikely | Unlikely | Likely  All patients were examined, trained and tested by the principal investigator (AF), except for the range of motion examinations, which were assessed by two other physiotherapists. | Likely | Unlikely | Unlikely | Unlikely |
| Kongsgaard, 2009 | A computer-generated minimization randomization procedure was used. The minimization randomization procedure was performed according to  activity level, symptom duration and age. | Unlikely | Unclear  Participants applied for trial submission, self-selection following  advertisement | Unclear | Unclear  The ultrasound and MRIs are conducted by a blinded investigator, however, it is not clear if the other outcomes were blinded evaluated. | Unlikely | Unlikely | Unlikely |
| Stasinopoulos, 2012 | Patients were allocated to two groups by sequential, alternate allocation: the first patient was assigned to the eccentric training and static stretching exercises group, the second to the eccentric training group, and so on. | Likely | Unclear  All patients received a written explanation of the trial before entry into the study and then gave signed consent to participate | Unclear | Unlikely  All assessments were conducted by PM who was blind to the patients’ therapy group | Unlikely | Unlikely  There were no dropouts | Unlikely |
| Van Ark, 2015 | Participants were randomised to an exercise program by the draw of a sealed opaque envelope from 40 identical envelopes that were randomised using a randomisation table created by computer software | Unlikely | Unlikely  Participants were not blinded, however it seems unlikely that it lead to bias | Unclear | Unclear | Unlikely | Likely | Unclear |
| Visnes, 2005 | Randomization by a statistician who was blinded to player identity. Players from the same teams were randomized in blocks to different groups. (32 teams) | Unlikely | Unclear  Blinding subjects to group allocation was not possible. | Likely  Participants were followed up by telephone when the training period started, and the training program was described and discussed in detail by 2 of the investigators (H.V. and A.H.). In addition, each player was instructed in person during the first half of the training period (2–6 weeks into the program) to ensure proper execution of the program and exercises. | Unclear  Self recorded by subjects. Weekly diary mailed to investigators who maintained weekly contact with subjects and monitored continuously. | Unclear  Pain is not reported in the results of both groups at baseline and after 12 weeks | Unlikely | Unlikely |

Table 5.6 Risk of bias table for intervention studies (randomized controlled trials)

1. Randomisation: generation of allocation sequences have to be unpredictable, for example computer generated random-numbers or drawing lots or envelopes. Examples of inadequate procedures are generation of allocation sequences by alternation, according to case record number, date of birth or date of admission.
2. Allocation concealment: refers to the protection (blinding) of the randomisation process. Concealment of allocation sequences is adequate if patients and enrolling investigators cannot foresee assignment, for example central randomisation (performed at a site remote from trial location) or sequentially numbered, sealed, opaque envelopes. Inadequate procedures are all procedures based on inadequate randomisation procedures or open allocation schedules..
3. Blinding: neither the patient nor the care provider (attending physician) knows which patient is getting the special treatment. Blinding is sometimes impossible, for example when comparing surgical with non-surgical treatments. The outcome assessor records the study results. Blinding of those assessing outcomes prevents that the knowledge of patient assignement influences the proces of outcome assessment (detection or information bias). If a study has hard (objective) outcome measures, like death, blinding of outcome assessment is not necessary. If a study has “soft” (subjective) outcome measures, like the assessment of an X-ray, blinding of outcome assessment is necessary.
4. Results of all predefined outcome measures should be reported; if the protocol is available, then outcomes in the protocol and published report can be compared; if not, then outcomes listed in the methods section of an article can be compared with those whose results are reported.
5. If the percentage of patients lost to follow-up is large, or differs between treatment groups, or the reasons for loss to follow-up differ between treatment groups, bias is likely. If the number of patients lost to follow-up, or the reasons why, are not reported, the risk of bias is unclear
6. Participants included in the analysis are exactly those who were randomized into the trial. If the numbers randomized into each intervention group are not clearly reported, the risk of bias is unclear; an ITT analysis implies that (a) participants are kept in the intervention groups to which they were randomized, regardless of the intervention they actually received, (b) outcome data are measured on all participants, and (c) all randomized participants are included in the analysis.

# Results

Pain (crucial)

Pain was measured by a visual analogue scale (VAS) in the studies of Frohm (2007), Kongsgaard (2009), and Visnes (2005), and by using the numerical rating scale (NRS) in the study of Van Ark (2015). Both tools range from 0- 10 or 100, where 0 is equal to no pain and 10 or 100 is the worst pain.

Frohm (2007) reported pain after 12 weeks of training. The eccentric overload training group scored a median of 4 (IQR 4 to 6) on the VAS at baseline and a median of 0 (IQR 0 to 1) after 12 weeks (n=11), the standardized eccentric training group scored a median of 5 (IQR 4 to 5) at baseline and a median of 1 (IQR 1 to 2) after 12 weeks (n=9). No between group analyses were performed by Frohm, 2007.

Kongsgaard (2009) showed a mean VAS score of 31 ± 26 in the eccentric group and 19 ± 15 in the HSR group after 12 weeks. After 6 months, the mean VAS score was 22 ± 17 in the eccentric group and 13 ± 16 in the HSR group. No significant differences were found in the relative improvement of the VAS between baseline and 6 months between the groups.

Visnes (2005) reported a mean VAS score of 5.1 ± 8.1 during the eccentric training, however no information is provided about the pain levels of the control group.

Van Ark (2015) presented pain during a single leg decline squat. The isometric group scored a median of 6.3 (IQR 5.3 to 7.0) on the NRS at baseline and a median of 4.0 (IQR 2.0 to 5.0) after 4 weeks (n=8), the isotonic group scored a median of 5.5 (IQR 4.0 to 6.0) at baseline and a median of 2.0 (IQR 1.0 to 3.0) after 4 weeks (n=11). There was no statistically significant difference in the NRS pain score change between the groups (p=0.208).

Due to heterogeneity in the studies and interventions it was not possible to pool these data.

Level of evidence of the literature

The level of evidence regarding the outcome pain started at high as it was based on RCTs, but was downgraded by 3 levels because of study limitations (risk of bias, -1) and number of included patients (imprecision, -2).

Function (crucial)

Pain and function were measured with the VISA-P questionnaire in patients with PT. The VISA-P ranged from 0-100, where a score of 100 means being a completely asymptomatic and fully functioning athlete. This questionnaire was used in all five studies (Frohm, 2007; Kongsgaard, 2009; Stasinopoulos, 2012; Van Ark, 2015; Visnes, 2005).

Frohm (2007) reported the VISA-P score after 12 weeks of training. The eccentric overload training group scored a median of 86 (95%CI 71 to 92) after 12 weeks (n=11), the standardized eccentric training group scored a median of 75 (95%CI 46 to 83) after 12 weeks (n=9). There were no statistically significant differences between the groups.

Kongsgaard (2009) showed a VISA-P score of 75 ± 3 in the eccentric group (n=12) and 78 ± 18 in the HSR group (n=13) after 12 weeks. After 6 months, the mean VISA-P score was 76 ± 16 in the eccentric group and 86 ± 12 in the HSR group. No statistically significant differences between these groups were reported.

Stasinopoulos (2012) reported the VISA-P after 4 and 24 weeks. The eccentric training group with static stretching (n=22) scored 86 (95%CI 70 to 94) after 4 weeks, and 94 (95%CI 75 to 100) after 24 weeks. The eccentric training group without stretching (n=21) scored 74 (95%CI 58 to 82) after 4 weeks, and 77 (95%CI 68 to 84) after 24 weeks. The change in the VISA-P scores between groups showed significant more improvement in the eccentric training group with static stretching at 4 weeks and at 24 weeks (p<0.05).

Van Ark (2015) presented a median score of 75.0 (IQR 72.5 to 87.0) after 4 weeks in the isometric group (n=8), and a median score of 79.0 (IQR 67.0 to 86.0) after 4 weeks the isotonic group (n=10). There was no significant difference in VISA-P score change (p=0.965) between the groups.

Visnes (2005) showed no significant differences between the training group (n=13) and the control group (n=16) in the VISA-P score from baseline to 12 weeks (post intervention), further no significant between-group differences were found at 6 weeks (p=0.71) or at 6 months (p=0.99). The VISA-P score at 12 weeks was 70.2 ± 15.4 for the eccentric training group and 75.4 ± 16.7 for the control group.

Due to heterogeneity in the studies and interventions it was not possible to pool these data.

Level of evidence of the literature

The level of evidence regarding the outcome function started at high as it was based on RCTs, but was downgraded by 3 levels because of study limitations (risk of bias, -1) and number of included patients (imprecision, -2).

Return to sport/work (important)

Return to sport/work was not described as an outcome in the included studies.

Level of evidence of the literature

The level of evidence regarding the outcome return to sport/work was not assessed due to lack of studies.

Duration of absenteeism (important)

Duration of absenteeism was not described as an outcome in the included studies.

Level of evidence of the literature

The level of evidence regarding the outcome duration of absenteeism was not assessed due to lack of studies.

Patient satisfaction (important)

Patient satisfaction was described by Kongsgaard (2009). After 12 weeks intervention, 42% (n=5) of the patients in the eccentric training group were satisfied with their clinical outcome and 70% (n=9) of the patients in the HSR group. After 6 months, 22% (n=2) of the patients in the eccentric training group were satisfied, and 73% (n=8) of the patients in the HSR group.

Level of evidence of the literature

The level of evidence regarding the outcome patient satisfaction started at high as it was based on a RCT trial, but was downgraded by 3 levels because of study limitations (risk of bias, -1) and number of included patients (imprecision, -2)

Patient recovery (important)

Patient recovery was not described as an outcome in the included studies.

Level of evidence of the literature

The level of evidence regarding the outcome patient recovery was not assessed due to lack of studies.

# Conclusions

Pain (crucial)

| **Very low GRADE** | The evidence is very uncertain about the effect of different training types (eccentric, isometric, and isotonic/ heavy slow resistance) over one another on pain.  *Sources: Frohm, 2007; Kongsgaard, 2009; Van Ark, 2015; Visnes, 2005* |
| --- | --- |

Function (crucial)

| **Very low GRADE** | The evidence is very uncertain about the effect of different training types (eccentric, isometric, and isotonic/ heavy slow resistance) over one another on function.  *Sources: Frohm, 2007; Kongsgaard, 2009; Stasinopoulos, 2012; Van Ark, 2015; Visnes, 2005* |
| --- | --- |

Return to sport/work (important)

| **- GRADE** | The outcome measure return to sport/ work was not reported in the included studies.  *Source: -* |
| --- | --- |

Duration of absenteeism (important)

| **- GRADE** | The outcome measure duration of absenteeism was not reported in the included studies.  *Source: -* |
| --- | --- |

Patient satisfaction (important)

| **Very low GRADE** | The evidence is very uncertain about the effect of exercise therapy on patient satisfaction.  *Sources: Kongsgaard, 2009* |
| --- | --- |

Patient recovery (important)

| **low GRADE** | The outcome measure patient recovery was not reported in the included studies.  Source: - |
| --- | --- |

# Considerations - from evidence to recommendation

Exercise therapy is most commonly used in clinical practice to treat PT. Several studies (see, for example, Kjaer 2009; Rudavsky, 2014, Van Rijn, 2019) confirm the importance of exercise therapy, and also in other tendinopathies, most of the evidence supports active treatment (de Vos, 2021)). In addition, studies on the effect of other treatments are almost always combined with exercise therapy. Therefore, exercise therapy is also seen by the expert panel as a cornerstone of the treatment of PT. The purpose of this module was to find the optimal form of exercise therapy based on the current literature.

Five randomized studies were found about the effectiveness of different forms of exercise therapy relative to each other. The sample size in these studies was very small, so few statements can be made about the effectiveness of the different interventions. Therefore, based on the literature, no strong recommendations can be formulated about the effect of the different forms of exercise therapy relative to each other.

It should be kept in mind that prescribing exercise therapy is not a "one size fits all prescription"; this is due, for example, to variations in the stage of PT (reactive or degenerative), differences in the kinetic chain in patients, and the extent and intensity of sports practice and/or workload. This also makes exercise therapy difficult to study, in part because multiple exercises are likely to be used in the strengthening and the timeframe will vary among individuals.

Cook (2016) has postulated a continuum model of tendon pathology, in which roughly two stages can be distinguished. A reactive stage where a rapid increase (of shorter duration) of symptoms is often seen and a degenerative stage where a relatively constant level of symptoms is often experienced. The studies described mostly involve the chronic, degenerative form of PT.

According to the expert panel, the structure of exercise therapy can be shaped by using the following roadmap based on Rudavsky (2014): 1. Pain management, 2. Strength improving, 3. Improving explosiveness/plyometrics, 4. Maintenance.

Pain management

Based on clinical expertise, the expert panel recommends considering patient education and loading recommendations as the basis of treatment of PT. The expert panel believes that patient education contributes to adequate expectation management and planning of more realistic goals. Load counseling has the important goal of making the patient more self-aware and self-reliant.

Effects of patient education are often understudied. Recent research in patients with gluteus medius tendinopathy shows that patient education combined with exercise therapy is more effective than a wait-and-see policy or an injection of corticosteroids (Mellor, 2018). Patient education for patients with PT has 3 elements, according to the expert panel: explanation of the condition, explanation of the prognosis and pain education. Specifically, this involves explaining of the overuse injury that often results in long-term symptoms. In addition, the symptoms may be recurrent, especially if the specific provocative (sports and/or work) load is continued. Pain education involves health care providers sharing their knowledge about pain with the patient. This includes explanation of the neurophysiology of acute and chronic pain (including central sensitization). In the early phase of a reactive tendinopathy, there may still be acute (physiological) pain, while in the chronic phase the pain may be pathological (dysfunctional) (Rio, 2014). In addition to physical factors, increasing attention is being paid to the influence of psychosocial aspects on long-term pain symptoms. Relative rest can probably protect the tendon briefly in the early (reactive) phase of tendinopathy. However, factors such as fear of developing more damage or a complete rupture, fear of movement and catastrophizing the symptoms, especially in the case of long-standing symptoms, can negatively affect recovery. In the presence of these types of factors, pain education can be effective in improving perceived health and reducing healthcare consumption. This has been studied mainly in patients with low back pain, but not yet in patients with PT (Louw, 2014).

Within the pain management stage, advice on load and load capacity of the patellar tendon plays an important role, according to the expert panel. The temporary discontinuation or replacement of provocative (sports and/or work) load by non-provocative (sports and/or work) load is central to reduce the pain in the short term, after which, based on the (pain) response (VAS of 3 (0-10) or lower), it can be gradually built up again. Here it is important to continue to stimulate movement, but to avoid an increase in symptoms as much as possible. Although this is an accepted method of rehabilitation for tendinopathies (Davenport, 2005), its effect has not been investigated in randomized studies.

In the case of a reactive PT, a quick temporary cessation of provocative (sports and/or work) loading may possibly be sufficient for a quick sports and/or work return.

In the short term (up to 45 minutes after an exercise session), heavy isometric contractions could potentially be pain-reducing (Rio et al, 2017). However, these results were not replicated in a later study, which found a small improvement in pain but saw no difference between heavy isometric exercises or heavy dynamic exercises (heavy slow resistance) (Holden, 2019). The acute effects of isometric exercises were also not found in a study in the Achilles tendon (O'Neill, 2018). Since initial studies of heavy isometric exercises currently show conflicting results, the expert panel believes that if there is a good reason for this (e.g., prior to an important competition), it can be applied on a trial and error basis.

Improving strength

After the initial pain management phase (which will be continued during rehabilitation), a phase aimed at improving strength follows. The expert panel recommends starting immediately with some form of strength exercises for the quadriceps whenever possible. It remains complicated to summarize the different interventions (exercises, duration, intensity) investigated in the different studies and evaluated with different outcome measures. Based on the current literature, no strong recommendations can be made as to what is the optimal form of exercise therapy. Exercise forms that can be applied are eccentric, isotonic (concentric and eccentric) and isometric exercises. A commonality within the programs studied seems to be exercises with slow and heavy loading of the quadriceps. Given the results, one form of contraction cannot be placed above another. This is consistent with previous reviews that have listed studies of multiple tendinopathies (Couppe, 2015; Malliaras, 2013). They also indicate that the previously superior eccentric exercises do not currently appear to have added value over concentric exercises of the same intensity. This is also confirmed by a recent RCT published after the literature search during the writing process of this guideline (Breda, 2020). This RCT shows a better result on the VISA-P of a progressive exercise program (with VAS pain < 3) compared to a daily provocative eccentric exercise program. The expert panel therefore recommends choosing slow and heavy exercises at this stage where one or more forms of contraction can be chosen depending on the knee tendon response. Based on the clinical experience of the expert panel, dynamic exercises are preferred here, if tolerated (pain during and after exercise (VAS ≤< 3)). Isometric exercises may be an alternative in case of a pain score higher than three points (0-10) when doing dynamic exercises. Both Frohm, 2007, and Visnes, 2005 recommended adding additional weight in case of a VAS of 3 (0-10) or reducing weight in case of VAS of >3 (0-10). In the experience of the expert panel, isometric exercises provoke less pain in a subset of patients. For those exercises where single-leg execution is possible, the expert panel, recommends doing so to avoid compensation with the other leg. More research is needed to advise on the number of times per week, number of repetitions and use of weights. The expert panel recommends a minimum duration of 12 weeks; at this time improvement in perceived symptoms may be expected after adequate exercise therapy, and this provides a good basis for further increase of tendon loading by the exercises.

A proper coordination between exercise therapy and sports practice/workload does seem to be important. The form of exercise therapy plays a role here. Most of the first (pilot) studies on the best-known eccentric single leg decline squat protocol (Alfredson) show that these provocative exercises are only effective when patients also stop sports participation (Visnes et al, 2006, Jonsson, 2005; Young et al 2005, Purdam, 2004)

Also, a study that applied this protocol preventively in a group of soccer players in preparation for the season showed that in case of already present abnormalities on ultrasound images, the risk of PT actually increased (Fredberg, 2007). Given the provocative loading of the single leg decline squat protocol (Alfredson protocol, Young et al, 2005), the expert panel advises against combining it with (full) sports practice.

Despite the very low evidence base, stretching may be considered in addition to strength exercises in individual cases, especially when there is hamstring and quadriceps shortening (Stasinopoulos, 2012).

In clinical practice, when designing exercise therapy, attention is also paid to the kinetic chain and possible addressing gaps in it. The kinetic chain is the set of muscles and joints throughout the body, where different muscles/joints influence each other. A weakness in another muscle/joint could potentially be explanatory for the development of PT. There has been limited research on risk factors in the kinetic chain for the development of a PT. There are no published studies on the effectiveness of corrections to the kinetic chain in PT. Therefore, the expert panel, did not include this in the recommendations.

Improving explosiveness/ plyometrics

The expert panel recommends a gradual build-up of explosive/plyometric activities during rehabilitation. Examples include jumping and rapid changes of direction. These activities are considered to bring the most load on the patellar tendon. As mentioned earlier, the balance between load and load capacity is important in improving strength. Depending on the (pain) response to explosive (sports) activities, this could be improved in an earlier state or later in the rehabilitation. This can include improving of jumping exercises, but also a (partial) sports return. In the improving of these activities, the degree of pain during and after the exercises will always be leading in the progression (a pain score of three or less can be accepted). Depending on the required capacity for the patient (a top athlete in an explosive sport needs much more capacity than a recreational athlete in a non-explosive sport), more or less attention should be paid to this phase.

Maintenance

Based on clinical experience, the expert panel believes that after full return to sport/work, the patient should be advised to continue (less frequently) strength training of the quadriceps and, in addition, remain mindful of the strain on the patellar tendon by avoiding rapid increases in strain.

Expectations and values of patients (and possibly their caretakers)

To gain more insight into the values and expectations of patients with PT, a questionnaire was prepared and distributed in cooperation with the Netherlands Patients Federation (NPF). All patients (n=9) who completed the questionnaire had received some form of exercise therapy. The main goals for the patient were to regain pain-free performance of daily activities and pain-free exercise, preferably at the old level. The completed questionnaire of the DPF suggests that shared-decision-making has a favourable influence on compliance, little is known about this in the scientific literature (Joosten, 2008. Tousignant-Laflamme, 2017). Furthermore, the expert panel recommends evaluating the exercise program regularly and in a structured manner (VAS and VISA-P) in consultation with the patient. Consultation between health care providers, especially the coordination between physician and physical therapist, is perceived as very valuable by the patient. Disadvantages mentioned are the time investment (practicing several times a week) and the duration until improvement/recovery. It is known that the treatment of PT generally requires a lot of patience and an integrated approach. It is important for the physician/physical therapist and the patient to recognize when a tendinopathy has existed for months, it may require a long rehabilitation period before the symptoms are resolved. The aforementioned patient education explaining the condition and its prognosis plays an important role here. Kongsgaard's (2009) study showed that patients who had to do an exercise session (HSR) three times a week were more satisfied with the treatment than patients who followed the Alfredson protocol (daily exercise), despite no difference in pain scores being observed. Also considering compliance, the expert panel therefore prefers a program (with slow and heavy loading of the quadriceps) involving exercise three times weekly. The expert panel believes that oral information can be well supported by another form of information, for example, an information leaflet or relevant information on reliable Internet sources (for example, [www.thuisarts.nl](http://www.thuisarts.nl/) and [www.sportzorg.nl](http://www.sportzorg.nl/)).

In cases of PT, access to (medical) fitness is desirable in the implementation of exercise therapy. The expert panel believes that exercise therapy using fitness equipment, especially for patients who wish to return to an explosive sport, is preferable to a home exercise regimen. Fitness equipment makes it possible to gradually increase the load and better isolate the quadriceps (using leg extension device), preventing compensation by other muscle groups. Physical therapy supervision is not necessary for every exercise session. Patients who do not play sports or recreational athletes who play non-explosive sports can, for cost reasons, be offered a home exercise regimen.

Cost

No studies have been conducted on the (cost) effectiveness of providing patient education, load recommendations, and advice and guidance for exercise therapy. For exercise therapy, in patients with PT, it is recommended to be performed partly under the supervision of the physical therapist. In general, patients with an unfavourable prognostic profile (e.g., high VAS or NRS and low score on VISA-P, long duration of symptoms, bilateral symptoms) and high functional demands (e.g., return to an explosive sport) have the need for more contacts with the physical therapist than patients with a more favourable prognostic profile and lower functional demands. If the patient has supplementary health insurance and the physical therapist provides contracted care, physical therapy counseling will be at the expense of the supplementary health insurance. In recent years, the scope of physical therapy counseling from the supplementary health insurance has decreased significantly, often limiting the contact reimbursed by health insurance to 6 or 9 treatments. In patients with a favourable prognostic profile and lower functional requirements, a combination of targeted exercise therapy under the supervision of the physical therapist and independently performed exercise therapy (at home or at the gym) this may be sufficient. In patients with an unfavourable prognostic profile and higher functional demands, 6 or 9 treatments are generally insufficient. Patients in this situation must pay for physical therapy care themselves. In addition, a patient will have to pay for the potentially additional medical fitness or gym membership themselves. In practice, this will often lead to non-optimal care and lack of therapeutic effects, because not every patient has the financial means to pay for it. This is an undesirable situation.

Acceptability, feasibility and implementation

The following factors could affect the implementation of the recommendations:

Exercise therapy for patients with PT is performed (in part) under the supervision of the physical therapist. Physical therapy is not covered from the basic insurance, but from the supplementary insurance. The number of covered treatments varies by health insurer and type of supplementary insurance. Patients without supplementary insurance or with a limited number of covered treatments, will have to pay (part of the therapy) themselves. The cost of any medical fitness or gym membership will also be for the patient. Compliancy plays an important role when it comes to the chance of recovery, because the patient will have to perform exercises under supervision or independently for an extended period of time. In the context of exercise therapy for PT, the following factors may influence compliance; patient education about the rationale for exercises and dosages, time frame over which effects can be expected and the possible increase in anterior knee pain due to the exercise therapy (expectation management).

For the provision of complete and standardized information and education regarding the condition and the advice and implementation of exercise therapy, it is desirable that there is agreement between the various health care providers. For the Dutch situation, where many disciplines are involved in the treatment of PT, further elaboration is likely to be conducive to implementation. The expert panel believes that there are sufficient trained health care providers and gyms available for successful implementation of the recommendations.

# Rationale of the recommendations

Exercise therapy is most commonly used in clinical practice as a treatment for PT. Various studies (see, for example, Kjaer, 2009; Rudavsky, 2014; Van Rijn, 2019) confirm the importance of exercise therapy. In addition, studies into the effect of additional conservative treatments are almost always combined with exercise therapy. Exercise therapy is therefore also seen by the expert panel as a base of the treatment of PT. The aim of this module was to find out what the optimal form of exercise therapy is based on the current literature in this area. Five randomized studies were found on the effectiveness of the different forms of exercise therapy in relation to each other. The study populations are very small, few statements can be made about the effectiveness of the various interventions. Therefore, no strong recommendations can be formulated on the literature about the effect of the different forms of exercise therapy in relation to each other.

Treatment of PT generally requires a long rehabilitation and an integrated approach. Patient education with explanations about the condition and the prognosis plays an important role in this. Limited number of studies and relatively low quality do not allow a recommendation for a specific exercise program. In addition, individual differences ensure that treatment of PT is not a 'one size fits all recipe'. Partly because the load on the patellar tendon plays an important role in PT, patient education and loading advice are crucial. Exercise therapy with slow and heavy exercises (3x per week) is preferred. The recommendations have been taking into account the (limited) available literature and clinical experience of the expert panel.

The expert panel recommends exercise therapy (partly) under the supervision of a physiotherapist. The advantage is supervision of the correct execution of the exercises, that the load can be further expanded in a safe and responsible manner and intervention can be made in the event of an impending overload. In addition, adherence to therapy plays an important role when it comes to the chance of recovery. Shared-decision-making has a beneficial effect on treatment adherence. The expert panel also recommends regularly evaluation of the exercise program and in a structured manner (VAS and VISA-P) in consultation with the patient. Consultation between care providers, and in particular the coordination between physician and physiotherapist, is marked as very valuable by the patient. The disadvantages mentioned are the time investment (exercising several times a week) and the time to improvement/recovery.

According to the expert panel, the structure of exercise therapy can be shaped by using the following step-by-step plan based on Rudavsky (2014): 1. Pain management, 2. Strength improving, 3. Explosiveness improving /plyometrics, 4. Maintenance.

# References

Breda, S. J., Oei, E. H., Zwerver, J., Visser, E., Waarsing, E., Krestin, G. P., & de Vos, R. J. (2021). Effectiveness of progressive tendon-loading exercise therapy in patients with patellar tendinopathy: a randomised clinical trial. *British Journal of Sports Medicine*;55(9):501-509.

Cook, J. L., Rio, E., Purdam, C. R., & Docking, S. I. (2016). Revisiting the continuum model of tendon pathology: What is its merit in clinical practice and research? *British Journal of Sports Medicine,* 50(19), 1187–1191.

Couppé, C., Svensson, R. B., Silbernagel, K. G., Langberg, H., & Magnusson, S. P. (2015). Eccentric or concentric exercises for the treatment of tendinopathies?. J*ournal of Orthopaedic & sports physical therapy*, *45*(11), 853-863.

Davenport, T. E., Kulig, K., Matharu, Y., & Blanco, C. E. (2005). The EdUReP model for nonsurgical management of tendinopathy. *Phys Ther, 85*(10), 1093-1103.

Fredberg, U., Bolvig, L., & Andersen, N. T. (2008). Prophylactic training in asymptomatic soccer players with ultrasonographic abnormalities in Achilles and patellar tendons: the Danish Super League Study. *The American journal of sports medicine*, *36*(3), 451-460.

Frohm, A., Saartok, T., Halvorsen, K., & Renström, P. (2007). Eccentric treatment for patellar tendinopathy: A prospective randomised shortterm pilot study of two rehabilitation protocols. *British Journal of Sports Medicine*, 41(7), e7.

Holden, S., Lyng, K., Graven-Nielsen, T., Riel, H., Olesen, J. L., Larsen, L. H., & Rathleff, M. S. (2020). Isometric exercise and pain in patellar tendinopathy: A randomized crossover trial. Journal of Science and Medicine in Sport, 23(3), 208-214.

Joosten, E. A., DeFuentes-Merillas, L., De Weert, G. H., Sensky, T., Van Der Staak, C. P. F., & de Jong, C. A. (2008). Systematic review of the effects of shared decision-making on patient satisfaction, treatment adherence and health status. *Psychotherapy and psychosomatics*, *77*(4), 219-226.

Jonsson, P., & Alfredson, H. (2005). Superior results with eccentric compared to concentric quadriceps training in patients with jumper’s knee: a prospective randomised study. *British journal of sports medicine*, *39*(11), 847-850.

Kjær, M., Langberg, H., Heinemeier, K., Bayer, M. L., Hansen, M., Holm, L., ... & Magnusson, S. P. (2009). From mechanical loading to collagen synthesis, structural changes and function in human tendon. *Scandinavian journal of medicine & science in sports*, *19*(4), 500-510.

Kongsgaard, M., Kovanen, V., Aagaard, P., et al. (2009). Corticosteroid injections, eccentric decline squat training and heavy slow resistance training in patellar tendinopathy. *Scand J Med Sci Sports*, 19: 790–802

Louw, A., Diener, I., Landers, M. R., & Puentedura, E. J. (2014). Preoperative pain neuroscience education for lumbar radiculopathy: a multicenter randomized controlled trial with 1-year follow-up. *Spine (Phila Pa 1976), 39*(18), 1449-1457.

Mellor, R., Bennell, K., Grimaldi, A., Nicolson, P., Kasza, J., Hodges, P., . . . Vicenzino, B. (2018). Education plus exercise versus corticosteroid injection use versus a wait and see approach on global outcome and pain from gluteal tendinopathy: prospective, single blinded, randomised clinical trial. *BMJ, 361*, k1662.

O’Neill, S., Radia, J., Bird, K., Rathleff, M. S., Bandholm, T., Jorgensen, M., & Thorborg, K. (2019). Acute sensory and motor response to 45-S heavy isometric holds for the plantar flexors in patients with Achilles tendinopathy. *Knee Surgery, Sports Traumatology, Arthroscopy*, 27(9), 2765-2773.

Purdam, C. R., Jonsson, P., Alfredson, H., Lorentzon, R., Cook, J. L., & Khan, K. M. (2004). A pilot study of the eccentric decline squat in the management of painful chronic patellar tendinopathy. *British journal of sports medicine*, *38*(4), 395-397.

Rio, E., Moseley, L., Purdam, C., Samiric, T., Kidgell, D., Pearce, A. J., . . . Cook, J. (2014). The pain of tendinopathy: physiological or pathophysiological? *Sports Med, 44*(1), 9-23.

Rio, E., Van Ark, M., Docking, S., Moseley, G. L., Kidgell, D., Gaida, J. E., ... & Cook, J. (2017). Isometric contractions are more analgesic than isotonic contractions for patellar tendon pain: an in-season randomized clinical trial. *Clinical Journal of Sport Medicine*, 27(3), 253-259.

Rudavsky, A., & Cook, J. (2014). Physiotherapy management of patellar tendinopathy (jumper's knee). *Journal of physiotherapy*, *60*(3), 122-129.

Silbernagel KG, Thomeé R, Eriksson BI, Karlsson J. (2007). Continued sports activity, using a pain-monitoring model, during rehabilitation in patients with Achilles tendinopathy: a randomized controlled study. *Am J Sports Med*.;35(6):897-906.

Sprague, A. L., Smith, A. H., Knox, P., Pohlig, R. T., & Silbernagel, K. G. (2018). Modifiable risk factors for patellar tendinopathy in athletes: a systematic review and meta-analysis. British journal of sports medicine, 52(24), 1575-1585.

Stasinopoulos, D., Manias, P., & Stasinopoulou, K. (2012). Comparing the effects of eccentric training with eccentric training and static stretching exercises in the treatment of patellar tendinopathy. A controlled clinical trial*. Clinical Rehabilitation,* 26(5), 423–430.

Tousignant-Laflamme, Y., Christopher, S., Clewley, D., Ledbetter, L., Cook, C. J., & Cook, C. E. (2017). Does shared decision making results in better health related outcomes for individuals with painful musculoskeletal disorders? A systematic review. *Journal of Manual & Manipulative Therapy*, *25*(3), 144-150.

van Ark, M., Cook, J., Docking, S., Zwerver, J., Gaida, J., van den Akker‐Scheek, I., & Rio, E. (2016). Do isometric and isotonic exercise programs reduce pain in athletes with patellar tendinopathy in‐season? A randomised clinical trial. *Journal of Science and Medicine in Sport*, 19(9), 702–706.

van Rijn, D., van den Akker-Scheek, I., Steunebrink, M., Diercks, R. L., Zwerver, J., & van der Worp, H. (2019). Comparison of the effect of 5 different treatment options for managing patellar tendinopathy: a secondary analysis. *Clinical Journal of Sport Medicine*, *29*(3), 181-187.

Visnes, H., Hoksrud, A., Cook, J., & Bahr, R. (2005). No effect of eccentric training on jumper's knee in volleyball players during the competitive season: A randomized clinical trial. *Scandinavian Journal of Medicine & Science in Sports*, 16(3), 227–234.

Young, M. A., Cook, J. L., Purdam, C. R., Kiss, Z. S., & Alfredson, H. (2005). Eccentric decline squat protocol offers superior results at 12 months compared with traditional eccentric protocol for patellar tendinopathy in volleyball players. *British journal of sports medicine, 39(2), 102-105.*

# Construction of Module 6 ‘Additional conservative treatments for PT’

Scoping Question: What is the value of additional conservative treatments (including foot orthoses, extracorporeal shock wave therapy (ESWT), low-intensity pulsed ultrasound (LIPUS), percutaneous electrolysis therapy (EPTE), taping, dry needling, patellar strap) in patients with PT?

# Search and select

A systematic review of literature was performed to answer the following question:

What is the effect of a conservative treatment (i.e. foot orthoses, shockwave, percutaneous electrolysis therapy, taping, dry needling, patellar straps) in patients with PT on pain, function, return to sport/work, duration of absenteeism, patients satisfaction, and patient recovery?

P: adult patients with PT;

I: conservative treatment (i.e. foot orthoses, shockwave, percutaneous electrolysis therapy, taping, dry needling, patellar straps);

C: control group/ placebo/ wait and see policy;

O: pain, function, return to sport/work, duration of absenteeism, patient satisfaction, and patient recovery.

Relevant outcome measures

The expert panel considered pain and function as critical outcome measures for decision making; and return to sport/work, duration of absenteeism, patient satisfaction, and patient recovery as important outcome measures for decision making.

The expert panel defined the outcome measures as following:

- Pain: as determined with the Visual Analogue Scale (VAS) or the Numeric Rating Scale (NRS).
- Function: as determined with the Victorian Institute of Sport Assessment-Patella (VISA-P) questionnaire, which is developed to measure severity of symptoms, pain, and function in patients with PT.
- Return to sport/work: as determined with the Tegner score.
- Patient satisfaction: as determined on a Likert scale.
- Patient recovery: as determined on a Likert scale.

A priori, the expert panel did not define the outcome measure ‘duration of absenteeism’ but used the definitions used in the studies.

The expert panel defined a minimal clinically important difference of 2 cm (out of 10 cm) change on the VAS or NRS scale, in accordance with Crossley (2004). Additionally, a difference of 13 points in the VISA-P score was the minimal clinically important difference (Hernandez-Sanchez, 2014). A minimal clinically important difference for return to sport/work, duration of absenteeism, patient satisfaction, and patient recovery was not predefined.

Search and select (Methods)

The databases Medline (via OVID) and Embase (via Embase.com) were searched with relevant search terms until July 2020, unrestricted to publication year. The detailed search strategy is presented in the tables. The systematic literature search resulted in 341 hits. Studies were selected based on the following inclusion criteria:

- Systematic reviews (searched in at least two databases, detailed search strategy, risk of bias assessment, and results of individual studies available) or randomized controlled trials (RCTs).
- Answering the search question described above.
- At least 20 patients with PT included.

Initially, 30 studies were selected based on title and abstract screening. After reading the full text, 24 studies were excluded (Table 6.3) and six RCTs were included.

Results

Six RCTs were included in the analysis of the literature. Important study characteristics and results were summarized in the evidence tables. The risk of bias assessment is presented in the risk of bias tables.

Key articles

1. Zwerver, J., Hartgens, F., Verhagen, E., van der Worp, H., van den Akker-Scheek, I., & Diercks, R. L. (2011). No effect of extracorporeal shockwave therapy on patellar tendinopathy in jumping athletes during the competitive season: a randomized clinical trial. *The American journal of sports medicine*, *39*(6), 1191-1199.

2. Thijs, K. M., Zwerver, J., Backx, F. J., Steeneken, V., Rayer, S., Groenenboom, P., & Moen, M. H. (2017). Effectiveness of shockwave treatment combined with eccentric training for patellar tendinopathy: a double-blinded randomized study. *Clinical journal of sport medicine*, *27*(2), 89-96.

3. Van der Worp, H., Zwerver, M., Hamstra, M. et al, No difference in effectiveness between focused and radial shockwave therapy for treating patellar tendinopathy: a randomized controlled trial. Knee Surg Sport Tr A. 2014;22:2026–2032

4. Clin Rehabil. 2004 Jun;18(4):347-52. Comparison of effects of exercise programme, pulsed ultrasound and transverse friction in the treatment of chronic patellar tendinopathy. Stasinopoulos D1, Stasinopoulos I.

5. Scand J Med Sci Sports. 2016 Oct;26(10):1217-24. doi: 10.1111/sms.12556. Epub 2015 Sep 17.

Effect of patellar strap and sports tape on pain in patellar tendinopathy: A randomized controlled trial.

de Vries A1, Zwerver J2, Diercks R2, Tak I3, van Berkel S4, van Cingel R5, van der Worp H2, van den Akker-Scheek I2.

| **Database** | **Search terms** |
| --- | --- |
| Embase | | **No.** | **Query** | **Results** | | --- | --- | --- | | #10 | #7 OR #8 OR #9 | 259 | | #9 | #3 AND #6 NOT (#7 OR #8) | 90 | | #8 | #3 AND #5 NOT #7 | 120 | | #7 | #3 AND #4 | 49 | | #6 | 'major clinical study'/de OR 'clinical study'/de OR 'case control study'/de OR 'family study'/de OR 'longitudinal study'/de OR 'retrospective study'/de OR 'prospective study'/de OR 'cohort analysis'/de OR ((cohort NEAR/1 (study OR studies)):ab,ti) OR (('case control' NEAR/1 (study OR studies)):ab,ti) OR (('follow up' NEAR/1 (study OR studies)):ab,ti) OR (observational NEAR/1 (study OR studies)) OR ((epidemiologic NEAR/1 (study OR studies)):ab,ti) OR (('cross sectional' NEAR/1 (study OR studies)):ab,ti) | 5326563 | | #5 | 'clinical trial'/exp OR 'randomization'/exp OR 'single blind procedure'/exp OR 'double blind procedure'/exp OR 'crossover procedure'/exp OR 'placebo'/exp OR 'prospective study'/exp OR rct:ab,ti OR random*:ab,ti OR 'single blind':ab,ti OR 'randomised controlled trial':ab,ti OR 'randomized controlled trial'/exp OR placebo*:ab,ti | 3075301 | | #4 | 'meta analysis'/de OR cochrane:ab OR embase:ab OR psycinfo:ab OR cinahl:ab OR medline:ab OR ((systematic NEAR/1 (review OR overview)):ab,ti) OR ((meta NEAR/1 analy*):ab,ti) OR metaanalys*:ab,ti OR 'data extraction':ab OR cochrane:jt OR 'systematic review'/de | 508544 | | #3 | #1 AND #2 NOT ('conference abstract'/it OR 'editorial'/it OR 'letter'/it OR 'note'/it) | 626 | | #2 | 'orthosis'/exp OR 'orthotic*':ti,ab,kw OR 'orthose*':ti,ab,kw OR 'inlay*':ti,ab,kw OR insole*:ti,ab,kw OR 'brace*':ti,ab,kw OR 'bracing':ti,ab,kw OR 'knee support':ti,ab,kw OR 'athletic tape'/exp OR 'tape*':ti,ab,kw OR 'taping':ti,ab,kw OR 'dry needling'/exp OR (dry:ti,ab,kw AND needl*:ti,ab,kw) OR 'acupuncture'/exp OR 'acupuncture':ti,ab,kw OR 'massage'/exp OR 'massag*':ti,ab,kw OR 'cryotherapy'/exp OR cryogenic:ti,ab,kw OR cryothermy:ti,ab,kw OR cryotreatment:ti,ab,kw OR (((cold OR hypothermal) NEAR/3 (bath OR therapy)):ti,ab,kw) OR 'transcutaneous electrical nerve stimulation'/exp OR (((transcutaneous OR percutaneous) NEAR/3 (electrostimulation OR 'nerve stimulation' OR 'electrical stimulation')):ti,ab,kw) OR tens:ti,ab,kw OR 'shock wave therapy'/exp OR shockwave:ti,ab,kw OR 'shock wave':ti,ab,kw OR eswt:ti,ab,kw OR epte:ti,ab,kw OR 'percutaneous electrolysis':ti,ab,kw OR 'electrolysis percutanea':ti,ab,kw OR 'phototherapy'/exp OR 'light therapy':ti,ab,kw OR 'phototherapy':ti,ab,kw OR 'laser therapy':ti,ab,kw OR lasertherapy:ti,ab,kw OR 'chiropractic'/exp OR 'chiropractic manipulation'/exp OR chiropractic*:ti,ab,kw OR cheirotherapy:ti,ab,kw OR chiropraxia:ti,ab,kw OR chiropraxis:ti,ab,kw OR chirotherapy:ti,ab,kw OR 'soft tissue therapy'/exp OR 'soft tissue therapy':ti,ab,kw OR 'patellar strap*':ti,ab,kw OR 'knee strap*':ti,ab,kw OR 'walking'/exp OR 'walk*':ti,ab,kw OR 'ambulation':ti,ab,kw OR 'gait':ti,ab,kw OR 'running'/exp OR 'running':ti,ab,kw OR 'jogging':ti,ab,kw OR 'cycling'/exp OR 'bicycling':ti,ab,kw OR 'biking':ti,ab,kw OR 'bike*':ti,ab,kw OR 'behavior therapy'/exp OR 'behavioral therapy':ti,ab,kw OR 'behavior therapy':ti,ab,kw OR 'behaviour therapy':ti,ab,kw OR 'behavioral therapies':ti,ab,kw OR 'behavior therapies':ti,ab,kw OR 'behaviour therapies':ti,ab,kw OR 'mindfulness'/exp OR 'mindfullness':ti,ab,kw OR 'meditation'/exp OR 'meditation':ti,ab,kw OR 'patient education'/exp OR 'patient education':ti,ab,kw OR 'patient re-education':ti,ab,kw OR 'patient reeducation':ti,ab,kw OR 'consumer health information'/exp OR 'health literacy':ti,ab,kw OR 'information':ti,ab,kw OR 'pain management':ti,ab,kw OR (('pain relief' NEAR/3 (therap* OR treatment*)):ti,ab,kw) OR 'psychology'/exp OR psycholog*:ti,ab,kw OR 'psychotherapy'/de OR psychotherap*:ti,ab,kw OR 'ultrasound therapy'/exp OR 'therapeutic ultrasonic':ti,ab,kw OR 'therapeutic ultrasound*':ti,ab,kw OR ((ultrasonic NEAR/2 therapy):ti,ab,kw) OR 'ultrasound therapy':ti,ab,kw OR 'conservative treatment'/exp OR 'conservative treatment*':ti,ab,kw OR 'conservative therap*':ti,ab,kw OR 'conservative management':ti,ab,kw | 3634420 | | #1 | 'patellar tendinopathy'/exp OR 'jumper s knee':ti,ab,kw OR 'patella apicitis':ti,ab,kw OR 'patellar apicitis':ti,ab,kw OR 'patella apex syndrome':ti,ab,kw OR 'patellar apex syndrome':ti,ab,kw OR 'patella tip syndrome':ti,ab,kw OR 'patellar tip syndrome':ti,ab,kw OR 'patella tenosynovitis':ti,ab,kw OR 'patellar tenosynovitis':ti,ab,kw OR (('patellar ligament'/exp OR 'patella'/exp OR 'patella':ti,ab,kw OR 'patellar':ti,ab,kw) AND ('tendinitis'/exp OR 'tendinopathy':ti,ab,kw OR 'tendinopathies':ti,ab,kw OR 'tendinosis':ti,ab,kw OR 'tendinoses':ti,ab,kw OR 'tendinitis':ti,ab,kw OR 'tendinitides':ti,ab,kw OR 'tendonitis':ti,ab,kw OR 'tendonitides':ti,ab,kw OR 'peritendinitis':ti,ab,kw OR 'paratenonitis':ti,ab,kw OR ('tendon':ti,ab,kw AND ('pathology':ti,ab,kw OR 'rupture':ti,ab,kw OR 'ruptures':ti,ab,kw)))) | 2842 | |
| Medline (OVID) | 1 ("jumper s knee" or "patella apicitis" or "patellar apicitis" or "patella apex syndrome" or "patellar apex syndrome" or "patella tip syndrome" or "patellar tip syndrome" or "patella tenosynovitis" or "patellar tenosynovitis").ti,ab,kf. or ((exp Patellar Ligament/ or exp Patella/ or "Patella".ti,ab,kf. or "patellar".ti,ab,kf.) and (Tendinopathy/ or "Tendinopathy".ti,ab,kf. or "Tendinopathies".ti,ab,kf. or "tendinosis".ti,ab,kf. or "tendinoses".ti,ab,kf. or "tendinitis".ti,ab,kf. or "Tendinitides".ti,ab,kf. or "tendonitis".ti,ab,kf. or "Tendonitides".ti,ab,kf. or "peritendinitis".ti,ab,kf. or "paratenonitis".ti,ab,kf. or ("tendon" and ("pathology" or "rupture" or "ruptures")).ti,ab,kf.)) (2100)  2 exp Orthotic Devices/ or 'orthotic*'.ti,ab,kf. or 'orthose*'.ti,ab,kf. or 'inlay*'.ti,ab,kf. or insole*.ti,ab,kf. or 'brace*'.ti,ab,kf. or 'bracing'.ti,ab,kf. or 'knee support'.ti,ab,kf. or exp Athletic Tape/ or 'tape*'.ti,ab,kf. or 'taping'.ti,ab,kf. or exp Acupuncture Therapy/ or 'acupuncture'.ti,ab,kf. or exp Dry Needling/ or (dry and needl*).ti,ab,kf. or Massage/ or 'massag*'.ti,ab,kf. or exp Cryotherapy/ or cryogenic.ti,ab,kf. or cryothermy.ti,ab,kf. or cryotreatment.ti,ab,kf. or ((cold or hypothermal) adj3 (bath or therapy)).ti,ab,kf. or exp Transcutaneous Electric Nerve Stimulation/ or ((transcutaneous or percutaneous) adj3 (electrostimulation or 'nerve stimulation' or 'electrical stimulation')).ti,ab,kf. or TENS.ti,ab,kf. or exp Extracorporeal Shockwave Therapy/ or shockwave.ti,ab,kf. or 'shock wave'.ti,ab,kf. or ESWT.ti,ab,kf. or EPTE.ti,ab,kf. or 'percutaneous electrolysis'.ti,ab,kf. or 'electrolysis percutanea'.ti,ab,kf. or exp Phototherapy/ or 'light therapy'.ti,ab,kf. or 'phototherapy'.ti,ab,kf. or exp Laser Therapy/ or 'laser therapy'.ti,ab,kf. or lasertherapy.ti,ab,kf. or exp Manipulation, Chiropractic/ or exp Chiropractic/ or chiropractic*.ti,ab,kf. or cheirotherapy.ti,ab,kf. or chiropraxia.ti,ab,kf. or chiropraxis.ti,ab,kf. or chirotherapy.ti,ab,kf. or exp Therapy, Soft Tissue/ or 'soft tissue therapy'.ti,ab,kf. or 'patellar strap*'.ti,ab,kf. or 'knee strap*'.ti,ab,kf. or exp Walking/ or walk*.ti,ab,kf. or ambulation.ti,ab,kf. or gait.ti,ab,kf. or exp Running/ or Running.ti,ab,kf. or jogging.ti,ab,kf. or exp Bicycling/ or bicycling.ti,ab,kf. or biking.ti,ab,kf. or bike*.ti,ab,kf. or exp behavior therapy/ or 'behavioral therapy'.ti,ab,kf. or 'behavior therapy'.ti,ab,kf. or 'behaviour therapy'.ti,ab,kf. or 'behavioral therapies'.ti,ab,kf. or 'behavior therapies'.ti,ab,kf. or 'behaviour therapies'.ti,ab,kf. or exp mindfulness/ or 'mindfullness'.ti,ab,kf. or exp meditation/ or 'meditation'.ti,ab,kf. or Patient Education as Topic/ or 'patient education'.ti,ab,kf. or 'patient re-education'.ti,ab,kf. or 'patient reeducation'.ti,ab,kf. or exp consumer health information/ or 'health literacy'.ti,ab,kf. or 'information'.ti,ab,kf. or exp Pain Management/ or 'pain management'.ti,ab,kf. or ('pain relief' adj3 (therap* or treatment*)).ti,ab,kf. or exp psychology/ or psycholog*.ti,ab,kf. or psychotherapy/ or psychotherap*.ti,ab,kf. or exp Ultrasonic Therapy/ or 'therapeutic ultrasonic'.ti,ab,kf. or 'therapeutic ultrasound*'.ti,ab,kf. or (ultrasonic adj2 therapy).ti,ab,kf. or 'ultrasound therapy'.ti,ab,kf. or exp Conservative Treatment/ or 'conservative treatment*'.ti,ab,kf. or 'conservative therap*'.ti,ab,kf. or 'conservative management'.ti,ab,kf. (2305621)  3 1 and 2 (453)  4 (meta-analysis/ or meta-analysis as topic/ or (meta adj analy$).tw. or ((systematic* or literature) adj2 review$1).tw. or (systematic adj overview$1).tw. or exp "Review Literature as Topic"/ or cochrane.ab. or cochrane.jw. or embase.ab. or medline.ab. or (psychlit or psyclit).ab. or (cinahl or cinhal).ab. or cancerlit.ab. or ((selection criteria or data extraction).ab. and "review"/)) not (Comment/ or Editorial/ or Letter/ or (animals/ not humans/)) (455749)  5 (exp clinical trial/ or randomized controlled trial/ or exp clinical trials as topic/ or randomized controlled trials as topic/ or Random Allocation/ or Double-Blind Method/ or Single-Blind Method/ or (clinical trial, phase i or clinical trial, phase ii or clinical trial, phase iii or clinical trial, phase iv or controlled clinical trial or randomized controlled trial or multicenter study or clinical trial).pt. or random*.ti,ab. or (clinic* adj trial*).tw. or ((singl* or doubl* or treb* or tripl*) adj (blind$3 or mask$3)).tw. or Placebos/ or placebo*.tw.) not (animals/ not humans/) (2004752)  6 Epidemiologic studies/ or case control studies/ or exp cohort studies/ or Controlled Before-After Studies/ or Case control.tw. or (cohort adj (study or studies)).tw. or Cohort analy$.tw. or (Follow up adj (study or studies)).tw. or (observational adj (study or studies)).tw. or Longitudinal.tw. or Retrospective*.tw. or prospective*.tw. or consecutive*.tw. or Cross sectional.tw. or Cross-sectional studies/ or historically controlled study/ or interrupted time series analysis/ [Onder exp cohort studies vallen ook longitudinale, prospectieve en retrospectieve studies] (3477525)  7 3 and 4 (49)  8 (3 and 5) not 7 (87)  9 (3 and 6) not (7 or 8) (115)  10 7 or 8 or 9 (251) |

Table 6.1 Literature search

|  | **Embase** | **OVID/MEDLINE** | **Deduplicated** |
| --- | --- | --- | --- |
| SRs | 49 | 49 | 59 |
| RCT | 120 | 87 | 140 |
| Observational | 90 | 115 | 142 |
| Total | 259 | 251 | 341 |

Table 6.2 Search results

| **Author and year** | **Reason for exclusion** |
| --- | --- |
| Chen 2019 | Other PICO (too broadly formulated, relevant studies missing) |
| Cheng 2019 | Prospective cohort study with matched controls |
| Dragoo 2014 | PRP = invasive (other PICO question) |
| Dupley 2017 | PRP = invasive (other PICO question), no control group. |
| Everhart 2017 | Other PICO (too broadly formulated, relevant studies missing) |
| Furia 2012 | Retrospective study |
| Kaux 2016 | PRP = invasive (other PICO question) |
| Korakakis 2018 | Other PICO (too broadly formulated, relevant studies missing) |
| Larsson 2012 | Other PICO (too broadly formulated, relevant studies missing) |
| Mani-Babu 2015 | Other PICO (too broadly formulated, relevant studies missing) |
| Mendonça 2020 | Other PICO (too broadly formulated, relevant studies missing) |
| Pellecchia 1994 | PRP = invasive (other PICO question) |
| Scott 2019 | PRP = invasive (other PICO question) |
| Smitt 2014 | commentary on Vetrano 2013 |
| Stasinopoulos 2004 | Transferred friction = invasive (other PICO question), invalid controll group (exercise) |
| Rodriguez-Merchan 2013 | Not a systematic review, no relevant comparisons in conservative treatment |
| van der Doelen 2020 | Other PICO (too broadly formulated, relevant studies missing) |
| van der Worp 2011 | Study protocol |
| van der Worp 2014 | No valid control group |
| van Leeuwen 2009 | Other PICO (relevant studies seem missing) |
| Van Rijn 2019 | Secondary analyses of 3 RCTs |
| Ventrano 2013 | PRP = invasive (other PICO question) |
| Wang 2007 | Unclear conservative treatment (uncertain which treatment is provided to which patients or who received standard care) |
| Zayni 2015 | (-) PRP = invasive (other question) |

Table 6.3 Excluded studies

| **Study reference** | **Study characteristics** | **Patient characteristics ^2^** | **Intervention (I)** | **Comparison / control (C) ^3^** | **Follow-up** | **Outcome measures and effect size ^4^** | **Comments** |
| --- | --- | --- | --- | --- | --- | --- | --- |
| Lee, 2017 | Type of study:  RCT  Setting and country:  Outpatient clinic of a Chinese university.  Funding and conflicts of interest: N.A. | Inclusion criteria:  - diagnosis patellar tendinopathy  - VISA-P score <80  Exclusion criteria*:*  - Patellofemoral pain,  - Fat pad irritation,  - Meniscal injury,  - Osteoarthritis,  - Rheumatoid arthritis,  - History of lower limb fracture,  inflammatory myopathy,  - Previous patellar tendon cortisone  injection, or other interventions for PT  within 3 months.  N total at baseline:  Intervention: 17  Control: 17  Important prognostic factors^2^:  *Age ± SD*  I: 21.1 ± 2.2  C: 24.1 ± 4.6  *Sex*  I: 100% M  C: 100% M  *BMI (kg/m^2^) ± SD*  I: 22.9 ± 1.5  C: 23.1 ± 2.7  *VISA-P ± SD*  I: 55.1 ± 12.9  C: 57.4 ± 8.2  Groups comparable at baseline? No significant baseline differences. | ESWT:  Device (Minilith, Storz Medical,  Tägerwilen, Switzerland) was delivered to the most tender  region of the proximal patellar tendon with 30° flexion.  1500 impulses at 0.08mJ/mm^2^ to the level that subject could maximally tolerate, repeated weekly over 6 weeks.  Both groups (n=34) performed a *12-week* single-legged eccentric  decline squat exercise. | Sham-shockwave treatment (placebo):  Same device, 1500 impulses ≤0.08mJ/mm^2^, repeated weekly over 6 weeks. | Length of follow-up:  12 weeks  Loss-to-follow-up:  I: 1 withdraw (too busy to participate) and 1 lost contact.  C: 3 withdraw (too busy to participate) and 1 lost contact.  Incomplete outcome data:  28 patients included in analyses:  I: 16  C: 14 | Correlation between pain (VAS) *and change in tendon stiffness 12wk*  Intervention: 0.01  Control: 0.55  (between-group difference p>0.05)  Correlation between pain (VAS) *and change in tendon strain 12wk*  Intervention: 0.01  Control: -0.63  (between-group difference p>0.05)  Correlation between VISA-P *and change in tendon strain 12wk*  Intervention: 0.37  Control: -0.58  (between-group difference p>0.05)  Correlation between VISA-P *and change in tendon strain 12wk*  Intervention: -0.40  Control: 0.60  (between-group difference p>0.05) | Limitations:  - Relatively large variance of tendon force and stiffness  among subjects. Perhaps due to a wide subject age and small sample size.  - Short follow-up.  - The intention-to-treat principle with the last observation carried forward was used for missing data.  Conclusions:  - Increase in tendon strain was not  enhanced by the addition of ESWT.  Comments:  - No raw comparison between I and C; only in association with tendon strain/stiffness. |
| Thijs, 2017 | Type of study:  RCT  Setting and country:  Sports medicine department of university hospital and general hospital (n=2), the Netherlands.  Funding and conflicts of interest: N.A. | Inclusion criteria:  - Aged 18-40 years;  - patellar tendinopathy diagnosed by sports medicine physicians based on:  (1) history of pain in patellar tendon or its insertion,  (2) palpation tenderness of patellar tendon or its insertion,  (3) >8wk symptoms,  (4) VISA-P score <80.  Exclusion criteria*:*  - Acute knee or patellar tendon injury;  - Chronic inflammatory joint disease;  -Immunosuppressive or corticosteroid medication in the last 6mo;  - Previous knee surgery;  - Local (corticosteroid) injection in knee past month;  - Contraindications ESWT (e.g., pregnancy, malignancy, coagulopathy);  - Participants who received ESWT before.  N total at baseline:  Intervention: 22  Control: 30  Important prognostic factors^2^:  *Age ± SD*  I: 30.5 ± 8.0  C: 27.3 ± 5.2  *Sex*  I: 63.3% M  C: 80.0% M  *BMI (kg/m^2^) ± SD*  I: 23.9 *±* 3.5  C: 23.4 *±* 2.4  *VISA-P ± SD*  I: 54.5 ± 15.4  C: 58.9 ± 14.6  Groups comparable at baseline? No significant baseline differences. | ESWT:  Device (Piezoelectric ESWT, Swiss PiezoClast; Electro Medical Systems, Switzerland) was placed on the most painful spot with knee extended.  1000 impulses at 0.2mJ/mm^2^, repeated 3 sessions at 1-week intervals.  Both groups (n=52) performed an eccentric exercise program  2x/day for 24wk. | Sham-shockwave treatment (placebo):  Same device, 1000 impulses ≤0.03mJ/mm^2^, repeated 3 sessions at 1-week intervals.  Transmission gel was applied between focusing pad-skin, but not between applicator and focusing pad (i.e., shockwaves were hardly conducted, ≤0.03mJ/mm^2^). | Length of follow-up:  (6, 12) 24wk  Loss-to-follow-up:  *Intervention: 7 (32%)*  (2 too busy to participate, 3 unclear, 1 no treatment effect, 1 corticosteroid injection)  *Control: 4 (13%)*  (1 too busy to participate, 2 unclear, 1 PRP injection)  Incomplete outcome data:  Missings/incomplete data not included in initial analyses. | VISA-P ± SD  Intervention: 70.9 ± 17.7  Control: 78.2 ± 15.8  (between-group difference VISA-P -4.8; 95%CI -12.7 to 3.0, p=0.150).  Pain (NRS): 10 decline  squats  Intervention: 1.8 ± 1.8  Control: 2.2 ± 2.3  (Between-group difference 0.4, 95%CI -1.0 to 1.9)  Pain (NRS): 3 single leg  jumps  Intervention: 1.8 ± 1.8  Control: 1.9 ± 1.9  (Between-group difference -0.2, 95%CI -1.6 to 1.2)  Pain (NRS): 3 maximal  vertical jumps  Intervention: 1.6 ± 1.9  Control: 1.5 ± 1.9  (Between-group difference 1.2, 95%CI -0.2 to 2.5)  *Only at 6 weeks there was a significant difference found during 3 maximal vertical jumps, in favour of the sham-shockwave group (between group difference 2.2, 95%CI 0.9 to 3.4).  Recovery (Likert scale):  No significant differences between both groups at 24wk (p=0.928).  Intervention/control  1: completely recovered — 2/5  2: much better — 8/13  3: a little better — 2/5  4: unchanged — 2/2  5: worse — 1/1  6: much worse – 0/0 | Limitations:  - Relatively large LTFU.  - Tests performed by two unblinded caregivers.  Conclusions:  No additional effect of ESWT over placebo (sham shockwave) in patellar tendinopathie patients who were following a daily eccentric exercise program.  Comments:  - Complete case analysis or imputation of the missing data did not alter the  outcome of the study.  - All participants performed  eccentric exercises during the study. |

Table 6.4 Evidence table

**ESWT = Extracorporeal shockwave therapy; LIPUS = Low-intensity pulsed ultrasound; US = ultrasonography; VISA-P = Victorian Institute of Sports Assessment questionnaire Patella. *Three functional tests: (1) single-leg decline squat (1× and 10×) on a platform with a 20° slope and 60° knee flexion; (2) vertical jump test; (3) triple-hop test. ^$^Calculated based on Height (1.79, 180) and Mass (81, 82) in article. ^&^Calculated based on Height (181.6, 181.6) and Mass (80.1, 78.3) in article**

| **Study reference**  (first author, publication year) | **Describe method of randomisation^1^** | **Bias due to inadequate concealment of allocation?^2^**  (unlikely/likely/unclear) | **Bias due to inadequate blinding of participants to treatment allocation?^3^**  (unlikely/likely/unclear) | **Bias due to inadequate blinding of care providers to treatment allocation?^3^**  (unlikely/likely/unclear) | **Bias due to inadequate blinding of outcome assessors to treatment allocation?^3^**  (unlikely/likely/unclear) | **Bias due to selective outcome reporting on basis of the results?^4^**  (unlikely/likely/unclear) | **Bias due to loss to follow-up?^5^**  (unlikely/likely/unclear) | **Bias due to violation of**  **intention to treat analysis?^6^**  (unlikely/likely/unclear) |
| --- | --- | --- | --- | --- | --- | --- | --- | --- |
| **Extracorporeal shockwave therapy (ESWT)** | | | | | | | | |
| Lee, 2017 | Unclear  Not reported: “Subjects with patellar tendinopathy were randomly assigned to a group” | Unclear  Not reported. | Unclear  Not reported. | Unclear  Not reported. | Unclear  Not reported. | Unlikely  Methods section corresponds to results section. | Unlikely  LTFU: 2 ESWT, 4 placebo (no sign. baseline differences). | Likely  Missing data inadequately handled (intention-to-treat; last observation carried  forward for missings). |
| Thijs, 2017 | Unlikely  Simple procedure (sealed identical nonopaque envelopes containing “A” or “B” on it) | Unclear  Unclear whether envelops are numbered conform Cochrane guidelines (seems easy to assess which treatment is A or B) | Unlikely  Allocation info was withheld from participants and outcome assessors during study. | Likely  Care providers (physical therapists) providing  treatments knew the treatment. | Unlikely  Allocation information was withheld from participants and outcome assessors during study. | Unlikely  Clear methods section. | Likely  LTFU: 32% ESWT, 13% placebo (no sign. differences baseline characteristics and VISA-P scores). | Likely  Missing data inadequately handled (complete-case analyses and imputation (last value carried forward). |
| Zhang, 2020 | Unlikely  Simple procedure (sealed identical nonopaque envelopes) | Unclear  Not stated in article. | Unlikely  Patients did not know the type of treatment. | Likely  Care providers (physical therapists) providing  treatments knew the treatment. | Likely  Care providers (physical therapists) providing  treatments knew, they provided treatment. | Unlikely  Outcomes described in methods section are presented in results section. | Unlikely  One session (no FU) | Unlikely  N=17 versus N=17. No statements made regarding missings. |
| Zwerver, 2011 | Unlikely  Computer-generated (team level) | Unclear  Computer-generated randomization (statistician knew) | Unlikely  Allocation information was withheld from athletes + assessors. | Likely  Physical therapists and statistician were informed about group allocation. | Unlikely  Allocation information was withheld from athletes + assessors. | Unlikely  Clear methods section. | Unlikely  92% completed measurements. N=5 LTFU had similar characteristics. | Unclear  Intention-to-treat analysis, no statements regarding missings. |
| **Low-intensity pulsed ultrasound (LIPUS)** | | | | | | | | |
| Warden, 2008 | Unlikely  Simple (blocks of 10) using computer generated tables. | Unlikely  1 investigator performed randomization: kept assignment scheme, provided blinded assessor with US codes. | Unlikely  Outcome assessor and participants were blinded during study. | Unlikely  Outcome assessor, statistician, investigator performing measures, examiners, data manager, were blinded during study. | Unlikely  Outcome assessor and participants were blinded during study. | Unlikely  Clear methods section. | Unlikely  N=10 of 37 missing (4 versus 6). | Likely  ITT and PP analyses performed; missing data were replaced group means. |
| **Patellar strap and sports tape** | | | | | | | | |
| De Vries, 2016 | Likely  Random (dependent on entry in the study), after stratification for severity of injury. | Unclear | Likely  Control (no intervention), placebo (disfunc leukotape), patellar strap, sports tape (func leukotape). | Likely  Probably unblinded, no statements regarding blinding. | Likely  Probably unblinded, no statements regarding blinding. | Unlikely  Clear methods section. | Unlikely  Week 1 (97) / week 2 (69). Week 1 -> Week 2; excl PRP n=4? | Unclear  Unclear if/how incomplete data was handled. Week 2 LTFU seem not included in analyses. |

Table 6.5 Risk of bias table for intervention studies (randomized controlled trials)

**1. Randomisation: generation of allocation sequences have to be unpredictable, for example computer generated random-numbers or drawing lots or envelopes. Examples of inadequate procedures are generation of allocation sequences by alternation, according to case record number, date of birth or date of admission.**

**2. Allocation concealment: refers to the protection (blinding) of the randomisation process. Concealment of allocation sequences is adequate if patients and enrolling investigators cannot foresee assignment, for example central randomisation (performed at a site remote from trial location) or sequentially numbered, sealed, opaque envelopes. Inadequate procedures are all procedures based on inadequate randomisation procedures or open allocation schedules.**

**3. Blinding: neither the patient nor the care provider (attending physician) knows which patient is getting the special treatment. Blinding is sometimes impossible, for example when comparing surgical with non-surgical treatments. The outcome assessor records the study results. Blinding of those assessing outcomes prevents that the knowledge of patient assignement influences the proces of outcome assessment (detection or information bias). If a study has hard (objective) outcome measures, like death, blinding of outcome assessment is not necessary. If a study has “soft” (subjective) outcome measures, like the assessment of an X-ray, blinding of outcome assessment is necessary.**

**4. Results of all predefined outcome measures should be reported; if the protocol is available, then outcomes in the protocol and published report can be compared; if not, then outcomes listed in the methods section of an article can be compared with those whose results are reported.**

**5. If the percentage of patients lost to follow-up is large, or differs between treatment groups, or the reasons for loss to follow-up differ between treatment groups, bias is likely. If the number of patients lost to follow-up, or the reasons why, are not reported, the risk of bias is unclear.**

**6. Participants included in the analysis are exactly those who were randomized into the trial. If the numbers randomized into each intervention group are not clearly reported, the risk of bias is unclear; an ITT analysis implies that (a) participants are kept in the intervention groups to which they were randomized, regardless of the intervention they actually received, (b) outcome data are measured on all participants, and (c) all randomized participants are included in the analysis.**

# Summary of literature

Six studies answered the search question. As different types of interventions were evaluated in these studies, we divided this summary in three parts:

- Extracorporeal shockwave therapy (ESWT).
- Low-intensity pulsed ultrasound (LIPUS).
- Patellar strap and sports tape.

None of the studies evaluated the effectiveness of foot orthoses, percutaneous electrolysis therapy, or dry needling.

# Extracorporeal shockwave therapy (ESWT)

We found four RCTs that studied the effectiveness of ESWT (Lee, 2017; Thijs, 2017; Zhang, 2020; Zwerver, 2011).

Lee (2017) performed an RCT which compared ESWT to sham-shockwave therapy (placebo) in 34 male in-season athletes with PT. ESWT consisted of 1500 impulses at 0.8 mJ/mm^2^, increasing to the level that the subject could maximally tolerate, at 4 Hz during 6 sessions (weekly interval). Placebo consisted of 1500 impulses at < 0.8 mJ/mm^2^ at 4 Hz during 6 sessions (weekly interval), at this dosage subjects did not report any pain. In the ESWT-group were 16 participants (age 21.1 ± 2.2 years, 16M) and in the placebo-group were 14 participants (age 24.1 ± 4.6 years, 14M). Additionally, all subjects performed a 12-week single-legged eccentric decline squat exercise.

The follow-up was 12 weeks. Authors assessed function (VISA-P) and pain during activity (VAS). The intention-to-treat principle with the last observation carried forward was used for missing data.

Thijs (2017) performed a multicentre RCT which compared ESWT to sham-shockwave therapy (placebo) in physically active participants with PT. ESWT consisted of 1000 impulses at 0.2 mJ/mm^2^ at 4 Hz during 3 sessions (weekly interval). ESWT or placebo was administered 1x/week for a period of three weeks. Both groups performed an additional eccentric exercise program 2x/day for 24 weeks. In the ESWT-group were 22 physically active participants (age 30.5 ± 8.0 years, 14M/8F) and in the placebo-group were 30 participants (age 27.3 ± 5.2 years, 24M/6F). Authors assessed function (VISA-P), pain (NRS), patient recovery (6-point Likert scale; 1: completely recovered to 6: much worse) at baseline and at 6, 12, and 24 weeks after the start with the ESWT or placebo. During follow-up, 7 athletes (31.8%) in the ESWT group and 4 athletes (13.3%) in the placebo group were lost to follow-up. Although there was a relatively large loss to follow-up, imputation of missing data (according to intention-to-treat principle, mean value substitution and last value carried forward), did not alter the VISA-P outcome.

Zwerver (2011) performed a multicentre RCT which compared ESWT to sham-shockwave therapy (placebo) in athletes with PT. ESWT consisted of 2000 impulses (0.1 to 0.58 mJ/mm^2^) at 4 Hz during 3 sessions (weekly interval). The treatment procedure for the athletes in the placebo group was the same, except no transmission gel was applied between the applicator and focusing pad. No other exercises or treatments were administered during the study period. A total of 62 patients were included, 31 were allocated to the intervention group (age 24.2 ± 5.2 years, 20M/11F) and 31 were allocated to the control group (age 25.7 ± 4.5 years, 21M/10F). Authors assessed function (VISA-P) and pain (VAS) at 1, 12, and 22 weeks after treatment. No statements made regarding missing values.

Zhang (2020) conducted an RCT in which male athletes with PT were included, and one session ESWT was compared with one session sham-shockwave therapy (placebo). ESWT consisted of 1500 impulse at 0.13-0.33 mJ/mm^2^ during a single session. In the sham-shockwave group the treatment intensity was standardized at < 0.08 mJ/mm^2^. Both groups included 17 participants (intervention: age 21.1 ± 2.2 years; control: age 23.2 ± 4.7 years). Authors assessed, among others, activity-related pain (VAS). This study measured the effectiveness of ESWT in only one session, which is too short to assess the long-term effectiveness.

# Results

Pain

All four RCTs (Lee, 2017; Thijs, 2017; Zwerver, 2011, Zhang, 2020) assessed pain, but the methods, time of measurements, and measurement circumstances differed between studies. Due to heterogeneity in the studies and interventions it was not possible to pool these data. Based on these studies no statistically significant nor clinically relevant differences were detected in pain scores between ESWT and placebo.

Lee (2017) measured pain using VAS (0=no pain to 10=worst pain) to assess the association between the change in tendon stiffness and with tendon pain. In the ESWT group the pain decreased from 6.7 ± 1.9 to 3.9 ± 1.9 whereas the placebo group decreased from 6.6 ± 2.0 to 3.2 ± 2.5 at 12 weeks follow-up (between-group difference at 12 weeks p>0.05).

Thijs (2017) measured pain during three types of functional knee tests, as rated verbally by NRS for pain (0=no pain to 10=worst pain ever). No statistically significant nor clinically relevant differences were found between the pain levels during the functional tests of the ESWT and the placebo groups in 24 weeks (except for pain during 3 maximal vertical jumps at 6 weeks, in favour of the sham-shockwave group). After 24 weeks, the largest between-group difference in change scores was found for the three maximal vertical jumps. In the ESWT group the NRS decreased from 2.8 ± 2.9 to 1.6 ± 1.9 at 24 weeks follow-up, whereas the placebo group decreased from 3.8 ± 2.4 to 1.5 ± 1.9 at 24 weeks follow-up (between-group difference at 24 weeks: 1.2, 95%CI -0.2 to 2.5).

Zwerver (2011) measured pain using the VAS (0=no pain to 10=worst pain). No statistically significant nor clinically relevant differences were found between the ESWT and the placebo group. During sports, the ESWT group decreased from 4.9 ± 2.3 to 3.2 ± 2.7 at 22 weeks follow-up, whereas the placebo group decreased from 4.6 ± 2.3 to 4.0 ± 3.0 at 22 weeks follow-up (between-group difference at 22 weeks: -1.0, 95%CI -2.6 to 0.6). Similar outcomes were detected after 1, 12, and 22 weeks.

Zhang (2020) measured “activity pain” (single-leg declined-squat test) using the VAS (0=no pain to 10=worst pain). No statistically significant nor clinically relevant differences were found between the ESWT and the placebo group. The ESWT group decreased from 5.1 ± 1.6 to 4.5±1.7, whereas the placebo group decreased from 5.2 ± 2.0 to 4.3 ± 2.0.

Function

Three studies (Lee, 2017; Thijs, 2017; Zwerver, 2011) assessed function with the VISA-P Questionnaire (0 to 100, with 100 representing optimal health). Due to heterogeneity in the studies and interventions it was not possible to pool these data. Based on these studies, no statistically significant nor clinically relevant differences in VISA-P scores between ESWT and placebo were detected.

Lee (2017) measured function using VISA-P to assess the association between the change in tendon stiffness and with dysfunction. In the ESWT group the VISA-P increased from 55.1 ± 12.9 to 72.9 ± 14.3 whereas the placebo group increased from 57.4 ± 8.3 to 77.3 ± 12.6 at 12 weeks follow-up (between-group difference at 12 weeks p>0.05).

Thijs (2017) reported that the change in VISA-P scores during the follow-up period was similar in both groups. In the ESWT group the VISA-P increased from 54.5 ± 15.4 to 70.9 ± 17.8 at 24 weeks follow-up, whereas the placebo group increased from 58.9 ± 14.6 to 78.2 ± 15.8 at 24 weeks follow-up (between-group difference at 24 weeks: -4.8, 95%CI -12.7 to 3.0, p= 0.150).

Zwerver (2011) reported that the change in VISA-P scores during the follow-up period was similar in both groups. In the ESWT group the VISA-P score increased from 59.4 ± 11.7 to 70.5 ± 18.9, whereas the placebo group increased from 62.4 ± 13.4 to 72.7 ± 18.0 (between-group difference at 22 weeks: 0.7, 95%CI -8.0 to 9.4, p=0.82).

Zhang (2020) did not include this outcome.

Patient recovery

Thijs (2017) measured patient recovery with a 6-point Likert scale (1: completely recovered to 6: much worse). Authors reported a non-statistically significant between-group difference at 24 weeks (p=0.928). In the ESWT group 67% reported good outcomes (i.e. much better or completely recovered), and in the sham-shockwave group 69% reported good after 24 weeks.

Lee (2017), Zwerver (2011), and Zhang (2020) did not include this outcome.

Return to sports/work, patient satisfaction, duration of absenteeism

Lee (2017), Thijs (2017), Zwerver (2011), and Zhang (2020) did not include these outcomes.

Level of evidence of the literature

The level of evidence regarding the outcome ‘pain’ started at high as it was based on RCTs, but was downgraded by two levels because study limitations (risk of bias, -1) and relatively small number of included patients (imprecision, -1). The final level is low.

The level of evidence regarding the outcome ‘function’ started at high as it was based on RCTs, but was downgraded by two levels because of study limitations (risk of bias, -1) and relatively small number of included patients (imprecision, -1). The level of evidence was assessed as low.

The level of evidence regarding the outcome ‘recovery’ started at high as it was based on one RCT, but was downgraded by three levels because of study limitations (risk of bias, -1) and the inclusion of a single study with a limited number of included patients (imprecision, -2). The level of evidence was assessed as very low.

The level of evidence regarding the outcomes ‘return to sports/work’, ‘patient satisfaction’, and ‘duration of absenteeism’ were not assessed due to lack of data.

# Conclusions

Pain (critical)

| **Low**  **GRADE** | ESWT in patients with PT may result in little to no difference in pain, compared to placebo treatment.  *Sources: (Lee, 2017; Thijs, 2017; Zwerver, 2011; Zhang, 2020)* |
| --- | --- |

Function (critical)

| **Low**  **GRADE** | ESWT in patients with PT may result in little to no difference in function, compared to placebo treatment.  *Sources: (Lee, 2017; Thijs, 2017; Zwerver, 2011)* |
| --- | --- |

Patient recovery (important)

| **Very low**  **GRADE** | It is unclear what the effect of ESWT is on patient recovery, compared to placebo treatment in patients with PT.  *Sources: (Thijs, 2017)* |
| --- | --- |

Return to sport/work (important), patient satisfaction (important), duration of absenteeism (important)

| **-**  **GRADE** | The outcome measure ‘return to sports/work’, ‘patient satisfaction’, and ‘duration of absenteeism’ was not reported in the included studies. |
| --- | --- |

# Low-intensity pulsed ultrasound (LIPUS)

We found one RCT (Warden, 2008) that studied the effectiveness of LIPUS. Warden (2008) performed an RCT which compared active-LIPUS to inactive-LIPUS (placebo) in volunteers with clinically and radiologically confirmed PT. LIPUS was self-administered by patients for 20 min/day, 7 days/week for 12 weeks. It consisted of 2-ms burst of 1.0 MHz with a frequency of 100 Hz and was applied during sitting with 90° knee flexion. Patients in the inactive-LIPUS group received identical appearing ultrasound units but the units produced no actual ultrasound output. In the LIPUS-group were 17 participants (age 27 ± 7 years, 12M/5F) and in the placebo-group were 20 participants (age 27 ± 7 years, 18M/2F). The follow-up was 24 weeks and both groups performed a standardized eccentric exercise program (conform current practice) during the study period. Outcome measures included pain (VAS-Usual; VAS-Worst), function (VISA-P), and patient-perceived response to treatment (5-point Likert scale). Missing data in the active- and inactive-LIPUS groups were replaced by the inactive- and active-LIPUS group means, respectively. For the global change outcome, all missing data were replaced by a score of 3 (‘no change’).

# Results

Pain

Warden (2008) measured pain on VAS for both usual (VAS-U) and worst (VAS-W) tendon pain. No statistically significant nor clinically relevant differences in VAS scores were detected. Regarding VAS-U, the LIPUS group decreased from 5.2 ± 2.5 to 3.8 ± 2.3, whereas the placebo group decreased from 5.6 ± 2.4 to 3.9 ± 2.2 (between-group change: -0.2 (95%CI -1.7 to 1.3, p=0.82). Regarding VAS-W, the LIPUS group decreased from 6.8 ± 2.4 to 4.1 ± 2.5, whereas the placebo group decreased from 6.8 ± 2.3 to 4.5 ± 2.6 (between-group change: -0.4 (95%CI -2.1; 1.3, p=0.65).

Function

Warden (2008) assessed function with the VISA-P and did not detect statistically significant nor clinically relevant difference between LIPUS and placebo at 12 weeks. Authors showed that in the LIPUS group the VISA-P increased from 58.1 ± 15.9 to 69.8 ± 14.5, whereas the placebo group increased from 56.6 ± 18.5 to 69.2 ± 17.9 (between-group change: -0.7, 95%CI -10.3 to 11.7, p=0.90).

Patient recovery

Warden (2008) assessed patient recovery by asking participants about the patient-perceived response to treatment (5-point Likert scale). Authors did not detect statistically significant difference between LIPUS and placebo. Overall, 11 out of 13 LIPUS-participants and 9 out of 14 placebo-participants felt that they had improved after the treatment (p=0.23).

Return to sports/ work, patient satisfaction, duration of absenteeism

Warden (2008) did not include these outcomes.

Level of evidence of the literature

The level of evidence regarding the outcome ‘pain’ started at high as it was based on an RCT but was downgraded by three levels because of study limitations (risk of bias, -1) and the inclusion of a single study with a limited number of included patients (imprecision, -2). The level is very low.

The level of evidence regarding the outcome ‘function’ started at high as it was based on an RCT but was downgraded by three levels because of study limitations (risk of bias, -1) and the inclusion of a single study with a limited number of included patients (imprecision, -2). The level of evidence was assessed as very low.

The level of evidence regarding the outcome ‘patient recovery’ started at high as it was based on an RCT but was downgraded by three levels because of study limitations (risk of bias, -1) and the inclusion of a single study with a limited number of included patients (imprecision, -2). The level of evidence was assessed as very low.

The level of evidence regarding the outcomes ‘return to sports/work’, ‘patient satisfaction’, and ‘duration of absenteeism’ were not assessed due to lack of data.

# Conclusions

Pain (critical)

| **Very low**  **GRADE** | It is unclear what the effect of LIPUS is on pain in patients with PT, compared to placebo treatment.  *Sources: (Warden, 2008)* |
| --- | --- |

Function (critical)

| **Very low**  **GRADE** | It is unclear what the effect of LIPUS is on function in patients with PT, compared to placebo treatment.  *Sources: (Warden, 2008)* |
| --- | --- |

Patient recovery (important)

| **Very low**  **GRADE** | It is unclear what the effect of LIPUS is on patient recovery, compared to placebo treatment in patients with PT.  *Sources: (Warden, 2008)* |
| --- | --- |

Return to sport/work (important), patient satisfaction (important), duration of absenteeism (important)

| **-**  **GRADE** | The outcome measure ‘return to sports/work’, ‘patient satisfaction’, and ‘duration of absenteeism’ was not reported in the included studies. |
| --- | --- |

# Patellar strap and sports tape

We found one RCT that studied the effectiveness of patellar strap and sports tape (de Vries, 2016). De Vries (2016) performed a multicentre RCT to compare four conditions (patellar strap, sports tape, placebo, and control) in physically active participants with PT. The first week was the baseline week for all participants (n=97, no intervention during sports) and the second week was the intervention week where 25% used a patellar strap during sports (n=21, age 26.3 ± 8.4, 12M/9F), 25% used sports tape (n=18, age 28.2 ± 9.3, 11M/7F), 25% used the placebo tape (n=16, 25.1 ± 7.9, 9M/7F), and 25% used no intervention during sports (n=14, age 30.0 ± 7.6, 5M/9F). There was no long-term follow-up carried out in this study (i.e. 2 weeks follow-up). Outcome measures included pain (VAS) during sports and 2h after sports ranging between 0 (no pain) and 100 mm (most severe pain).

# Results

Pain

De Vries (2016) measured pain using a VAS scale during sports and 2h after sports. The use of sports tape decreased pain significantly between both weeks (during sports difference: -7mm, p=0.04; 2h after sports difference: -10mm, p=0.001). The use of patellar straps (difference: -8mm, p<0.001) and placebo (difference: -7mm, p=0.03) decreased pain significantly 2h after sports compared to the control week. Interestingly, also the use of placebo tape decreased pain significantly during sports (difference: -6mm, p=0.04) and 2h after sports (difference: -7mm, p=0.03) compared to the control week. However, the differences between the groups were considered not clinically relevant.

Function, return to sports/work, patient satisfaction, patient recovery, duration of absenteeism

De Vries (2016) did not include these outcomes.

Level of evidence of the literature

The level of evidence regarding the outcome ‘pain’ started at high as it was based on an RCT, but was downgraded by 3 levels because of study limitations (risk of bias, -1) and the inclusion of a single study with a limited number of included patients (imprecision, -2). The level is very low.

The level of evidence regarding outcomes ‘function’, ‘return to sports/work’, ‘duration of absenteeism’, ‘patient satisfaction’, and ‘patient recovery’ were not assessed due to lack of data.

# Conclusions

Pain (critical)

| **Very low**  **GRADE** | It is unclear what the effect of patellar strap and sports tape is on pain in patients with PT, compared to placebo treatment.  *Sources: (De vries, 2016)* |
| --- | --- |

Function (critical), return to sports/work (important), patient satisfaction (important), patient recovery (important), and duration of absenteeism (important)

| **-**  **GRADE** | The outcome measure ‘function’, ‘return to sports/work’, ‘patient satisfaction’, ‘patient recovery’, ‘duration of absenteeism’ was not reported in the included study. |
| --- | --- |

# Considerations - from evidence to recommendation

Extracorporeal shockwave therapy (ESWT)

Based on the literature, treatment with ESWT, in addition to exercise therapy, does not appear to have a clinically relevant effect on the crucial outcome measures pain and function and the important outcome measure recovery. The low to very low strength of evidence is mainly caused by limitations in the study design and small numbers of patients. There is therefore a knowledge gap here. A number of (relative) contraindications to the use of ESWT do exist: pregnant women, tumors, open growth plates and osteomyelitis (Eid, 2016). Given the potential (relative) contraindication of shockwave in lithotripsy when using anticoagulant medications and platelet inhibitors, this should be included when considering the use of shockwave (Schnabel, 2014).

Key outcome measures return to sport/work and satisfaction cannot further contribute to the direction of decision making. Regarding these outcome measures, no literature was included in the abstract.

Low-intensity pulsed ultrasound (LIPUS)

Based on the literature, treatment with LIPUS, in addition to exercise therapy, does not appear to have a clinically relevant effect on the crucial outcome measures pain and function. The very low strength of evidence is mainly caused by the fact that only 1 RCT was found, limitations in study design and small numbers of patients. Key outcome measures return to sport/work, satisfaction and recovery could not contribute further to the direction of decision making. Regarding these outcome measures, no literature was included in the abstract.

Taping (straps and tape)

Based on the literature, treatment with tape, in addition to exercise therapy, does not seem to have a clinically relevant effect on the crucial outcome measure pain. The low to very low strength of evidence is mainly caused by the fact that only 1 RCT was found, limitations in the study design and small numbers of patients. This RCT only determined the immediate effect of tape on pain and did not look at mid- or long-term effects. Therefore, there is a knowledge gap here. The crucial outcome measure function and the important outcome measures return to sport/work, satisfaction and recovery cannot further contribute to the direction of decision making. Regarding these outcome measures, no literature was included in the summary.

Foot orthoses, percutaneous electrolysis therapy (EPTE) and dry needling

No RCTs or reviews were found on other additional conservative treatments, such as foot orthoses, EPTE and dry needling, which address the predefined crucial and important outcome measures. Since the expert panel notes that these interventions are regularly used in practice, we will briefly discuss them.

In an observational study, Williams (2001) found more PT in runners with a lower arch compared to runners with a higher arch. It is possible that this is related to a greater maximum knee flexion angle and thus a greater quadriceps force to counteract it. By using foot orthoses, one could theoretically compensate for this biomechanical effect and thus affect symptoms. Morgan's (2018) review also identifies foot morphology and leg length difference as possible risk factors. These could also potentially be influenced by foot orthoses. However, no RCT has yet been conducted on the effect of such foot orthoses on pain or function in PT.

Percutaneous electrolysis therapy (EPTE) is a relatively new treatment. The expert panel is aware of only one study of very low level of evidence on the effect of EPTE in PT (Abat, 2015). This is an observational study where a significant improvement in VISA-P was found after three months. To date, no RCT has been performed. Little else is known about possible side effects of the treatment. Based on the current evidence, EPTE cannot be recommended; more research is needed.

Dry needling of the quadriceps or surrounding muscles may be considered for stiffness of these muscles. Flexibility of the quadriceps and hamstrings (Sprague, 2018) and increased muscle tension of the quadriceps (Zhang, 2017) are related to PT. By reducing stiffness, lower stress on the patellar tendon could be achieved. No RCTs were found on the effect on pain and function in PT.

Summary

In summary, the evidence in the literature regarding the added value of additional interventions besides exercise therapy for PT is very limited. The number of RCTs and systematic reviews is very low (n=6) and they cover three different interventions. Multiple studies (n=4) were found only for shockwave therapy. All in all, no preferred intervention can be identified based on the literature. If shockwave treatment is chosen, the expert panel recommends, based on the application in the available literature (1-6x) and the opinion of the expert panel, to try a maximum of 6 treatment sessions with shockwave and if this does not improve, to stop shockwave.

The RCTs on the three interventions (ESWT, LIPUS and patellar strap) do not describe any side effects or adverse effects. To the knowledge of the expert panel, no adverse effects of these interventions are reported in other studies not included in this guideline either. Skin allergy to tape treatment however is a known side effect.

Expectations and values of patients (and possibly their caretakers)

To gain more insight into the values and expectations of patients with PT, a questionnaire was prepared and distributed in collaboration with the Netherlands Patients Federation (NPF). The questionnaire was completed by nine PT patients (three patients who have had it and six patients who currently have it). Patients would prefer to be able to function normally again (as soon as possible) in daily life; in addition, resuming work and being able to perform their sports load as before are considered important.

Patients indicated that they were generally well and fully informed about the possible additional interventions. It is important to discuss these interventions in the context of the scientific rationale and its limitations.

In the responses to this module, a number of advantages and disadvantages are mentioned by the patients with regard to additional treatment. The advantages noted are mainly short-term positive effects. The disadvantages that weigh heavily for patient are the costs involved and possible (temporary) increase in pain.

Cost

To the knowledge of the expert panel, no cost-effectiveness studies have been conducted on conservative treatments for PT. The conservative treatments such as ESWT, dry needling, taping, EPTE and LIPUS will not incur additional costs for the patient when used during a physical therapy session. However, the direct costs for these sessions will be for the patient. If the patient has supplemental health insurance and the physical therapist provides contracted care, the physical therapy will be at the expense of the supplemental health insurance. Foot orthoses and patellar straps will involve (one-time) costs for the patient. Overall, the cost of tape/straps and foot orthoses is low (€20 to €50). Fitted foot orthoses are usually more expensive (ranging from €100 to €250). Although the costs are relatively low and may be partially covered, the effect of complementary treatments is unclear. From this standpoint, the patient should make a personal assessment before starting complementary treatment(s).

Acceptability, feasibility and implementation

Based on the limited current literature on additional conservative treatments, these treatments cannot be recommended. In the absence of results from initial exercise therapy, additional conservative treatment options may be considered in combination with exercise therapy for possible effect. Additional conservative treatments involve limited costs. Together with the fact that no side effects or adverse effects of these conservative treatments have been described, their use may be considered for possible effect.

# Rationale of the recommendations

There is a lack of evidence for effectiveness of additional conservative treatments in PT. Informing the patient is important. Initially, the expert panel advises against the use of additional conservative treatments. No intervention can be designated as preferred the literature. Since no side effects of these additional conservative treatments have been reported, if there is no improvement of the symptoms, after consistent exercise therapy, the additional conservative treatments can be considered in combination with exercise therapy for a possible effect. The expert panel prefers a patellar strap because of its low cost and low-threshold application.

No side effects or adverse effects are described in the RCTs on the three interventions (ESWT, LIPUS and patellar strap).

Patients indicate as important to be able to function normally in daily life, work and the sports (as soon as possible). Benefits as seen by patients are the short-term positive effects. The disadvantages are the costs involved and any (temporary) increase in pain. Informing the patient about the lack of evidence this is important.

# References

Abat F, Gelber PE, Polidori F, Monllau JC, Sanchez-Ibañez JM. (2015) Clinical results after ultrasound-guided intratissue percutaneous electrolysis (EPI®) and eccentric exercise in the treatment of patellar tendinopathy. *Knee Surg Sports Traumatol Arthrosc.*;23(4):1046-52.

de Vries A, Zwerver J, Diercks R, Tak I, van Berkel S, van Cingel R, van der Worp H, van den Akker-Scheek I. (2016). Effect of patellar strap and sports tape on pain in patellar tendinopathy: A randomized controlled trial. *Scand J Med Sci Sports*;26(10):1217-24. doi: 10.1111/sms.12556. Epub 2015 Sep 17. PMID: 26376953.

Eid, J. (2016). Consensus statement on ESWT indications and contraindications. *Int. Soc. Med. Shockwave Treat.(ISMST)*, 1-4.

Lee WC, Ng GY, Zhang ZJ, Malliaras P, Masci L, Fu SN. (2020). Changes on Tendon Stiffness and Clinical Outcomes in Athletes Are Associated With Patellar Tendinopathy After Eccentric Exercise. *Clin J Sport Med*;30(1):25-32.

Morgan S, Coetzee FF. (2018). Proposing a Patellar Tendinopathy Screening tool following a systematic review. *S Afr J Physiother*;74(1):454.

Schnabel MJ, Gierth M, Bründl J, Chaussy CG, Burger M, Fritsche HM. (2014). Antiplatelet and anticoagulative medication during shockwave lithotripsy. *J Endourol*;28(9):1034-9.

Sprague, A. L., Smith, A. H., Knox, P., Pohlig, R. T., & Silbernagel, K. G. (2018). Modifiable risk factors for patellar tendinopathy in athletes: a systematic review and meta-analysis. *British journal of sports medicine*, *52*(24), 1575-1585.

Thijs KM, Zwerver J, Backx FJ, Steeneken V, Rayer S, Groenenboom P, Moen MH. (2017). Effectiveness of Shockwave Treatment Combined With Eccentric Training for Patellar Tendinopathy: A Double-Blinded Randomized Study. *Clin J Sport Med*;27(2):89-96.

Warden SJ, Metcalf BR, Kiss ZS, Cook JL, Purdam CR, Bennell KL, Crossley KM. (2008). Low-intensity pulsed ultrasound for chronic patellar tendinopathy: a randomized, double-blind, placebo-controlled trial. *Rheumatology (Oxford)*;47(4):467-71.

Williams DS III, McClay IS, Hamill J. (2001). Arch structure and injury patterns in runners. *Clin Biomech* (Bristol, Avon);16:341-7.

Zhang ZJ, Lee WC, Fu SN. (2020). One Session of Extracorporeal Shockwave Therapy-Induced Modulation on Tendon Shear Modulus is Associated with Reduction in Pain. *J Sports Sci Med*;19(2):309-316.

Zhang, ZJ, Ng, GYF, Lee, WC, & Fu, SN. (2017). Increase in passive muscle tension of the quadriceps muscle heads in jumping athletes with patellar endinopathy. *Scandinavian journal of medicine & science in sports*, *27*(10), 1099-1104

Zwerver J, Hartgens F, Verhagen E, van der Worp H, van den Akker-Scheek I, Diercks RL. (2011). No effect of extracorporeal shockwave therapy on patellar tendinopathy in jumping athletes during the competitive season: a randomized clinical trial. *Am J Sports Med*;39(6):1191-9.

# Construction of Module 7 ‘Pain medications for PT’

Scoping Question: What is the value of pain medications, (including injection therapy) for patients with PT?

# Search and select

A systematic review of literature was performed to answer the following question:

What is the effect of a medical treatment, including injection therapy, in patients with PT on pain, function, return to sport/work, duration of absenteeism, patients’ satisfaction, and patient recovery?

P:	adult patients with PT;

I:	medical treatments, including injection therapy;

C:	control group/ placebo/ wait and see policy;

O:	pain, function, return to sports/ work, duration of absenteeism and patient satisfaction.

Relevant outcome measures

The expert panel considered pain and function as *critical* outcome measures for decision making; and return to sport/work, duration of absenteeism and patient satisfaction as *important* outcome measures for decision making.

The expert panel defined the outcome measures as following:

- Pain: as determined with the Visual Analogue Scale (VAS) or the Numeric Rating Scale (NRS).
- Function: as determined with the Victorian Institute of Sport Assessment-Patella (VISA-P) questionnaire, which is developed to measure severity of symptoms, pain, and function in patients with PT, or the Kujala score/ Anterior Knee Pain Score (AKPS) could also be used.
- Return to sport/work: as determined with the Tegner score.
- Patient satisfaction: as determined on a Likert scale.
- Patient recovery: as determined on a Likert scale.

A priori, the expert panel did not define the outcome measure ‘duration of absenteeism’ but used the definitions used in the studies.

The expert panel defined a minimal clinically important difference of 2 cm (out of 10 cm) change on the VAS or NRS scale, in accordance with Crossley (2004). Additionally, a difference of 13 points in the VISA-P score was the minimal clinically important difference (Hernandez-Sanchez, 2014). For the Kujala score/ AKPS score, a difference of 10 point (out of 100 points) was defined as a minimal clinically (patient) important difference, according to Crossley (2004). A minimal clinically important difference for return to sport/work, duration of absenteeism, patient satisfaction, and patient recovery was not predefined.

Search and select (Methods)

The databases Medline (via OVID) and Embase (via Embase.com) were searched with relevant search terms from 2000 until November 2020. The detailed search strategy is presented in the tables. The systematic literature search resulted in 200 hits. Studies were selected based on the following inclusion criteria:

- Systematic reviews (searched in at least two databases, detailed search strategy, risk of bias assessment, and results of individual studies available) or randomized controlled trials (RCTs).
- Answering the search question described above.
- > 1 month follow-up.

Initially, 31 studies were selected based on title and abstract screening. After reading the full text, 25 studies were excluded (Table 7.4). Eventually, one systematic review was included and two RCTs were included. To answer our clinical question, we distinguished between the short-term effects (after 12 weeks) and the long-term effects (after one year).

Results

Three studies were included in the analysis of the literature. Important study characteristics and results were summarized in the evidence tables. The risk of bias assessment is presented in the risk of bias tables.

| **No.** | **Query** | **Results** |
| --- | --- | --- |
| #11 | #8 OR #9 OR #10 | 159 |
| #10 | #4 AND #7 NOT (#8 OR #9) | 19 |
| #9 | #4 AND #6 NOT #8 | 86 |
| #8 | #4 AND #5 | 54 |
| #7 | 'major clinical study'/de OR 'clinical study'/de OR 'case control study'/de OR 'family study'/de OR 'longitudinal study'/de OR 'retrospective study'/de OR 'prospective study'/de OR 'cohort analysis'/de OR cohort*:ab,ti OR (('case control' NEAR/1 (study OR studies)):ab,ti) OR (('follow up' NEAR/1 (study OR studies)):ab,ti) OR (observational NEAR/1 (study OR studies)) OR ((epidemiologic NEAR/1 (study OR studies)):ab,ti) OR (('cross sectional' NEAR/1 (study OR studies)):ab,ti) | 5749128 |
| #6 | 'clinical trial'/exp OR 'randomization'/exp OR 'single blind procedure'/exp OR 'double blind procedure'/exp OR 'crossover procedure'/exp OR 'placebo'/exp OR 'prospective study'/exp OR rct:ab,ti OR random*:ab,ti OR 'single blind':ab,ti OR 'randomised controlled trial':ab,ti OR 'randomized controlled trial'/exp OR placebo*:ab,ti | 3159901 |
| #5 | 'meta analysis'/exp OR 'meta analysis (topic)'/exp OR metaanaly*:ti,ab OR 'meta analy*':ti,ab OR metanaly*:ti,ab OR 'systematic review'/de OR 'cochrane database of systematic reviews'/jt OR prisma:ti,ab OR prospero:ti,ab OR (((systemati* OR scoping OR umbrella OR 'structured literature') NEAR/3 (review* OR overview*)):ti,ab) OR ((systemic* NEAR/1 review*):ti,ab) OR (((systemati* OR literature OR database* OR 'data base*') NEAR/10 search*):ti,ab) OR (((structured OR comprehensive* OR systemic*) NEAR/3 search*):ti,ab) OR (((literature NEAR/3 review*):ti,ab) AND (search*:ti,ab OR database*:ti,ab OR 'data base*':ti,ab)) OR (('data extraction':ti,ab OR 'data source*':ti,ab) AND 'study selection':ti,ab) OR ('search strategy':ti,ab AND 'selection criteria':ti,ab) OR ('data source*':ti,ab AND 'data synthesis':ti,ab) OR medline:ab OR pubmed:ab OR embase:ab OR cochrane:ab OR (((critical OR rapid) NEAR/2 (review* OR overview* OR synthes*)):ti) OR ((((critical* OR rapid*) NEAR/3 (review* OR overview* OR synthes*)):ab) AND (search*:ab OR database*:ab OR 'data base*':ab)) OR metasynthes*:ti,ab OR 'meta synthes*':ti,ab | 682078 |
| #4 | #1 AND (#2 OR #3) AND ([english]/lim OR [dutch]/lim) AND [2000-2020]/py NOT ('conference abstract'/it OR 'editorial'/it OR 'letter'/it OR 'note'/it) | 378 |
| #3 | 'nonsteroid antiinflammatory agent'/exp OR 'lidocaine'/exp OR 'paracetamol'/exp OR ((('non steroid*' OR nonsteroid*) NEAR/2 ('anti inflammatry' OR antiinflammatory) NEAR/2 (drug* OR agent* OR compound*)):ti,ab,kw) OR lidocaine:ti,ab,kw OR paracetamol:ti,ab,kw OR acetaminophen*:ti,ab,kw OR 'diclofenac':ti,ab,kw OR voltaren:ti,ab,kw OR naproxen:ti,ab,kw OR ibuprofen:ti,ab,kw OR 'steroid'/exp OR steroid*:ti,ab,kw OR 'corticosteroid'/exp OR (((cortex OR cortical OR cortico OR adrenocortical) NEAR/2 (hormone* OR steroid*)):ti,ab,kw) OR corticoid*:ti,ab,kw OR corticosteroid*:ti,ab,kw OR cortisone:ti,ab,kw OR adrenocorticosteroid*:ti,ab,kw OR 'triamcinolone':ti,ab,kw OR kenacort:ti,ab,kw OR methylprednisolone:ti,ab,kw OR 'polidocanol'/exp OR 'polidocanol':ti,ab,kw OR 'glycosaminoglycan polysulfate'/exp OR 'glycosaminoglycan polysul*ate':ti,ab,kw OR 'hyaluronic acid'/exp OR 'hyaluronic acid':ti,ab,kw OR 'thrombocyte rich plasma'/exp OR 'platelet rich plasma':ti,ab,kw OR 'thrombocyte rich plasma':ti,ab,kw OR prp:ti,ab,kw OR 'autologous conditioned plasma':ti,ab,kw OR acp:ti,ab,kw OR 'autologous blood injection*':ti,ab,kw OR 'prolotherapy'/exp OR 'prolotherapy':ti,ab,kw OR 'proliferation therapy':ti,ab,kw OR 'regenerative injection therapy':ti,ab,kw OR 'stromal vascular fraction':ti,ab,kw OR svf:ti,ab,kw | 2586365 |
| #2 | 'drug therapy'/exp OR (((drug OR pharma* OR medicament OR medicinal) NEAR/2 (treatment* OR therap*)):ti,ab,kw) OR 'pharmaco therapy':ti,ab,kw OR 'pharmaco treatment':ti,ab,kw OR pharmacotherapy:ti,ab,kw OR pharmacotreatment:ti,ab,kw OR 'injection'/exp OR injection*:ti,ab,kw | 3772793 |
| #1 | 'patellar tendinopathy'/exp OR 'jumper* knee':ti,ab,kw OR 'patella apicitis':ti,ab,kw OR 'patellar apicitis':ti,ab,kw OR 'patella apex syndrome':ti,ab,kw OR 'patellar apex syndrome':ti,ab,kw OR 'patella tip syndrome':ti,ab,kw OR 'patellar tip syndrome':ti,ab,kw OR 'patella tenosynovitis':ti,ab,kw OR 'patellar tenosynovitis':ti,ab,kw OR (('patellar ligament'/exp OR 'patella'/exp OR 'patella':ti,ab,kw OR 'patellar':ti,ab,kw) AND ('tendinitis'/exp OR 'tendinopathy':ti,ab,kw OR 'tendinopathies':ti,ab,kw OR 'tendinosis':ti,ab,kw OR 'tendinoses':ti,ab,kw OR 'tendinitis':ti,ab,kw OR 'tendinitides':ti,ab,kw OR 'tendonitis':ti,ab,kw OR 'tendonitides':ti,ab,kw OR 'peritendinitis':ti,ab,kw OR 'paratenonitis':ti,ab,kw OR ('tendon':ti,ab,kw AND ('pathology':ti,ab,kw OR 'rupture':ti,ab,kw OR 'ruptures':ti,ab,kw)))) | 2856 |

Table 7.1 Embase search

| **#** | **Searches** | **Results** |
| --- | --- | --- |
| 12 | 9 or 10 or 11 | 140 |
| 11 | (5 and 8) not (9 or 10) | 31 |
| 10 | (5 and 7) not 9 | 67 |
| 9 | 5 and 6 | 42 |
| 8 | Epidemiologic studies/ or case control studies/ or exp cohort studies/ or Controlled Before-After Studies/ or Case control.tw. or cohort*.tw. or Cohort analy$.tw. or (Follow up adj (study or studies)).tw. or (observational adj (study or studies)).tw. or Longitudinal.tw. or Retrospective*.tw. or prospective*.tw. or consecutive*.tw. or Cross sectional.tw. or Cross-sectional studies/ or historically controlled study/ or interrupted time series analysis/ | 3705519 |
| 7 | exp clinical trial/ or randomized controlled trial/ or exp clinical trials as topic/ or randomized controlled trials as topic/ or Random Allocation/ or Double-Blind Method/ or Single-Blind Method/ or (clinical trial, phase i or clinical trial, phase ii or clinical trial, phase iii or clinical trial, phase iv or controlled clinical trial or randomized controlled trial or multicenter study or clinical trial).pt. or random*.ti,ab. or (clinic* adj trial*).tw. or ((singl* or doubl* or treb* or tripl*) adj (blind$3 or mask$3)).tw. or Placebos/ or placebo*.tw. | 2226544 |
| 6 | meta-analysis/ or meta-analysis as topic/ or (metaanaly* or meta-analy* or metanaly*).ti,ab,kf. or systematic review/ or cochrane.jw. or (prisma or prospero).ti,ab,kf. or ((systemati* or scoping or umbrella or "structured literature") adj3 (review* or overview*)).ti,ab,kf. or (systemic* adj1 review*).ti,ab,kf. or ((systemati* or literature or database* or data-base*) adj10 search*).ti,ab,kf. or ((structured or comprehensive* or systemic*) adj3 search*).ti,ab,kf. or ((literature adj3 review*) and (search* or database* or data-base*)).ti,ab,kf. or (("data extraction" or "data source*") and "study selection").ti,ab,kf. or ("search strategy" and "selection criteria").ti,ab,kf. or ("data source*" and "data synthesis").ti,ab,kf. or (medline or pubmed or embase or cochrane).ab. or ((critical or rapid) adj2 (review* or overview* or synthes*)).ti. or (((critical* or rapid*) adj3 (review* or overview* or synthes*)) and (search* or database* or data-base*)).ab. or (metasynthes* or meta-synthes*).ti,ab,kf. | 485243 |
| 5 | limit 4 to (english language and yr="2000 -Current") | 256 |
| 4 | 1 and (2 or 3) | 310 |
| 3 | exp "Anti-Inflammatory Agents, Non-Steroidal"/ or exp Lidocaine/ or exp Acetaminophen/ or (('non steroid*' or nonsteroid*) adj2 ('anti inflammatry' or antiinflammatory) adj2 (drug* or agent* or compound*)).ti,ab,kf. or lidocaine.ti,ab,kf. or paracetamol.ti,ab,kf. or acetaminophen*.ti,ab,kf. or 'diclofenac'.ti,ab,kf. or voltaren.ti,ab,kf. or naproxen.ti,ab,kf. or ibuprofen.ti,ab,kf. or exp Steroids/ or steroid*.ti,ab,kf. or exp "Adrenal Cortex Hormones"/ or ((cortex or cortical or cortico or adrenocortical) adj2 (hormone* or steroid*)).ti,ab,kf. or corticoid*.ti,ab,kf. or corticosteroid.ti,ab,kf. or cortisone.ti,ab,kf. or adrenocorticosteroid*.ti,ab,kf. or 'triamcinolone'.ti,ab,kf. or kenacort.ti,ab,kf. or methylprednisolone.ti,ab,kf. or Polidocanol/ or Polidocanol.ti,ab,kf. or Glycosaminoglycans/ or 'glycosaminoglycan polysul*ate'.ti,ab,kf. or exp Hyaluronic Acid/ or 'hyaluronic acid'.ti,ab,kf. or exp "Platelet-Rich Plasma"/ or 'platelet rich plasma'.ti,ab,kf. or 'thrombocyte rich plasma'.ti,ab,kf. or prp.ti,ab,kf. or 'autologous conditioned plasma'.ti,ab,kf. or acp.ti,ab,kf. or 'autologous blood injection*'.ti,ab,kf. or exp Prolotherapy/ or 'prolotherapy'.ti,ab,kf. or 'proliferation therapy'.ti,ab,kf. or 'regenerative injection therapy'.ti,ab,kf. | 1392099 |
| 2 | exp Drug Therapy/ or dt.fs. or ((drug or pharma* or medicament or medicinal) adj2 (treatment* or therap*)).ti,ab,kf. or pharmaco-therapy.ti,ab,kf. or pharmaco-treatment.ti,ab,kf. or pharmacotherapy.ti,ab,kf. or pharmacotreatment.ti,ab,kf. or exp Injections/ or injection*.ti,ab,kf. | 3478317 |
| 1 | ("jumper* knee" or "patella apicitis" or "patellar apicitis" or "patella apex syndrome" or "patellar apex syndrome" or "patella tip syndrome" or "patellar tip syndrome" or "patella tenosynovitis" or "patellar tenosynovitis").ti,ab,kf. or ((exp Patellar Ligament/ or exp Patella/ or "Patella".ti,ab,kf. or "patellar".ti,ab,kf.) and (Tendinopathy/ or "Tendinopathy".ti,ab,kf. or "Tendinopathies".ti,ab,kf. or "tendinosis".ti,ab,kf. or "tendinoses".ti,ab,kf. or "tendinitis".ti,ab,kf. or "Tendinitides".ti,ab,kf. or "tendonitis".ti,ab,kf. or "Tendonitides".ti,ab,kf. or "peritendinitis".ti,ab,kf. or "paratenonitis".ti,ab,kf. or ("tendon" and ("pathology" or "rupture" or "ruptures")).ti,ab,kf.)) | 2223 |

Table 7.2 Ovid/medline search

|  | **Embase** | **OVID/MEDLINE** | **Deduplicated** |
| --- | --- | --- | --- |
| SRs | 54 | 42 | 36 |
| RCT | 86 | 67 | 100 |
| Observational | 19 | 31 | 36 |
| Total | 159 | 140 | 200 |

Table 7.3 Search results

| 1. **Author and year** | 2. **Reason for exclusion** |
| --- | --- |
| 3. Aicale, 2020 | 4. Wrong study design (no systematic review) |
| 5. Andia, 2015 | 6. Included studies already included in more recent review. |
| 7. Andriolo, 2019 | 8. Included studies had wrong study design or wrong control group. |
| 9. Balasubramaniam, 2015 | 10. Included studies described wrong population or used wrong control group. |
| 11. Challoumas, 2019 | 12. Only one RCT describes PT, which is already included in our analysis. |
| 13. De Vos, 2010 | 14. Included studies had wrong design or described another population. |
| 15. Di Matteo, 2015 | 16. Included studies were already included in more recent review. |
| 17. Dragoo, 2014 | 18. Wrong control group used. |
| 19. Everhart, 2016 | 20. Included studies were already included in more recent review. |
| 21. Ferrero, 2012 | 22. Wrong study design (no RCT) |
| 23. Filardo, 2018 | 24. Included studies were already included in more recent review. |
| 25. Filardo, 2010 | 26. Wrong control group. |
| 27. Gaida, 2011 | 28. Included studies were already included in more recent review. |
| 29. Hoksrud, 2006 | 30. Wrong control group. |
| 31. Jeong, 2014 | 32. Included studies did not meet inclusion criteria for our analysis. |
| 33. Kaux, 2013 | 34. Wrong study design (no systematic review). |
| 35. Liddle, 2015 | 36. Included studies were already included in more recent review. |
| 37. Mautner, 2014 | 38. Wrong study design (no systematic review). |
| 39. Moreas, 2013 | 40. Included studies described wrong population or used another control group. |
| 41. Morath, 2020 | 42. One RCT met inclusion criteria, but was already described in included review. |
| 43. Nourissat, 2015 | 44. Wrong study design (no systematic review). |
| 45. Rigby, 2015 | 46. Wrong follow-up (two weeks). |
| 47. Sisk, 2020 | 48. Wrong study design (no systematic review). |
| 49. Taylor, 2020 | 50. Included studies described other population or were not RCTs. |
| 51. Van Rijn, 2019 | 52. Included studies were already included in more recent review. |
| 53. Van der Doelen, 2020 | 54. One RCT met inclusion criteria, but was already described in included review. |
| 55. Hart, 2011 | 56. Wrong study design (no systematic review). |

Table 7.4 Excluded studies

Onderkant formulier

| **Study reference** | **Study characteristics** | **Patient characteristics ^2^** | **Intervention (I)** | **Comparison / control (C) ^3^** | **Follow-up** | **Outcome measures and effect size ^4^** | **Comments** |
| --- | --- | --- | --- | --- | --- | --- | --- |
| Resteghini, 2016 | Type of study:  RCT  Setting and country:  Homerton Hospital Sports Medicine department.  Funding and conflicts of interest: The authors report no conflicts of interest. | Inclusion criteria:  - >18 years.  - Diagnosis of patellar tendinopathy (by ultrasound examination).  Exclusion criteria*:* Contraindications to injection: allergic reaction, possible local sepsis, concomitant anticoagulant therapy, peripheral vascular disease, <18 years old.  N total at baseline:  Intervention: 11  Control: 11  Important prognostic factors^2^:  *Age ± SD*  I: 38.9 ± 8.9  C: 42.5 ± 13.0  *Sex:* I: 90.0% M C: 72.7% M  *Duration of symptoms (mo ± SD)*  I: 16.7 ± 9.7  C: 19.2 ± 12.2  *Tendon grade (neovascularity ± SD)*  I: 2.9 ± 0.3  C: 2.5 ± 0.7  *Pain (mean VAS)* I: 7.1 C: 7.9  *Function (mean VISA-P)* I: 34.1 C: 19.6  Groups comparable at baseline?  n.r. | ABI:  The area of injection was prepared with chlorhexide spray, followed by an injection of 2 mL 1% lidocaine into subcutaneous tussue around the tendon to be injected. In the meantime, 2mL of autologous blood was withdrawn from the contralateral antecubital fossa using aseptic technique. Patients were asked to stay in the department for  20 minutes to monitor for adverse reactions.  Patients were advised on relative rest with normal  activities of daily living. Patients were advised that  the only analgesia permitted was paracetamol, and that no  form of nonsteroidal anti-inflammatory drug whether topical  or parental was permitted so as not to confound results. The ultrasound-guide ABI were given 2 occasions, 4 weeks apart. | Saline:  The syringe was covered to disguise the contents and an  identical syringe containing the same volume of control  substance of normal saline was drawn up. | Length of follow-up:  1 year (minimum).  Loss-to-follow-up:  I: 0  C: 0  Incomplete outcome data:  n.r. | Pain Measured with the visual analogue scale (VAS: 0 – 100)  Intervention: At 1 month: 4.5 At 3 months: 3.5 At 1 year: 3.1  Control: At 1 month: 4.5 At 3 months: 4.0 At 1 year: 3.3  Function  Measured with the Victoria institute of sport assessment for patellar tendinopathy scores (VISA-P)  Intervention: At 1 month: 50.7 At 3 months: 57.7 At 1 year: 62.5  Control: At 1 month: 39.2 At 3 months: 39.2 At 1 year: 48.6  Return to sports n.r.  Absence duration  n.r.  Satisfaction  n.r. | Limitations:  - The clinician injectingthe patient could have been able to differentiate whether saline or blood was being injected due to the viscosity difference when injecting. - Less then 28 patients were recruited, as were required for power calculations.  Conclusions:  This study demonstrated that both the ABI and saline  groups experienced a significant improvement in symptoms. However,  when the results were compared, there was no statistical  difference between the 2 groups.  Comments:  Baseline data of function differs. |
| Scott, 2019 | Type of study:  RCT  Setting and country:  Investigation performed at the University of Washington, Seattle, Washington, USA;  Olympiatoppen, Oslo, Norway; the Rizzolli Institute, Bologna, Italy;  and the University of British Columbia, Vancouver, British Columbia, Canada.  Funding and conflicts of interest:  Funding by an unrestricted research grant from the American Orthopeadic Society for Sports Medicine. Cytomedix provided free PRP preparation kits. Cytomedix provided a free loan of centrifugation equipment to the University of Washington and Norway. The following authors received funds: A.S., L.E, S.D.V., K.G.H, R.F.L., G.F., | Inclusion criteria:  All patients were between 18 and 50 years old and were diagnosed by the physician investigators using standard  diagnostic criteria for patellar tendinopathy. Symptoms had to be resent for at least 6 months, and patients had to have already attempted to resolve their conditions with exercise-based rehabilitation for a minimum of 6 weeks.  Exclusion criteria*:*  history of knee or patellar tendon surgery or any inflammatory or prominent degenerative joint condition affecting the knee (ie, physical examination and history  suggestive of steoarthritis or chondral lesions).  N total at baseline:  Intervention: LR-PRP: 19 LP-PRP: 19 Control: Saline: 19  Important prognostic factors^2^:  *Age ± SD*  LR-PRP: 32 ± 9.8 LP-PRP: 33 ± 7.3 C: 31 ± 7.9  *Sex:* LR-PRP: 94.7% M LP-PRP: 78.9% M C: 94.7% M  *Body mass index (kg/m2)* LR-PRP: 24 ± 3.3 LP-PRP: 26 ± 4.1 C: 24 ± 2.4  Groups comparable at baseline?  The 3 groups of 19 participants were demographically  and clinically similar. | LR-PRP/LP-PRP  Venous blood (52 mL) was drawn, added to 8 mL of anticoagulant citrate dextrose solution–solution A, and then run through the collection system. At 1 study site an  aliquot of the PRP was sent for hematological analysis by the local laboratory to determine the platelet and WBC  counts (n = 6, LR-PRP; n = 6, LP-PRP). The LR-PRP contained 230.000 platelets and the LP-PRP contained 227.000 platelets before centrifugation.  After injection of up to 3.5 mL of PRP, the patient lay quietly with the  knee extended for 15 minutes. Ice was applied for pain as  desired by the patient. For 48 hours after injection, patients  were instructed to refrain from exercise. After 1 week, rehabilitation  began.  All patients engaged in a supervised  gym-based rehabilitation program based on the slow heavy  loading program (concentric and eccentric) described by Kongsgaard et al.20 They trained in the rehabilitation facility 3 times per week for 6 weeks. At least 1 session per week was directly supervised by a physical therapist. | Saline:  The same volume of PRP and  saline (3.5 mL) was used to control for mechanical effects  of the injected fluid, and patients assigned to saline also had blood withdrawn (then discarded) to maintain participant  blinding.  After the injection the patient lay quietly with the  knee extended for 15 minutes. Ice was applied for pain as  desired by the patient. For 48 hours after injection, patients were instructed to refrain from exercise. After 1 week, rehabilitation  began.  All patients engaged in a supervised  gym-based rehabilitation program based on the slow heavy  loading program (concentric and eccentric) described by Kongsgaard et al.20 They trained in the rehabilitation facility 3 times per week for 6 weeks. At least 1 session per week was directly supervised by a physical therapist. | Length of follow-up:  6wk, 12 wk, 24 wk and 1year.  Loss-to-follow-up:  LR-PRP: 6 LP-PRP: 3 C: 3  Reasons: participants leaving the study  Incomplete outcome data:  Baseline: 1 6 wk: 2 12 wk: 2  24wk: 2 1 year: 2 | Pain Measured with the Numerical Rating Scale (0-100)  LR-PRP: - 6 wk: 3.6 ± 2.0 - 12 wk: 3.4 ± 1.9 - 24 wk: 3.3 ± 1.5 - 1 year: 4.0 ± 2.4  LP-PRP: - 6 wk: 4.0 ± 2.4 - 12 wk: 2.7 ± 2.1 - 24 wk: 2.1 ± 1.8 - 1 year: 2.4 ± 2.3  Control:  - 6 wk: 3.4 ± 2.2 - 12 wk: 2.9 ± 2.1 - 24 wk: 3.1 ± 2.1 - 1 year: 2.0 ± 1.9  Function  Measured with the VISA-P (1-100)  LR-PRP: - 6 wk: 55 ± 22 - 12 wk:63 ± 22 - 24 wk: 58 ± 22 - 1 year: 58 ± 29  LP-PRP: - 6 wk: 57 ± 24 - 12 wk: 67 ± 21 - 24 wk: 71 ± 19 - 1 year: 71 ± 20  Control:  - 6 wk: 63 ± 19 - 12 wk: 69 ± 18 - 24 wk: 74 ± 18 - 1 year: 80 ± 18  Return to sports  n.r.  Absence duration  n.r.  Satisfaction  Measured with the global rating of change (GROC) score.  LR-PRP: - 6 wk: 4.6 ± 1.2 - 12 wk: 4.9 ± 1.2 - 24 wk: 5.0 ± 1.3 - 1 year: 4.7 ± 1.6  LP-PRP: - 6 wk: 4.8 ± 1.7 - 12 wk: 5.3 ± 1.3 - 24 wk: 5.6 ± 1.3 - 1 year: 5.6 ± 1.0  Control:  - 6 wk: 5.0 ± 0.9 - 12 wk: 5.6 ± 1.0 - 24 wk: 5.4 ± 1.0 - 1 year: 5.7 ± 1.2 | Limitations:  The variability of treatment responses was higher than that accounted for in our sample size calculation, which leads to the possibility that the study is underpowered. Indeed, this is a relatively small clinical trial.  Conclusions:  When combined with an exercise-based  rehabilitation program, a single injection of LR-PRP or  LP-PRP was no more effective than saline for the improvement  of patellar tendinopathy symptoms.  Comments |
| Steunebrink, 2013 | Type of study:  RCT  Setting and country:  Department of Steunebrink  Sportsmedicine, Eelde, The  Netherlands  Department of  Sportsmedicine, University  Medical Center Groningen,  Groningen, The Netherlands  Department of  Sportsmedicine, Medical Center  Haaglanden, Leidschendam,  The Netherlands  Funding and conflicts of interest:  No competing interests. | Inclusion criteria:  presence of chronic patellar tendinopathy and age of 18–40 years. The diagnosis was based on clinical findings by an  experienced sports physician: all patients had a painful tendon  related to activity and/or thickened tendon on palpation. Symptoms had to be present for >3 months. MRI and ultrasound were not used. If patients were diagnosed with bilateral  patellar tendinopathy, only the most painful knee was included. The other knee was treated with the eccentric training programme, but not by using patches.  Exclusion criteria  (1) duration >24 months,  a Victorian Institute of Sports Assessment-Patella (VISA-P) score  of 80 or more, previous surgery or a local (corticosteroid)  injection of the knee; (2) previous performance of a complete heavy-load eccentric exercise programme of the knee in the last 2 years or inability to perform it because of serious illness; or (3) current pregnancy or other contra-indication for GTN-use.follow-up  N total at baseline:  I: 16 C: 17  Important prognostic factors^2^:  *Age ± SD*  I: 31.9 ± 9.6 C: 33.8 ± 10.5  *Sex:* I: 68.8% M C: 82.4% M  Groups comparable at baseline?  n.r. | GTN  The active patches (Minitran 5 glyceryltrinitrate  patch 3M Pharma Nederland BV) delivered 5 mg of  GTN every 24 h.  Patients were instructed on the use of the patches at the time of their initial visit. They were informed that the dosing regimen  called for one-quarter of a transdermal patch to be applied daily  to the affected patellar tendon. Each quarter patch was to be left  in situ for 24 h and then replaced with a new quarter patch. The patches were to be applied at the site of maximal tenderness  within a region of 1–2 cm around this point. The patients were instructed to rotate the application for the duration of the study in an attempt to avoid irritation of the skin. | Control  The placebo patches used were blister-plasters  (Kruidvat Renswoude BV).  Patients were instructed on the use of the patches at the time  of their initial visit. They were informed that the dosing regimen called for one-quarter of a transdermal patch to be applied daily  to the affected patellar tendon. Each quarter patch was to be left  in situ for 24 h and then replaced with a new quarter patch. The patches were to be applied at the site of maximal tenderness  within a region of 1–2 cm around this point. The patients were instructed to rotate the application for the duration of the study in an attempt to avoid irritation of the skin. | Length of follow-up:  after 6, 12 and 24 weeks.  Loss-to-follow-up:  GTN: - 6wk: 1 - 12 wk: 0 - 24 wk: 1  Control: - 6 wk: 1 - 12 wk: 0 - 24 wk: 2  Incomplete outcome data:  0 | Pain Measured with the VAS (0-10)  GTN:  - 6wk: 5.9 ± 3.1 - 12wk: 6.6 ± 2 - 24wk: 6.6 ± 3  Control:  - 6wk: 5.4 ± 3.3  - 12wk: 6.6 ± 3.7 - 24wk: 7.8 ± 3.1  Function  Measured with the VISA-P (0-100)  GTN: - 6wk: 64 ± 21.8 - 12wk: 73 ± 15.3 - 24wk: 75 ± 16.2  Control: - 6wk: 67.7 ± 15.6 - 12wk: 73.7 ± 20.7 - 24wk: 80.7 ± 22.1  Return to sports  n.r.  Absence duration  n.r.  Satisfaction  Measured with a 4 point scale (% patients who scored good/excellent)  GTN: - 6wk: 18.8% - 12wk: 56.3% - 24wk: 43.8%  Control: -6wk: 41.2% - 12wk: 52.9% - 24wk: 52.9% | Limitations:  It was difficult to have a No MRI and no ultrasound imaging were performed. This could be considered  a limitation of the study. It is well known, however, that there is limited correlation between clinical symptoms and ultrasound  or MRI,31 32 and that they are not the gold standard.  Another limitation of this study is that the tendon rehabilitation programme used in this clinical trial was home based and not  supervised.  Conclusions:  It seems that continuous topical GTN treatment in addition to an eccentric exercise programme does not improve clinical  outcome in patients with chronic patellar tendinopathy compared to placebo patches in combination with an eccentric exercise  programme.  Comments |

Table 7.5 Evidence table

| **Study reference**  (first author, publication year) | **Describe method of randomisation^1^** | **Bias due to inadequate concealment of allocation?^2^**  (unlikely/likely/unclear) | **Bias due to inadequate blinding of participants to treatment allocation?^3^**  (unlikely/likely/unclear) | **Bias due to inadequate blinding of care providers to treatment allocation?^3^**  (unlikely/likely/unclear) | **Bias due to inadequate blinding of outcome assessors to treatment allocation?^3^**  (unlikely/likely/unclear) | **Bias due to selective outcome reporting on basis of the results?^4^**  (unlikely/likely/unclear) | **Bias due to loss to follow-up?^5^**  (unlikely/likely/unclear) | **Bias due to violation of**  **intention to treat analysis?^6^**  (unlikely/likely/unclear) |
| --- | --- | --- | --- | --- | --- | --- | --- | --- |
| **Blood agent injections** | | | | | | | | |
| Resteghini, 2016 | Through a computer-  generated randomization program, practitioner A could  decipher whether this patient would receive autologous blood or normal saline. | Unlikely. | Unlikely. | Unclear  A limitation of our study was that despite our best  attempts to keep this double blind, the experienced clinician injecting the patient may have been able to differentiate whether saline or blood was being injected due to the viscosity difference when injecting. | Unclear  Not reported. | Unlikely. | Unlikely. | Unlikely. |
| Scott, 2019 | After the patient was registered, an allocation code was automatically generated (saline, LR-PRP, or LP-PRP). Randomization was created automatically by  Tenalea in balanced blocks of 6 (1:1:1 ratio), stratified by  age (18-34 and 36-50 years) and site. The participants were enrolled in and allocated to their treatment groups  by the study coordinator at each treatment site independent of the authors. | Unlikely. | Unlikely. | Likely. | Unlikely. | Unlikely. | Unlikely. | Unlikely. |
| **Passive transcutaneous medication** | | | | | | | | |
| Steunebrink, 2013 | Randomisation was performed using sealed opaque, identical envelopes. One unblinded sports physician (AW) coded the  envelopes with A or B. To ensure concealment of allocation, data on allocation were stored in a secret location. | Unlikely. | Unlikely. | Unclear. | Unlikely. | Unlikely. | Unlikely. | Unlikely. |

Table 7.6 Risk of bias table for intervention studies (randomized controlled trials)

1. Randomisation: generation of allocation sequences have to be unpredictable, for example computer generated random-numbers or drawing lots or envelopes. Examples of inadequate procedures are generation of allocation sequences by alternation, according to case record number, date of birth or date of admission.
2. Allocation concealment: refers to the protection (blinding) of the randomisation process. Concealment of allocation sequences is adequate if patients and enrolling investigators cannot foresee assignment, for example central randomisation (performed at a site remote from trial location) or sequentially numbered, sealed, opaque envelopes. Inadequate procedures are all procedures based on inadequate randomisation procedures or open allocation schedules..
3. Blinding: neither the patient nor the care provider (attending physician) knows which patient is getting the special treatment. Blinding is sometimes impossible, for example when comparing surgical with non-surgical treatments. The outcome assessor records the study results. Blinding of those assessing outcomes prevents that the knowledge of patient assignement influences the proces of outcome assessment (detection or information bias). If a study has hard (objective) outcome measures, like death, blinding of outcome assessment is not necessary. If a study has “soft” (subjective) outcome measures, like the assessment of an X-ray, blinding of outcome assessment is necessary.
4. Results of all predefined outcome measures should be reported; if the protocol is available, then outcomes in the protocol and published report can be compared; if not, then outcomes listed in the methods section of an article can be compared with those whose results are reported.
5. If the percentage of patients lost to follow-up is large, or differs between treatment groups, or the reasons for loss to follow-up differ between treatment groups, bias is likely. If the number of patients lost to follow-up, or the reasons why, are not reported, the risk of bias is unclear
6. Participants included in the analysis are exactly those who were randomized into the trial. If the numbers randomized into each intervention group are not clearly reported, the risk of bias is unclear; an ITT analysis implies that (a) participants are kept in the intervention groups to which they were randomized, regardless of the intervention they actually received, (b) outcome data are measured on all participants, and (c) all randomized participants are included in the analysis.

# Summary of literature

Lopez-Royo (2020) performed a systematic review and meta-analysis assessing the effectiveness of minimally invasive techniques in patients with chronic PT. A total of 10 RCTs, comprising 326 patients, were included in this review. Different minimally invasive techniques were evaluated: Dry needling, platelet-rich-plasma injections, corticosteroid injections, polidocanol injections, steroid injections, shockwave therapy and autologous blood injections. As a comparison, placebo injections, eccentric training and saline were used. To answer the clinical question, only the data from two RCTs was extracted from this review.

The RCT of Resteghini (2016) compared autologous blood injections (ABI) to saline injections in 22 patients with chronic PT. All patients were prepared with chlorhexidine spray, followed by an injection of 2 ml 1% lidocaine into subcutaneous tissue around the tendon. In the meantime, 2 ml of autologous blood was withdrawn from the contralateral antecubital fossa using aseptic technique. In the ABI group, autologous blood was injected into the tendon under ultrasound guidance. In the saline group, the same volume of saline was injected under ultrasound guidance. All patients were advised on relative rest with normal activities of daily living and were only permitted to take paracetamol as an analgesia. The injections were given on two occasions, four weeks apart. Patients were evaluated after one month, after three months and after one year. Authors assessed pain (VAS) and function (VISA-P).

Scott (2019) performed a parallel randomized single-blind saline-controlled study with three injection arms: Leukocyte-poor platelet-rich plasma injections (LP-PRP), leukocyte-rich platelet-rich plasma injections (LR-PRP) and saline injections in 57 athletes with PT. After injection the patient lay quietly with the knee extended for 15 minutes. Ice was applied for pain as desired by the patient. For 48 hours after injection, patients were instructed to refrain from exercise. After one week, rehabilitation began. All patients engaged in a supervised gym-based rehabilitation program based on the slow heavy loading program (concentric and eccentric). They trained in the rehabilitation facility three times per week for six weeks. At least 1 session per week was directly supervised by a physical therapist. Patients were evaluated after six weeks, 12 weeks, 24 weeks and one year. Authors assessed pain (NRS), function (VISA-P) and patient satisfaction (global rating of change GROC).

In addition to the RCTs included in the review of Lopez-Royo (2020), one separate RCT was added to the analysis (Steunebrink, 2013). Steunebrink (2013) performed a double-blind, placebo-controlled clinical trial comparing glyceryl trinitrate (GTN) treatment with placebo treatment in 33 patients with chronic PT. The researcher (MS) prepared packages of the patches in one envelope for every patient for treatment lasting 12 weeks. Two kinds of patches were used, active and placebo patches. The active patches delivered 5mg of GTN every 24 h. The placebo patches used were blister-plasters. Patients were instructed to avoid the use of co-interventions within the follow-up period. Patients were also given a supply of 20 acetaminophen tablets (500 mg) and were instructed to use them for any headache that they might experience. All patients completed a self-administered questionnaire, consisting of standardised outcome measures at baseline and after six, 12 and 24 weeks, authors assessed pain (VAS), function (VISA-P) and satisfaction.

# Results

As different types of interventions and controls were compared in the included studies, we divided the results in three comparisons:

- Blood agent injection (Resteghini, 2016; Scott, 2019).
- Passive transcutaneous medication (glyceryl trinitrate, GTN) (Steunebrink, 2013).

# Blood agent injections

Pain (critical)

Pain was measures with a visual analogue scale (VAS) or by the numeric rating scale (NRS) (0=no pain to 10=worst pain) in two studies included in the review (Resteghini, 2016; Scott, 2019), comprising 79 patients. Resteghini (2016) presented mean pain scores in the short-term (after three months) and in the long-term (after 12 months). Standard deviations were estimated based on figure 8.2 of the article for pooling the results. Scott (2019) also presented pain in the short-term and in the long-term (mean ± SD). Scott (2019) distinguished between leukocyte-rich platelet-rich plasma and leukocyte-poor platelet-rich plasma. In the short-term, data resulted in a MD of -0.31 (95% CI: -0.91 to 0.29), favoring the blood agent injection. This difference was not clinically relevant. In the long-term, data resulted in a MD of 0.60 (95% CI: -0.70 to 1.90). This difference was not clinically relevant.

Level of evidence of the literature

The level of evidence regarding the outcome pain started at high, because it was based on randomized controlled trials. In the short-term and in the long-term, the level of evidence was downgraded by three levels due to statistical heterogeneity (inconsistency, -1), limited number of included patients and crossing the border of clinical relevance (imprecision, -2). The final level is very low.

Function (critical)

Function was measured with the Victorian Institute of Sport Assessment-Patella (VISA-P) questionnaire (0=worst function to 100=best function) in two studies included in the review (Resteghini, 2016; Scott, 2019), comprising 79 patients. Resteghini (2016) presented mean function scores in the short-term (after three months) and in the long-term (after 12 months). Standard deviations were estimated based of the article for pooling the results. Scott (2019) presented function in the short-term and in the long-term (mean ± SD). Scott (2019) distinguished between leukocyte-rich platelet-rich plasma and leukocyte-poor platelet-rich plasma. Pooled data is shown in a forest plot (Figure 7.1). In the short-term, data resulted in a MD of 4.62 (95% CI: -13.18 to 22.41), favoring the blood agent injection. This difference was not clinically relevant. In the long-term, data resulted in a MD of -4.62 (95% CI: -27.72 to 18.47), favoring the saline injection. This difference was not clinically relevant.

Level of evidence of the literature

The level of evidence regarding the outcome function started at high, because it was based on randomized controlled trials. In the short-term and in the long-term, the level of evidence was downgraded by three levels due to inadequate blinding of care providers (-1; risk-of-bias), statistical heterogeneity (-1; inconsistency) and limited number of included patients (-1; imprecision). The final level is very low.

Figure 7.1 Pooled results blood agent injections and saline injections on pain

Function (critical)

Function was measured with the Victorian Institute of Sport Assessment-Patella (VISA-P) questionnaire (0=worst function to 100=best function) in two studies included in the review (Resteghini, 2016; Scott, 2019), comprising 79 patients. Resteghini (2016) presented mean function scores in the short-term (after three months) and in the long-term (after 12 months). Standard deviations were estimated based on figure 8.2 of the article for pooling the results. Scott (2019) presented function in the short-term and in the long-term (mean ± SD). Scott (2019) distinguished between leukocyte-rich platelet-rich plasma and leukocyte-poor platelet-rich plasma. Pooled data is shown in a forest plot (Figure 7.2). In the short-term, data resulted in a MD of 4.62 (95% CI: -13.18 to 22.41), favoring the blood agent injection. This difference was not clinically relevant. In the long-term, data resulted in a MD of -4.62 (95% CI: -27.72 to 18.47), favoring the saline injection. This difference was not clinically relevant.

Figure 7.2 Pooled results of blood agent injections and saline injections on function

Level of evidence of the literature

The level of evidence regarding the outcomes ‘return to sports/work’, ‘patient satisfaction’, ‘patient recovery’ and ‘duration of absenteeism’ were not assessed due to lack of studies.

# Conclusions

Pain (critical)

| **Very low**  **GRADE** | It is unclear what the effect of blood agent injections is on pain in the short-term and in the long-term.  *Sources: (Resteghini, 2016; Scott, 2019)* |
| --- | --- |

Function (critical)

| **Very low**  **GRADE** | It is unclear what the effect of blood agent injections is on function in the short-term and in the long-term.  *Sources: (Resteghini, 2016; Scott, 2019)* |
| --- | --- |

Duration of absenteeism (important)

| **-**  **GRADE** | The outcome measure ‘return to sports/work’, ‘patient satisfaction’, ‘patient recovery’ and ‘duration of absenteeism’ was not reported in the included studies. |
| --- | --- |

# Passive transcutaneous medication

Pain

Pain was measures with a visual analogue scale (VAS) (0=worst pain to 10=no pain) in one RCT (Steunebrink, 2013), comprising 33 patients. Steunebrink (2013) presented pain in the short-term (after three months) and in the long-term (after six months). In the short-term, data resulted in an equal score in both groups, MD of 0 (95% CI: -2.01 to 2.01). In the long-term, data resulted in a MD of -1.2 (95% CI: -3.31 to 0.91), favoring placebo. This difference was clinically relevant.

Level of evidence of the literature

The level of evidence regarding the outcome pain started at high, because it was based on a randomized controlled trial. In the short-term and in the long-term, the level of evidence was downgraded by two levels due to limited number of included patients and crossing the borders of clinical relevance (-2; imprecision). The final level is low.

Function

Function was measured with the Victorian Institute of Sport Assessment-Patella (VISA-P) questionnaire (0=worst function to 100=best function) in one RCT (Steunebrink, 2013), comprising 33 patients. Steunebrink (2013) presented function in the short-term (after three months) and in the long-term (after six months). In the short-term, data resulted in a MD of -0.70 (95% CI: -13.07 to 11.67), favoring placebo. In the long-term, data resulted in a MD of -5.7 (95% CI: -18.87 to 7.47), favoring placebo. These differences were not clinically relevant.

Level of evidence of the literature

The level of evidence regarding the outcome function started at high, because it was based on a randomized controlled trial. In the short-term and in the long-term, the level of evidence was downgraded by two levels due to limited number of included patients and crossing the borders of clinical relevance (-2; imprecision). The final level is low.

Return to sports/work

Return to sports/work was not reported in the included study.

Level of evidence of the literature

The level of evidence regarding the outcomes ‘return to sports’ was not assessed due to lack of studies.

Patient satisfaction

Patient satisfaction was measured with a subjective satisfaction score (rated as poor, fair, good or excellent) in one RCT (Steunebrink, 2013), comprising 33 patients. Steunebrink (2013) presented patients satisfaction as the number of patients who rated their satisfaction good or excellent in the short-term (after three months) and in the long-term (after six months). In the short-term, data resulted in a RR of 1.06 (95% CI: 0.57 to 1.98), favoring placebo. In the long-term, data resulted in a RR of 0.83 (95% CI: 0.40 to 1.69), favoring placebo. These differences were not clinically relevant.

Level of evidence of the literature

The level of evidence regarding the outcome patient satisfaction started at high because it was based on a randomized controlled trial. At six weeks, 12 weeks and 24 weeks, the level of evidence was downgraded by two levels due to limited number of included patients and crossing the borders of clinical relevance (-2; imprecision). The final level is low.

Patient recovery; Duration of absenteeism (important) were not reported in the included studies.

Level of evidence of the literature

The level of evidence regarding the outcomes ‘patient recovery’ and ‘duration of absenteeism’ were not assessed due to lack of studies.

# Conclusions

Pain (critical)

| **Low**  **GRADE** | The evidence suggests that passive transcutaneous medication (glyceryl trinitrate, GTN) results in little to no difference in pain in the short-term.  *Sources: (Steunebrink, 2013)* |
| --- | --- |

| **Low**  **GRADE** | The evidence suggests that passive transcutaneous medication (glyceryl trinitrate, GTN) may result in a slightly negative effect on return to sports in the long-term.  *Sources: (Steunebrink, 2013)* |
| --- | --- |

Function (critical)

| **Low**  **GRADE** | The evidence suggests that passive transcutaneous medication (glyceryl trinitrate, GTN) results in little to no difference in function in the short-term and in the long-term.  *Sources: (Steunebrink, 2013)* |
| --- | --- |

Return to sport/work, 3.5 Patient recovery and 3.6 Duration of absenteeism (important)

| **-**  **GRADE** | The outcome measure ‘return to sports/work’, ‘patient recovery’ and ‘duration of absenteeism’ was not reported in the included studies. |
| --- | --- |

Patient satisfaction

| **Low**  **GRADE** | The evidence suggests that passive transcutaneous medication (glyceryl trinitrate, GTN) results in little to no difference in patient satisfaction in the short-term and in the long-term.  *Sources: (Steunebrink, 2013)* |
| --- | --- |

# Considerations - from evidence to recommendation

Injections with blood

Based on the literature, injections with autologous blood, platelet-rich plasma and platelet-poor plasma do not appear to have a clinically relevant effect on the crucial outcome measures pain and function. The low to very low strength of evidence is mainly caused by the small numbers of patients and crossing the threshold of "no effect." The important outcome measures cannot contribute further to the direction of decision-making. Regarding these outcome measures, no literature was included in the abstract.

Passive transcutaneous medication.

Based on the literature, treatment with Glyceryl Trinitrate (GTN) seems to have only a small clinically relevant adverse effect on the crucial outcome measure pain after 12 months. There seems to be no clinically relevant effect on function. The low strength of evidence is mainly caused by the fact that only 1 RCT was found with few patients and crossing the threshold of "no effect." Also, there seems to be no clinically relevant effect on the important outcome measure patient satisfaction. The other important outcome measures cannot further contribute to the direction of decision making. Regarding these outcome measures, no literature was included in the abstract.

Active transcutaneous medication

In addition to passive transcutaneous medication, PT can also be treated with active transcutaneous medication. One example is iontophoresis. However, there were no studies describing this treatment that met the selection criteria. However, this treatment was investigated by an RCT with a two-week follow-up (Rigby, 2015). A clinically relevant positive effect was found on the crucial outcome measures pain, but not on function and the important outcome measures work absence/ sport, recovery, resumption of work/sport or patient satisfaction. However, a follow-up of two weeks is too short to determine whether the long-term (>1 month) effects of treatment persist.

Paracetamol (Acetaminophen) and NSAIDs.

No literature found.

NSAIDs, especially in the elderly, also have an unfavourable side effect. Another potential disadvantage of paracetamol (Acetaminophen) and NSAIDs is their short-term analgesic effect and therefore interference with the load recommendations advised by the expert panel. The use of these pain medications makes a pain scale less reliable and this may lead patients to engage in more loaded activities than can be tolerated by the patellar tendon at that time.

Injections of cortisone, with or without analgesia

No literature found.

The effect of cortisone injections has been studied in midportion Achilles tendinopathy (see Achilles tendinopathy guideline). There are indications this treatment is not effective in patients with midportion Achilles tendinopathy, it has an unfavourable long-term effect and there are problems with the safety of this treatment (especially with an increase in the number of injections) (Coombes, 2010; DaCruz, 1988; Seeger, 2006).

High volume injections with physiological saline

No literature found.

Prolotherapy

No literature found.

Summary

In summary, the evidence in the literature regarding the added value of pain medications for PT is extremely limited. The number of RCTs and systematic reviews is very low. All in all, no preferred intervention can be identified based on the literature. For the above reasons, the expert panel recommends caution in the use of paracetamol (Acetaminophen), NSAIDs and cortisone injections.

One RCT reported adverse effects of injections with blood (Scott, 2019). Eight of 38 patients described reduction on the 7-point global rating of change scale after six weeks. One patient could not participate in the rehabilitation program because of localized pain. No adverse effects are reported from active transcutaneous medication. However, one adverse effect is reported after passive transcutaneous medication, namely itching (n=3). However, headache and contact dermatitis are common adverse effects after treatment with GTN according to previous studies (Steunebrink, 2013).

The expert panel refers to the guideline 'Conservative treatment of osteoarthritis in hip or knee' for the main side effects of paracetamol, oral NSAIDs and dermal NSAIDs. In addition, the expert panel refers to the Pharmacotherapeutic Compass (also available as an app) for the description of medications and possible side effects.

Expectations and values of patients (and possibly their caretakers)

In order to gain more insight into the values and expectations of patients with PT, a questionnaire was prepared and distributed in cooperation with the Netherlands Patients Federation (NPF). Of the 9 PT patients who completed the questionnaire, 2 patients have had some form of medication (anti-inflammatory n = 1, injection therapy n = 1). None of the patients received pain medications. The main benefit of choosing anti-inflammatory medication is to reduce inflammation of the tendon (n=1) and no benefit has yet been noticed for injection therapy (n=1).

The expert panel concludes that medicinal therapy is used in PT, but no further conclusions can be drawn due to the small sample size. Pain medications does not provide demonstrable benefits in the treatment of PT. Alertness is required to the side effects of some medication. The expert panel noticed that patients do not see pain medications as a long-term solution.

Cost

No studies have been performed on the (cost) effectiveness of medication of PT. The cost of pain medications (paracetamol, oral and dermal NSAIDs) is low. Somewhat higher costs are associated with use of oral NSAIDs and the comedication with a gastric protector, but this should not prevent short-term use, according to the expert panel.

An intra-articular injection of autologous platelets (PRP) is not covered by most health insurance companies. To perform such a treatment, the practitioner will need centrifuge equipment, a sterile work place, and it has to be guaranteed that only patient-specific material is used; as a result, the treatment is currently not possible at every clinic or practice.

An intra-articular cortisone injection is covered within the basic package by health insurance companies and the injected product is not very expensive.

Acceptability, feasibility and implementation

Medicamentous treatment of PT in the form of NSAIDs and injection therapy is used in practice despite the absence of evidence based literature. NSAIDs are readily available, but PRP injections are not widely available and because of the very limited evidence base, this is not recommended by the expert panel. All other forms of pain medications in PT have not been sufficiently studied and therefore cannot be recommended either.

# Rationale of the recommendations

The limited number of low quality studies do not allow the recommendation of a specific pain medications. The expert panel does not recommend the use of pain medications to treat patients with PT. The expert panel recommends starting with exercise therapy and possibly additional conservative therapy (see module on exercise therapy PT and module on additional conservative treatments PT).

For severe pain, short-term use of oral or dermal pain medications may be considered. If the use of NSAIDs is nevertheless considered, preference is given to the drug with the fewest side effects and the lowest cost, as there is no evidence that one NSAID is superior to another.

There are no studies regarding the effectiveness of paracetamol (Acetaminophen) in reducing pain in patients with PT, but given its better safety profile compared to NSAIDs, this is the preferred option. The evidence in the literature regarding the added value of medicative treatments is extremely limited. The number of RCTs and systematic reviews is very low and they cover three different interventions. All in all, no intervention can be preferred on the literature. Medication treatment does not provide demonstrable benefits in the treatment of PT.

Patients with PT may experience daily pain and as a result be limited in their daily functioning. They are looking for a quick solution and prescribing medication is sometimes insurmountable in these situations. However, one must be aware medication can also have disadvantages; dependence and/or overload due to pain suppression. In exceptional situations with a lot of pain and limitations, short-term use of pain medications can be considered. Based on possible side effects, the expert panel prefers 1st choice paracetamol (Acetaminophen) and 2nd choice dermal NSAID. The patient must be informed that medication is not a solution. The expert panel recommends starting with exercise therapy and, if necessary, additional conservative treatment. The expert panel does not recommend the use of medication for the treatment of patients with PT.

# References

López-Royo MP, Ortiz-Lucas M, Gómez-Trullén EM, Herrero P. (2020). The Effectiveness of Minimally Invasive Techniques in the Treatment of Patellar Tendinopathy: A Systematic Review and Meta-Analysis of Randomized Controlled Trials*. Evid Based Complement Alternat Med*.:8706283.

Resteghini P, Khanbhai TA, Mughal S, Sivardeen Z. (2016) Double-Blind randomized controlled trial. *Clinical Journal of Sport Medicine*;26(1): 17–23.

Rigby JH, Mortensen BB, Draper DO. Wireless Versus Wired Iontophoresis For Treating Patellar Tendinopathy: A Randomized Clinical Trial. J*ournal of Athletic Training*;50(11):1165–1173.

Scott A, LaPrade RF, Harmon KG et al. (2019) Platelet-rich plasma for patellar tendinopathy: a randomized controlled trial of leukocyte-rich PRP or leukocyte-poor PRP versus saline. *American Journal of Sports Medicine*;47(10): 1654–1661.

Steunebrink M, Zwerver J, Brandsema R, Groeneboom P, Akker-Scheek I, Weir A. (2013) Topical glyceryl trinitrate treatment of chronic patellar tendinopathy: a randomised, double-blind, placebo-controlled clinical trial. *Br J Sports Med*;47, 34–39.

# Construction of Module 8 ‘Open surgery for PFP and PT’

Scoping Question: What is the value of open surgery for patients with PFP and PT?

# Search and select

A systematic review of the literature was performed to answer the following question: What is de effect of open surgery in patients with PFP and in patients with PT on pain, self-reported knee function, IKDC, stability and range of motion?

PICO 1

P:	patients with PFP

I:	open surgery

C:	conservative treatment or no treatment

O:	pain (VAS, NRS), self-reported knee function (Kujala, KOOS, IKDC (subjective), BANFF, Norwich score etc.), IKDC (objective), stability and range of motion.

PICO 2

P:	patients with PT

I:	open surgery

C:	conservative treatment or no treatment

O:	pain (VAS, NRS), self-reported knee function (Kujala, KOOS, IKDC (subjective), BANFF, Norwich score), IKDC (objective), stability, range of motion, return to sports/work, and satisfaction

Relevant outcome measures

The expert panel considered pain (measured with VAS or NRS) as a critical outcome measure for decision making; and self-reported knee function (measured with Kujala, KOOS, IKDC (subjective), BANFF, Norwich score), return to work, return to sports and satisfaction as important outcome measures for decision making.

A priori, the expert panel did not define the outcome measures listed above but used the definitions used in the studies. The expert panel defined a difference of 2 cm (of 10 cm) on the VAS or 2 categories on the NRS scale as a minimal clinically (patient) important difference, according to Crossley, 2004. Additionally, a difference of 13 points in the VISA score was seen as a minimal clinically important difference (Hernandez-Sanchez, 2014).

Search and select (Methods)

The databases Medline (via OVID) and Embase (via Embase.com) were searched with relevant search terms until 16-11-2020. The detailed search strategy is depicted in the tables. The systematic literature search resulted in 373 hits. Studies were selected based on the following criteria; systematic reviews (searched in at least two databases, and detailed search strategy, risk of bias assessment and results of the individual studies available) and randomized controlled trials (RCTs) that included at least 20 patients with PFP or PT, compared open surgery with a control group of conservative treatment or no treatment, and included at least one of the defined outcome measures. A total of five studies were initially selected based on title and abstract screening. After reading the full text, four studies were excluded (Table 8.4), and one study was included.

Results

One systematic review was included in the analysis of the literature. Important study characteristics and results are summarized in the evidence tables. The assessment of the risk of bias is summarized in the risk of bias tables.

Key articles

1. Surgery for patellar tendinopathy (jumper's knee). [Dan M](https://www.ncbi.nlm.nih.gov/pubmed/?term=Dan%20M%5BAuthor%5D&cauthor=true&cauthor_uid=31546279)1, [Phillips A](https://www.ncbi.nlm.nih.gov/pubmed/?term=Phillips%20A%5BAuthor%5D&cauthor=true&cauthor_uid=31546279), [Johnston RV](https://www.ncbi.nlm.nih.gov/pubmed/?term=Johnston%20RV%5BAuthor%5D&cauthor=true&cauthor_uid=31546279), [Harris IA](https://www.ncbi.nlm.nih.gov/pubmed/?term=Harris%20IA%5BAuthor%5D&cauthor=true&cauthor_uid=31546279).

2. How does surgery compare to sham surgery or physiotherapy as a treatment for tendinopathy? A systematic review of randomised trials. [Challoumas D](https://www.ncbi.nlm.nih.gov/pubmed/?term=Challoumas%20D%5BAuthor%5D&cauthor=true&cauthor_uid=31191975)1, [Clifford C](https://www.ncbi.nlm.nih.gov/pubmed/?term=Clifford%20C%5BAuthor%5D&cauthor=true&cauthor_uid=31191975)1,2, [Kirwan P](https://www.ncbi.nlm.nih.gov/pubmed/?term=Kirwan%20P%5BAuthor%5D&cauthor=true&cauthor_uid=31191975)3,4, [Millar NL](https://www.ncbi.nlm.nih.gov/pubmed/?term=Millar%20NL%5BAuthor%5D&cauthor=true&cauthor_uid=31191975)1.

3. Results of Surgical Treatment of Chronic Patellar Tendinosis (Jumper's Knee): A Systematic Review of the Literature. [Matthias Brockmeyer](https://pubmed.ncbi.nlm.nih.gov/?term=Brockmeyer+M&cauthor_id=26248496) [1](https://pubmed.ncbi.nlm.nih.gov/26248496/#affiliation-1), [Nora Diehl](https://pubmed.ncbi.nlm.nih.gov/?term=Diehl+N&cauthor_id=26248496) [1](https://pubmed.ncbi.nlm.nih.gov/26248496/#affiliation-1), [Cornelia Schmitt](https://pubmed.ncbi.nlm.nih.gov/?term=Schmitt+C&cauthor_id=26248496) [1](https://pubmed.ncbi.nlm.nih.gov/26248496/#affiliation-1), [Dieter M Kohn](https://pubmed.ncbi.nlm.nih.gov/?term=Kohn+DM&cauthor_id=26248496) [1](https://pubmed.ncbi.nlm.nih.gov/26248496/#affiliation-1), [Olaf Lorbach](https://pubmed.ncbi.nlm.nih.gov/?term=Lorbach+O&cauthor_id=26248496) [2](https://pubmed.ncbi.nlm.nih.gov/26248496/#affiliation-2)

4. Current Concepts and the Role of Surgery in the Treatment of Jumper’s Knee. [Casey R Stuhlman](https://pubmed.ncbi.nlm.nih.gov/?term=Stuhlman+CR&cauthor_id=27482730), [Kris Stowers](https://pubmed.ncbi.nlm.nih.gov/?term=Stowers+K&cauthor_id=27482730), [Lucas Stowers](https://pubmed.ncbi.nlm.nih.gov/?term=Stowers+L&cauthor_id=27482730), [Jay Smith](https://pubmed.ncbi.nlm.nih.gov/?term=Smith+J&cauthor_id=27482730)

5. Open versus arthroscopic surgical treatment of chronic proximal patellar tendinopathy. A systematic review. 2013 Marcheggiani Muccioli

| **No.** | **Query** | **Results** |
| --- | --- | --- |
| #11 | #4 NOT (#8 OR #9 OR #10) = overige studies | 189 |
| #10 | #4 AND #7 NOT (#8 OR #9) = observationeel | 81 |
| #9 | #4 AND #6 NOT #8 = RCT | 45 |
| #8 | #4 AND #5 = SR | 21 |
| #7 | 'major clinical study'/de OR 'clinical study'/de OR 'case control study'/de OR 'family study'/de OR 'longitudinal study'/de OR 'retrospective study'/de OR 'prospective study'/de OR 'cohort analysis'/de OR cohort*:ab,ti OR (('case control' NEAR/1 (study OR studies)):ab,ti) OR (('follow up' NEAR/1 (study OR studies)):ab,ti) OR (observational NEAR/1 (study OR studies)) OR ((epidemiologic NEAR/1 (study OR studies)):ab,ti) OR (('cross sectional' NEAR/1 (study OR studies)):ab,ti) | 5747479 |
| #6 | 'clinical trial'/exp OR 'randomization'/exp OR 'single blind procedure'/exp OR 'double blind procedure'/exp OR 'crossover procedure'/exp OR 'placebo'/exp OR 'prospective study'/exp OR rct:ab,ti OR random*:ab,ti OR 'single blind':ab,ti OR 'randomised controlled trial':ab,ti OR 'randomized controlled trial'/exp OR placebo*:ab,ti | 3159901 |
| #5 | 'meta analysis'/exp OR 'meta analysis (topic)'/exp OR metaanaly*:ti,ab OR 'meta analy*':ti,ab OR metanaly*:ti,ab OR 'systematic review'/de OR 'cochrane database of systematic reviews'/jt OR prisma:ti,ab OR prospero:ti,ab OR (((systemati* OR scoping OR umbrella OR 'structured literature') NEAR/3 (review* OR overview*)):ti,ab) OR ((systemic* NEAR/1 review*):ti,ab) OR (((systemati* OR literature OR database* OR 'data base*') NEAR/10 search*):ti,ab) OR (((structured OR comprehensive* OR systemic*) NEAR/3 search*):ti,ab) OR (((literature NEAR/3 review*):ti,ab) AND (search*:ti,ab OR database*:ti,ab OR 'data base*':ti,ab)) OR (('data extraction':ti,ab OR 'data source*':ti,ab) AND 'study selection':ti,ab) OR ('search strategy':ti,ab AND 'selection criteria':ti,ab) OR ('data source*':ti,ab AND 'data synthesis':ti,ab) OR medline:ab OR pubmed:ab OR embase:ab OR cochrane:ab OR (((critical OR rapid) NEAR/2 (review* OR overview* OR synthes*)):ti) OR ((((critical* OR rapid*) NEAR/3 (review* OR overview* OR synthes*)):ab) AND (search*:ab OR database*:ab OR 'data base*':ab)) OR metasynthes*:ti,ab OR 'meta synthes*':ti,ab | 682078 |
| #4 | (#1 OR #2) AND #3 AND ([english]/lim OR [dutch]/lim) AND [1996-2020]/py NOT ('conference abstract'/it OR 'editorial'/it OR 'letter'/it OR 'note'/it) | 336 |
| #3 | 'open surgery'/exp OR (open:ti,ab,kw AND ('surgery'/exp OR 'surgeon'/exp OR surger*:ti,ab,kw OR surgical*:ti,ab,kw OR surgeon*:ti,ab,kw OR operation*:ti,ab,kw OR operative*:ti,ab,kw OR incisi*:ti,ab,kw OR extracti*:ti,ab,kw OR excisi*:ti,ab,kw OR invasive*:ti,ab,kw OR restorati*:ti,ab,kw)) OR 'osteotomy'/exp OR osteotom*:ti,ab,kw OR artrotom*:ti,ab,kw OR trochleaplast*:ti,ab,kw OR 'tibial tuber* transposition*':ti,ab,kw OR maquet:ti,ab,kw OR fulkerson:ti,ab,kw | 300971 |
| #2 | 'patellar tendinopathy'/exp OR 'jumper* knee':ti,ab,kw OR 'patella apicitis':ti,ab,kw OR 'patellar apicitis':ti,ab,kw OR 'patella apex syndrome':ti,ab,kw OR 'patellar apex syndrome':ti,ab,kw OR 'patella tip syndrome':ti,ab,kw OR 'patellar tip syndrome':ti,ab,kw OR 'patella tenosynovitis':ti,ab,kw OR 'patellar tenosynovitis':ti,ab,kw OR (('patellar ligament'/exp OR 'patella'/exp OR 'patella':ti,ab,kw OR 'patellar':ti,ab,kw) AND ('tendinitis'/exp OR 'tendinopathy':ti,ab,kw OR 'tendinopathies':ti,ab,kw OR 'tendinosis':ti,ab,kw OR 'tendinoses':ti,ab,kw OR 'tendinitis':ti,ab,kw OR 'tendinitides':ti,ab,kw OR 'tendonitis':ti,ab,kw OR 'tendonitides':ti,ab,kw OR 'peritendinitis':ti,ab,kw OR 'paratenonitis':ti,ab,kw OR ('tendon':ti,ab,kw AND ('pathology':ti,ab,kw OR 'rupture':ti,ab,kw OR 'ruptures':ti,ab,kw)))) | 2856 |
| #1 | 'patellofemoral pain syndrome'/exp OR 'patellofemoral pain':ti,ab,kw OR 'patellofemoral syndrome':ti,ab,kw OR 'patellofemoral dysfunction*':ti,ab,kw OR 'patellofemoral disorder*':ti,ab,kw OR 'pfp*':ti OR 'pfps':ti OR ptfp:ti OR 'retro-patellar pain':ti,ab,kw OR 'retropatellar pain':ti,ab,kw OR 'peri-patellar pain':ti,ab,kw OR 'anterior knee pain':ti,ab,kw OR 'patella chondropath*':ti,ab,kw OR 'patellar chondropath*':ti,ab,kw OR 'patella chrondromalacia*':ti,ab,kw OR 'patellar chrondromalacia*':ti,ab,kw OR 'runner s knee':ti,ab,kw OR 'lateral facet compression syndrome':ti,ab,kw OR 'chondromalacia patella*':ti,ab,kw | 5172 |

Table 8.1 Embase search

| **#** | **Searches** | **Results** |
| --- | --- | --- |
| 12 | 5 not (9 or 10 or 11) = Overige studies | 128 |
| 11 | (5 and 8) not (9 or 10) = Observationeel | 110 |
| 10 | (5 and 7) not 9 = RCT | 28 |
| 9 | 5 and 6 = SR | 19 |
| 8 | Epidemiologic studies/ or case control studies/ or exp cohort studies/ or Controlled Before-After Studies/ or Case control.tw. or cohort*.tw. or Cohort analy$.tw. or (Follow up adj (study or studies)).tw. or (observational adj (study or studies)).tw. or Longitudinal.tw. or Retrospective*.tw. or prospective*.tw. or consecutive*.tw. or Cross sectional.tw. or Cross-sectional studies/ or historically controlled study/ or interrupted time series analysis/ | 3703772 |
| 7 | exp clinical trial/ or randomized controlled trial/ or exp clinical trials as topic/ or randomized controlled trials as topic/ or Random Allocation/ or Double-Blind Method/ or Single-Blind Method/ or (clinical trial, phase i or clinical trial, phase ii or clinical trial, phase iii or clinical trial, phase iv or controlled clinical trial or randomized controlled trial or multicenter study or clinical trial).pt. or random*.ti,ab. or (clinic* adj trial*).tw. or ((singl* or doubl* or treb* or tripl*) adj (blind$3 or mask$3)).tw. or Placebos/ or placebo*.tw. | 2225728 |
| 6 | meta-analysis/ or meta-analysis as topic/ or (metaanaly* or meta-analy* or metanaly*).ti,ab,kf. or systematic review/ or cochrane.jw. or (prisma or prospero).ti,ab,kf. or ((systemati* or scoping or umbrella or "structured literature") adj3 (review* or overview*)).ti,ab,kf. or (systemic* adj1 review*).ti,ab,kf. or ((systemati* or literature or database* or data-base*) adj10 search*).ti,ab,kf. or ((structured or comprehensive* or systemic*) adj3 search*).ti,ab,kf. or ((literature adj3 review*) and (search* or database* or data-base*)).ti,ab,kf. or (("data extraction" or "data source*") and "study selection").ti,ab,kf. or ("search strategy" and "selection criteria").ti,ab,kf. or ("data source*" and "data synthesis").ti,ab,kf. or (medline or pubmed or embase or cochrane).ab. or ((critical or rapid) adj2 (review* or overview* or synthes*)).ti. or (((critical* or rapid*) adj3 (review* or overview* or synthes*)) and (search* or database* or data-base*)).ab. or (metasynthes* or meta-synthes*).ti,ab,kf. | 484819 |
| 5 | limit 4 to (english language and yr="1996 -Current") | 285 |
| 4 | (1 or 2) and 3 | 379 |
| 3 | (open.ti,ab,kf. and (exp Surgical Procedures, Operative/ or orthopedics/ or exp Orthopedic Procedures/ or surgery.fs. or surger*.ti,ab,kf. or surgical*.ti,ab,kf. or surgeon*.ti,ab,kf. or operation*.ti,ab,kf. or operative*.ti,ab,kf. or incisi*.ti,ab,kf. or extracti*.ti,ab,kf. or excisi*.ti,ab,kf. or invasive*.ti,ab,kf. or restorati*.ti,ab,kf.)) or exp Osteotomy/ or osteotom*.ti,ab,kf. or artrotom*.ti,ab,kf. or trochleaplast*.ti,ab,kf. or 'tibial tuber* transposition*'.ti,ab,kf. or maquet.ti,ab,kf. or Fulkerson.ti,ab,kf. | 212298 |
| 2 | ("jumper* knee" or "patella apicitis" or "patellar apicitis" or "patella apex syndrome" or "patellar apex syndrome" or "patella tip syndrome" or "patellar tip syndrome" or "patella tenosynovitis" or "patellar tenosynovitis").ti,ab,kf. or ((exp Patellar Ligament/ or exp Patella/ or "Patella".ti,ab,kf. or "patellar".ti,ab,kf.) and (Tendinopathy/ or "Tendinopathy".ti,ab,kf. or "Tendinopathies".ti,ab,kf. or "tendinosis".ti,ab,kf. or "tendinoses".ti,ab,kf. or "tendinitis".ti,ab,kf. or "Tendinitides".ti,ab,kf. or "tendonitis".ti,ab,kf. or "Tendonitides".ti,ab,kf. or "peritendinitis".ti,ab,kf. or "paratenonitis".ti,ab,kf. or ("tendon" and ("pathology" or "rupture" or "ruptures")).ti,ab,kf.)) | 2223 |
| 1 | exp "Patellofemoral Pain Syndrome"/ or "Patellofemoral Pain".ti,ab,kf. or 'Anterior Knee Pain'.ti,ab,kf. or 'Patellofemoral Syndrome'.ti,ab,kf. or 'patellofemoral dysfunction*'.ti,ab,kf. or 'patellofemoral disorder*'.ti,ab,kf. or 'PFP'.ti,ab,kf. or 'PFP*'.ti,ab,kf. or ptfp.ti,ab,kf. or 'retro-patellar pain'.ti,ab,kf. or 'retropatellar pain'.ti,ab,kf. or 'peri-patellar pain'.ti,ab,kf. or 'anterior knee pain'.ti,ab,kf. or 'Patella chondropath*'.ti,ab,kf. or 'Patellar chondropath*'.ti,ab,kf. or 'Patella chrondromalacia*'.ti,ab,kf. or 'Patellar chrondromalacia*'.ti,ab,kf. or 'runner* knee'.ti,ab,kf. or 'lateral facet compression syndrome'.ti,ab,kf. or 'chondromalacia patella*'.ti,ab,kf. | 6865 |

Bovenkant formulier

Table 8.2 Pubmed search

|  | **Embase** | **OVID/MEDLINE** | **Deduplicated** |
| --- | --- | --- | --- |
| SRs | 21 | 19 | 23 |
| RCT | 45 | 28 | 47 |
| Observational | 81 | 110 | 97 |
| Other | 189 | 128 | 206 |
| Total | 336 | 285 | 373 |

Table 8.3 Search results

| **Author and year** | **Reason for exclusion** |
| --- | --- |
| Brockmeyer, 2015 | Only review discribted studies, no RCTs. |
| Muccioli, 2013 | Review with only 1 RTC, also describted in included study (Dan, 2019). |
| Saltzman, 2017 | Descripted study no control group |
| Bahr, 2006 | RCT, describted included study (Dan, 2019) |

Table 8.4 Excluded studies

| **Study reference** | **Study characteristics** | **Patient characteristics** | **Intervention (I)** | **Comparison / control (C)** | **Follow-up** | **Outcome measures and effect size** | **Comments** |
| --- | --- | --- | --- | --- | --- | --- | --- |
| Dan, 2019 | SR and meta-analysis of RTC’s  *Literature search up to July 2018*  **A**: Bahr, 2006  Study design: RCT parallel  Setting and Country:  **A**: n.r. Norway  Source of funding and conflicts of interest:  **A**: funding from Norwegian Eastern Health Corporate, Royal Norwegian Ministry of Culture, Norwegian Olympic Committee and Confederation of Sport, Norsk Tipping AS, and Pfizer AS. | Inclusion criteria SR:  *Trials were eligible if they compared surgical techniques (open or arthroscopic) with placebo surgery, exercise or other non-surgical modalities.*  Exclusion criteria SR:  *No RTC / no surgical intervention.*  *1 study included*  Important patient characteristics at baseline per intervention group:  N, mean age  **A**: 1) 20 patients, 30 yrs 2) 20 patients, 31 yrs.  Sex:  **A**: 1) 85% male 2) 90% male  Duration of symptoms: **A.** 1) 25 months 2) 33 months  Training hours **A.** 1) 12,2 2) 11,2  Pain (VAS-score) **A.** 1) 4.3 2) 3.9  Function (VISA-score) **A:** 1) 31 2) 29  Groups comparable at baseline? Yes: p=0.15 to 0.85. | Describe intervention:  **A**: Open surgical excision: Surgery involved a 5 cm longitudinal midline incision from the inferior pole of the patella distally. A tourniquet was not used. The paratenon was split longitudinally and any pathologic paratenon tissue was removed. The tendon was split longitudinally in the midline to expose the deepest layers, and tissue that appeared abnormal was removed, or if not seen macroscopically the area calculated from the MRI was removed. Once the sutures were removed the participants were exposed to the same physiotherapist and eccentric exercises as the non-operative group. Two surgeons performed the operations. Prior to starting the eccentric exercises participants gradually increased activities, focusing on knee range-of-motion and walking without crutches. | Describe control:  **A**: Eccentric exercise: Involved use of a 25-degree decline board at home, where the downward (eccentric) component was performed on the affected leg and the upward (concentric) component was performed on the asymptomatic leg. The participant was instructed to perform the squat to 90 degrees with the back in a vertical position. The patient was instructed to take two seconds for the eccentric component. It involved twice-daily sessions of three sets of 15 repetitions for a total duration of 12 weeks, with weekly supervision by a physiotherapist. No warm-up was performed. Six weeks after surgery, participants were exposed to the same rehabilitation/eccentric exercises programme as the eccentric exercise group, except they were not allowed to tolerate pain. | End-point of follow-up:  **A**: 6 months and 12 months  Loss-to-follow-up 6 months: **A:** 2 (10%): Intervention group, 3 (15%) in the control group. Reasons: n.r.  Loss-to-follow-up 12 months: **A:** 0 (0%) in the intervention group), 5 (25%) in the control group. Reasons: secondary surgery after failing eccentric training (final score before surgery was carried forward  For how many participants were no complete outcome data available?  (intervention/control)  **A:** n.r. | Outcome measure pain  (12 months)  Defined as mean pain scores with standing jump measured on a 10-point VAS scale.  Effect measure: mean difference (95% CI):  **A**: -0.4 (-1.2 to 0.4)  Outcome measure function  (6 months and 12 months ) Defined as mean function on the 100-point VISA-P scale.  Effect measure: mean difference (95% CI):  **A (6 months)**: 3.2 (-9.5 - 16) **A (12 months):** 7.17 (-4.45 -18.79)  Outcome measure IKDC  **A.** n.r.  Outcome measure stability **A:** n.r.  Outcome measure range of motion  **A:** n.r.  Outcome measure return to sports/work (12 months)  Defined a four-point categorical scale, where 0 is no sport, 1 (reduced level),  2 (full training but some symptoms), 3 (full training and no symptoms).  Effect measure: Risk ratio (95%CI): **A:** 0.83 (0.3 - 2.29)  Outcome measure satisfaction (12 months) Defined as the number of people with no pain.  Effect measure: Mean difference (95%CI): **A:** 0.71 (0.27 - 1.88) | Author’s conclusion:  We are uncertain if surgery is beneficial over other therapeutic interventions, namely eccentric exercises or injectables. Low-certainty evidence shows that surgery for patellar tendinopathy may not provide clinically important benefits over eccentric exercise in terms of pain, function or participant-reported treatment success, but may provide clinically meaningful pain reduction and treatment success when compared with sclerosing injections. However, further research is likely to change these results. The evidence was downgraded two levels due to the small sample sizes and susceptibility to bias. We are uncertain if there are additional risks associated with surgery as study authors failed to report adverse events. Surgery seems to be embedded in clinical practice for late-stage patellar tendinopathy, due to exhaustion of other therapeutic methods rather than evidence of benefit.  Level of evidence: GRADE  Outcome measures for pain, function, return to sports and satisfaction were low (downgraded 2 levels for imprecision). The outcome measures stability and range of motion were not reported.  Sensitivity analyses: Our planned subgroup analysis to assess if any differences in outcome occurred with arthroscopic or open surgical excision could not be performed as we did not identify more than one study with a common comparator. As we were unable to conduct a meta-analysis, we did not perform any sensitivity analyses. |

Table 8.6 Evidence table for systematic review of RCTs and observational studies (intervention studies)

Table 8.7 Quality assessment for systematic reviews of RCTs and observational studies

Based on AMSTAR checklist (Shea et al.; 2007, BMC Methodol 7: 10; doi:10.1186/1471-2288-7-10) and PRISMA checklist (Moher et al 2009, PLoS Med 6: e1000097; doi:10.1371/journal.pmed1000097)

| **Study**  **First author, year** | **Appropriate and clearly focused question?^1^**  **Yes/no/unclear** | **Comprehensive and systematic literature search?^2^**  **Yes/no/unclear** | **Description of included and excluded studies?^3^**  **Yes/no/unclear** | **Description of relevant characteristics of included studies?^4^**  **Yes/no/unclear** | **Appropriate adjustment for potential confounders in observational studies?^5^**  **Yes/no/unclear/notapplicable** | **Assessment of scientific quality of included studies?^6^**  **Yes/no/unclear** | **Enough similarities between studies to make combining them reasonable?^7^**  **Yes/no/unclear** | **Potential risk of publication bias taken into account?^8^**  **Yes/no/unclear** | **Potential conflicts of interest reported?^9^**  **Yes/no/unclear** |
| --- | --- | --- | --- | --- | --- | --- | --- | --- | --- |
| Dan, 2019 | Yes | Yes | Yes | Yes | Not applicable | Yes | No | Yes | Yes |

1. Research question (PICO) and inclusion criteria should be appropriate and predefined
2. Search period and strategy should be described; at least Medline searched; for pharmacological questions at least Medline + EMBASE searched
3. Potentially relevant studies that are excluded at final selection (after reading the full text) should be referenced with reasons
4. Characteristics of individual studies relevant to research question (PICO), including potential confounders, should be reported
5. Results should be adequately controlled for potential confounders by multivariate analysis (not applicable for RCTs)
6. Quality of individual studies should be assessed using a quality scoring tool or checklist (Jadad score, Newcastle-Ottawa scale, risk of bias table etc.)
7. Clinical and statistical heterogeneity should be assessed; clinical: enough similarities in patient characteristics, intervention and definition of outcome measure to allow pooling? For pooled data: assessment of statistical heterogeneity using appropriate statistical tests (e.g. Chi-square, I^2^)?
8. An assessment of publication bias should include a combination of graphical aids (e.g., funnel plot, other available tests) and/or statistical tests (e.g., Egger regression test, Hedges-Olken). Note: If no test values or funnel plot included, score “no”. Score “yes” if mentions that publication bias could not be assessed because there were fewer than 10 included studies.
9. Sources of support (including commercial co-authorship) should be reported in both the systematic review and the included studies. Note: To get a “yes,” source of funding or support must be indicated for the systematic review AND for each of the included studies.

# Summary of literature

The Cochrane systematic review (Dan, 2019) described the effects of open surgery on pain, function and recovery in adolescents and adults with PT. A total of 2 parallel randomized studies, comprising 92 participants, were included in this review. Different comparisons were included: Open surgical excision versus eccentric exercises, and surgery (arthroscopic) vs sclerosing injection. To answer the clinical question, only the data from one study included in the comparison ‘Open surgical excision versus eccentric exercises’ was extracted from this review (Bahr, 2006). This study included open surgery and a control group with eccentric exercises.

|  | **Intervention group** | **Control group** |
| --- | --- | --- |
| Bahr (2006) | Open surgical excision:  Surgery involved a 5 cm longitudinal midline incision from the inferior pole of the patella distally. A tourniquet was not used. The paratenon was split longitudinally and any pathologic paratenon tissue was removed. The tendon was split longitudinally in the midline to expose the deepest layers, and tissue that appeared abnormal was removed, or if not seen macroscopically the area calculated from the MRI was removed. Once the sutures were removed the participants were exposed to the same physiotherapist and eccentric exercises as the non-operative group. Two surgeons performed the operations. Prior to starting the eccentric exercises participants gradually increased activities, focusing on knee range-of-motion and walking without crutches. | Eccentric exercises:  Involved use of a 25-degree decline board at home, where the downward (eccentric) component was performed on the affected leg and the upward (concentric) component was performed on the asymptomatic leg. The participant was instructed to perform the squat to 90 degrees with the back in a vertical position. The patient was instructed to take two seconds for the eccentric component. It involved twice-daily sessions of three sets of 15 repetitions for a total duration of 12 weeks, with weekly supervision by a physiotherapist. No warm-up was performed. Six weeks after surgery, participants were exposed to the same rehabilitation/eccentric exercises programme as the eccentric exercise group, except they were not allowed to tolerate pain. |

# Table 8.5 Intervention type and control group

# Results

PICO 1:

No studies were found which could be used answering PICO 1.

PICO 2:

Pain (critical)

Pain was measured using a 10-point visual analogue scale (VAS), where a higher score means more pain.

Pain with standing jump after 12 months was presented in the selected study in the review (Bahr, 2006; n=40). The data resulted in a mean difference (MD) of -0.4 (95%CI:-1.18 - 0.38), favoring open surgical excision. The difference was not large enough to reach the border of clinical relevance.

Level of evidence of the literature

The level of evidence regarding the outcome pain (after 12 months) started at high as it was based on a randomized controlled trial, but was downgraded by 2 levels due to limited number of included patients (imprecision, -2). The final level is low.

Function (important)

Function was measured by a 100-point VISA scale, where a higher score means better function.

Function (measured at 6 months) was presented in the selected study in the review (Bahr, 2006; n=38). The data resulted in a mean difference (MD) of 3.24 (95% CI: -9.52 - 16), favoring open surgical excision. The difference was not large enough to reach the border of clinical relevance.

Function (measured at 12 months) was presented in the selected study in the review (Bahr, 2006; n=40). The data resulted in a mean difference (MD) of 7.17 (95% CI: -4.45 - 18.79), favoring open surgical excision. The difference was not large enough to reach the border of clinical relevance.

Level of evidence of the literature

The level of evidence regarding the outcome function (after 6 and 12 months) started at high as it was based on a randomized controlled trial, but was downgraded by 2 levels due to limited number of included patients (imprecision, -2). The final level is low.

Stability (important)

Stability was not described as an outcome in the included studies. The level of evidence regarding the outcome stability was not assessed due to lack of studies.

Range of motion (important)

Range of motion was not described as an outcome in the included studies. The level of evidence regarding the outcome range of motion was not assessed due to lack of studies.

Return to sports (important)

Return to sports was measured by a 4-point category scale, where 0 is no sport, 1(reduced level), 2 (full training but some symptoms), 3 (full training and no symptoms) after 12 months.

The return to sports score after 12 months was presented in the selected study in the review (Bahr, 2006; n=40). The data resulted in a risk ratio (RR) of 0.83 (95% CI 0.3 - 2.29), favoring open eccentric exercises. The difference was not large enough to reach the border of clinical relevance.

Level of evidence of the literature

The level of evidence regarding the outcome return to sports after 12 months started at high as it was based on a randomized controlled trial, but was downgraded by 2 levels due to limited number of included patients and crossing the line of clinical relevance (imprecision, -2). The final level is low.

Satisfaction (important)

Satisfaction was measured as the proportion with no symptoms after 6 months and 12 months. The satisfaction score after 6 months was presented in the selected study in the review (Bahr, 2006; n=38). The data resulted in a mean difference (MD) of -1.05 (95% CI -2.24 -0.14), favoring eccentric exercises. The satisfaction score after 12 months was presented in the selected study in the review (Bahr, 2006; n=40). The data resulted in a mean difference (MD) of -0.21 (-0.84 - 1.26), favoring open surgery.

Level of evidence of the literature

The level of evidence regarding the outcome pain (after 12 months) started at high as it was based on a randomized controlled trial, but was downgraded by 2 levels due to study limited number of included patients (imprecision, -2). The final level is low.

Conclusions
PICO 1: No conclusions were formulated for this PICO.

PICO 2:

Pain at 12 months (critical)

| **low GRADE** | The evidence suggests that there is probably no difference in pain after 12 months when comparing open surgery and eccentric exercises.  *Sources: Dan, 2019 (Bahr, 2006)* |
| --- | --- |

Function at 6 months (important)

| **low GRADE** | The evidence suggests that there is probably no difference in function after 6 months when comparing open surgery and eccentric exercises.  *Sources: Dan, 2019 (Bahr, 2006)* |
| --- | --- |

Function at 12 months (important)

| **low GRADE** | The evidence suggests that there is probably no difference in function after 12 months when comparing open surgery and eccentric exercises.  *Sources: Dan, 2019 (Bahr, 2006)* |
| --- | --- |

Stability (important)

| **- GRADE** | The outcome measure stability was not reported in the included study.  *Sources: -* |
| --- | --- |

Range of motion (important)

| **- GRADE** | The outcome measure range of motion was not reported in the included study.  *Sources: -* |
| --- | --- |

Return to sports at 12 months (important)

| **low GRADE** | The evidence suggests that there is probably no difference in return to sports after 12 months when comparing open surgery to eccentric exercises.  *Sources: Dan, 2019 (Bahr, 2006)* |
| --- | --- |

Satisfaction at 6 months (important)

| **low GRADE** | The evidence suggests that there is probably no difference in satisfaction after 6 months when comparing open surgery to eccentric exercises.  *Sources: Dan, 2019 (Bahr, 2006)* |
| --- | --- |

Satisfaction at 12 months (important)

| **low GRADE** | The evidence suggests that there is probably no difference in satisfaction after 12 months when comparing open surgery to eccentric exercises.  *Sources: Dan, 2019 (Bahr, 2006)* |
| --- | --- |

# Considerations - from evidence to recommendation PFP

No literature was included regarding this intervention. However, retrospective and cohort studies do show a significant effect of surgery (tibial tubercle osteotomy) in the domains of 'pain and function' in the short term (3 months) and in the medium term (2 years and 10 years) (Saltzman, 2017; Middleton, 2019; Koëter, 2007; Tigchelaar, 2015; Ding, 2016). Patients with only PFP score better than patients with patellar dislocations.

In practice, surgery is exceptionally considered when conservative (nonoperative) treatment is not sufficiently effective. Many scopic procedures were previously performed, but arthroscopy for PFP has no place, as previously described in the guideline arthroscopy of the knee (https://richtlijnendatabase.nl/richtlijn/artroscopie_van_de_knie/artroscopische_behandeling_van_knieklachten/artroscopie_bij_patellofemoraal_pijnsyndroom.html). If a possible anatomic cause has been identified with imaging, an osteotomy around the knee can be considered as a method to improve patellar tracking and thus reduce pain and improve functional load capacity.

The expert panel realizes this application in the clinical setting, although the evidence for it is lacking. It cannot be ruled out that observed improvements are based on a placebo effect. Surgery may involve side effects or complications. The expert panel believes that this should be considered in the decision.

Expectations and values of patients (and possibly their caretakers)

To gain more insight into the values and expectations of patients with PFP, a questionnaire was prepared and distributed in collaboration with the Netherlands Patients Federation (NPF). The questionnaire was completed by 43 patients with PFP (21 who have had it and 22 who currently have it). 13% of respondents (n=8) have had surgical treatment. Patients would prefer to be able to function normally again (as soon as possible) in daily life, in addition, being able to move and play sports again is considered important. Patients would like to be informed about the possibilities or impossibilities of surgical treatment and the post-operative (physiotherapeutic) rehabilitation. In the responses to this questionnaire, a number of advantages and disadvantages are mentioned regarding the treatment. Many patients mention that recovery after surgery is long (n=4). The outcome of the surgery varies, six out of eight patients are very satisfied or satisfied with the surgery, two patients are dissatisfied.

Cost

There are costs associated with surgery. No economic analysis is available on the costs of this intervention or whether they outweigh the benefits (cost-effectiveness). The costs are relatively high compared with conservative treatment. Surgery is generally covered by health insurance (subject to the deductible).

Acceptability, feasibility and implementation.

Surgical treatment is very rarely used in clinical practice when conservative treatment proves ineffective. Possible objection is that there is no RCT describing the effectiveness of surgical intervention. Good knowledge of surgical options, likelihood of success, risks of complications and side effects are essential in good decision making. For this, a setting with sufficient expertise in performing adequate diagnostics and experience of these surgeries is important.

Objections that may be raised by the patient probably lie mainly in access to this care. Although it is regular care, expertise in performing these procedures is not present in every hospital. If expertise with diagnostics, indication and surgical treatment is lacking, referral to a specialized center by the physician is appropriate. This expertise is not equally present in all regions so patients sometimes have to travel far. According to the expert panel, there are enough healthcare providers in the Netherlands to meet the demand.

# Rationale of the recommendations

If conservative therapy is ineffective or inadequate, surgery may be considered to achieve reduction in pain and improvement in function (Rixe, 2013). This will be done in consultation with the patient where the unpredictable outcome and risks of complications of surgery should be discussed. An indication for surgery can only be made if there is an anatomical substrate demonstrable with imaging. This always requires radiological examination. Conventional examination is almost always done first in the Netherlands. Additionally, an MRI or CT scan can be made, looking for posttraumatic, degenerative as well as congenital causes.

# Considerations - from evidence to recommendation PT

Regarding this intervention, one study was included. Regarding the crucial outcome measure pain, no clinically relevant effect of surgery was demonstrated. No other crucial outcome measures were defined. The strength of evidence for all crucial and important outcome measures is therefore low, so the overall strength of evidence is low. The reason is twofold: (1) the very small study population and (2) failure to cross the line of clinical relevance. The expert panel concludes that no unequivocal opinion can be given on the preference for open surgery or eccentric exercises based on the crucial outcome measure pain. There are no subgroups for which the effects of the intervention or the quality of evidence are different because only one study was selected (Dan, 2019).

In practice, surgery is considered primarily when complaints in PT are therapy-resistant to conservative (non-operative) treatments. In the open procedure, the (diseased) tendinopathic tissue is excised with the goal of tissue regeneration. The procedure is not extensive or invasive, but, as with any surgical treatment, there is a risk of complications. The risks are small in absolute terms but greater in relative terms than with conservative or expectant management.

Cost

There are costs associated with surgery. No economic analysis is available on the costs of this intervention, nor whether they outweigh the benefits (cost-effectiveness). The expert panel estimate is that the costs for surgery with postoperative rehabilitation will be higher than for rehabilitation alone. Surgery is generally covered by health insurance (subject to deductible).

Since the existing literature shows no clinically relevant improvement in multiple domains (including pain, patient satisfaction, function and return to sport), the greater complication rate and cost will weigh heavily in the expert panels recommendation.

Expectations and preferences of patients (and possibly their caretakers).

The questionnaire administered in cooperation with the Netherlands Patients Federation (NPF) shows no respondents who have been treated surgically for PT. The questionnaire did show that patients would like to be informed about the possibilities or impossibilities of a surgical treatment option and the (physiotherapeutic) treatment path after surgery. Patients often do not initially have a preference for surgery, however if there are long-term therapy-resistant symptoms they may consider surgery.

Acceptability, feasibility and implementation.

One RCT comparing surgical treatment with eccentric exercise therapy is available (Bahr, 2016) reporting no clinically relevant difference between surgery and exercise therapy. Based on this literature, no distinction can be made between the effect of surgery and exercise therapy for PT. Since the costs and complication rates for surgery with postoperative exercise therapy are estimated to be higher than for exercise therapy alone, initial exercise therapy is preferred. In clinical practice, surgical intervention for PT is considered only in patients with chronic PT where conservative therapy have resulted in an unsatisfactory outcome. This will be done in consultation with the patient where the unpredictable outcome and risks of complications of surgery should be discussed. The knowledge and experience of specialists in the surgical treatment of PT varies by hospital and region. As a result, a patient with PT may receive different advice by region or treating physician.

# Rationale of the recommendations

It is unclear whether the diagnosis of chronic PT can be made with sufficient sensitivity and specificity based on history and physical examination (see module Diagnosing PT). Therefore, based on its expertise, the expert panel recommends before deciding on possible surgical intervention, an MRI should be performed to confirm the diagnosis and also exclude other relevant pathology. In addition, for planning the surgery, it is useful to properly map the exact location and extent of the affected tendon tissue using MRI.

# References

Bahr, R., Fossan, B., Loken, S., Engebretsen, L. (2006) Surgical treatment compared with eccentric training for patellar tendinopathy (Jumper's Knee). A randomized, controlled trial. *Journal of Bone* *and Joint Surgery* - *American volume*; 88(8), 1689-98.

Crossley, K.M., Bennell, K.L., Cowan, S.M., Green, S. (2004). Analysis of outcome measures for persons with patellofemoral pain: which are reliable and valid? *Archives of Physical Medicine & Rehabilitation*; 85(5):815-22.

Dan, M., Phillips, A., Johnston, R.V., Harris, I.A. (2019). Surgery for patellar tendinopathy	(jumper’s knee). *Cochrane Database of Systematic Reviews*, Issue 9. Art. No.:	CD013034.

Ding, D.Y., Kanevsky, R., Strauss, E.J., Jazrawi, L.M. (2016). Anteromedialisation tibial tubercle osteotomy for recurrent patellar instability in young active patients: A retrospective case series. *LM.Injury*; 47(3):737-41.

Hernandez-Sanchez S, Hidalgo MD, Gomez A. Responsiveness of the VISA-P scale for patellar tendinopathy in athletes. (2014). *Br J Sports Med*.; 48(6):453-7.

Koëter, S., Diks, M.J., Anderson, P.G., Wymenga, A.B. (2007). A self-centring osteotomy of the tibial tubercle for patellar maltracking or instability: results with ten-years' follow-up. *A.Bone Joint J.*; 97-B(3):329-36.

Middleton, K.K., Gruber, S., Shubin Stein, B.E. (2019). A modified tibial tubercle osteotomy for patellar maltracking: results at two years. *Sports Med Arthrosc Rev.* ;27(4):154-160.

Rixe, J.A., Glick, J.E., Bradym. J., Olympia, R.P. (2013). A review of the management of PFPS. *Phys Sportsmen*; 41(3): 19-28.

Saltzman, B.M., Rao, A., Erickson, B.J., Cvetanovich, G.L. Levy, D., Bach, B.R., Cole, B.J. (2017). A Systematic Review of 21 Tibial Tubercle Osteotomy Studies and More Than 1000 Knees: Indications, Clinical Outcomes, Complications, and Reoperations. *Am J Orthop (Belle Mead NJ)*; 46(6): E396-E407.
